# Nucleophilic Substitution of 1,3-Diiodobicyclo[1.1.1]pentane: Synthesis of Bicyclo[1.1.1]pentylpyridinium, Quinolinium, Isoquinolinium, and Pyrazolium Salts

**DOI:** 10.1021/acs.joc.5c00565

**Published:** 2025-05-27

**Authors:** Harvey J. C. Monroe, Dolapo J. Bello, Bradley J. Duff, Mark R. J. Elsegood, Kohei Watanabe, Gareth J. Pritchard, Marc C. Kimber

**Affiliations:** † Department of Chemistry, School of Science, 5156 Loughborough University, Ashby Road, Loughborough LE11 3TU, U.K.; ‡ Faculty of Education, Chiba University, 1-33, Yayoi-cho, Inage-ku, Chiba 263-8522, Japan

## Abstract

In this study, we describe the synthesis of bicyclo[1.1.1]­pentane salts by the nucleophilic reaction of 1,3-diodobicyclo[1.1.1]­pentane (DIBCP) with several classes of nucleophiles. The bicyclo[1.1.1]­pentane fragments are established isosteres for *^t^* butyl, alkynyl, and 1,4-diaryl structural units, whose synthesis is typically achieved by addition to the unstable, cryogenically stored, [1.1.1]­propellane precursor. In contrast, DIBCP is a stable crystalline solid, with the potential to be a feedstock in the synthesis of BCP fragments. This work provides a straightforward, practical synthetic route to bicyclo[1.1.1]­pentylpyridinium, quinolinium, isoquinolinium and pyrazolium salts. This transformation displays a broad substrate scope, good yield profile, with several of the BCP products being fully characterized by single-crystal X-ray crystallography. The reaction proceeds by nucleophilic substitution on 1,3-diodobicyclo[1.1.1]­pentane (DIBCP), and we provide detailed computational analysis, showing the role of two nucleophiles in stabilizing a key carbocation intermediate. The synthesized salts are isosteres of existing arylpyridinium and arylquinolinium salts used within pharmaceuticals and high-value commodity chemicals within the industrial chemical sector. Finally, the synthetic utility of these salts is examined, providing practical synthetic routes to *N*-pyridin-4-one and *N*-quinolin-4-one substituted bicyclo[1.1.1]­pentanes.

## Graphical abstract

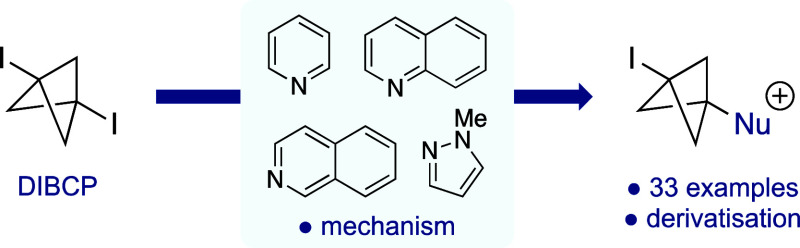

## Introduction

The bicyclo[1.1.1]­pentane (**1**, BCP) is a recognized isostere for the *^t^* butyl group, internal alkynes, and disubstituted arenes (Figure 1).^1–3^ The appeal of the BCP unit lies in its unique structure with a core comprised solely of sp^3^ carbons, providing a unique 3-dimensional structure with an increased fraction of *sp* ^3^-hybridized carbons (F*sp* ^3^) compared with traditional flat aromatic fragments.^4^ This has seen its inclusion as a bioisostere in several drug scaffolds,^1a,2,3c^ and its use in developing novel materials^5^ and ligands for catalysis.^6^

**Figure 1:**
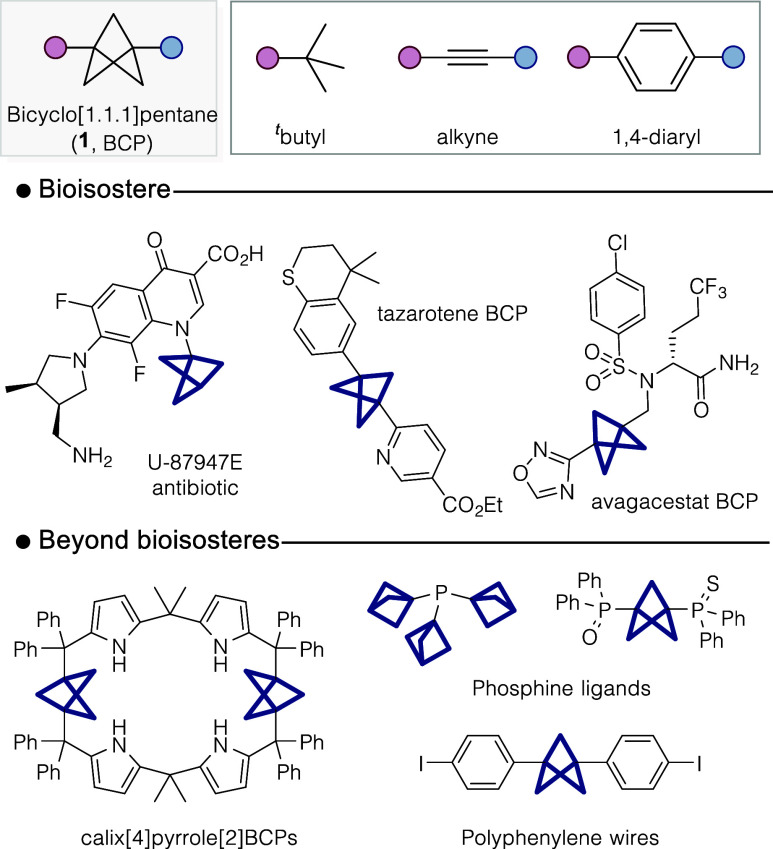
Bicyclo­[1.1.1]­pentane and its use as a bioisostere and application in materials chemistry and ligand design.

Three synthetic approaches have been explored to address the demand for multisubstituted BCP fragments as isosteres in materials chemistry and drug discovery. The first two strategies involve radical or anionic addition to [1.1.1]­propellane (**2**),^7a,7b^ with the third approach being electrophilic activation of **2**, with subsequent nucleophilic addition.^7c^

These three approaches rely on access to **2**, which is synthesized from commercially available dichloride **3** (Scheme 1).^8^ This is a well-established synthetic route and can be transferred into a continuous flow setting.^9^ However, the practical isolation and storage of the product [1.1.1]­propellane (**2**) can be problematic; it is isolated as a solution in ethereal solvents, it must be cryogenically stored, and should be used before its rapid degradation. In contrast, 1,3-diiodo[1.1.1]­bicyclopentane (**4**) (DIBCP) is a stable crystalline solid, and results from the treatment of [1.1.1]­propellane (**2**) with iodine (Scheme 1). It is principally observed as an unwanted byproduct, whose formation is keenly suppressed in the synthesis of functionalized BCPs.

**Scheme 1:**
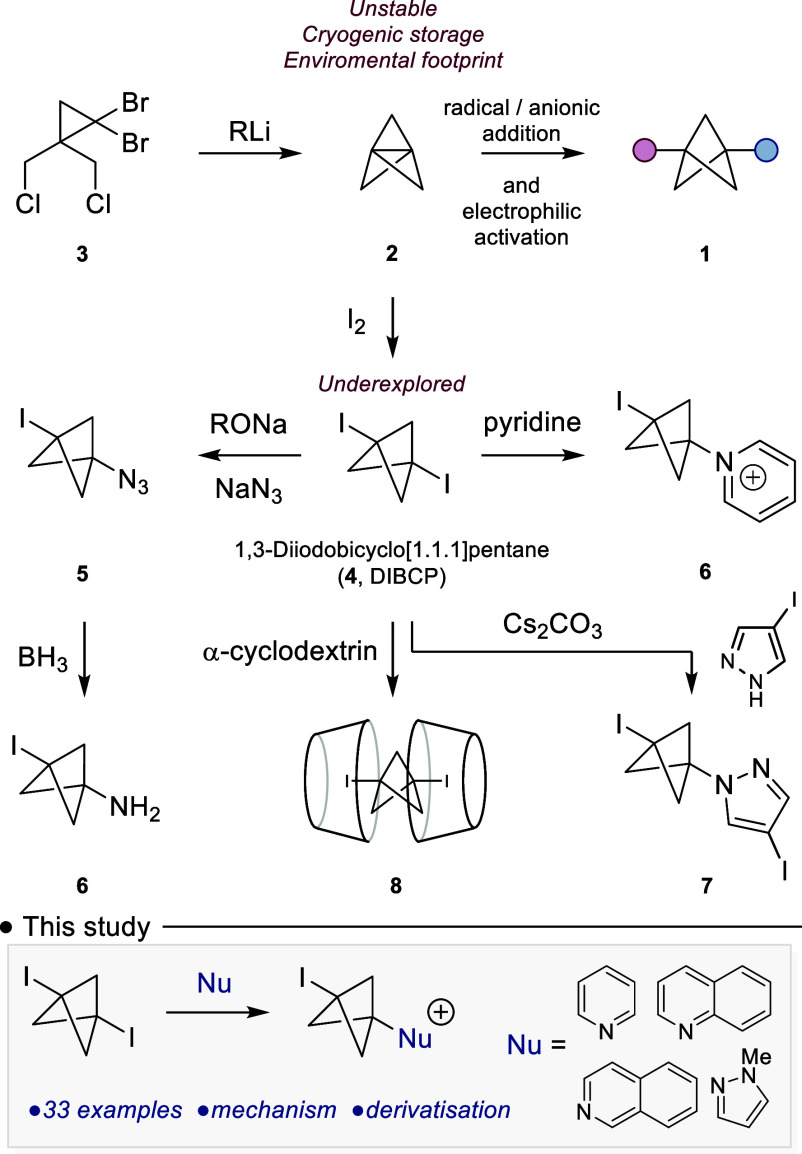
Synthesis of Disubstituted Bicyclo[1.1.1]­pentanes

The use of **4** as a precursor in synthesizing substituted BCPs is sparse within the literature. Wiberg and co-workers discovered that treatment of **4** with alkoxides and NaN_3_ delivered the azido BCP **5**.^10a^ Subsequent work by Hossain and co-workers demonstrated **5** could be reduced to the amine building block **6**,^10b^ although in this work **5** was synthesized by treatment of [1.1.1]­propellane **2** with IN_3_. Adcock and co-workers examined the addition of pyridine (and tertiary amines) to **4**, with the transformation being described as a nucleophilic addition, affording a new class of pyridinium BCP analogues (**6**), though in this disclosure, the scope of the transformation was limited.^11^ More recently, **4** has been used as a precursor to [1.1.1]­propellane (**2**), as exemplified by Zarate and co-workers use of **4** in their synthesis of the pyrazole BCP **7**,^12^ and Uchiyama and co-workers who examined **4** as a storable feedstock of [1.1.1]­propellane through its inclusion within α-cyclodextrins (**8**).^13^

Therefore, given the limited investigations of using **4** as a BCP feedstock, we sought to re-examine the reaction of **4** with a comprehensive range of pyridine nucleophiles as well as unexplored heteroarenes including quinolines, isoquinolines and pyrazoles. Additionally, we wished to explore the synthetic value of these BCP products to access material and medicinal relevant heteroaryl isosteres. Finally, a mechanism for the nucleophilic addition to **4** is proposed, which incorporates detailed computational analysis.

## Results and Discussion

We commenced this study^14^ by focusing on the pyridine scope (Scheme 2). This would provide a direct route into bicyclo[1.1.1]­pentylpyridinium salts, conceivable isosteres of valuable arylpyridiniums.^15^ The starting DIBCP (**4**) was synthesized by treatment of [1.1.1]­propellane with iodine. Caution should be exercised when synthesizing 1,3-diiodobicyclo[1.1.1]­pentane given its potential impact sensitivity, although its thermal sensitivity is lower than that of related cubane derivatives.^16^ With **4** in hand, we began by using the original conditions reported by Adcock and co-workers;^11b^ however, we found that we could reduce the amount of the nucleophile to 5 equiv with very little impact in isolated yield. Pyridine reacted well with **4** to give the pyridinium salt **10a** in 77% isolated yield, an improvement on the current literature (67%). Substitution at the 4-position of the pyridine ring followed the expected reactivity profile. Electron donating groups methyl, methoxy and phenyl provided the salts **10b**, **10f** and **10g** in good to excellent isolated yields. The structure of the 4-phenylpyridinium BCP salt (**10g**) was confirmed by single crystal X-ray analysis.^17^ Electron withdrawing groups at the 4-position of the pyridine ring, such as carboxyethyl, nitrile and trifluoromethyl, all provided the pyridinium BCP salts **10c**, **10d** and **10e**, but in modest isolated yields. Again, single crystal X-ray analysis confirmed the structures of the 4-ethylcarboxy pyridinium BCP salt (**6c**) and 4-methoxy pyridinium BCP salt (**10f**), respectively.^17^ The reaction conditions proved accepting of substitution at the 3-position of the pyridine ring, with electron-donating and withdrawing groups being tolerated, with pyridinium salts **10h**–**n** being isolated in good to excellent isolated yields. Substituents adjacent to the pyridinyl nitrogen were tolerated with the addition 2-methylpyridine providing salt **10o** in 69% yield. Disubstituted pyridines such as 2,4-lutidine and 3,5-lutidine gave the pyridinium BCP salts **10p** and **10q** in 86 and 73% yield, respectively. This was also extended to the 2,3-cyclopentapyridine, giving the pyridinium BCP salt **10r** in excellent isolated yields. 2,3,5-Trimethylpyridine added to **4**, providing the pyridinium salt **10s** in an excellent 92% isolated yield; 3,3-bipyridine provided the mono BCP salt **10t** exclusively, whereas the reaction of DMAP with **4** gave the BCP salt **10u** in 17% yield, where the only alkylation product detected, as confirmed by NOE, occurred on the pyridine nitrogen. Finally, to complete this pyridyl screen salt **10x** was prepared in 12% yield, providing a BCP analogue of pralidoxime.

**Scheme 2:**
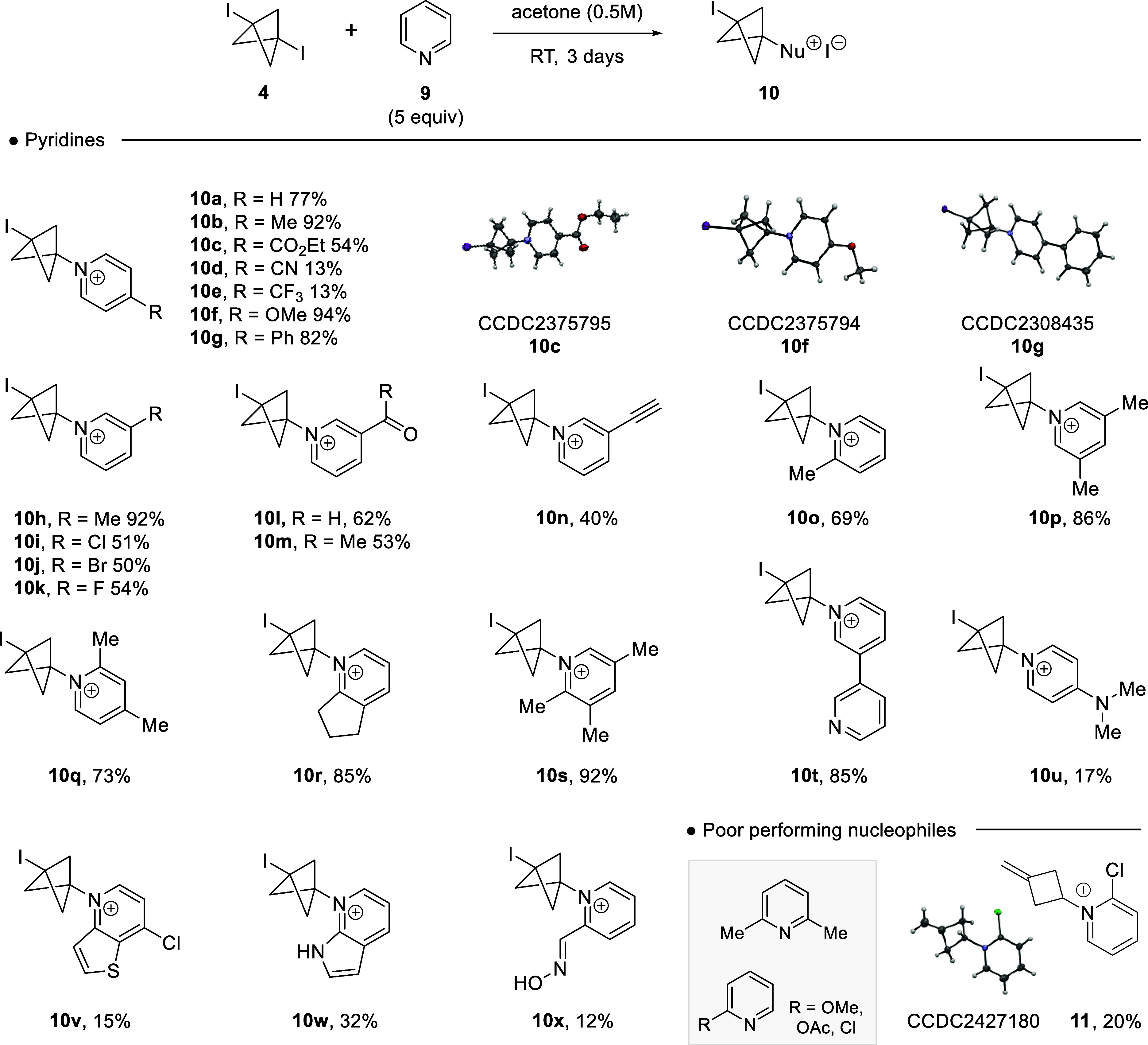
Pyridine Scope in the Addition to DIBCP **4** ^a^

a Anisotropic displacement ellipsoids are at the 50% probability level.

Poor performing nucleophiles included 2,6-lutidine which failed to add to **4**, with only starting material being observed after 3 days. Additionally, 2-methoxypyridine, 2-chloropyridine and 2-acetoxypyridine also failed to provide the anticipated pyridinium BCP salts. In the case of 2-chloropyridine, the reaction was attempted in acetone, but this provided a modest yield of the pyridinium cyclobutane salt **11**, as confirmed by single crystal X-ray analysis.^17^ We postulate this occurs from the rearrangement of DIBCP **4** to 1-iodo-3-methylenecyclobutane,^11a,13^ followed by nucleophilic displacement with 2-chloropyridine.

We next examined the reaction with quinoline and isoquinoline nucleophiles (Scheme 3). Direct *N*-alkylation of the quinoline with **4** had been elusive in prior reports,^11a,11b^ but if successful would provide direct route into bicyclo[1.1.1]­pentylquinolinium salts, potential precursors to quinolone antibiotic analogues (e.g., Figure 1). Reaction of quinoline with **4** using the modified reaction conditions gave the anticipated quinolinium salt **12a** in good yield of 71%. 4-Methoxy quinoline performed well, giving **12b** in 73%. 5-Bromo- and 7-chloro-4-methoxyquinoline proved challenging substrates, with **12c** and **12d** being isolated in yields of 9 and 15%, respectively. Additionally, purification of these salts was problematic given the large excess of nucleophile. Consequently, a limited optimization was performed for the reaction of quinoline with **4**, consequently we were able to improve the isolated yield of **12d** to 45% by using 2 equiv of the nucleophile.

**Scheme 3:**
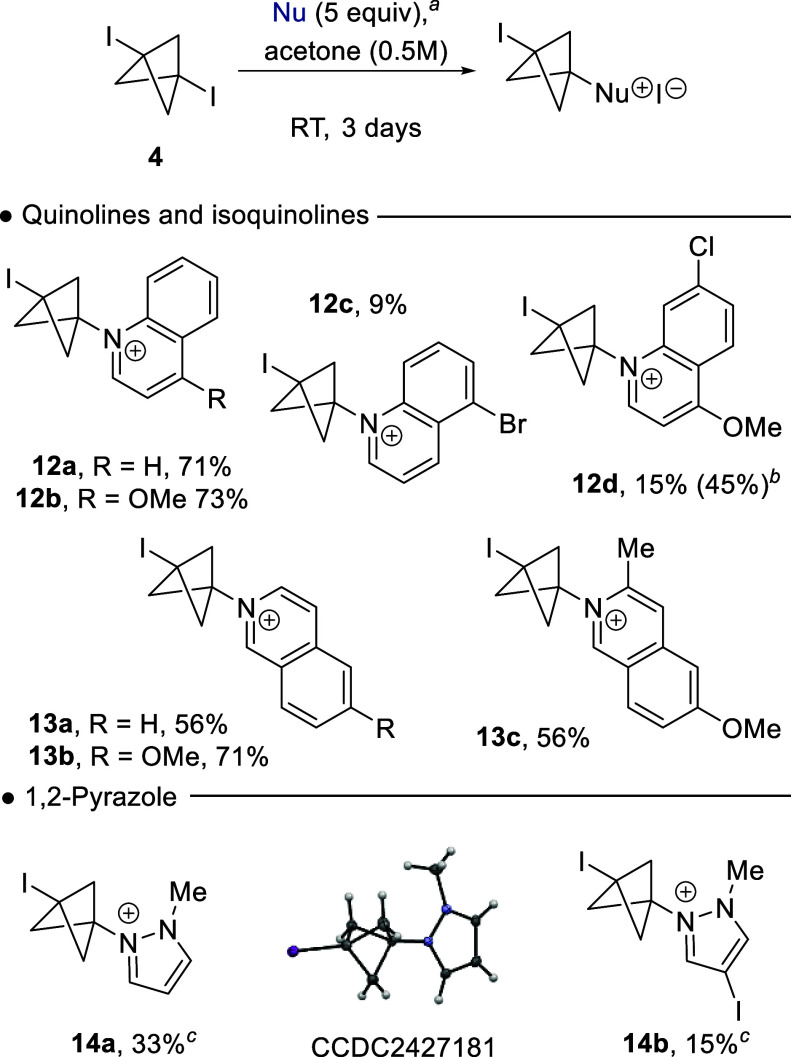
Addition of Quinolines, Isoquinolines, and Pyrazoles to **4** [Fn sch3-fn1]

a
^a^5 equiv of nucleophile unless otherwise stated; ^b^2 equiv of quinoline; ^c^10 equiv of pyrazole. Anisotropic displacement ellipsoids are at the 50% probability level.

Isoquinoline and 6-methoxyisoquinoline gave the BCP salts **13a** and **13b** in good, isolated yields of 56 and 71%, respectively; this yield profile was also observed for the BCP salt **13c** (Scheme 3). The synthesis of bicyclo[1.1.1]­pentylpyrazoles (BCPPs) pyrazole fragments has become an area of recent focus, given their structural relevance to the arylpyrazoles motif contained within several marketed drugs (e.g., celecoxib).^12^ Consequently, we examined the reaction of 1-methyl-1,2-pyrazole with **4** using the standard conditions, which failed to provide any product; however, a 2-fold increase in **4** did provide **14a** in 33% yield. Initially, identification of the product structure was complicated by which pyrazole nitrogen had reacted in the addition to **4**; however, this was subsequently confirmed by single crystal X-ray analysis.^17^ The reaction of 3-iodo-1-methyl-1,2-pyrazole with **4** was also performed, providing **14b** in a low 15% isolated yield.

Adcock and Gakh proposed a mechanism for the formation of the bicyclo[1.1.1]­pentylpyridinium salts, but it has remained underexplored since its original report in 1992.^11b^ Consequently, several questions remain unanswered, including the stability of the pyridinium salts, the reversibility of their formation, and regeneration of the [1.1.1]­propellane **2**. We therefore exposed **10a** to pyridine **9p** in both refluxing acetone and refluxing methanol. This gave only starting material with none of the pyridinium **10p** being observed by ^1^H NMR spectroscopy (Scheme 4a). This result indicates the formation of the pyridinium salt **10a** is irreversible under the reaction conditions. Next, we examined an energy profile for the formation of the bicyclo[1.1.1]­pentylpyridinium salt **10a** from **4** (Scheme 4b).

**Scheme 4:**
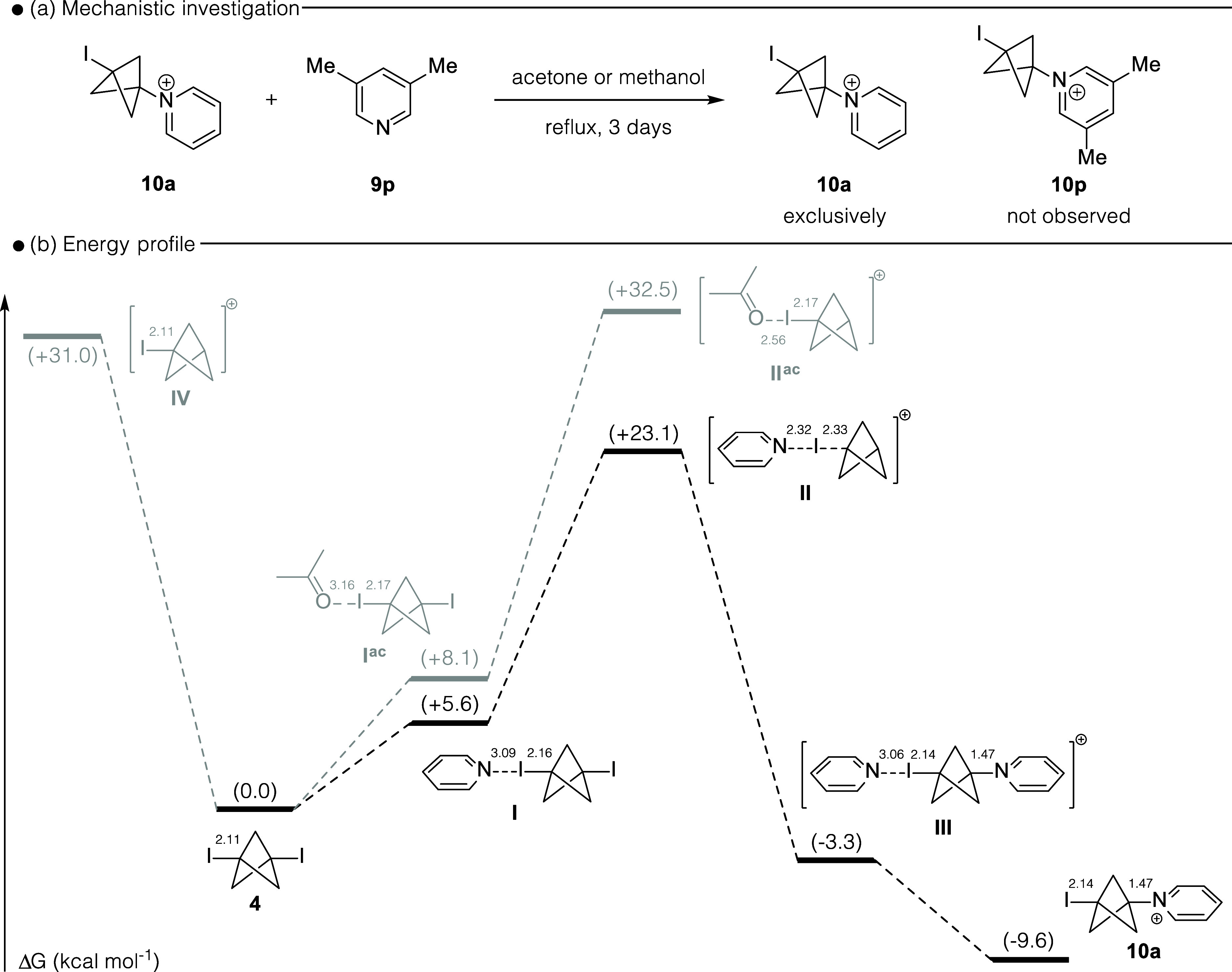
(a) Reversibility of Bicyclo[1.1.1]­pentylpyridinium Formation; (b) Calculated Reaction Profile To Produce Pyridinium BCP Salt^a^

a The iodide counterion has been omitted for clarity. Energy changes and bond lengths at the M06-2X/SDD (for iodide atom) and 6-311+G* (for the rest)/SMD (acetone) and shown in kcal mol^–1^ and Å, respectively.

The starting diiodide **4** can interact with the solvent, acetone, and pyridine through a halogen bond; with the acetone interaction (**I** ^**ac**^) being 2.5 kcal mol^–1^ higher in energy compared with pyridine (**I**; + 5.6 kcal mol^–1^). A similar reaction manifold had been observed by Aissa and co-workers, in their reaction of [1.1.1]­propellane with *N*-iodosuccinimide.^7c^ Adcock and Gakh^11a,11b^ determined that the reaction of **4** was first order with respect pyridine and based on this we next determined the energy of carbocation intermediate **II** (+23.1 kcal mol^–1^). We also calculated the energy of carbocation **II** ^**ac**^ (+32.5 kcal mol^–1^) which could potentially derive from the halogen bond complex **I** ^**ac**^. This intermediate **II** ^**ac**^ was found to be 9.4 kcal mol^–1^ higher in energy than the intermediate (**II**) previously proposed by Adcock and Gakh.^11a,11b^ Intermediate **II** can undergo pyridine addition forming intermediate **III**, which is 3.3 kcal mol^–1^ lower in energy than **4**. Finally, dissociation of the coordinated pyridine from **III** provides the observed product **10a**, with the formation of **10a** from **4** being exothermic by 9.6 kcal mol^–1^. We also examined the formation of carbocation **IV**, formed directly from **4** without prior halogen bond activation, but this appears an unfavorable pathway compared to the halogen bond activation and carbocation formation with pyridine. Our calculations demonstrated a significant energy barrier between **II** and **III** (Δ*G* = −26.4 kcal mol^–1^). This appears consistent with the experimental results in Scheme 4a where no pyridine exchange was observed.

Finally, our focus moved to explore the synthetic utility of the bicyclo[1.1.1]­pentylpyridinium and bicyclo[1.1.1]­pentylquinolinium salts (Scheme 5).

**Scheme 5:**
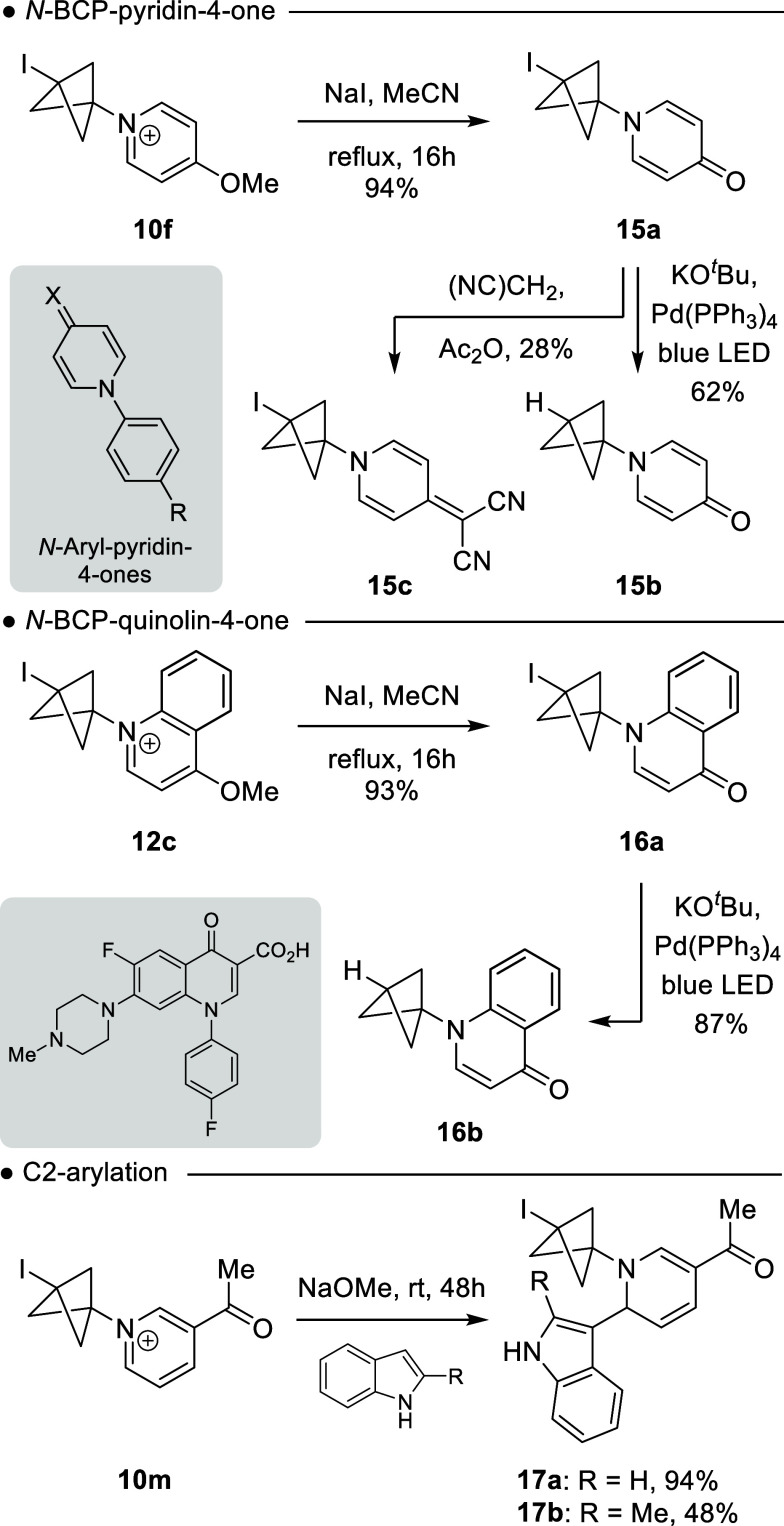
Synthetic Utility of Pyridinium and Quinolinium BCP Salts

*N*-Aryl-pyridin-4-ones have been used within the materials sector, as building blocks in organelle DNA marker synthesis, and as agrochemical fungicide and bactericides.^18^ Consequently, we found that treatment of 4-methoxy pyridinium **10f** with NaI cleanly produced the BCP-pyridin-4-one **15a** in an excellent 94% isolated yield, thereby providing an *N*-arylpyridin-4-one isostere. Furthermore, **15a** could be deiodinated using Pd-photocatalytic conditions,^19^ providing **15b** in 62% yield, as well as its reaction with malonitrile to provide **15c**, a BCP isostere of an *N*-aryl-pyridin-4-ones liquid crystal building block.^18a^ Similarly, treatment of **12c** with NaI provided bicyclo[1.1.1]­pentylquinolone **16a** in 93%; this substrate also underwent clean deiodination to give the BCP-quinolone **16b** in 87% yield. This later synthetic sequence provides anovel route to *N*-bicyclo­[1.1.1]­pentyl analogous *of* quinolin-4-ones, privileged medicinal chemistry scaffolds.^20^ Finally, we investigated the direct C2-arylation of **10m** with indole and 2-methylindole. We found addition was not impeded by the bicyclo[1.1.1]­pentyl unit with the addition providing **17a** and **17b** in 94 and 48% yield, respectively.

## Conclusions

In conclusion, we have shown the value of DIBCP as a feedstock in synthesizing bicyclo[1.1.1]­pentanes through a nucleophilic substitution reaction manifold. The reaction conditions are mild, with the desired bicyclo[1.1.1]­pentylpyridinium salts obtained through simple trituration and filtration, with recrystallization required in some instances. The pyridinium substrate scope is extensive, and in several cases, the unique structure of these bicyclo[1.1.1]­pentanes salts has been determined through single-crystal X-ray crystallography. The reaction scope has been expanded to include quinolines, isoquinolines and pyrazoles, thereby providing new routes into quinoline and pyrazole-substituted bicyclo[1.1.1]­pentanes which could have applications in synthesizing novel quinolone antibiotics and arylpyrazole bioisosteres. A mechanism, supported by detailed computational analysis, is proposed which involves the formation of a pyridine-iodine-BCP cation complex, that undergoes further addition by a second pyridine nucleophile, providing the observed pyridinium BCP. The synthetic potential of the synthesized pyridinium BCP isosteres has been explored, by providing an expedient synthetic route to pyridinone and quinolinone BCP analogues. Finally, we hope that this disclosure demonstrates the synthetic potential of DIBCP in accessing bicyclo[1.1.1]­pentanes of material and medicinal value.

## Experimental Section

### General Information

All reactants and reagents were purchased from commercial suppliers and used without further purification unless otherwise stated. Column chromatography was carried out using silica gel 60, 40–60 μm mesh (Apollo Scientific). Analytical thin-layer chromatography was performed on precoated aluminum silica gel 60 F254 plates (Merck), which were approximately 2.5 × 5 cm in size and visualized using ultraviolet light (254/365 nm) and a vanillin stain when necessary. NMR spectra were recorded on Jeol ECS 400 MHz and Jeol ECZ 500 MHz spectrometers. Chemical shifts are reported in ppm downfield of tetramethylsilane (TMS) using TMS or the residual solvent as an internal reference. NMR spectra were processed using MestReNova. Multiplicities are reported as singlet (s), doublet (d), triplet (t), and multiplet (m). HRMS were recorded using a Thermo Scientific Exactive Orbitrap mass spectrometer. IR spectra were collected on a Thermo Scientific Nicolet FTIR spectrometer. Melting points were determined in open-ended capillaries using a Stuart Scientific SMP10 melting point apparatus at a ramping rate of 1 °C/min.

### Preparation of 1,3-Diiodobicyclo[1.1.1]­pentane (4, DIBCP)

A 100 mL round-bottomed flask that has been flame-dried was charged with 1,1-dibromo-2,2-bis­(chloromethyl)­cyclopropane (2.97 g, 10.00 mmol, 1 equiv) and a stirrer bar. The flask was then purged with nitrogen. 45 mL of anhydrous diethyl ether was added to the flask and the solid was dissolved as the reaction was stirred. The flask was cooled to −78 °C with a dry ice-acetone bath before a methyllithium solution (1.6 M in diethyl ether, 13.75 mL, 22.00 mmol, 2.2 equiv) transferred via a syringe over 10 min dropwise. The reaction mixture was maintained at −78 °C for another 20 min; then the reaction was warmed to 0 °C by replacing the cooling bath with an ice–water bath. Stirring was continued for an additional two hours; 7 mL of methanol was then added to quench any excess methyllithium. The reaction was stirred for another 10 min, and then iodine (2.54 g, 10.00 mmol, 1 equiv) was added with the solution going clear to dark brown. The reaction was stirred for an hour in an ice–water bath after which the cooling bath was removed. The reaction was left to stir overnight, during this time the color gradually changed to a pale yellow. The mixture was diluted with ethyl acetate (50 mL) and transferred to a separating funnel before being washed with a saturated sodium thiosulfate solution (50 mL × 3). The organic phase was then washed with brine (50 mL × 2) and dried over magnesium sulfate. After filtration, the solvent was removed under reduced pressure yielding a white solid (2.72 g, 8.50 mmol, 85%). ^1^H NMR (500 MHz, CDCl_3_) δ 2.67 (s, 6H); ^13^C NMR (Chloroform-*d*, 101 MHz, CDCl_3_) δ 68.2, −1.6. Data in agreement with the literature.^9^

### Preparation of Bicyclo[1.1.1]­pentane Salts

#### General Procedure

To a 10 mL round-bottomed flask, a stirrer bar, DIBCP **4** (0.5 mmol, 1 equiv), acetone (1 mL) and nucleophile (2.5 mmol, 5 eq. unless otherwise stated) were added. This mixture was stirred at a low frequency for 3 days. On completion, the reaction had 7.5 mL of diethyl ether added. The contents of the flask were then filtered under vacuum. A spatula was utilized for the removal of any solid remaining on the glassware. At this point the solid was then washed with a further 15 mL diethyl ether, before being placed in a glass sample vial and dried on a vacuum line. Vapor diffusion recrystallization (methanol/diethyl ether) was then employed for certain compounds.

#### 1-(3-Iodobicyclo­[1.1.1]­pentanyl)­pyridinium Iodide (**10a**)^11b^

Brown solid (153 mg, 77%); mp 225–227 °C (decomp.); ^1^H NMR (500 MHz, DMSO-*d*_6_) δ 9.09 (d, *J* = 5.8 Hz, 2H), 8.68 (t, *J* = 7.7 Hz, 1H), 8.21 (t, *J* = 7.0 Hz, 2H), 2.97 (s, 6H); ^13^C NMR (126 MHz, DMSO-*d*_6_) δ 146.6, 142.5, 127.8, 61.2, 60.7, −4.8; IR (cm^–1^): 3112, 3070, 3039, 2993, 2919, 2878, 1624, 1572, 1472, 1359, 1274, 1237, 1201, 1170, 1100, 1082, 1020, 938; HRMS (ESI) *m*/*z*: [M]^+^ Calcd for C_10_H_11_IN 271.9931: Found 271.9930.

#### 1-(3-Iodobicyclo­[1.1.1]­pentanyl)-4-methylpyridinium Iodide (**10b**)

Light brown solid (190 mg, 92%); mp 246–248 °C (decomp.); ^1^H NMR (500 MHz, DMSO-*d*_6_) δ 8.92 (d, *J* = 6.6 Hz, 2H), 8.05 (d, *J* = 6.5 Hz, 2H), 2.92 (s, 6H), 2.63 (s, 3H); ^13^C NMR (126 MHz, DMSO-*d*_6_) δ 160.3, 141.4, 128.1, 60.7, 60.6, 21.6, −4.7; IR (cm^–1^): 3030, 2883, 1636, 1282, 1241, 1201, 1080, 1037, 899, 818, 765, 703, 608, 557, 529, 505, 452; HRMS (ESI) *m*/*z*: [M]^+^ Calcd for C_11_H_13_IN 286.0087: Found 286.0086.

#### 1-(3-Iodobicyclo­[1.1.1]­pentanyl)-4-ethoxycarbonylpyridinium Iodide (**10c**)

Yellow solid (128 mg, 54%); mp 193–195 °C (decomp.); ^1^H NMR (400 MHz, DMSO-*d*_6_) δ 9.22 (d, *J* = 6.9 Hz, 2H), 8.51 (d, *J* = 6.9 Hz, 2H), 4.45 (q, *J* = 7.1 Hz, 2H), 2.98 (s, 6H), 1.37 (t, *J* = 7.1 Hz, 3H); ^13^C NMR (126 MHz, DMSO-*d*_6_) δ 161.9, 144.6, 144.0, 126.8, 63.1, 61.5, 60.9, 13.9, −5.1; IR (cm^–1^): 3100, 3019, 2983, 2923, 1724, 1638, 1572, 1273, 1240, 1200, 1174, 1114, 1081, 1041, 1009; HRMS (ESI) *m*/*z*: [M]^+^ Calcd for C_13_H_15_INO_2_ 344.0142: Found 344.0141. Crystals suitable for X-ray diffraction were obtained by vapor diffusion (methanol/diethyl ether).

#### 1-(3-Iodobicyclo­[1.1.1]­pentanyl)-4-cyanopyridinium Iodide (**10d**)

Yellow solid (28 mg, 13%); mp 220–222 °C (decomp.); ^1^H NMR (500 MHz, DMSO-*d*_6_) δ 9.34 (d, *J* = 7.0 Hz, 2H), 8.79 (d, *J* = 6.8 Hz, 2H), 2.95 (s, 6H). ^13^C NMR (126 MHz, DMSO-*d*_6_) δ 144.1, 130.8, 127.7, 114.8, 61.8, 61.0, −5.4; IR (cm^–1^): 3093, 3003, 2922, 2827, 2244, 1634, 1228, 1198, 1120, 1075, 1050, 972; HRMS (ESI) *m*/*z*: [M]^+^ Calcd for C_11_H_10_IN_2_ 296.9883: Found 296.9883.

#### 1-(3-Iodobicyclo­[1.1.1]­pentanyl)-4-trifluoromethylpyridinium Iodide (**10e**)

Yellow solid (30 mg, 13%); mp 214–216 °C (decomp.); ^1^H NMR (500 MHz, DMSO-*d*_6_) δ 9.39 (d, *J* = 6.7 Hz, 2H), 8.72 (d, *J* = 6.7 Hz, 2H), 2.99 (s, 6H); ^13^C NMR (126 MHz, DMSO-*d*_6_) δ 145.1, 143.4 (q, *J* = 35.8 Hz), 124.7 (d, *J* = 4.3 Hz), 121.3 (q, *J* = 275.2 Hz), 61.7, 61.0, −5.3; ^19^F NMR (471 MHz, DMSO-*d*_6_) δ −63.6 (s, 3F); IR (cm^–1^): 3042, 2918, 1641, 1508, 1245, 1216, 1199, 1178, 1147, 1117, 1092, 1070, 1049; HRMS (ESI) *m*/*z*: [M]^+^ Calcd for C_11_H_10_F_3_IN 339.9805: Found 339.9804.

#### 1-(3-Iodobicyclo­[1.1.1]­pentanyl)-4-methoxypyridinium Iodide (**10f**)

Light brown solid (202 mg, 94%); mp 193–195 °C (decomp.); ^1^H NMR (500 MHz, DMSO-*d*_6_) δ 8.82–8.80 (m, 2H), 7.67–7.64 (m, 2H), 4.12 (s, 3H), 2.89 (s, 6H); ^13^C NMR (101 MHz, DMSO-*d*_6_) δ 171.4, 143.7, 113.3, 60.7, 60.0, 58.5, −4.6; IR (cm^–1^): 2986, 2961, 2915, 1634, 1574, 1519, 1270, 1251, 1196, 1118, 1099, 1087, 1033, 1012; HRMS (ESI) *m*/*z*: [M]^+^ Calcd for C_11_H_13_INO 302.0036: Found 302.0035. Crystals suitable for X-ray diffraction were obtained by vapor diffusion (methanol/diethyl ether).

#### 1-(3-Iodobicyclo­[1.1.1]­pentanyl)-4-phenylpyridinium Iodide (**10g**)

Dark yellow solid (196 mg, 82%); mp 230–232 °C (decomp.); ^1^H NMR (500 MHz, DMSO-*d*_6_) δ 9.06 (d, *J* = 7.0 Hz, 2H), 8.56 (d, *J* = 7.0 Hz, 2H), 8.12 (dd, *J* = 8.1, 1.5 Hz, 2H), 7.71–7.64 (m, 3H), 2.99 (s, 6H); ^13^C NMR (126 MHz, DMSO-*d*_6_) δ 155.7, 142.5, 133.4, 132.4, 129.7, 128.4, 124.2, 60.8, 60.7, −4.6; IR (cm^–1^): 3018, 2917, 2881, 1627, 1596, 1544, 1506, 1322, 1294, 1249, 1204, 1127, 1087, 1016, 964; HRMS (ESI) *m*/*z*: [M]^+^ Calcd for C_16_H_15_IN 348.0244: Found 348.0243. Crystals suitable for X-ray diffraction were obtained by vapor diffusion (methanol/diethyl ether).

#### 1-(3-Iodobicyclo­[1.1.1]­pentanyl)-3-methylpyridinium Iodide (**10h**)

Brown solid (190 mg, 92%); mp 219–221 °C (decomp.); ^1^H NMR (500 MHz, DMSO-*d*_6_) δ 9.02 (s, 1H), 8.90 (d, *J* = 6.1 Hz, 1H), 8.52 (d, *J* = 8.0 Hz, 1H), 8.10 (dd, *J* = 8.0, 6.1 Hz, 1H), 2.96 (s, 6H), 2.53 (s, 3H); ^13^C NMR (126 MHz, DMSO-*d*_6_) δ 146.9, 141.8, 139.6, 138.8, 127.1, 61.0, 60.8, 17.8, −4.8; IR (cm^–1^): 3071, 3039, 2995, 2919, 2878, 1625, 1584, 1265, 1240, 1205, 1187, 1124, 1105, 1085, 1020; HRMS (ESI) *m*/*z*: [M]^+^ Calcd for C_11_H_13_IN 286.0087: Found 286.0086.

#### 1-(3-Iodobicyclo­[1.1.1]­pentanyl)-3-chloropyridinium Iodide (10i)

Off-white solid (110 mg, 51%); mp 191–193 °C (decomp.); ^1^H NMR (500 MHz, DMSO-*d*_6_) δ 9.38 (t, *J* = 1.5 Hz, 1H), 9.06 (dt, *J* = 6.1, 1.1 Hz, 1H), 8.83 (ddd, *J* = 8.5, 2.0, 1.0 Hz, 1H), 8.23 (dd, *J* = 8.4, 6.1 Hz, 1H), 2.96 (s, 6H); ^13^C NMR (126 MHz, DMSO-*d*_6_) δ 146.1, 142.0, 141.5, 134.1, 128.4, 61.3, 60.9, −5.2; IR (cm^–1^): 3069, 3029, 2998, 2923, 2883, 2798, 1622, 1482, 1443, 1274, 1246, 1229, 1205, 1192, 1129, 1112, 1103, 980; HRMS (ESI) *m*/*z*: [M]^+^ Calcd for C_10_H_10_^35^ClIN 305.9541: Found 305.9542.

#### 1-(3-Iodobicyclo­[1.1.1]­pentanyl)-3-bromopyridinium Iodide (**10j**)

Dark yellow solid (117 mg, 50%); mp 171–173 °C (decomp.); ^1^H NMR (500 MHz, DMSO-*d*_6_) δ 9.38 (m, 1H), 9.07 (d, *J* = 6.1 Hz, 1H), 8.92 (ddd, *J* = 8.5, 1.8, 1.0 Hz, 1H), 8.13 (dd, *J* = 8.3, 6.1 Hz, 1H), 2.95 (s, 6H); ^13^C NMR (126 MHz, DMSO-*d*_6_) δ 148.8, 143.7, 141.7, 128.5, 122.2, 61.2, 61.0, −5.1; IR (cm^–1^): 3112, 3070, 3039, 2993, 2919, 2878, 1625, 1359, 1274, 1238, 1202, 1171, 1100, 1083, 1050, 1020; HRMS (ESI) *m*/*z*: [M]^+^ Calcd for C_10_H_10_^79^BrIN 349.9036: Found 349.9035.

#### 1-(3-Iodobicyclo­[1.1.1]­pentanyl)-3-fluoropyridinium Iodide (**10k**)

Light brown solid (112 mg, 54%) mp 198–200 °C (decomp.); ^1^H NMR (500 MHz, DMSO-*d*_6_) δ 9.48–9.46 (m, 1H), 9.00 (dt, *J* = 6.0, 1.1 Hz, 1H), 8.75–8.71 (m, 1H), 8.30 (*J* = 8.9, 5.8 Hz, 1H), 2.96 (s, 6H); ^13^C NMR (126 MHz, DMSO-*d*_6_) δ 159.8 (d, *J* = 253.0 Hz), 139.8 (d, *J* = 4.9 Hz), 134.0 (d, *J* = 18.6 Hz), 133.2 (d, *J* = 38.1 Hz), 129.2 (d, *J* = 8.3 Hz), 61.4, 60.9, −5.3; ^19^F NMR (471 MHz, DMSO-*d*_6_) δ −116.9 (s, 1F); IR (cm^–1^): 3059, 3032, 3000, 2894, 1631, 1585, 1273, 1208, 1177, 1131, 1103, 1077, 1029, 950; HRMS (ESI) *m*/*z*: [M]^+^ Calcd for C_10_H_10_FIN 289.9836: Found 289.9835.

#### 1-(3-Iodobicyclo­[1.1.1]­pentanyl)-3-formylpyridinium Iodide (**10l**)

Orange solid (132 mg, 62%); mp 207–209 °C (decomp.); ^1^H NMR (500 MHz, DMSO-*d*_6_) δ 10.20 (s, 1H), 9.54 (s, 1H), 9.25 (td, *J* = 3.7, 2.3 Hz, 1H), 9.02 (dt, *J* = 8.0, 1.4 Hz, 1H), 8.37 (dd, *J* = 7.7, 6.0 Hz, 1H), 3.00 (s, 6H); ^13^C NMR (126 MHz, DMSO-*d*_6_) δ 188.7, 146.1, 145.2, 144.4, 134.2, 128.3, 61.5, 60.9, −5.2; IR (cm^–1^): 3002, 2936, 1712, 1627, 1585, 1286, 1258, 1200, 1113, 1089, 1074, 1031, 939; HRMS (ESI) *m*/*z*: [M]^+^ Calcd for C_11_H_11_INO 299.9880: Found 299.9882.

#### 1-(3-Iodobicyclo­[1.1.1]­pentanyl)-3-acetylpyridinium Iodide (**10m**)

Dark yellow solid (118 mg, 53%); mp 202–204 °C (decomp.); ^1^H NMR (500 MHz, DMSO-*d*_6_) δ 9.25–9.24 (m, 1H), 9.20 (dt, *J* = 6.1, 1.3 Hz, 1H), 9.06–9.04 (m, 1H), 8.34 (ddd, *J* = 8.2, 6.1, 0.5 Hz, 1H), 3.01 (s, 6H), 2.77 (s, 3H); ^13^C NMR (126 MHz, DMSO-*d*_6_) δ 194.2, 145.3, 145.1, 142.5, 135.3, 128.0, 61.5, 60.9, 27.6, −5.0; IR (cm^–1^): 3056, 3000, 1695, 1620, 1322, 1281, 1272, 1210, 1082, 1027, 1018, 1003; HRMS (ESI) *m*/*z*: [M]^+^ Calcd for C_12_H_13_INO 314.0036: Found 314.0035.

#### 1-(3-Iodobicyclo­[1.1.1]­pentanyl)-3-ethynylpyridinium Iodide (**10n**)

Light brown solid (85 mg, 40%); mp 181–183 °C (decomp.); ^1^H NMR (400 MHz, DMSO-*d*_6_) δ 9.28 (s, 1H), 9.03 (dt, *J* = 6.2, 1.2 Hz, 1H), 8.75 (dt, *J* = 8.0, 1.3 Hz, 1H), 8.20 (ddd, *J* = 8.1, 6.2, 0.5 Hz, 1H), 5.04 (s, 1H), 2.94 (s, 6H); ^13^C NMR (101 MHz, DMSO-*d*_6_) δ 148.4, 145.1, 142.3, 127.8, 122.3, 89.1, 76.7, 61.3, 60.9, −5.1; IR (cm^–1^): 3158, 3013, 2932, 2109, 1618, 1570, 1248, 1210, 1130, 1107, 1086, 1027; HRMS (ESI) *m*/*z*: [M]^+^ Calcd for C_12_H_11_IN 295.9931: Found 295.9930.

#### 1-(3-Iodobicyclo­[1.1.1]­pentanyl)-2-methylpyridinium Iodide (**10o**)

Light brown solid (143 mg, 69%); mp 191–193 °C (decomp.); ^1^H NMR (500 MHz, DMSO-*d*_6_) δ 8.81 (dd, *J* = 6.3, 1.0 Hz, 1H), 8.54 (td, *J* = 7.8, 1.3 Hz, 1H), 8.04 (d, *J* = 7.7 Hz, 1H), 7.98 (t, *J* = 7.0 Hz, 1H), 3.07 (s, 6H), 2.89 (s, 3H); ^13^C NMR (101 MHz, DMSO-*d*_6_) δ 155.5, 146.3, 144.0, 130.2, 125.4, 62.1, 61.5, 21.1, −3.6; IR (cm^–1^): 3064, 3032, 2974, 2912, 1626, 1570, 1560, 1275, 1262, 1240, 1214, 1171, 1059, 1042, 1021; HRMS (ESI) *m*/*z*: [M]^+^ Calcd for C_11_H_13_IN 286.0087: Found 286.0087.

#### 1-(3-Iodobicyclo­[1.1.1]­pentanyl)-3,5-dimethylpyridinium Iodide (**10p**)

Brown solid (183 mg, 86%); mp 233–235 °C (decomp.); ^1^H NMR (400 MHz, DMSO-*d*_6_) δ 8.80 (s, 2H), 8.37 (s, 1H), 2.93 (s, 6H), 2.47 (s, 6H); ^13^C NMR (126 MHz, DMSO-*d*_6_) δ 147.3, 139.1, 137.9, 60.9, 60.8, 17.7, −4.7; IR (cm^–1^): 3009, 2918, 2883, 1624, 1598, 1507, 1473, 1382, 1307, 1285, 1224, 1203, 1145, 1108, 1049, 1021; HRMS (ESI) *m*/*z*: [M]^+^ Calcd for C_12_H_15_IN 300.0244: Found 300.0241.

#### 1-(3-Iodobicyclo­[1.1.1]­pentanyl)-2,4-dimethylpyridinium Iodide (**10q**)

Dark brown solid (157 mg, 73%); mp 183–185 °C (decomp.); ^1^H NMR (400 MHz, DMSO-*d*_6_) δ 8.64 (d, *J* = 6.6 Hz, 1H), 7.88 (m, 1H), 7.82 (dd, *J* = 6.6, 2.0 Hz, 1H), 3.03 (s, 6H), 2.81 (s, 3H), 2.56 (s, 3H); ^13^C NMR (126 MHz, DMSO-*d*_6_) δ 159.7, 154.0, 143.0, 130.3, 125.8, 61.6, 61.5, 21.2, 20.8, −3.5; IR (cm^–1^): 3009, 2908, 1636, 1557, 1500, 1466, 1434, 1257, 1216, 1204, 1141, 1123, 1070, 1021; HRMS (ESI) *m*/*z*: [M]^+^ Calcd for C_12_H_15_IN 300.0244: Found 300.0242.

#### 1-(3-Iodobicyclo­[1.1.1]­pentanyl)-6,7-dihydro-5*H*-cyclopenta­[*b*]­pyridinium Iodide (**10r**)

Black solid (187 mg, 85%); mp 224–226 °C (decomp.); ^1^H NMR (500 MHz, DMSO-*d*_6_) δ 8.57 (dd, *J* = 6.3, 1.0 Hz, 1H), 8.40 (dd, *J* = 7.7, 1.1 Hz, 1H), 7.86 (dd, *J* = 7.6, 6.4 Hz, 2H), 3.39 (t, *J* = 7.7 Hz, 2H), 3.05 (t, *J* = 7.7 Hz, 2H), 2.97 (s, 6H), 2.21–2.15 (m, 2H); ^13^C NMR (126 MHz, DMSO-*d*_6_) δ 160.4, 145.5, 141.7, 140.1, 125.3, 62.0, 61.1, 32.0, 29.9, 22.4, −3.9; IR (cm^–1^): 2989, 2949, 2898, 1607, 1582, 1290, 1269, 1236, 1206, 1112, 1079, 1057; HRMS (ESI) *m*/*z*: [M]^+^ Calcd for C_13_H_15_IN 312.0244: Found 312.0241.

#### 1-(3-Iodobicyclo­[1.1.1]­pentanyl)-2,3,5-trimethylpyridinium Iodide (**10s**)

Light brown solid (203 mg, 92%); mp 189–191 °C (decomp.); ^1^H NMR (400 MHz, DMSO-*d*_6_) δ 8.48 (s, 1H), 8.30 (s, 1H), 3.07 (s, 6H), 2.73 (s, 3H), 2.43 (s, 3H), 2.43 (s, 6H); ^13^C NMR (126 MHz, DMSO-*d*_6_) δ 151.9, 147.3, 140.7, 137.8, 134.7, 62.6, 61.8, 19.1, 17.3, 17.2, −3.5; IR (cm^–1^): 3010, 2920, 1621, 1512, 1492, 1466, 1444, 1392, 1264, 1244, 1212, 1174, 1135, 1039; HRMS (ESI) *m*/*z*: [M]^+^ Calcd for C_13_H_17_IN 314.0400: Found 314.0400.

#### 1-(3-Iodobicyclo­[1.1.1]­pentanyl)-[3,3′-bipyridin]-ium Iodide (**10t**)

Brown solid (202 mg, 85%); mp 224–226 °C (decomp.); ^1^H NMR (400 MHz, DMSO-*d*_6_) δ 9.33–9.32 (m, 1H), 9.13 (dd, *J* = 2.4, 0.8 Hz, 1H), 9.07–9.02 (m, 2H), 8.78 (dd, *J* = 4.8, 1.6 Hz, 1H), 7.67 (ddd, *J* = 8.0, 4.8, 0.9 Hz, 1H), 3.02 (s, 6H); ^13^C NMR (101 MHz, DMSO-*d*_6_) δ 150.9, 148.5, 144.3, 141.3, 140.6, 137.0, 135.6, 129.1, 127.9, 124.1, 61.4, 61.0, −5.0; IR (cm^–1^): 2999, 1621, 1584, 1506, 1339, 1308, 1272, 1222, 1206, 1127, 1091, 1013; HRMS (ESI) *m*/*z*: [M]^+^ Calcd for C_15_H_14_IN_2_ 349.0196: Found 349.0196.

#### 1-(3-Iodobicyclo­[1.1.1]­pentanyl)-*N*,*N*-dimethylpyridin-4-aminium Iodide (**10u**)

Dark brown solid (38 mg, 17%); mp 212–214 °C (decomp.); ^1^H NMR (500 MHz, DMSO-*d*_6_) δ 8.23–8.20 (m, 2H), 7.05–7.02 (m, 2H), 3.22 (s, 6H), 2.81 (s, 6H); ^13^C NMR (126 MHz, DMSO-*d*_6_) δ 156.1, 139.1, 107.5, 60.4, 58.9, 40.1, −3.6; IR (cm^–1^): 3378, 2912, 1636, 1560, 1435, 1396, 1342, 1276, 1206, 1084; HRMS (ESI) *m*/*z*: [M]^+^ Calcd for C_12_H_16_IN_2_ 315.0353: Found 315.0352.

#### 7-Chloro-4-(3-iodobicyclo­[1.1.1]­pentanyl)­thieno­[3,2-*b*]­pyridinium Iodide (**10v**)

Dark yellow solid (37 mg, 15%); mp 197–199 °C (decomp.); ^1^H NMR (500 MHz, DMSO-*d*_6_) δ 8.98 (d, *J* = 6.6 Hz, 1H), 8.93 (d, *J* = 5.7 Hz, 1H), 8.42 (d, *J* = 5.7 Hz, 1H), 8.32 (d, *J* = 6.6 Hz, 1H), 3.14 (s, 6H); ^13^C NMR (126 MHz, DMSO-*d*_6_) δ 147.3, 145.2, 143.5, 143.4, 138.2, 120.2, 119.4, 61.4, 61.0, −4.2; IR (cm^–1^): 3071, 3053, 2922, 1583, 1545, 1303, 1231, 1206, 1140, 1132, 1080, 1051; HRMS (ESI) *m*/*z*: [M]^+^ Calcd for C_12_H_10_^35^ClINS 361.9262: Found 361.9258.

#### 7-(3-Iodobicyclo­[1.1.1]­pentanyl)-1*H*-pyrrolo­[2,3-*b*]­pyridinium Iodide (**10w**)

Light brown solid (70 mg, 32%; mp 204–206 °C (decomp.); ^1^H NMR (500 MHz, DMSO-*d*_6_) δ 12.75 (s, 1H), 8.81 (d, *J* = 7.8 Hz), 8.47 (d, *J* = 6.2 Hz), 7.95 (d, *J* = 3.5 Hz), 7.66 (t, *J* = 7.0 Hz), 7.04 (d, *J* = 3.5 Hz), 3.15 (s); ^13^C NMR (126 MHz, DMSO-*d*_6_) δ 138.6, 137.0, 135.1, 130.6, 127.2, 116.1, 103.9, 60.0, 58.8, −3.9; IR (cm^–1^): 3368, 3099, 3078, 1606, 1247, 1210, 1173, 1144, 1112, 1057, 1040; HRMS (ESI) *m*/*z*: [M]^+^ Calcd for C_12_H_12_IN_2_ 311.0040: Found 311.0038.

#### (*E*)-2-(Hydroxyiminomethyl)-1-(3-iodobicyclo­[1.1.1]­pentanyl)­pyridinium Iodide (**10x**)

Light brown solid (12%; mp 175–177 °C); ^1^H NMR (400 MHz, DMSO-*d*_6_) δ 13.16 (s, 1H), 8.85 (dd, *J* = 6.3, 1.0 Hz, 1H), 8.77 (s, 1H), 8.60 (td, *J* = 7.8, 1.0 Hz, 1H), 8.32 (dd, *J* = 8.1, 1.4 Hz, 1H), 8.12 (ddd, *J* = 7.8, 6.4, 1.6 Hz, 1H), 3.04 (s, 6H); ^13^C NMR (126 MHz, DMSO-*d*_6_) δ 147.1, 146.6, 144.4, 141.4, 127.4, 126.6, 62.2, 62.0, −4.0; IR (cm^–1^): 3132, 3059, 2991, 2843, 2708, 1622, 1601, 1568, 1496, 1445, 1114, 1108, 1082, 1058; HRMS (ESI) *m*/*z*: [M]^+^ Calcd for C_11_H_12_IN_2_O 314.9989: Found 311.0038.

#### 2-Chloro-1-(3-methylenecyclobutyl)­pyridinium Iodide (**11**)

Dark red solid (20%; mp 92–94 °C (decomp.); ^1^H NMR (400 MHz, DMSO-*d*_6_) δ 9.20 (dd, *J* = 6.4, 1.4 Hz, 1H), 8.69–8.53 (m, 1H), 8.38 (dd, *J* = 8.2, 1.4 Hz, 1H), 8.12 (ddd, *J* = 7.7, 6.4, 1.4 Hz, 1H), 5.46 (p, *J* = 7.9 Hz, 1H), 5.07–5.04 (m, 2H), 3.54–3.32 (m, 4H); ^13^C NMR (101 MHz, DMSO-*d*_6_) δ 147.1, 146.1, 145.2, 137.5, 130.0, 126.1, 108.1, 59.1; IR (cm^–1^): 3116, 1687, 1626, 1608, 1564, 1285, 1237, 1191, 1159, 1145, 1097, 1062; HRMS (ESI) *m*/*z*: [M]^+^ Calcd for C_10_H_11_Cl^35^N 180.0575: Found 180.0575.

#### 1-(3-Iodobicyclo­[1.1.1]­pentanyl)­quinolinium Iodide (**12a**)

Dark yellow solid (159 mg, 71%); mp 204–206 °C (decomp.); ^1^H NMR (500 MHz, DMSO-*d*_6_) δ 9.36 (d, *J* = 8.3 Hz, 1H), 9.27 (dd, *J* = 5.9, 1.3 Hz, 1H), 8.83 (d, *J* = 9.0 Hz, 1H), 8.53 (dd, *J* = 8.2, 1.4 Hz, 1H), 8.25 (ddd, *J* = 8.8, 7.0, 1.5 Hz, 1H), 8.18 (dd, *J* = 8.3, 5.9 Hz, 1H), 8.08 (t, *J* = 7.8 Hz, 1H), 3.26 (s, 6H); ^13^C NMR (126 MHz, DMSO-*d*_6_) δ 149.1, 148.9, 137.1, 136.0, 131.1, 129.9, 129.5, 121.9, 119.5, 62.4, 61.6, −3.4; IR (cm^–1^): 3072, 3041, 2994, 2971, 1619, 1595, 1575, 1519, 1486, 1474, 1439, 1400, 1370, 1086, 1022, 1005; HRMS (ESI) *m*/*z*: [M]^+^ Calcd for C_14_H_13_IN 322.0087: Found 322.0087.

#### 1-(3-Iodobicyclo­[1.1.1]­pentanyl)-4-methoxyquinolinium Iodide (**12b**)

Light yellow solid (174 mg, 73%); mp 143–145 °C; ^1^H NMR (400 MHz, DMSO-*d*_6_) δ 9.02 (d, *J* = 7.2 Hz, 1H), 8.67 (d, *J* = 8.9 Hz, 1H), 8.48 (dd, *J* = 8.4, 1.4 Hz, 1H), 8.19 (ddd, *J* = 8.8, 7.1, 1.6 Hz, 1H), 8.00–7.96 (m, 1H), 7.57 (d, *J* = 7.3 Hz, 1H), 4.37 (s, 3H), 3.20 (s, 6H); ^13^C NMR (126 MHz, DMSO-*d*_6_) δ 169.6, 150.1, 137.9, 135.6, 129.0, 124.2, 120.8, 119.4, 102.3, 61.4, 61.3, 59.4, −3.0; IR (cm^–1^): 3106, 2994, 2882, 1619, 1597, 1584, 1573, 1530, 1325, 1227, 1212, 1169, 1100, 1032, 1004; HRMS (ESI) *m*/*z*: [M]^+^ Calcd for C_15_H_15_INO 352.0193: Found 352.0193.

#### 1-(3-Iodobicyclo­[1.1.1]­pentanyl)-5-bromoquinolinium Iodide (**12c**)

Light yellow solid (23 mg, 9%); mp 184–186 °C; ^1^H NMR (400 MHz, DMSO-*d*_6_) δ 9.42 (d, *J* = 8.5 Hz, 1H), 9.35 (dd, *J* = 1.4, 6.0 Hz, 1H), 8.86 (d, *J* = 9.1 Hz, 1H), 8.44 (d, *J* = 7.4 Hz, 1H); 8.27–8.25 (m, 1H), 8.11 (dd, *J* = 7.7, 9.1 Hz, 1H), 3.25 (s, 6H); ^13^C NMR (101 MHz, DMSO-*d*_6_) δ 169.4, 151.3, 141.1, 138.9, 130.1, 126.7, 119.9, 118.3, 103.2, 61.7, 59.9, −3.0; IR (cm^–1^): 3059, 1519, 1316, 1148; HRMS (ESI) *m*/*z*: [M]^+^ Calcd for C_14_H_12_IBrN 399.9192: Found 399.9185.

#### 1-(3-Iodobicyclo­[1.1.1]­pentanyl)-7-chloro-4-methoxyquinolinium Iodide (**12d**)

Light yellow solid (194 mg, 45%); mp 139–142 °C; ^1^H NMR (400 MHz, DMSO-*d*_6_) δ 9.04 (d, *J* = 6.9 Hz, 1H), 8.50 (t, *J* = 9.2 Hz, 2H), 8.02 (d, J = 9.2 Hz, 1H), 7.58 (d, *J* = 7.3 Hz, 1H), 4.37 (s, 3H), 3.2 (s, 6H); ^13^C NMR (101 MHz, DMSO-*d*_6_) δ 150.6, 147.9, 138.9, 136.6, 134.5, 128.9, 124.4, 123.7, 120.1, 62.2, −3.2; IR (cm^–1^): 2968, 1603, 1453, 1321; HRMS (ESI) *m*/*z*: [M]^+^ Calcd for C_15_H_14_IClON 385.9803: Found 385.9801.

#### 2-(3-Iodobicyclo­[1.1.1]­pentanyl)­isoquinolinium Iodide (**13a**)

Brown solid (126 mg, 56%); mp 210–212 °C (decomp.); ^1^H NMR (500 MHz, DMSO-*d*_6_) δ 10.01 (s, 1H), 8.81 (dd, *J* = 6.8, 1.5 Hz, 1H), 8.65 (d, *J* = 6.9 Hz, 1H), 8.57 (dd, *J* = 8.3, 0.7 Hz, 1H), 8.37 (d, *J* = 8.2 Hz, 1H), 8.30 (ddd, *J* = 8.3, 6.9, 1.2 Hz, 1H), 8.11 (ddd, *J* = 8.2, 6.9, 1.2 Hz, 1H), 3.02 (s, 1H); ^13^C NMR (126 MHz, DMSO-*d*_6_) δ 148.1, 137.5, 137.2, 132.0, 131.4, 130.7, 127.3, 126.8, 125.6, 61.3, 60.8, −4.4; IR (cm^–1^): 3114, 3043, 3004, 2921, 2880, 1637, 1627, 1604, 1350, 1277, 1251, 1106, 1086, 1073, 1051, 1021; HRMS (ESI) *m*/*z*: [M]^+^ Calcd for C_14_H_13_IN 322.0087: Found 322.0086.

#### 2-(3-Iodobicyclo­[1.1.1]­pentanyl)-6-methoxyisoquinolinium Iodide (**13b**)

Light brown solid (170 mg, 71%); mp 221–223 °C; ^1^H NMR (400 MHz, DMSO-*d*_6_) δ 9.76 (s, 1H), 8.66 (dd, *J* = 6.9, 1.5 Hz, 1H), 8.45 (d, *J* = 9.2 Hz, 1H), 8.39 (d, *J* = 7.0 Hz, 1H), 7.77 (d, *J* = 2.4 Hz, 1H), 7.70 (dd, *J* = 9.1, 2.5 Hz, 1H), 4.06 (s, 3H), 2.99 (s, 6H); ^13^C NMR (126 MHz, DMSO-*d*_6_) δ 166.0, 146.0, 140.2, 132.7, 132.2, 124.2, 123.7, 122.2, 106.0, 60.8, 56.8, −4.3 (one aromatic environment masked); HRMS (ESI) *m*/*z*: [M]^+^ Calcd for C_15_H_15_INO 352.0193: Found 352.0188.

#### 2-(3-Iodobicyclo­[1.1.1]­pentanyl)-6-methoxy-3-methylisoquinolinium Iodide (**13c**)

Light yellow solid (138 mg, 56%); mp 203–205 °C (decomp.); ^1^H NMR (400 MHz, DMSO-*d*_6_) δ 9.56 (s, 1H), 8.45 (d, *J* = 9.2 Hz, 1H), 8.14 (s, 1H), 7.61 (dd, *J* = 9.1, 2.4 Hz, 1H), 7.55 (d, *J* = 2.4 Hz, 1H), 4.05 (s, 3H), 3.11 (s, 6H), 2.86 (s, 3H); ^13^C NMR (101 MHz, DMSO-*d*_6_) δ 166.2, 147.8, 144.1, 141.1, 132.6, 124.1, 123.6, 121.4, 104.4, 62.1, 61.8, 56.7, 20.4, −3.5; HRMS (ESI) *m*/*z*: [M]^+^ Calcd for C_16_H_17_INO 366.0349: Found 366.0349.

#### 2-(3-Iodobicyclo­[1.1.1]­pentanyl)-1-methylpyrazolium Iodide (**14a**)

Alteration to the general procedure undertaken with DIBCP **4** (320 mg, 1.0 mmol) and 10 equiv of the nucleophile. Light orange solid (134 mg, 33%); mp 165–168 °C (decomp.); ^1^H NMR (400 MHz, DMSO-*d*_6_) δ 8.53 (d, *J* = 2.8 Hz, 1H), 8.50 (d, *J* = 3.0 Hz, 1H), 6.91 (t, *J* = 3.0 Hz, 1H), 4.17 (s, 3H), 2.98 (s, 6H); ^13^C NMR (101 MHz, DMSO-*d*_6_) δ 139.6, 136.8, 107.2, 61.4, 54.6, 38.5, −3.2; IR (cm^–1^): 3147, 2879, 1512, 1406, 1253; HRMS (ESI) *m*/*z*: [M]^+^ Calcd for C_9_H_12_IN_2_ 275.0040: Found 275.0040.

#### 4-Iodo-2-(3-Iodobicyclo­[1.1.1]­pentanyl)-1-methylpyrazolium Iodide (**14b**)

Alteration to the general procedure undertaken with DIBCP **4** (320 mg, 1.0 mmol) and 10 equiv of the nucleophile. Light cream solid (82 mg, 15%); mp 172–174 °C (decomp.); ^1^H NMR (400 MHz, DMSO-*d*_6_) δ 8.31 (s, 1H); 8.26 (s, 1H), 3.69 (s, 3H), 2.89 (s, 6H); ^13^C NMR (101 MHz, DMSO-*d*_6_) δ 143.4, 140.7, 61.5, 54.7, 54.6, 38.7, −3.5; IR (cm^–1^): 3071, 2967, 1705, 1522, 1368; HRMS (ESI) *m*/*z*: [M]^+^ Calcd for C_9_H_11_I_2_N_2_ 400.9006: Found 400.8991.

#### 1-(3-Iodobicyclo­[1.1.1]­pentanyl)­pyridin-4-one (**15a**)

To a round-bottomed flask, a stirrer bar, 1-(3-iodobicyclo[1.1.1]­pentanyl)-4-methoxypyridinium iodide **10f** (0.22 g, 0.50 mmol, 1 equiv), 30 mL of acetonitrile and sodium iodide (0.15 g, 1.00 mmol, 2 equiv) were added. The reaction mixture was refluxed for 24 h at 82 °C (heating block). On completion, the reaction mixture was allowed to cool before the solvent was removed under vacuum. The residue was taken up in 40 mL of chloroform; the resulting suspension was filtered through filter paper into a round-bottomed flask. The solvent was removed under reduced pressure to yield the title compound (0.27 g, 0.94 mmol, 94%); mp 205–207 °C (decomp.); ^1^H NMR (400 MHz, CDCl_3_) δ 7.25–7.22 (m, 2H), 6.38–6.34 (m, 2H), 2.66 (s, 6H); ^13^C NMR (101 MHz, CDCl_3_) δ 178.8, 136.3, 118.7, 60.8, 58.8, −4.8; IR (cm^–1^): 2916, 2879, 1634, 1571, 1507, 1490, 1457, 1271, 1203, 1173, 1072; HRMS (ESI) *m*/*z*: [M + H]^+^ Calcd for C_10_H_10_INO 287.9880: Found 287.9879.

#### 1-Bicyclo­[1.1.1]­pentanylpyridin-4-one (**15b**)

To a flame-dried round-bottomed flask, a stirrer bar, 1-(3-iodobicyclo[1.1.1]­pentanyl)­pyridin-4-one **15a** (0.11 g, 0.38 mmol, 1 equiv) and tetrakis­(triphenylphosphine)­palladium(0) (0.044 g, 0.038 mmol, 0.1 equiv) were added. The flask was then purged with argon. Eight mL of anhydrous isopropyl alcohol was added before potassium *tert*-butoxide (1 M in THF, 0.76 mL, 0.76 mmol, 2 equiv) was added to the stirred suspension. Argon was bubbled through the suspension for approximately 10 min before the flask was irradiated by a blue LED light (Kessil A160WE Tuna Blue, 40 W). After stirring for 22 h at room temperature the reaction was diluted with 20 mL of water and the resulting aqueous layer was extracted with dichloromethane (3 × 30 mL). The organic layers were combined and washed with brine (30 mL). This solution was dried with magnesium sulfate, filtered and concentrated under reduced pressure. The crude product was purified using flash column chromatography (petrol ether/chloroform 1:1 to chloroform/methanol 19:1) to give the title compound as a light brown solid (0.038 g, 0.236 mmol, 62%); mp 210–212 °C; ^1^H NMR (400 MHz, CDCl_3_) δ 7.35–7.32 (m, 2H), 6.38–6.34 (m, 2H), 2.68 (s, 1H), 2.22 (s, 6H); ^13^C NMR (101 MHz, CDCl_3_) δ 179.0, 136.3, 118.3, 56.1, 52.0, 21.2; IR (cm^–1^): 3204, 3012, 2919, 2882, 1681, 1634, 1569, 1541, 1272, 1218, 1192, 1070, 1020; HRMS (ESI) *m*/*z*: [M + H]^+^ Calcd for C_10_H_11_NO 162.0913: Found 162.0915.

#### 2-(1-(3-Iodobicycloc­[1.1.1]­pentanyl)­pyridine-4-(1H)-ylidene)­malononitrile (**15c**)

To a round-bottomed flask, a stirrer bar, 1-(3-iodobicyclo[1.1.1]­pentanyl)­pyridin-4-one **15a** (0.115 g, 0.40 mmol, 1 equiv) and malononitrile (0.026 g, 0.40 mmol, 1 equiv) were added. The flask was then purged with nitrogen and anhydrous acetonitrile (12 mL) then acetic anhydride (0.38 mL, 0.40 mmol, 1 equiv) were subsequently added. This reaction mixture was stirred for 16 h at 82 °C (heating block). On completion, the flask was allowed to cool slightly before the solvent was removed under reduced pressure. The crude product was purified using flash column chromatography (chloroform/methanol 19:1) to give the pure product as a yellow solid (0.038 g, 0.112 mmol, 28%); mp 215–217 °C (decomp.); ^1^H NMR (400 MHz, CDCl_3_) δ 7.90–7.87 (m, 2H), 6.82–6.79 (m, 2H), 2.76 (s, 6H); ^13^C NMR (101 MHz, CDCl_3_) δ 155.6, 137.2, 117.9, 112.4, 60.4, 59.0, 45.6, −3.5; IR (cm^–1^): 3059, 3013, 2920, 2197, 2166, 1644, 1506, 1310, 1284, 1197, 1177, 1081; HRMS (ESI) *m*/*z*: [M + Na]^+^ Calcd for C_13_H_10_IN_3_ 357.9812: Found 357.9811.

#### 1-(3-Iodobicyclo­[1.1.1]­pentanyl)­quinolin-4-one (**16a**)

To a round-bottomed flask, a stirrer bar, 1-(3-iodobicyclo[1.1.1]­pentanyl)-4-methoxyquinoline iodide **12c** (0.24 g, 0.50 mmol, 1 equiv), 70 mL of acetonitrile and sodium iodide (0.15 g, 1.00 mmol, 2 equiv) were added. The reaction mixture was refluxed for 24 h at 82 °C (heating block). On completion, the reaction mixture was allowed to cool before the solvent was removed under vacuum. The residue was taken up in 50 mL of chloroform; the resulting suspension was filtered through filter paper into a round-bottomed flask. The solvent was removed under reduced pressure to yield the title compound (157 mg, 0.93 mmol, 93%); mp 156–158 °C (decomp.); ^1^H NMR (400 MHz, CDCl_3_) δ 8.46–8.43 (m, 1H), 7.68–7.65 (m, 2H), 7.43–7.40 (m, 2H), 6.27 (d, *J* = 7.9 Hz, 1H), 2.94 (2, 6H); ^13^C NMR (101 MHz, CDCl_3_) δ 178.3, 140.3, 139.9, 132.1, 127.3, 127.0, 124.2, 116.6, 110.5, 62.0, 60.2, −2.8;; IR (cm^–1^): 2912, 1608, 1581, 1547, 1478, 1284, 1240, 1191, 1152, 1133, 1106, 1069, 1032, 1002; HRMS (ESI) *m*/*z*: [M + H]^+^ Calcd for C_14_H_12_INO 338.0036: Found 338.0036.

#### 1-Bicyclo­[1.1.1]­pentanylquinolin-4-one (**16b**)

To a flame-dried round-bottomed flask, a stirrer bar, 1-(3-iodobicyclo[1.1.1]­pentanyl)­quinolin-4-one **15a** (0.14 g, 0.40 mmol, 1 equiv) and tetrakis­(triphenylphosphine)­palladium(0) (0.045 g, 0.04 mmol, 0.1 equiv) were added. The flask was then purged with argon. Eight mL of anhydrous isopropyl alcohol was added before potassium *tert*-butoxide (1 M in THF, 0.80 mL, 0.80 mmol, 2 equiv) was added to the stirred suspension. Argon was bubbled through the suspension for approximately 10 min before the flask was irradiated by a blue LED light (Kessil A160WE Tuna Blue, 40 W). After stirring for 22 h at room temperature the reaction was diluted with 20 mL of water and the resulting aqueous layer was extracted with dichloromethane (3 × 30 mL). The organic layers were combined and washed with brine (30 mL). This solution was dried with magnesium sulfate, filtered and concentrated under reduced pressure. The crude product was purified using flash column chromatography (chloroform to chloroform/methanol 49:1) to give the title compound as a light brown solid (73 mg, 0.35 mmol, 87%); mp 95–97 °C; ^1^H NMR (400 MHz, CDCl_3_) δ 8.45 (ddd, *J* = 0.4, 1.7, 8.1 Hz, 1H), 7.83 (d, *J* = 8.6 Hz, 1H), 7.64 (ddd, *J* = 1.7, 7.0, 8.7 Hz, 1H); 7.55 (d, *J* = 7.9 Hz, 1H); 7.38 (ddd, *J* = 1.0, 7.0, 8.0 Hz, 1H), 6.26 (d, *J* = 7.9 Hz, 1H), 2.80 (s, 1H), 2.51 (s, 6H); ^13^C NMR (101 MHz, CDCl_3_) δ 178.4, 140.4, 131.7, 127.2, 127.0, 123.8, 117.0, 110.1, 57.6, 53.5, 23.5 (one carbon environment masked); IR (cm^–1^): 3056, 2995, 2922, 2873, 1626, 1605, 1582; HRMS (ESI) *m*/*z*: [M + H]^+^ Calcd for C_14_H_13_NO 212.1070: Found 212.1072.

#### 1-(1-(3-Iodobicyclo­[1.1.1]­pentanyl)-(6-(1H-indol-3-yl)-1,6-dihydropyridin-3-yl)­ethenone (**17a**)

To a flame-dried, argon flushed round-bottomed flask, a stirrer bar and indole (40 mg, 0.34 mmol, 1 equiv) were added. The flask was then purged with nitrogen. Anhydrous methanol (20 mL/mmol) was added to dissolve the solid before sodium methoxide (0.16 mL, 5.4 M in methanol, 2.5 eqv.) was added to the stirred solution; stirring was continued for a further 30 min. While this stirred a separate round-bottomed flask had a stirrer bar and 1-(3-iodobicyclo[1.1.1]­pentanyl)-3-acetylpyridinium iodide **10m** (180 mg, 0.41 mmol, 1.2 equiv) added. The flask was then purged with nitrogen before 30 mL of anhydrous methanol was added. The pyridinium solution was added to the stirred indole solution using a syringe pump (1.00 mL min^–1^). The reaction mixture was stirred at room temperature for forty-eight hours. On completion, the reaction mixture was concentrated under reduced pressure. The crude product was purified using flash column chromatography (chloroform/methanol 99:1) to give the title compound (154 mg, 94%); mp 96–98 °C (decomp.); ^1^H NMR (500 MHz, CDCl_3_) δ 8.12 (s, 1H), 7.60 (d, *J* = 8.1 Hz, 1H), 7.32 (dt, *J* = 8.2, 0.9 Hz, 1H), 7.15 (ddd, *J* = 8.2, 7.1, 1.2 Hz, 1H), 7.11 (d, *J* = 1.5 Hz, 1H), 7.06 (ddd, *J* = 8.0, 7.1, 1.0 Hz, 1H), 6.97 (d, *J* = 2.4 Hz, 1H), 5.96 (ddd, *J* = 7.8, 1.5, 0.8 Hz, 1H), 5.14 (dd, *J* = 7.8, 5.0 Hz, 1H), 4.89 (d, *J* = 5.0 Hz, 1H), 2.58 (s, 6H), 2.09 (s, 3H); ^13^C NMR (126 MHz, CDCl_3_) δ 196.1, 136.8, 136.5, 136.4, 126.1, 122.5, 122.3, 121.8, 119.3, 119.2, 113.3, 111.6, 110.3, 60.8, 58.7, 29.2, 25.2, −2.1; IR (cm^–1^): 3260, 2997, 2916, 1668, 1621, 1567, 1150, 1078, 1034; HRMS (ESI) *m*/*z*: [M-H]^−^ for C_20_H_18_IN_2_O 429.0469: Found 429.0459.

#### 1-(1-(3-Iodobicyclo­[1.1.1]­pentanyl)-(6-(2-methyl-1H-indol-3-yl)-1,6-dihydropyridin-3-yl)­ethenone (**17b**)

To a round-bottomed flask and a stirrer bar was added 2-methylindole (45 mg, 0.34 mmol, 1.0 equiv). The flask was then purged with nitrogen and anhydrous methanol (7.5 mL) was added to dissolve the solid before sodium methoxide (5.4 M in methanol, 0.16 mL, 0.848 mmol, 2.5 equiv) was added to the stirred solution; stirring was continued for a further 30 min. While this stirred a separate round-bottomed flask had a stirrer bar and 1-(3-iodobicyclo[1.1.1]­pentanyl)-3-acetylpyridinium iodide **10m** (0.18 g, 0.41 mmol, 1.2 equiv) added. The flask was then purged with nitrogen before anhydrous methanol (25 mL) was added. After the 30 min the indole solution was added to the pyridinium solution. The reaction mixture was stirred at room temperature for 24 h. On completion water (30 mL) was added to reaction mixture before it was transferred to a separatory funnel. The mixture was extracted with ethyl acetate (3 × 15 mL). The organic layers were combined and washed with brine (30 mL). This solution was dried with magnesium sulfate, filtered and concentrated under reduced pressure. The crude product was purified using flash column chromatography (chloroform/methanol 99:1) to give the title compound as a light brown solid (72 mg, 48%); mp 110–112 °C (decomp.); ^1^H NMR (500 MHz, CDCl_3_) δ 7.78 (s, 1H), 7.36 (d, *J* = 7.9 Hz, 1H), 7.24 (d, *J* = 8.0 Hz, 1H), 7.07–7.04 (m, 2H), 7.00–6.97 (m, 1H), 6.00–5.98 (m, 1H), 4.93 (dd, *J* = 7.8, 4.8 Hz, 1H), 4.83 (d, *J* = 4.8 Hz, 1H), 2.61 (s, 6H), 2.47 (s, 3H), 2.05 (s, 3H); ^13^C NMR (126 MHz, CDCl_3_) δ 195.7, 136.3, 135.4, 131.4, 127.9, 122.5, 120.7, 119.0, 118.4, 116.5, 113.4, 110.6, 109.4, 60.8, 58.8, 28.4, 25.0, 12.1, −2.1; IR (cm^–1^): 3388, 3246, 3060, 3003, 1773, 1698, 1622, 1584, 1354, 1273, 1217, 1193, 1129, 1083, 1017.

## Supplementary Material



## Data Availability

The data underlying thus study are available in the published article and its Supporting Information.

## Notes

These data can be obtained free of charge from The Cambridge Crystallographic Data Centre via www.ccdc.cam.ac.uk/structures
  Experimental procedures, compound characterization, computational tables, copies of NMR spectra, and crystallographic data (PDF)
