# Supplementary material for: Nucleophilic Substitution of 1,3-Diiodobicyclo[1.1.1]pentane: Synthesis of Bicyclo[1.1.1]pentylpyridinium, Quinolinium, Isoquinolinium, and Pyrazolium Salts
Source: J Org Chem. 2025 May 27;90(23):7712–22. doi: 10.1021/acs.joc.5c00565 (PMC12172055; doi:10.1021/acs.joc.5c00565)

Supporting Information

Nucleophilic substitution of 1,3-diiodobicyclo[1.1.1]pentane: synthesis of bicyclo[1.1.1]pentylpyridinium, quinolinium, isoquinolinium and pyrazolium salts

Harvey J. C. Monroe,[a] Dolapo J. Bello,[a] Bradley J. Duff,[a] Mark R. J. Elsegood,[a] Kohei Watanabe,[b] Gareth J. Pritchard*[a] and Marc C. Kimber*[a]

[a] Department of Chemistry, School of Science, Loughborough University, Ashby Road, Loughborough, LE11 3TU, UK

[b] Faculty of Education, Chiba University, 1-33, Yayoi-cho, Inage-ku, Chiba 263-8522, Japan

*M.C.Kimber@lboro.ac.uk, G.J.Pritchard@lboro.ac.uk

1. Crystallography S2
2. Mechanistic reaction S18
3. DFT calculations S19
4. ^{1}H and ^{13}C NMR data S28

Crystallography

X ray data for **10c**, **10f**, **10g**, **11** and **14a**.

Diffraction data for **10c**, **10f**, **10g**, **11** and **14a** were collected on Rigaku FRE+ diffractometers equipped with Arc)Sec VHF or HF Varimax confocal mirrors and HyPix 6000HE or Arc-100 detectors. Data were corrected for polarisation and Lp effects and for absorption. The structures were solved using a dual-space algorithm (SHELXT)[1] and refined on F^2 using SHELXL.[2] H atoms were included using a riding model. CCDC 2375795 (for **10c**), 2375794 (for **10f**), 2308435 (for **10g**), 2427180 (for **11**), and 2427181 (for **14a**) contain the supplementary crystallographic data for this paper. These data can be obtained free of charge from The Cambridge Crystallographic Data Centre via www.ccdc.cam.ac.uk/structures.

Tabulated data, figures, and structure specifics are given blow.

For **10c**: $[C_{16}H_{15}IN^{+}][I^{-}][I_2]_{0.125}$. There are 4 propellane molecules, 4 iodide ions, {I(5), I(6), I(9), plus I(8) or I(10)} and half an iodine I_2 molecule {I(7) & I(8)} in the asymmetric unit. The situation is complex since not all iodides are full weight. Two, non-coordinated iodines were modelled at half weight following investigation of refined occupancy factors for all iodines. These are I(7) and I(10). This suggests that I(7)/I(8) are sometimes neutral I_2, when I(7) is occupied, in which case I(10) is I^{-}, and sometimes I^{-} when I(8) is fully occupied and I(7) and I(10) are vacant. Each coordinated I forms a linear interaction with an iodide ion. Sometimes that iodide ion bridges two propellane molecules. There are many C–H···I interactions from both the propellane CH_2 and some aromatic ring hydrogens.

Twist angles between the two aromatic rings:

About C(8)–C(11) = 6.55(14)°

About C(24)–C(27) = 13.6(4)°

About C(40)–C(43) = 33.95(18)°

About C(56)–C(59) = 26.9(2)°

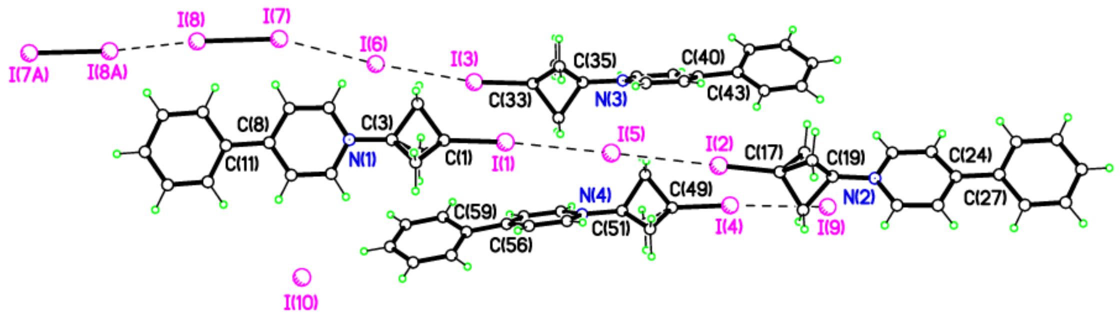

Fig. S1. The asymmetric unit of **10c**.

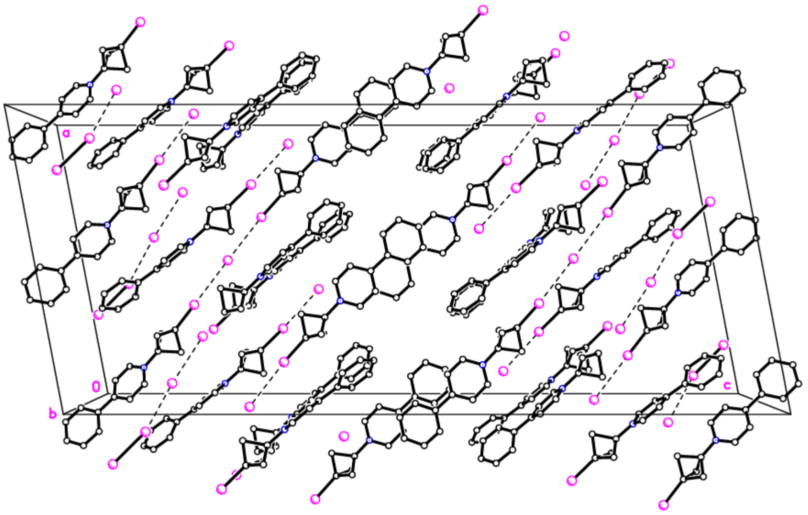

Fig. S2. Packing plot for **10c** viewed parallel to *b*.

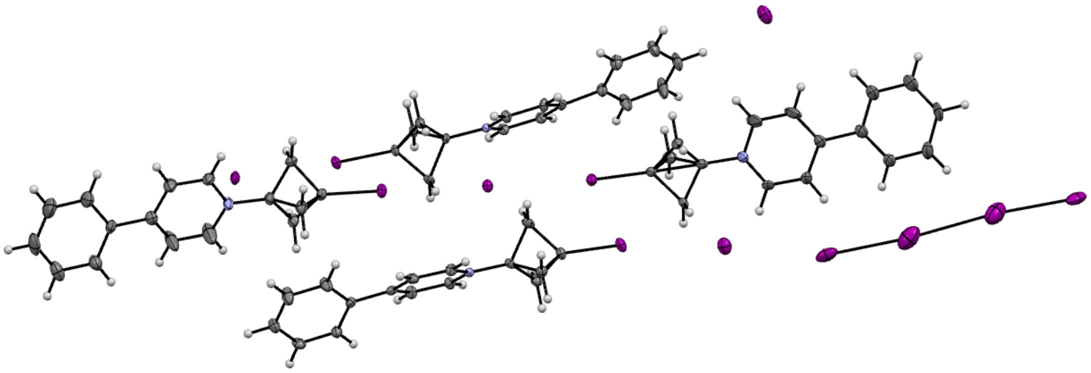

Fig S3. Thermal epilsoid plot of **10c** (anisotropic displacement ellipsoids are at the 50% probability level).

Table S1

*Experimental details for **10c**.*

Crystal data	
Chemical formula	$C_{16}H_{15}IN^{+}\cdot I^{-}\cdot I_{0.25}$
M_r	506.81
Crystal system, space group	Monoclinic, *I*2/*a*
Temperature (K)	100
a, *b*, *c* (Å)	19.3371 (4), 15.2755 (3), 44.6145 (8)
β (°)	100.7945 (18)
V (Å^3)	12945.2 (4)
Z	32
Radiation type	Mo *K*α
μ (mm^{-1})	4.35
Crystal size (mm^3)	0.28 × 0.02 × 0.02
Data collection	
Diffractometer	Rigaku FRE+ equipped with Arc)Sec VHF Varimax confocal mirrors and an UG2 goniometer and HyPix 6000HE detector
Absorption correction	Multi-scan. *CrysAlis PRO* 1.171.42.74a (Rigaku Oxford Diffraction, 2022). Empirical absorption correction using spherical harmonics, implemented in SCALE3 ABSPACK scaling algorithm.
T_{min}, T_{max}	0.866, 1.000
No. of measured, independent and observed [$I > 2\sigma(I)$] reflections	96273, 19769, 14211
R_{int}	0.054
$(\sin \theta/\lambda)_{max}$ (Å^{-1})	0.714
Refinement	
$R[F^2 > 2\sigma(F^2)]$, $wR(F^2)$, S	0.041, 0.106, 1.03
No. of reflections	19769
No. of parameters	699
H-atom treatment	H-atom parameters constrained
	$w = 1/[\sigma^2(F_o^2) + (0.0511P)^2 + 49.2002P]$ where $P = (F_o^2 + 2F_c^2)/3$
Δ〉max, Δ〉min (e Å^{-3})	3.14, -3.60

Computer programs: *CrysAlis PRO* 1.171.42.74a (Rigaku OD, 2022), SHELXT-2018/2 (Sheldrick, 2015), *SHELXL2018*/3 (Sheldrick, 2018), Bruker *SHELXTL*.

For **10f**: $C_{11}H_{13}INO^{+}\cdot I^{-}$. The cation and anion lie on a mirror plane, so half the formula is unique. Only C(2)/H(2A)/H(2B), H(4A), and H(10B) lie off the mirror plane. There was one substantial residual electron density peak close to O(1), but this could not be modelled/rationalised as disorder in that group due to unfeasible geometry and the complications of the mirror plane. In the *a* direction, molecules are linked via head-to-tail pairs of $(CH_2)C$–H···I interactions giving rise to a zig-zag ladder motif. In the *c* direction molecules interact via (Ar)C–H···I/O contacts. The cation/anion I···I (3.5874(4) Å) interaction is in the *b* direction. Overall, a weakly bonded 3D network.

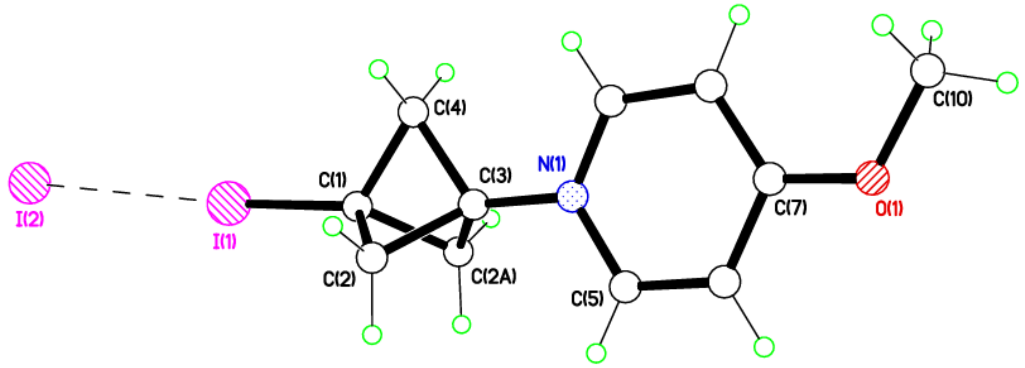

Fig. S4. A cation/anion pair in **10f**.

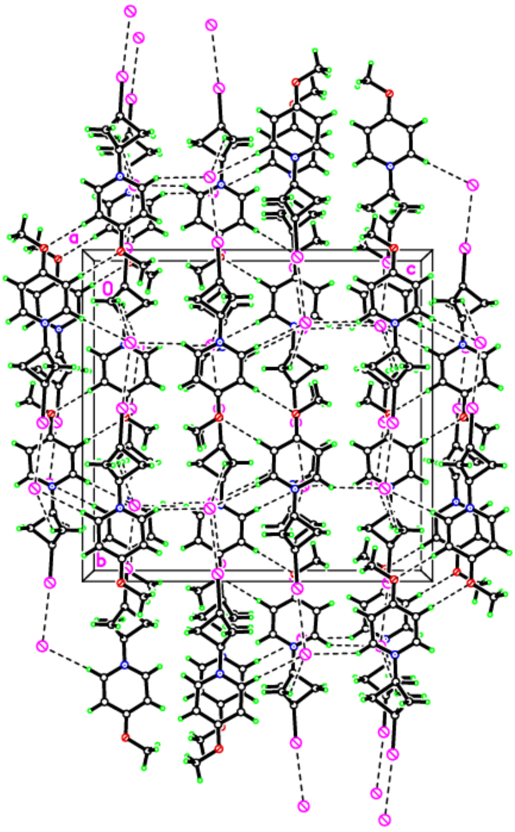

Fig. S5. Packing plot of **10f** viewed parallel to *a*.

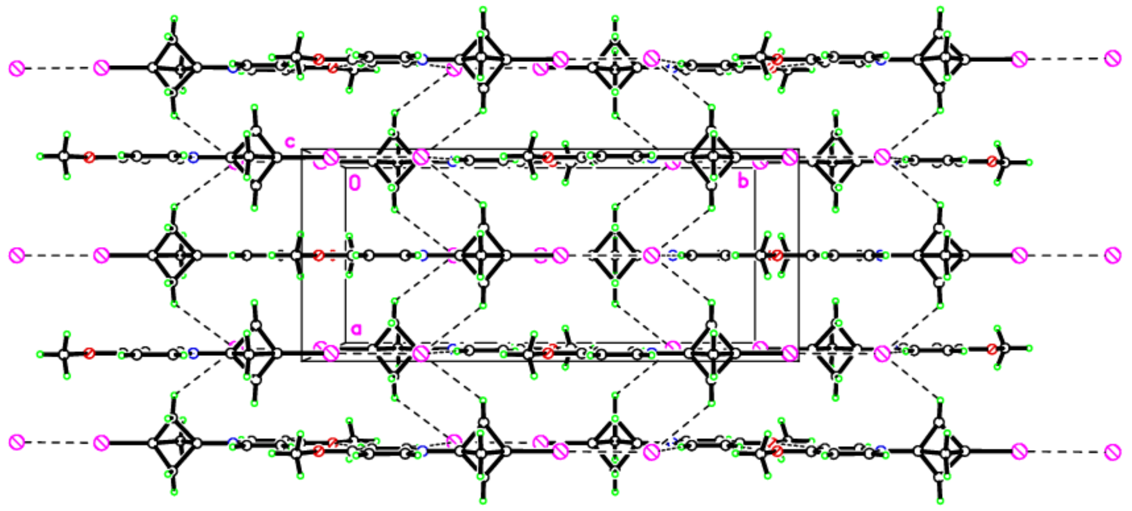

Fig. S6. Packing plot of **10f** viewed parallel to *c*. The mirror is in the *b*/*c* plane.

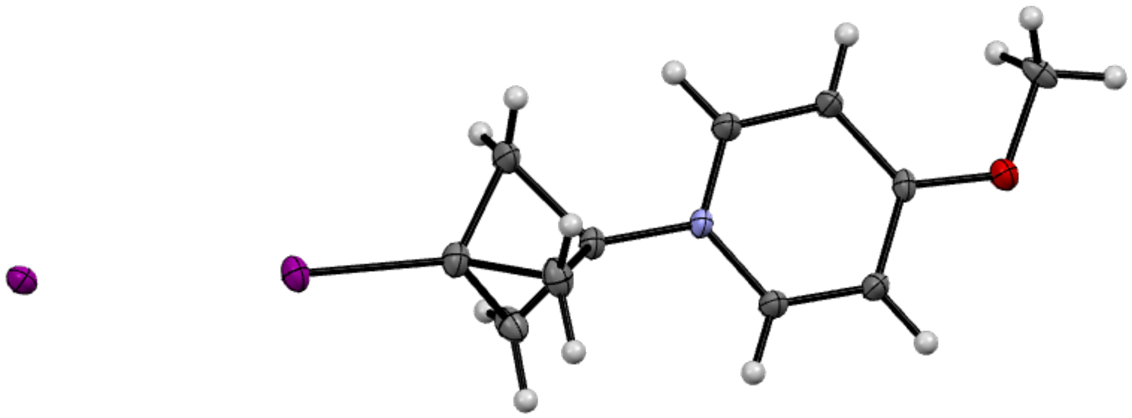

Fig S7. Thermal epilsoid plot of **10f** (anisotropic displacement ellipsoids are at the 50% probability level).

Table S2

*Experimental details for10**4f**.*

Crystal data	
Chemical formula	$C_{11}H_{13}INO^{+}\cdot I^{-}$
M_r	429.02
Crystal system, space group	Orthorhombic, *Cmca*
Temperature (K)	100

a, *b*, *c* (Å)	7.6795 (3), 17.9788 (5), 19.0014 (6)
V ($Å^3$)	2623.49 (15)
Z	8
Radiation type	Mo *K*α
μ (mm^{-1})	4.77
Crystal size (mm^3)	0.10 × 0.07 × 0.02
Data collection	
Diffractometer	Rigaku FRE+ equipped with Arc)Sec VHF Varimax confocal mirrors and an UG2 goniometer and HyPix 6000HE detector
Absorption correction	Multi-scan. *CrysAlis PRO* 1.171.43.124a (Rigaku Oxford Diffraction, 2024). Empirical absorption correction using spherical harmonics, implemented in SCALE3 ABSPACK scaling algorithm.
T_{min}, T_{max}	0.887, 1.000
No. of measured, independent and observed [$I > 2\sigma(I)$] reflections	18573, 3621, 2990
R_{int}	0.046
$(\sin \theta/\lambda)_{max}$ ($Å^{-1}$)	0.867
Refinement	
$R[F^2 > 2\sigma(F^2)]$, $wR(F^2)$, S	0.042, 0.096, 1.08
No. of reflections	3621
No. of parameters	88
H-atom treatment	H-atom parameters constrained
	$w = 1/[\sigma^2(F_o^2) + (0.0315P)^2 + 24.1642P]$ where $P = (F_o^2 + 2F_c^2)/3$
$\Delta\rangle_{max}$, $\Delta\rangle_{min}$ (e $Å^{-3}$)	2.95, -1.99

Computer programs: *CrysAlis PRO* system (CCD 43.124a 64-bit (release 23-05-2024)), *CrysAlis PRO* 1.171.43.124a (Rigaku OD, 2024), SHELXT 2018/2 (Sheldrick, 2018), *SHELXL2019*/3 (Sheldrick, 2019), Bruker *SHELXTL*.

For **10g**: $C_{13}H_{15}I_2NO_2 \cdot 0.5(H_2O)$. Two cations, two anions and one water molecule in the asymmetric unit. The ethyl groups have different conformations in the two unique molecules; in one that group is approx. co-planar with the aromatic ring, while it is

twisted out of that plane in the other. The C(1)···C(3) and C(14)···C(16) distances are very similar at 1.819(16) and 1.817(17) Å, respectively. The structure was refined as a 2-component twin with twin ratio 0.1877:0.8123 (10). Twinned via a 180° rotation about direct axis [0 0 1]. One water molecule of crystallisation is the most likely explanation for the one residual electron density peak, given that methanol was used in the crystallisation & acetone in the synthesis, both of which are often wet. The amount of electron density also tallied with an oxygen atom, but H atoms could not be located so were not included in the model and may well be disordered in any case. The O atom occupancy was initially refined freely and came to approx. 1 before being fixed at 1.0. Iodide anions are 3.8076(11) and 3.6078(10) Å from the coordinated iodine for cation/anion pairs I(1)/I(3) and I(2)/I(4), respectively. I(4)···N(1') = 3.590 Å; a fairly long intermolecular contact.

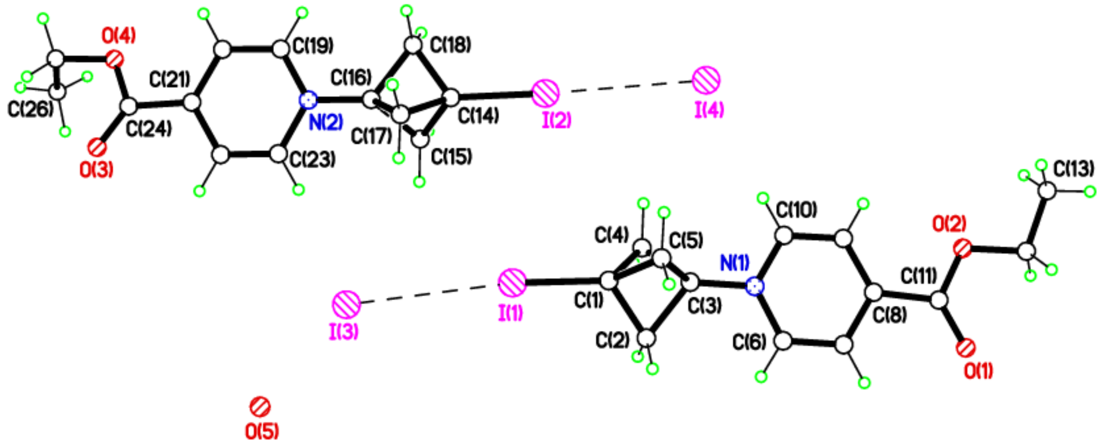

Fig. S8. The asymmetric unit in **10g**.

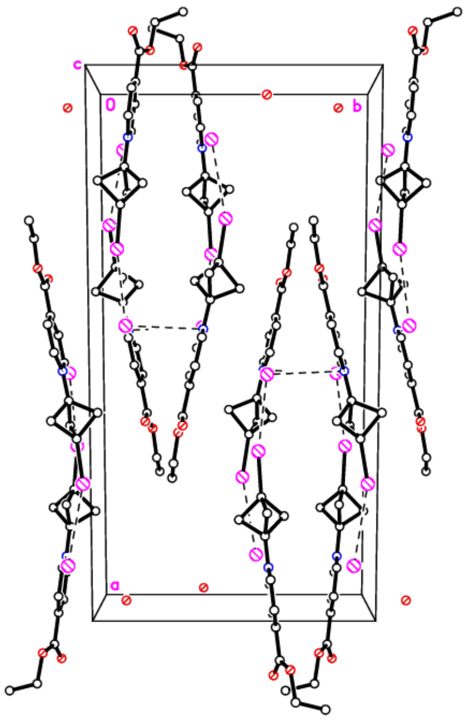

Fig. S9. Packing plot of **10g** viewed parallel to *c*.

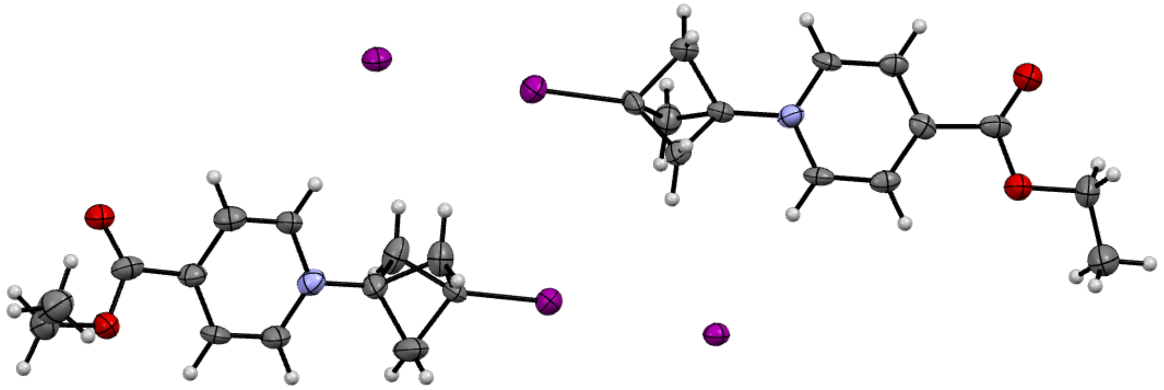

Fig S10. Thermal epilsoid plot of **10g** (anisotropic displacement ellipsoids are at the 50% probability level).

Table S3

*Experimental details for **10g**.*

Crystal data	
Chemical formula	$C_{13}H_{15}I_2NO_2{\cdot}0.5(H_2O)$
M_r	480.07

Crystal system, space group	Monoclinic, $P2_1/c$
Temperature (K)	100
a, *b*, *c* (Å)	25.4758 (15), 13.0990 (7), 9.6987 (6)
β (°)	100.065 (7)
V (Å^3)	3186.7 (3)
Z	8
Radiation type	Cu $K\alpha$
μ (mm^{-1})	31.01
Crystal size (mm^3)	0.11 × 0.03 × 0.02
Data collection	
Diffractometer	Rigaku 007HF diffractometer with HF Varimax confocal mirrors, an UG2 goniometer and HyPix Arc-100 detector
Absorption correction	Multi-scan. *CrysAlis PRO* 1.171.43.124a (Rigaku Oxford Diffraction, 2024). Empirical absorption correction using spherical harmonics, implemented in SCALE3 ABSPACK scaling algorithm.
T_{min}, T_{max}	0.402, 1.000
No. of measured, independent and observed [$I > 2\sigma(I)$] reflections	10140, 10140, 8177
R_{int}	0.087
$(\sin \theta/\lambda)_{max}$ (Å^{-1})	0.622
Refinement	
$R[F^2 > 2\sigma(F^2)]$, $wR(F^2)$, S	0.063, 0.185, 1.05
No. of reflections	10140
No. of parameters	338
H-atom treatment	H-atom parameters constrained
	$w = 1/[\sigma^2(F_o^2) + (0.1174P)^2 + 11.7748P]$<br>where $P = (F_o^2 + 2F_c^2)/3$
$\Delta\rangle_{max}$, $\Delta\rangle_{min}$ (e Å^{-3})	1.76, -1.80

Computer programs: *CrysAlis PRO* system (CCD 43.124a 64-bit (release 23-05-2024)), *CrysAlis PRO* 1.171.43.124a (Rigaku OD, 2024), SHELXT-2018/2 (Sheldrick, 2015), *SHELXL2019*/3 (Sheldrick, 2019), Bruker *SHELXTL*.

For **11** $C_{9.97}H_{10.95}ClI_{0.03}N^+\cdot I_3^-$. One cation & one anion in the asymmetric unit. The C(1)=C(2) bond length clearly denotes a double bond. An analysis of the residual

electron density peaks revealed a small residual amount of iodine in place of the =CH_2 group with relative occupancies of 0.0272:0.9728(11). Otherwise this structure refined routinely. Several weak C–H···I interactions and also a short contact Cl(1)···I(1') = 3.532 Å. Overall a 3D network of such interactions.

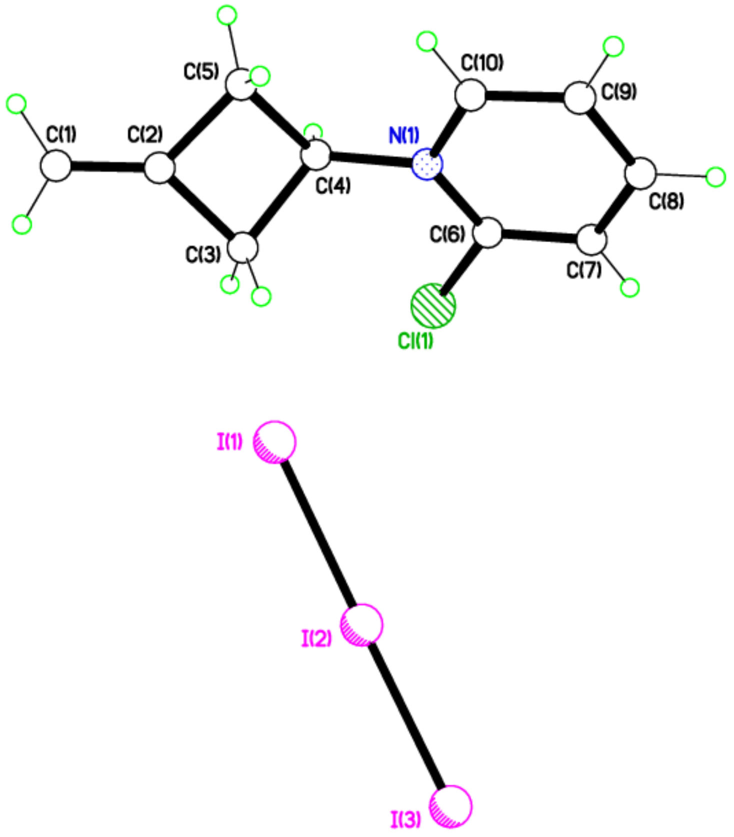

Fig. S11. The asymmetric unit in **11**.

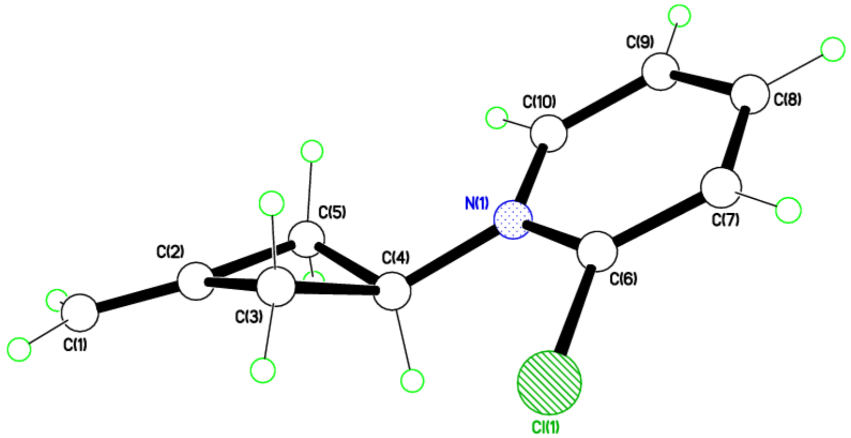

Fig. S12. Side view of the cation in **11**.

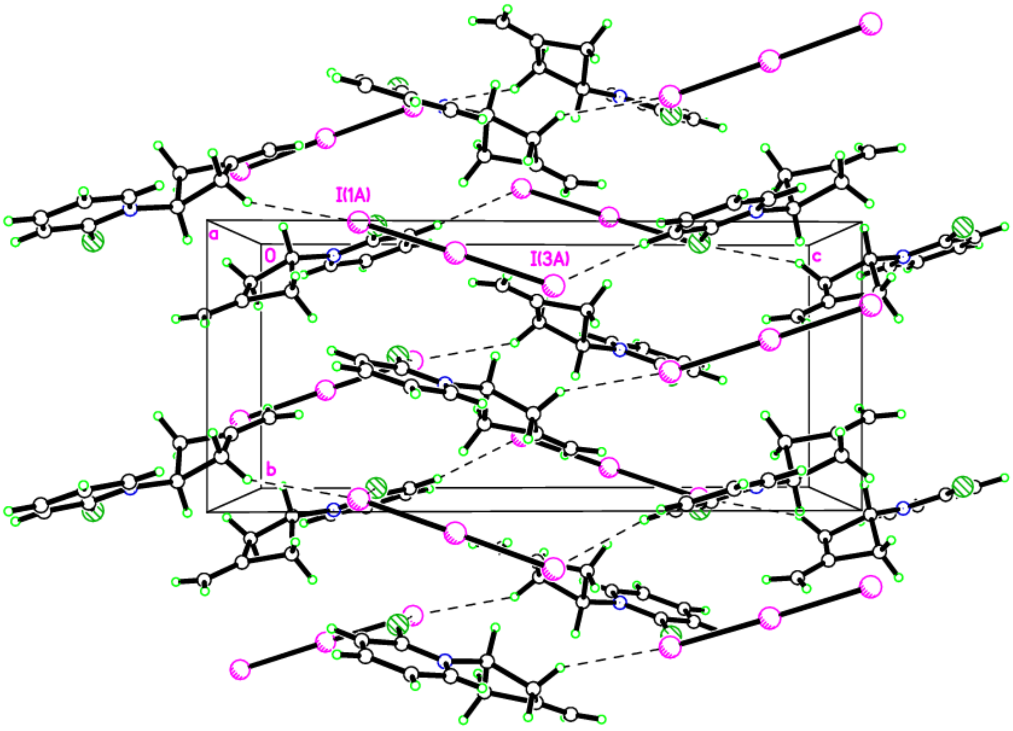

Fig. S13. Packing plot of **11** viewed parralel to *a*.

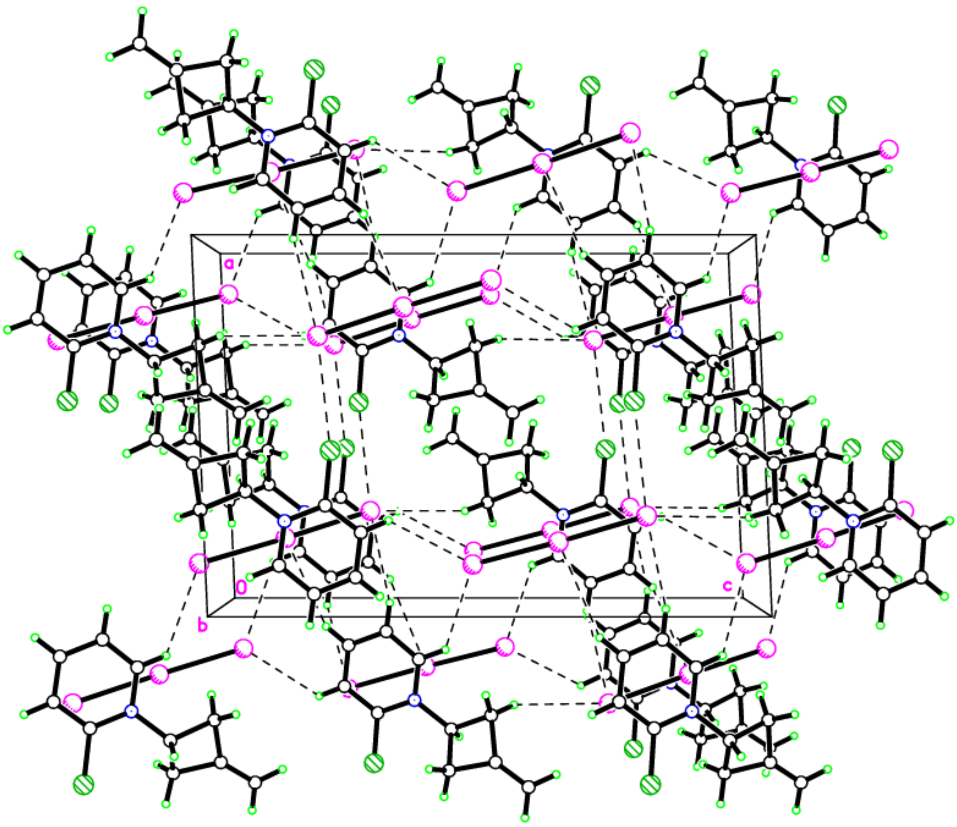

Fig. S14. Packing plot of **11** viewed parralel to *b*.

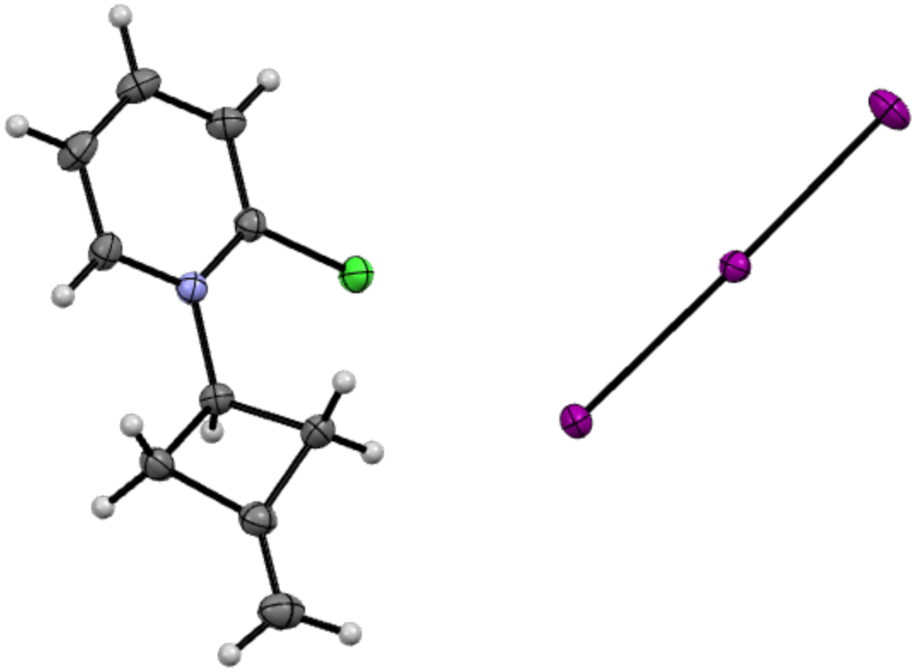

Fig. S15. Thermal epilsoid plot of **11** (anisotropic displacement ellipsoids are at the 50% probability level).

Table S4

Experimental details for ***11****.*

Crystal data	
Chemical formula	$C_{9.97}H_{10.95}ClI_{0.03}N^{+}\cdot I_3^{-}$
M_r	564.41
Crystal system, space group	Monoclinic, $P2_1/c$
Temperature (K)	100
a, *b*, *c* (Å)	11.67702 (17), 7.62972 (11), 17.2084 (2)
β (°)	92.4257 (13)
V (Å^3)	1531.76 (4)
Z	4
Radiation type	Mo $K\alpha$
μ (mm^{-1})	6.32
Crystal size (mm^3)	0.32 × 0.04 × 0.02
Data collection	
Diffractometer	Rigaku FRE+ equipped with Arc)Sec VHF Varimax confocal mirrors and an UG2 goniometer and HyPix 6000HE detector.
Absorption correction	Gaussian. *CrysAlis PRO* 1.171.43.95a (Rigaku Oxford Diffraction, 2023). Numerical absorption correction based on gaussian integration over a multifaceted crystal model Empirical absorption correction using spherical harmonics, implemented in SCALE3 ABSPACK scaling algorithm.
T_{min}, T_{max}	0.792, 1.000
No. of measured, independent and observed [$I > 2\sigma(I)$] reflections	73930, 8079, 6776
R_{int}	0.040
$(\sin \theta/\lambda)_{max}$ (Å^{-1})	0.870
Refinement	
$R[F^2 > 2\sigma(F^2)]$, $wR(F^2)$, S	0.028, 0.059, 1.07
No. of reflections	8079
No. of parameters	141
No. of restraints	22
H-atom treatment	H-atom parameters constrained
$\Delta\rangle_{max}$, $\Delta\rangle_{min}$ (e Å^{-3})	2.34, -1.52

Computer programs: *CrysAlis PRO* system (CCD 43.95a 64-bit (release 03-11-2023)), *CrysAlis PRO* 1.171.43.95a (Rigaku OD, 2023), SHELXT-2018/2 (Sheldrick, 2015), *SHELXL2019*/3 (Sheldrick, 2019), Bruker *SHELXTL*.

For **14a** [$C_9H_{12}IN_2^+$][I^-]. This is the asymmetric unit with one cation and one anion. Linear arrangement of cation and anion with an I(1)···I(2) separation of 3.754 Å. Typical monoclinic herringbone arrangement of cations/anions within the unit cell. Routine structure refinement.

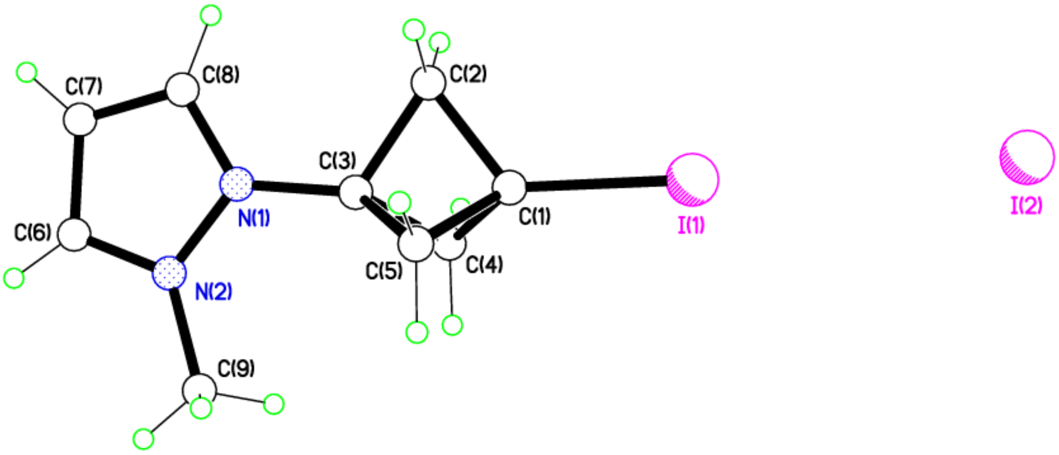

Fig. S16. The asymmetric unit in **14a**

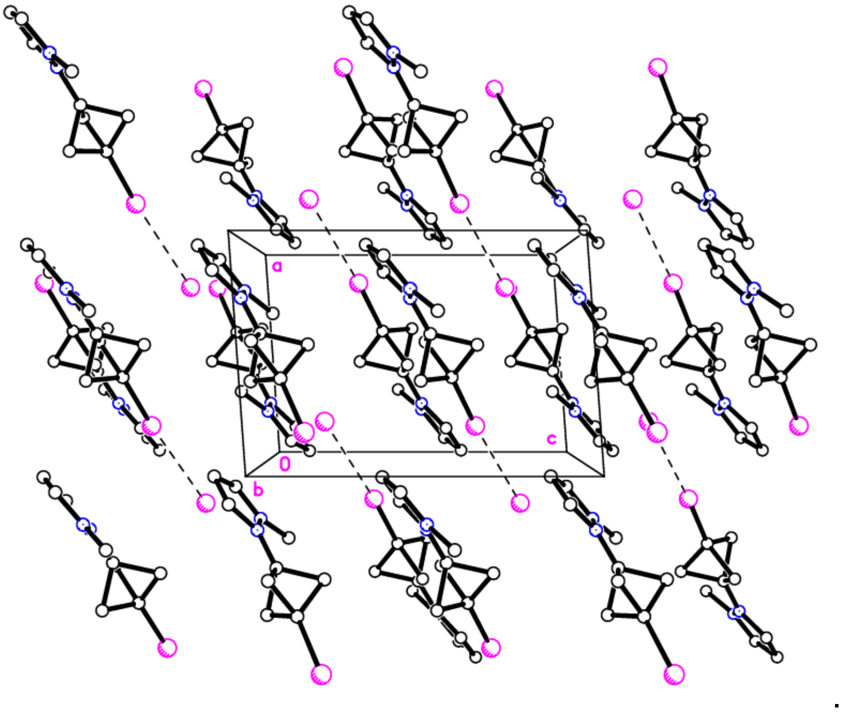

Fig. S17. Packing plot of **14a** viewed parralel to *b*.

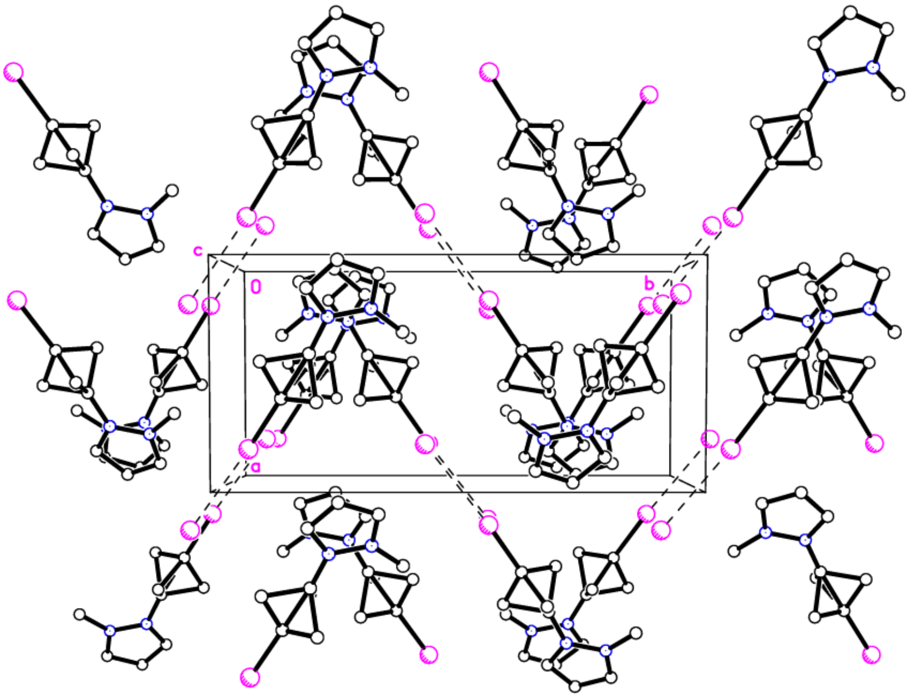

Fig. S18. Packing plot of **14a** viewed parralel to *c* showing herringbone arrangement.

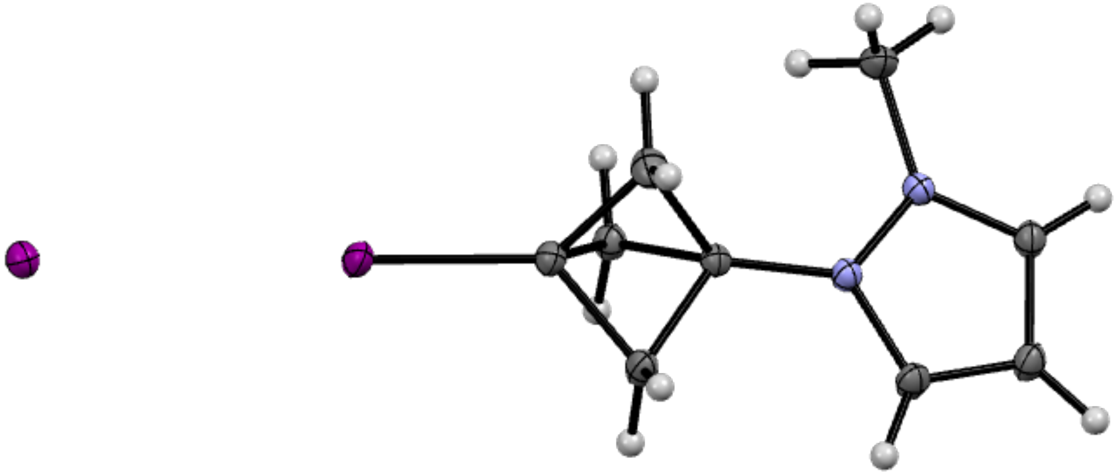

Fig. S19. Thermal epilsoid plot of **14a** (anisotropic displacement ellipsoids are at the 50% probability level).

Table S5.

*Experimental details for **14a**.*

Crystal data

Chemical formula	$C_9H_{12}IN_2^{+}\cdot I^{-}$
M_r	402.01
Crystal system, space group	Monoclinic, $P2_1/c$
Temperature (K)	100
a, *b*, *c* (Å)	7.37128 (6), 15.35216 (13), 10.70049 (9)
β (°)	94.0578 (8)
V (Å^3)	1207.89 (2)
Z	4
Radiation type	Mo *K*α
μ (mm^{-1})	5.17
Crystal size (mm^3)	0.10 × 0.08 × 0.04
Data collection	
Diffractometer	Rigaku FRE+ equipped with Arc)Sec VHF Varimax confocal mirrors and an UG2 goniometer and HyPix 6000HE detector
Absorption correction	Multi-scan *CrysAlis PRO* 1.171.43.130a (Rigaku Oxford Diffraction, 2024). Empirical absorption correction using spherical harmonics, implemented in SCALE3 ABSPACK scaling algorithm.
T_{min}, T_{max}	0.783, 1.000
No. of measured, independent and observed [$I > 2\sigma(I)$] reflections	196983, 22215, 15999
R_{int}	0.042
$(\sin \theta/\lambda)_{max}$ (Å^{-1})	1.306
Refinement	
$R[F^2 > 2\sigma(F^2)]$, $wR(F^2)$, S	0.028, 0.062, 1.03
No. of reflections	22215
No. of parameters	120
H-atom treatment	H-atom parameters constrained
$\Delta\rangle_{max}$, $\Delta\rangle_{min}$ (e Å^{-3})	1.81, -2.46

Computer programs: *CrysAlis PRO* system (CCD 43.130a 64-bit (release 05-07-2024)), *CrysAlis PRO* 1.171.43.130a (Rigaku OD, 2024), SHELXT-2018/2 (Sheldrick, 2015), *SHELXL2019*/3 (Sheldrick, 2019), Bruker *SHELXTL*.

1 References

1. G. M. Sheldrick, *Acta Cryst.*, 2015, **A71,** 3-8.

2. G. M. Sheldrick, *Acta Cryst.*, 2015, **C71,** 3-8.

Mechanistic investigation

To a 10 mL round-bottomed flask, a stirrer bar, **10a** (200 mg, 0.5 mmol, 1 eq.), acetone (1 mL) and 3,5-dimethylpyridine (53 mg, 0.5 mmol, 1 eq.) were added. This mixture was stirred at a low frequency for 3 days at reflux. On completion, the solvent was removed under reduced pressure and the crude product analysed by ^{1}H NMR. Similarly, the reaction was undertaken in methanol (1 mL).

1-(3-Iodobicyclo[1.1.1]pentanyl)pyridinium iodide (10a)

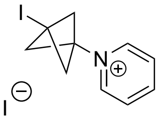

^{1}H NMR (500 MHz, DMSO-d_6)

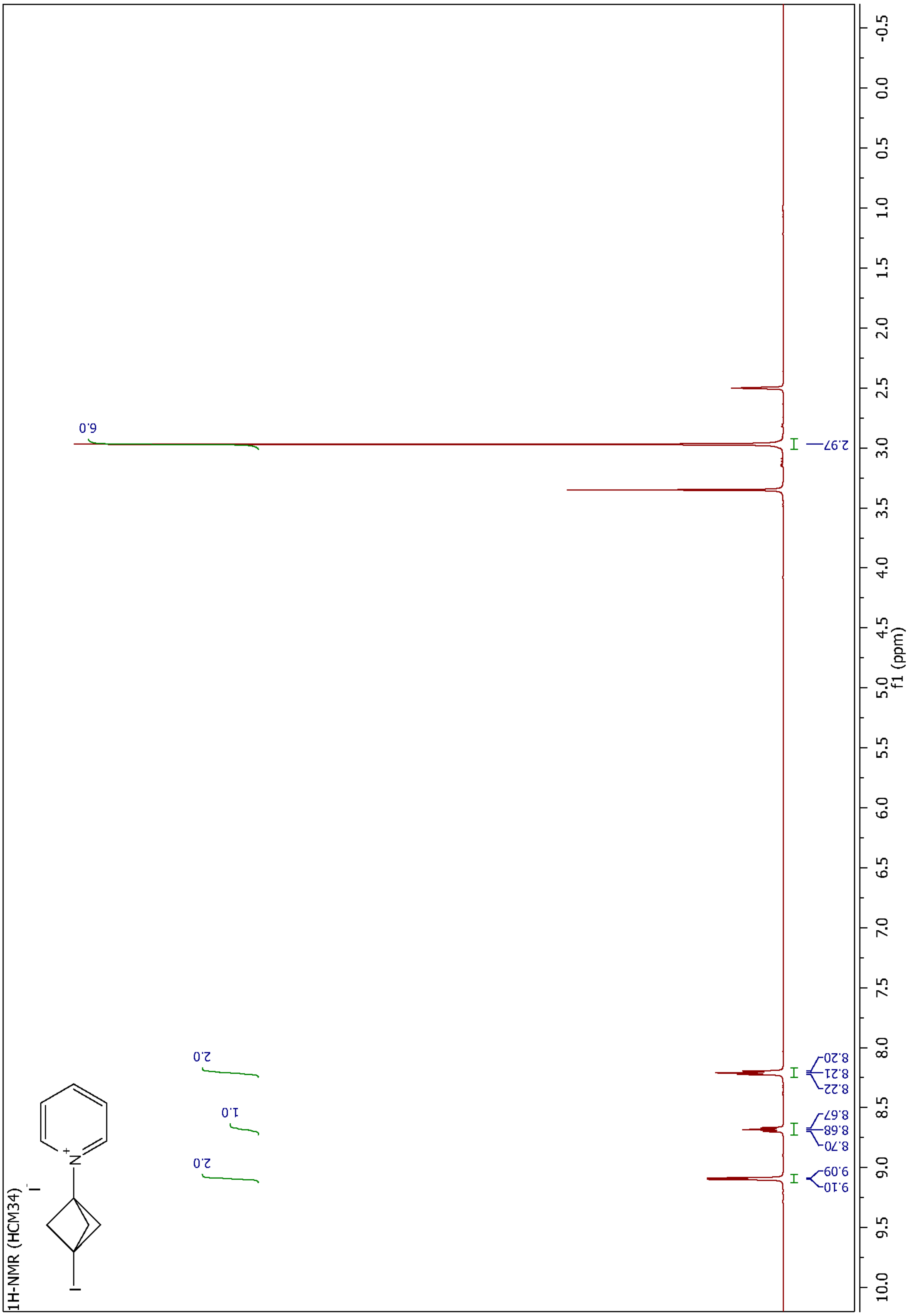

1-(3-Iodobicyclo[1.1.1]pentanyl)pyridinium iodide (10a)

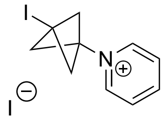

^{13}C NMR (126 MHz, DMSO-d_6)

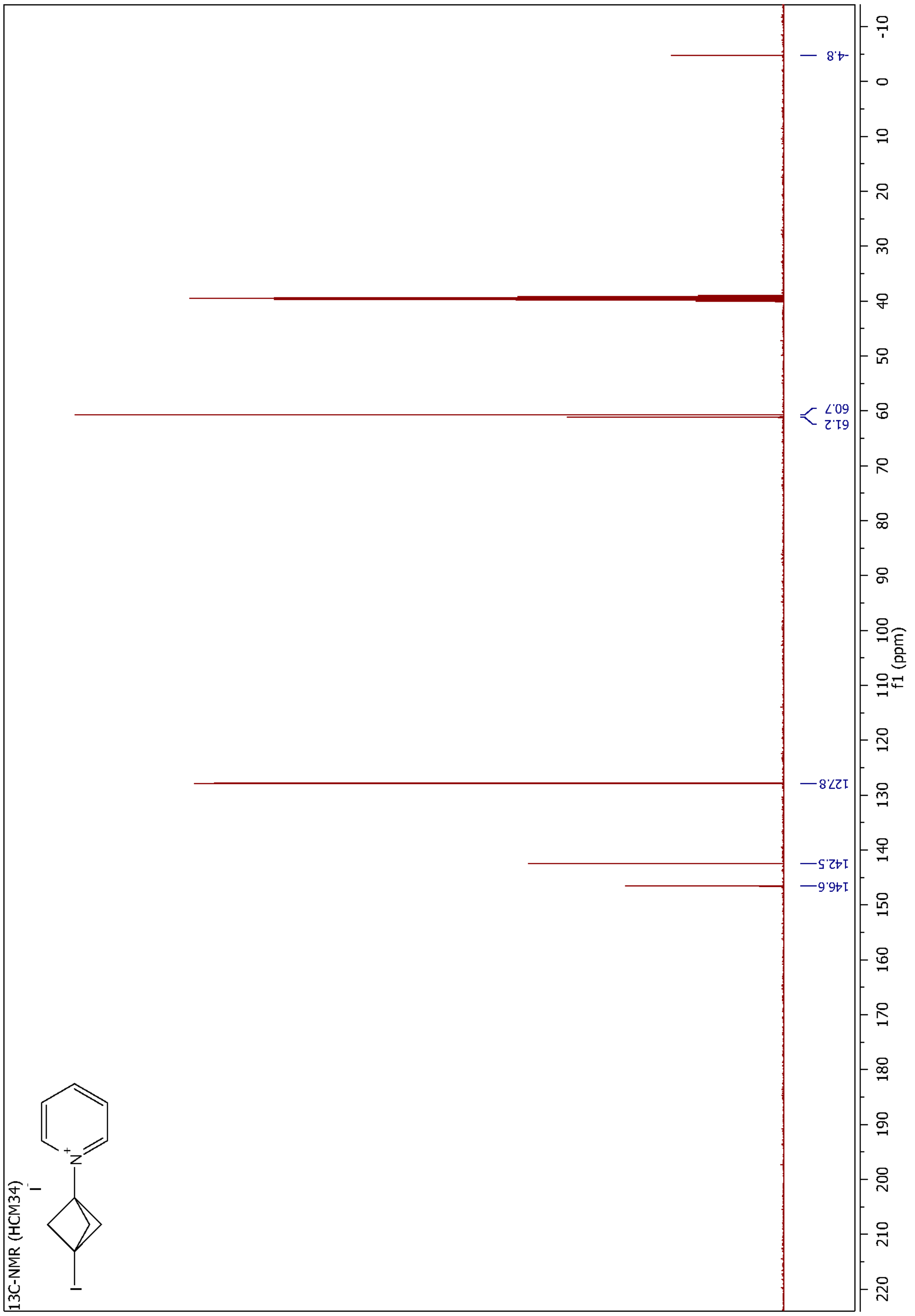

1-(3-Iodobicyclo[1.1.1]pentanyl)-4-methylpyridinium iodide (10b)

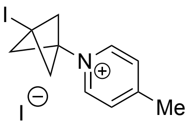

^{1}H NMR (500 MHz, DMSO-d_6)

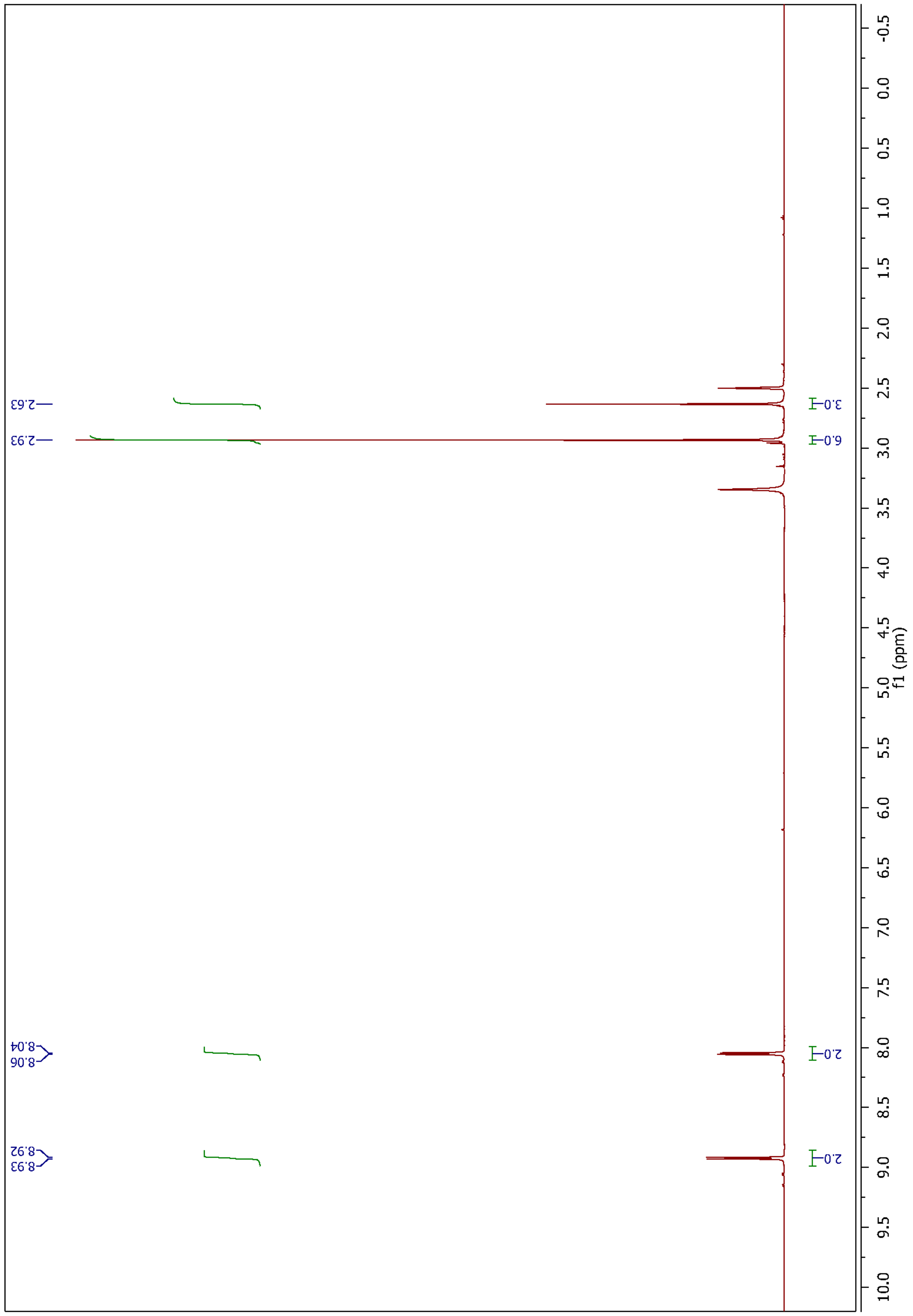

1-(3-Iodobicyclo[1.1.1]pentanyl)-4-methylpyridinium iodide (10b)

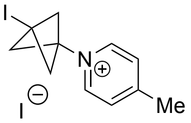

^{13}C NMR (126 MHz, DMSO-d_6)

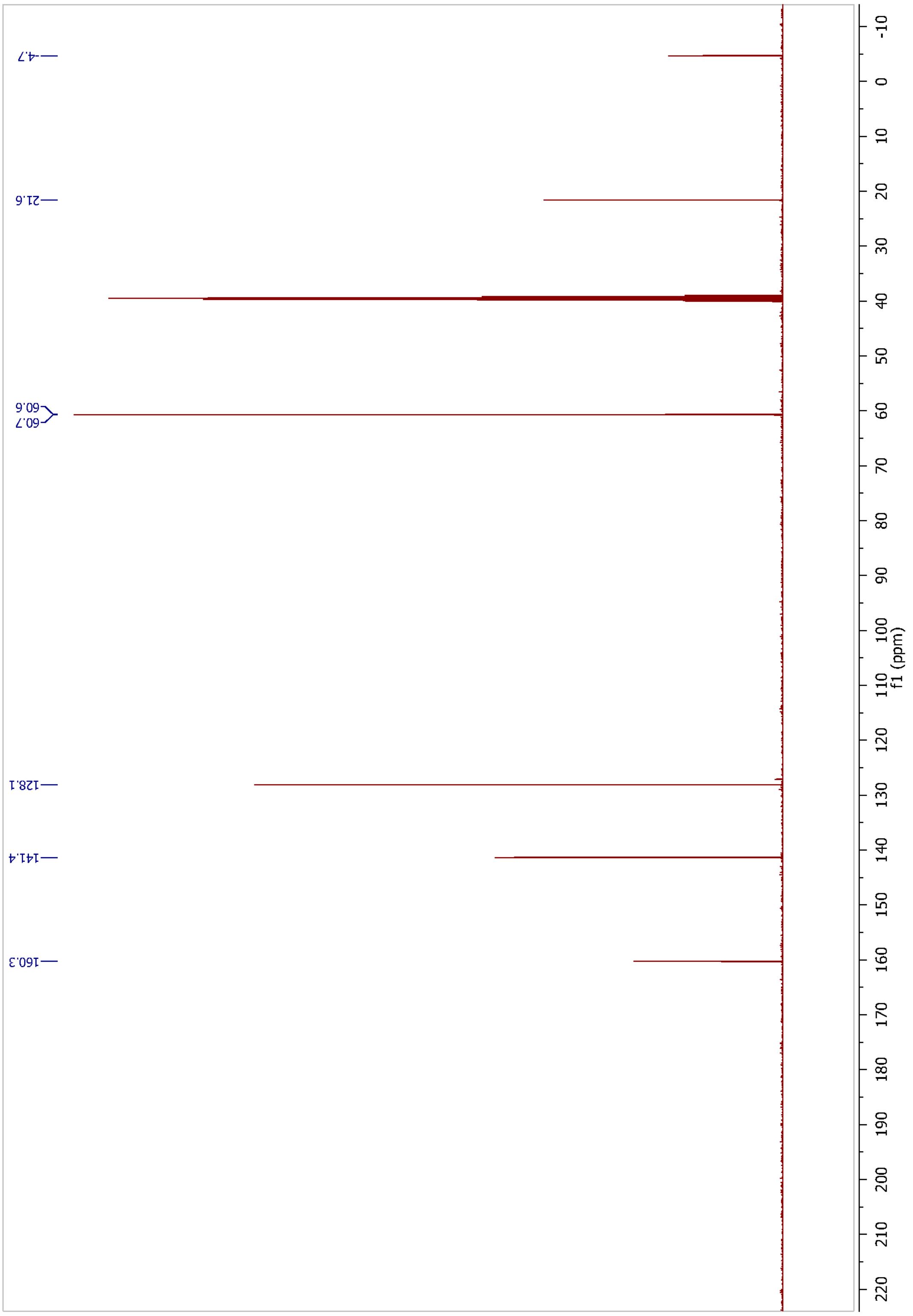

1-(3-Iodobicyclo[1.1.1]pentanyl)-4-ethoxycarbonylpyridinium iodide (10c)

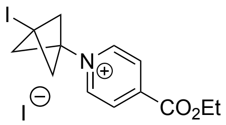

^{1}H NMR (400 MHz, DMSO-d_6)

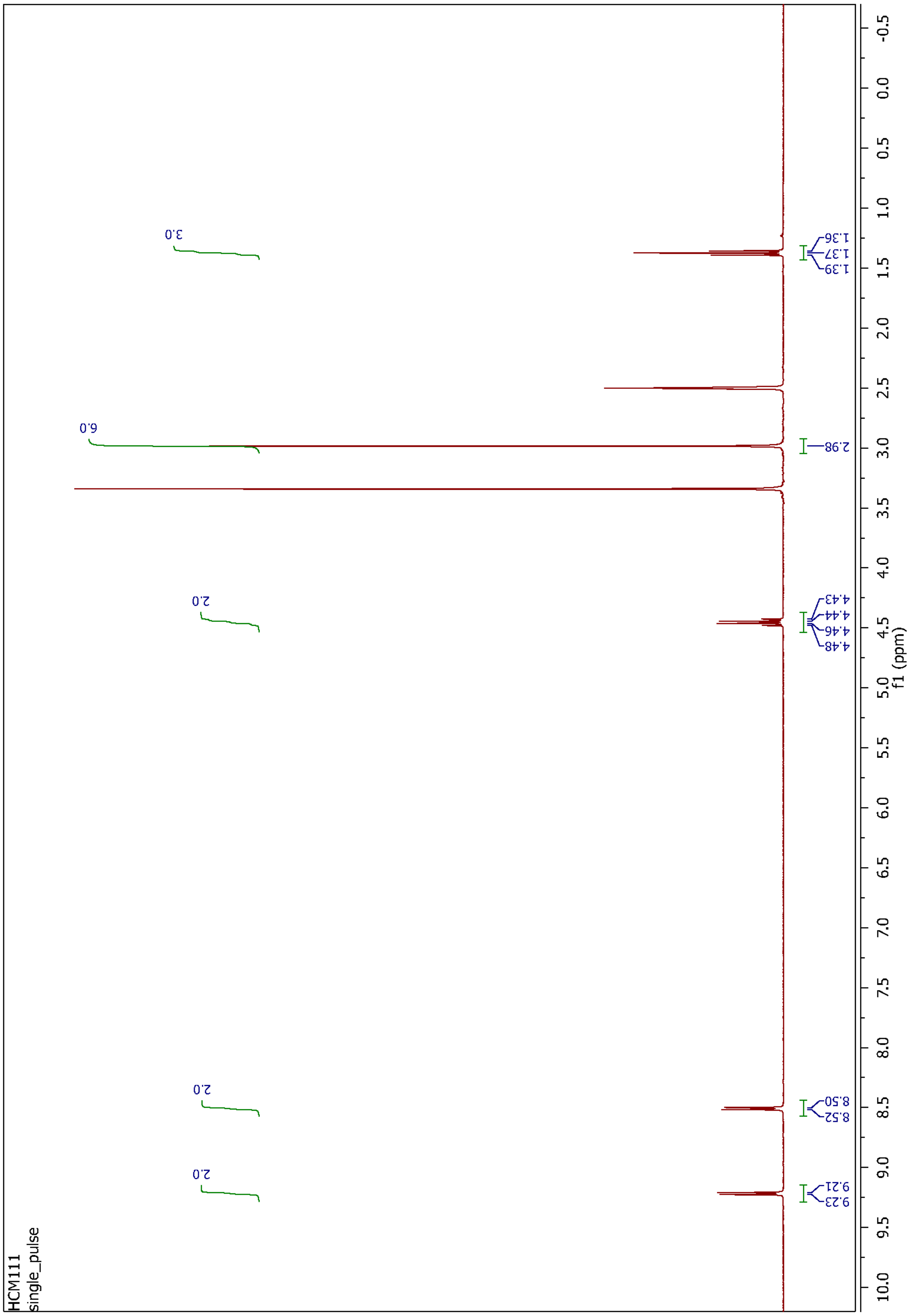

1-(3-Iodobicyclo[1.1.1]pentanyl)-4-ethoxycarbonylpyridinium iodide (10c)

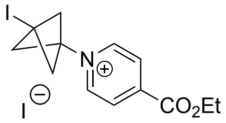

^{13}C NMR (126 MHz, DMSO-d_6)

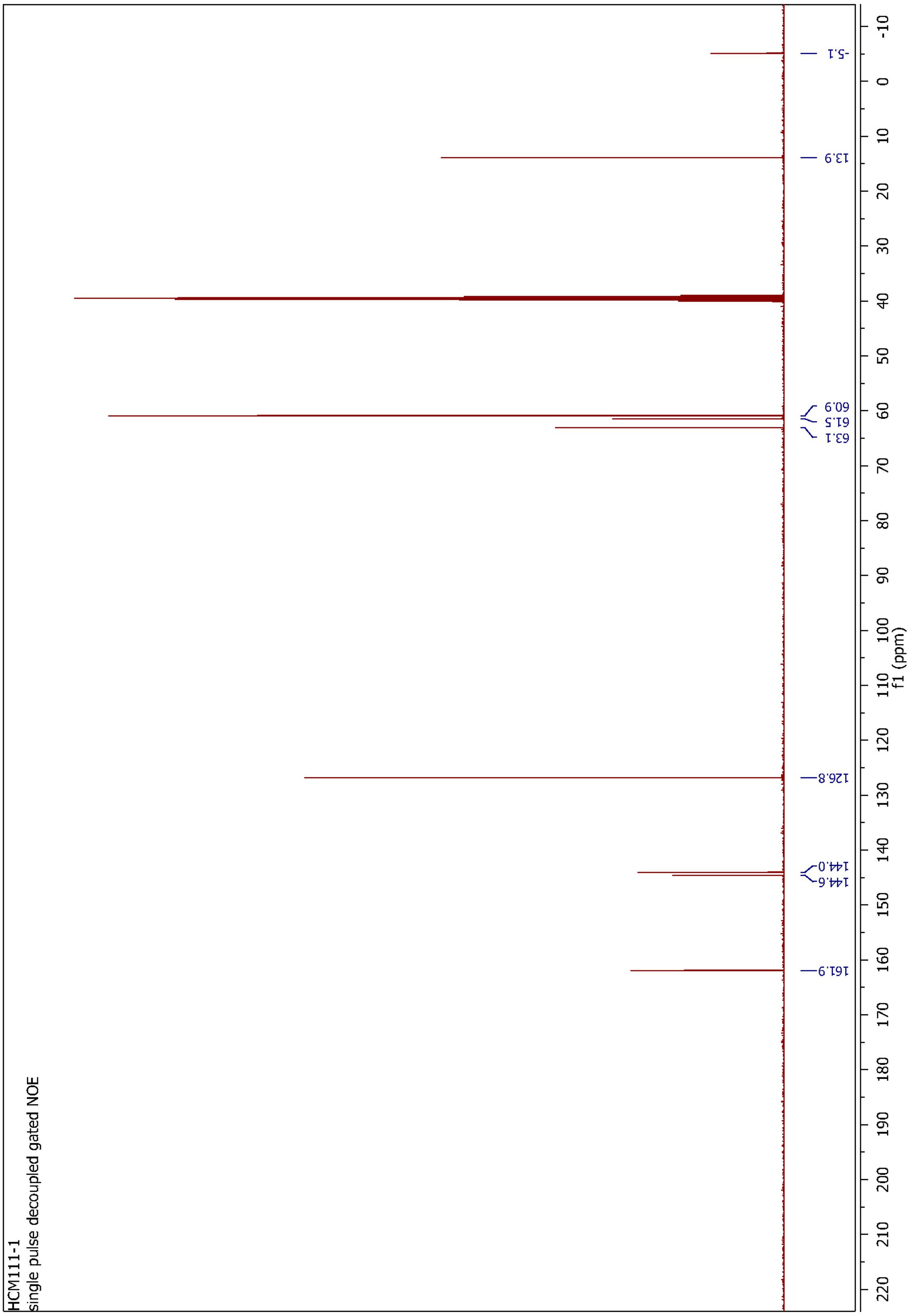

1-(3-Iodobicyclo[1.1.1]pentanyl)-4-cyanopyridinium iodide (10d)

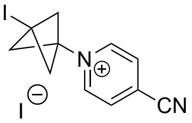

^{1}H NMR (500 MHz, DMSO-d_6)

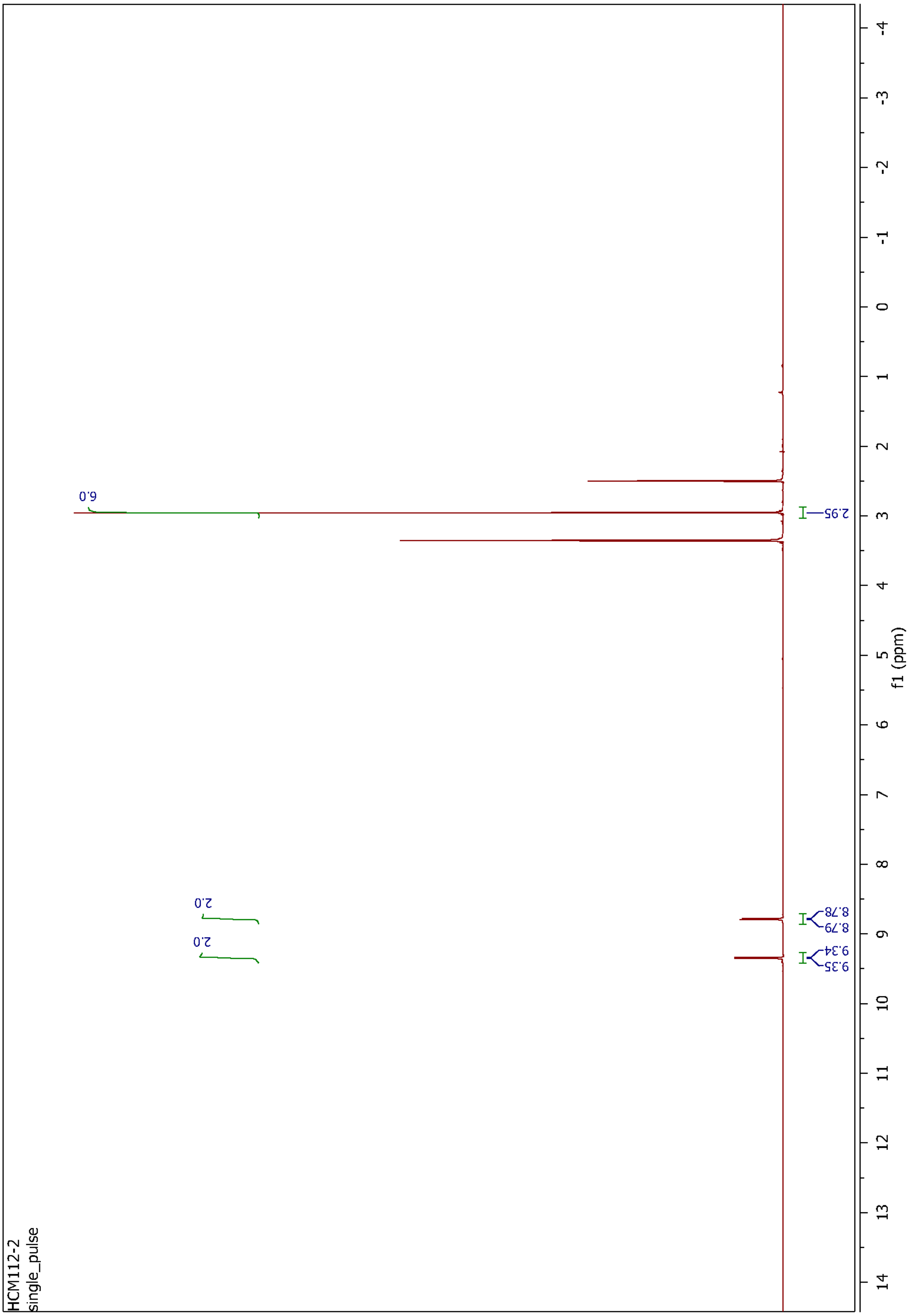

1-(3-Iodobicyclo[1.1.1]pentanyl)-4-cyanopyridinium iodide (10d)

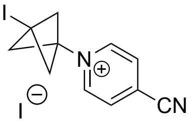

^{13}C NMR (126 MHz, DMSO-d_6)

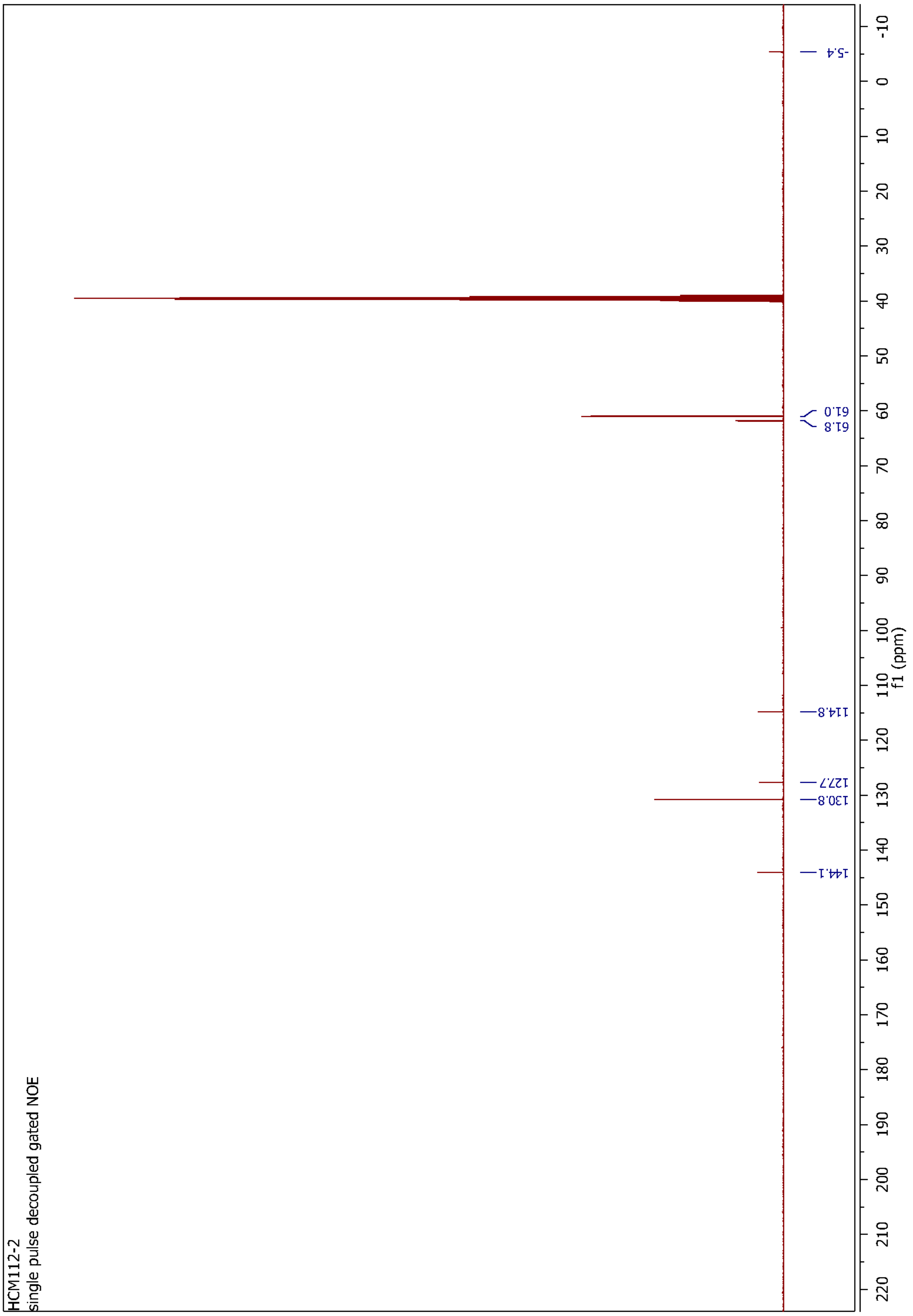

1-(3-Iodobicyclo[1.1.1]pentanyl)-4-trifluoromethylpyridinium iodide (10e)

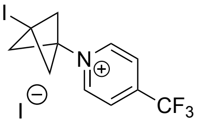

^{1}H NMR (500 MHz, DMSO-d_6)

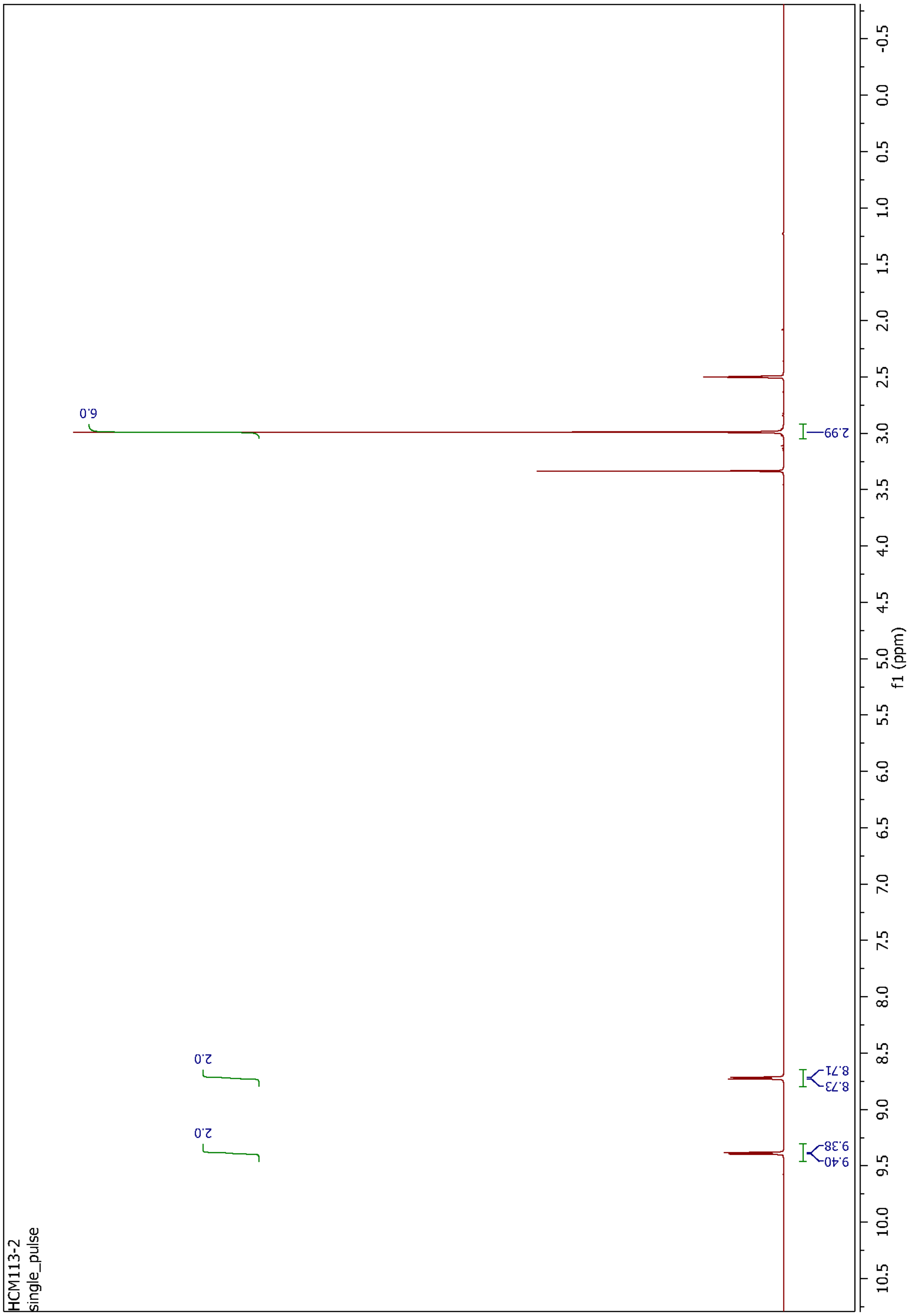

1-(3-Iodobicyclo[1.1.1]pentanyl)-4-trifluoromethylpyridinium iodide (10e)

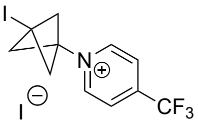

^{13}C NMR (126 MHz, DMSO-d_6)

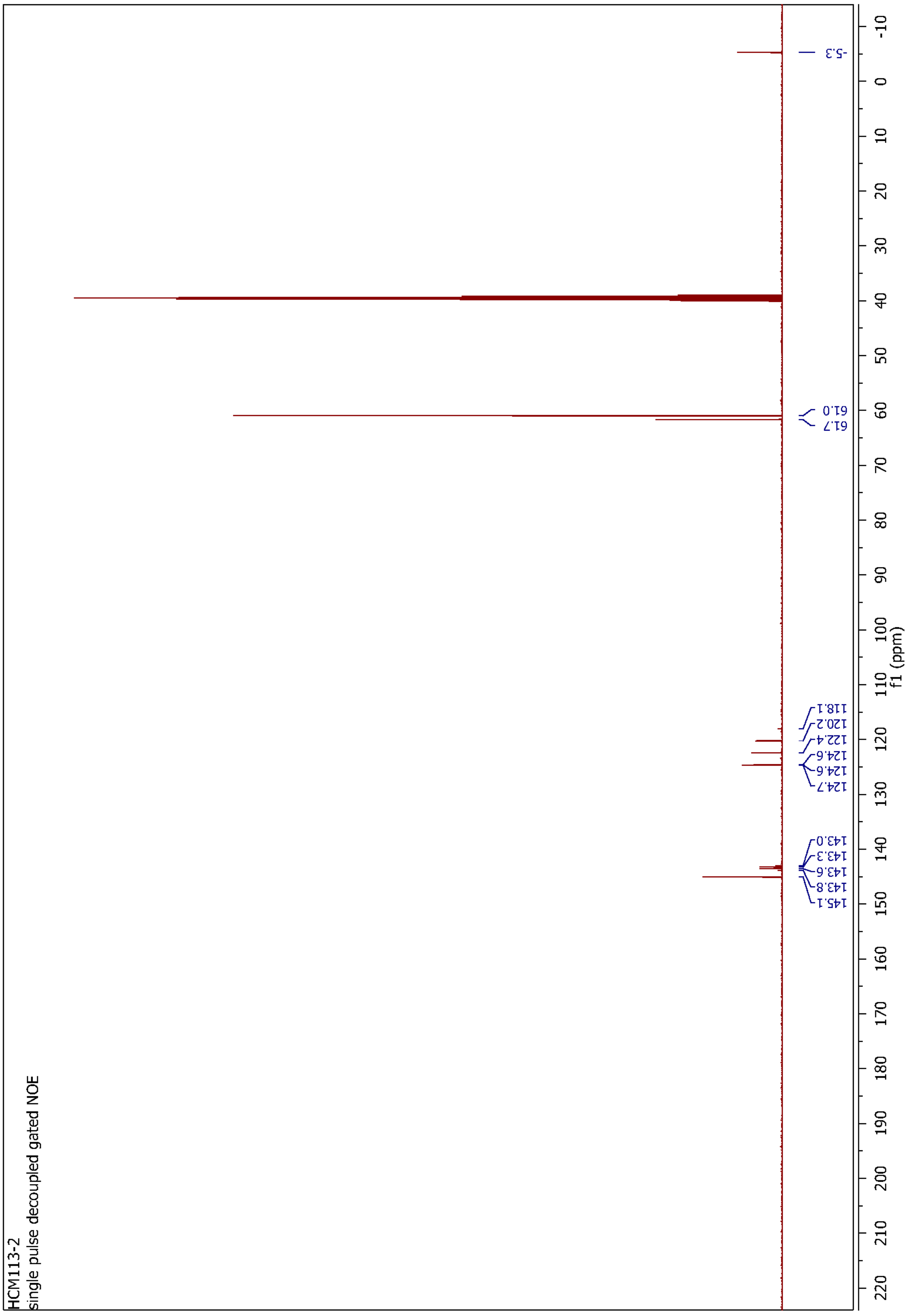

1-(3-Iodobicyclo[1.1.1]pentanyl)-4-trifluoromethylpyridinium iodide (10e)

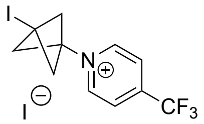

^{19}F NMR (500 MHz, DMSO-d_6)

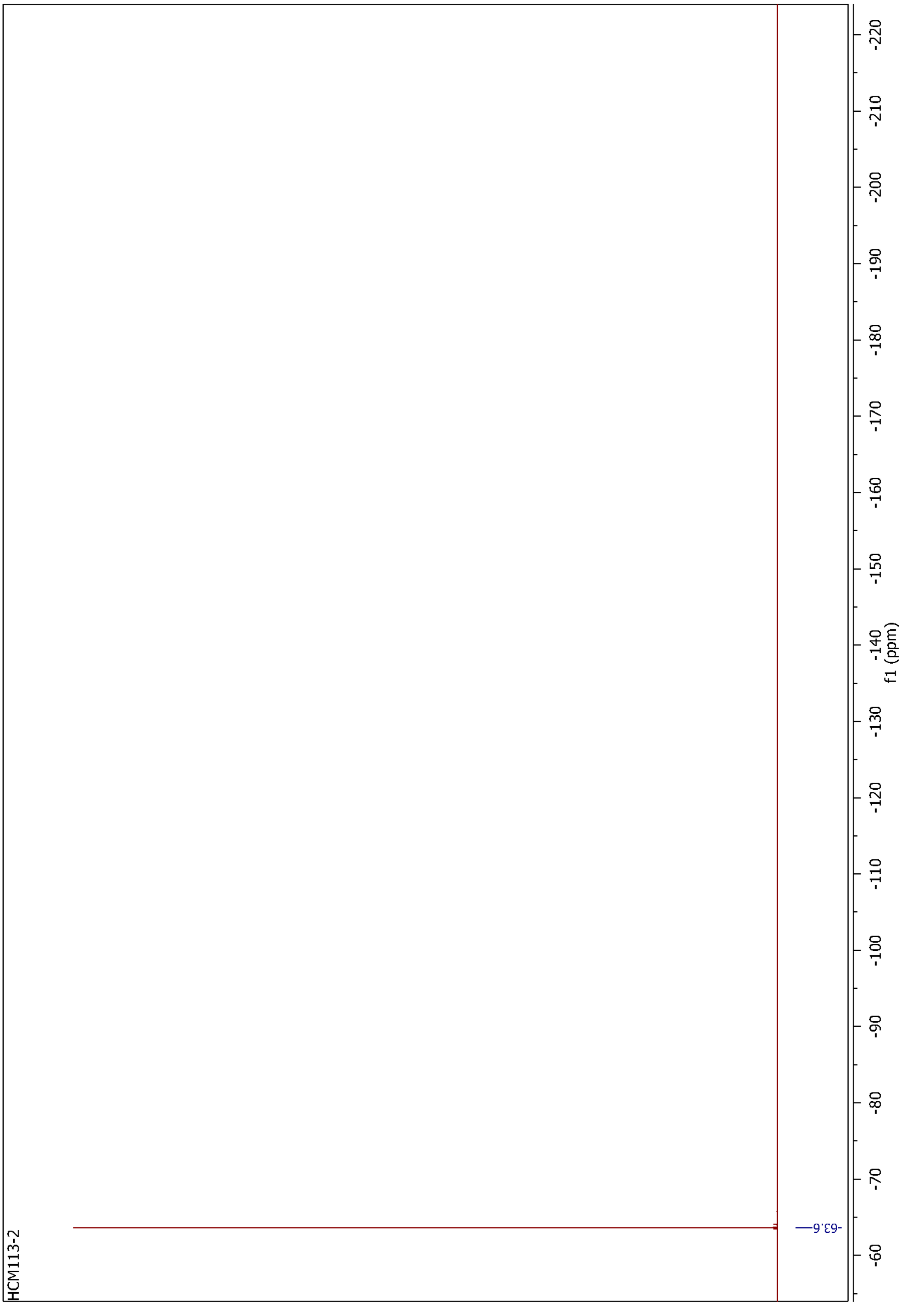

1-(3-Iodobicyclo[1.1.1]pentanyl)-4-methoxypyridinium iodide (10f)

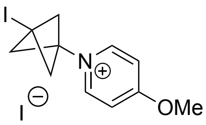

^{1}H NMR (500 MHz, DMSO-d_6)

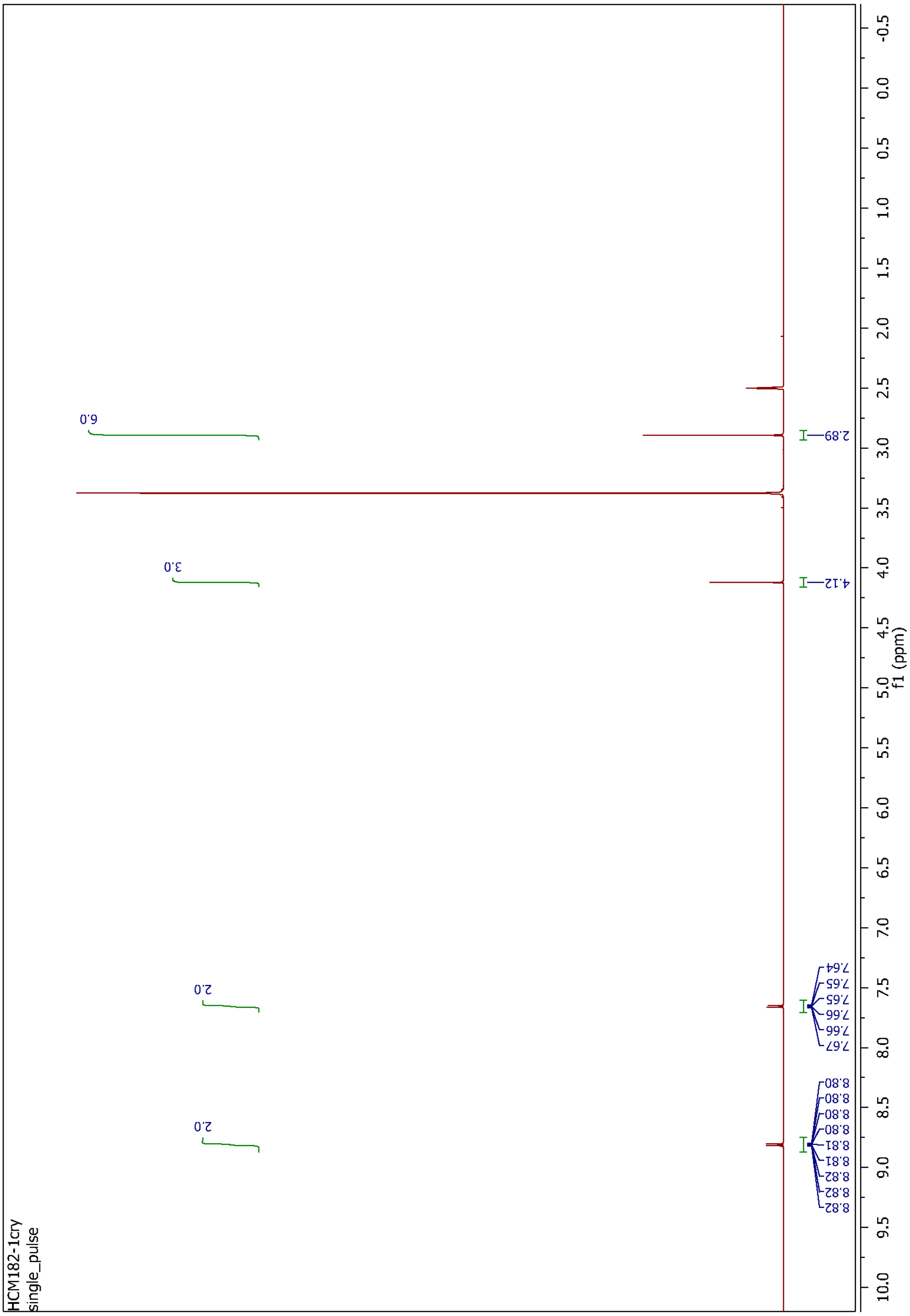

1-(3-Iodobicyclo[1.1.1]pentanyl)-4-methoxypyridinium iodide (10f)

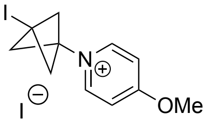

^{13}C NMR (101 MHz, DMSO-d_6)

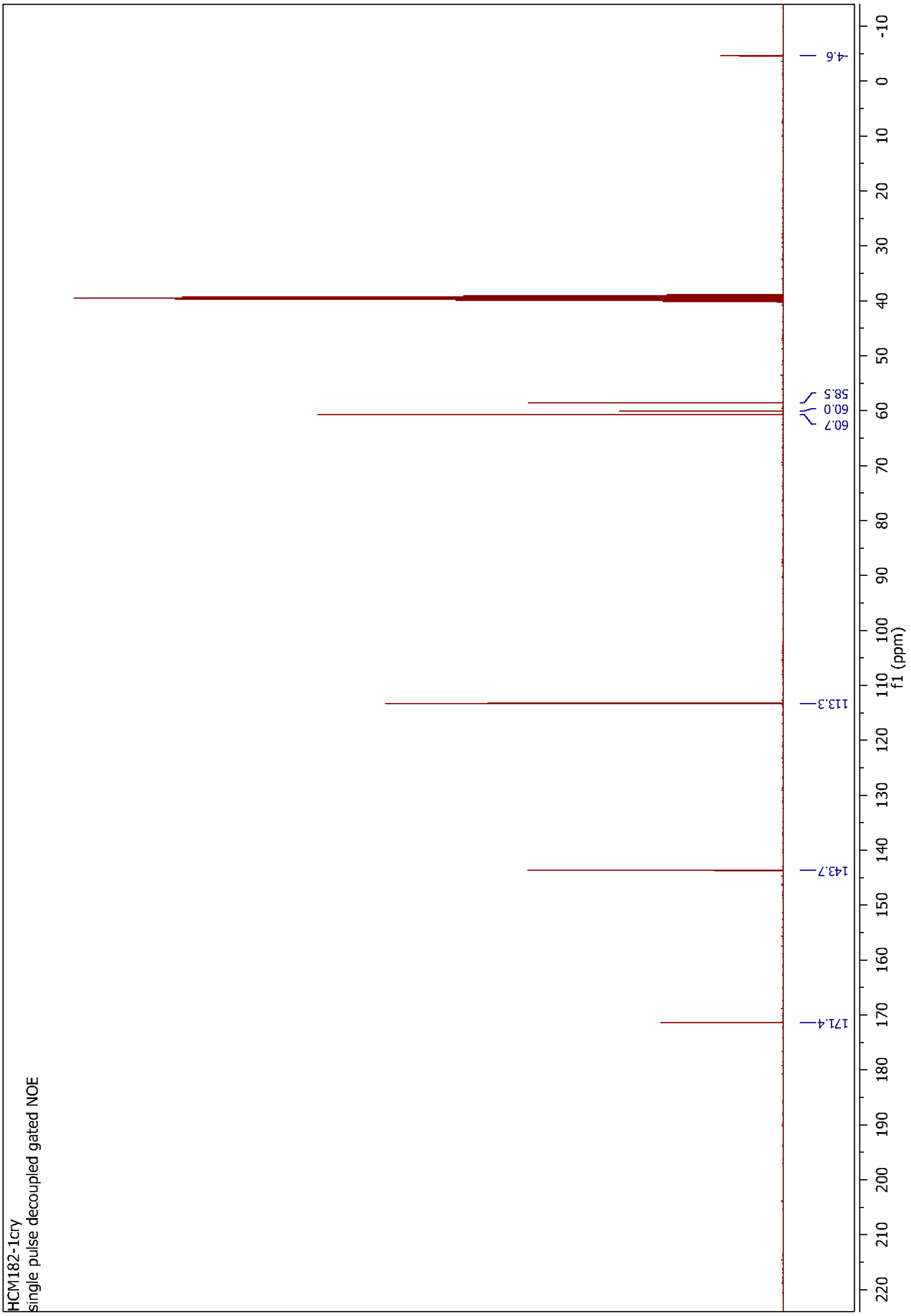

1-(3-Iodobicyclo[1.1.1]pentanyl)-4-phenylpyridinium iodide (10g)

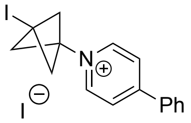

^{1}H NMR (500 MHz, DMSO-d_6)

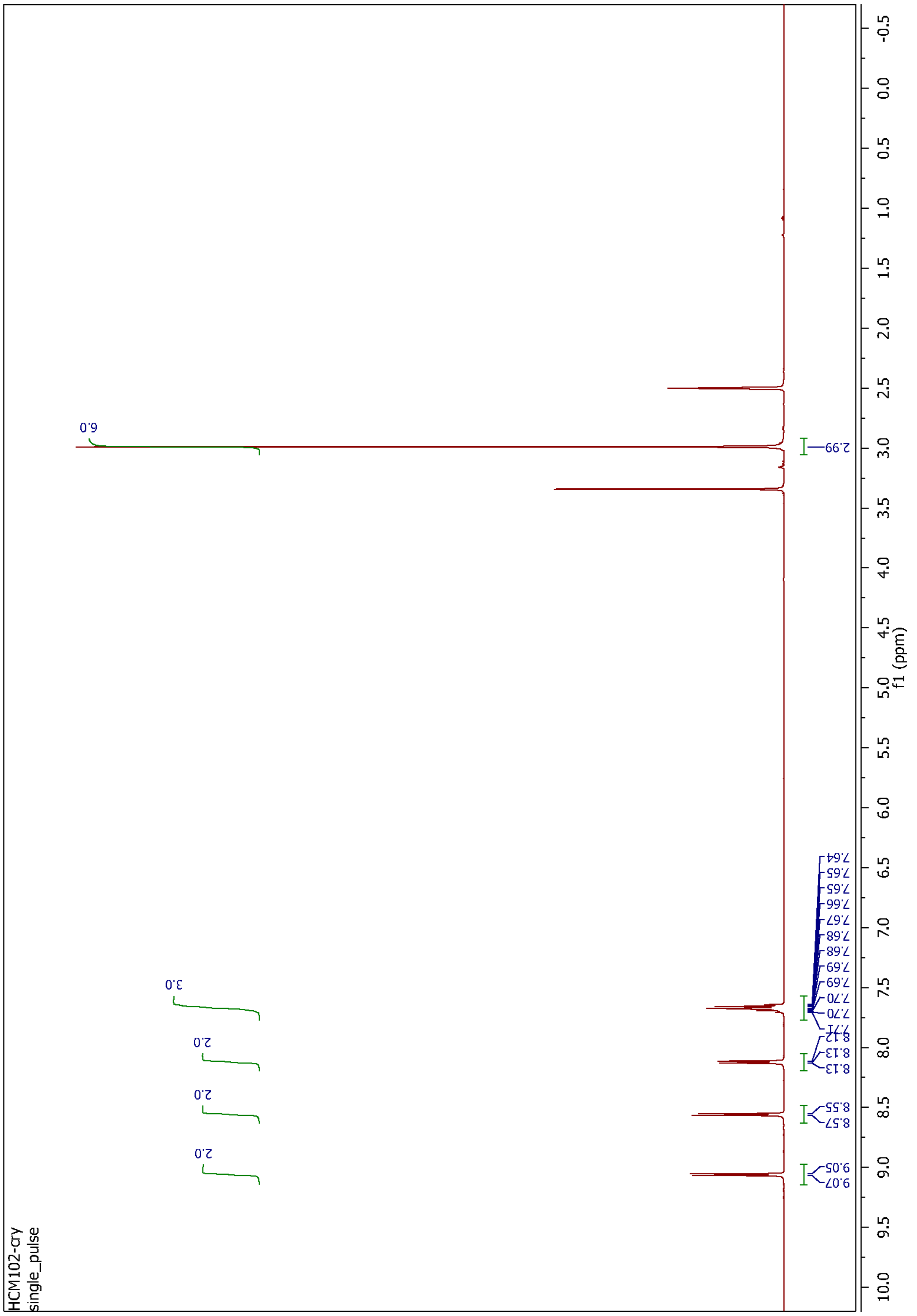

1-(3-Iodobicyclo[1.1.1]pentanyl)-4-phenylpyridinium iodide (10g)

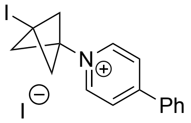

^{13}C NMR (126 MHz, DMSO-d_6)

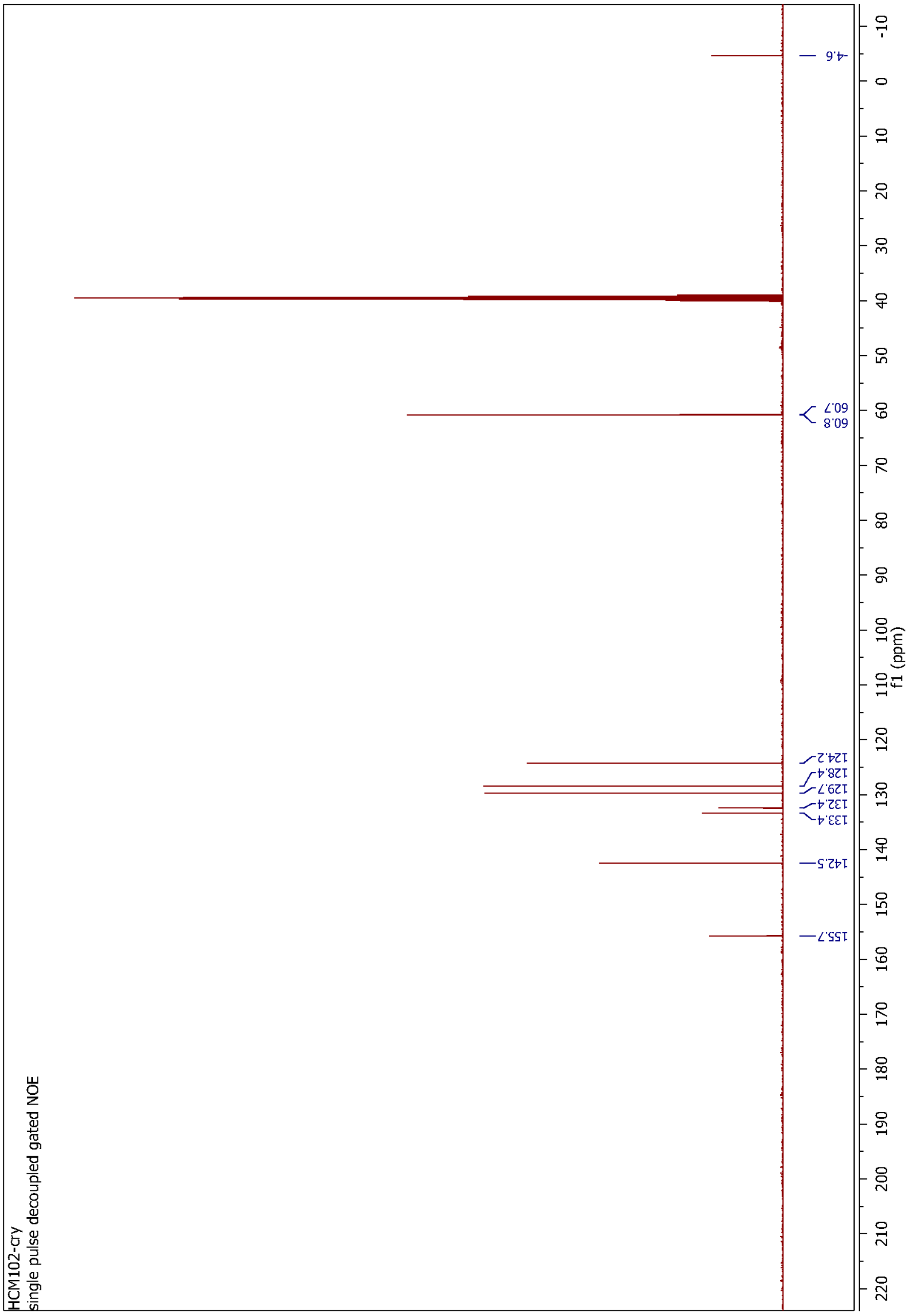

1-(3-Iodobicyclo[1.1.1]pentanyl)-3-methylpyridinium iodide (10h)

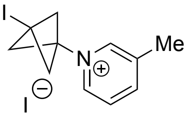

^{1}H NMR (500 MHz, DMSO-d_6)

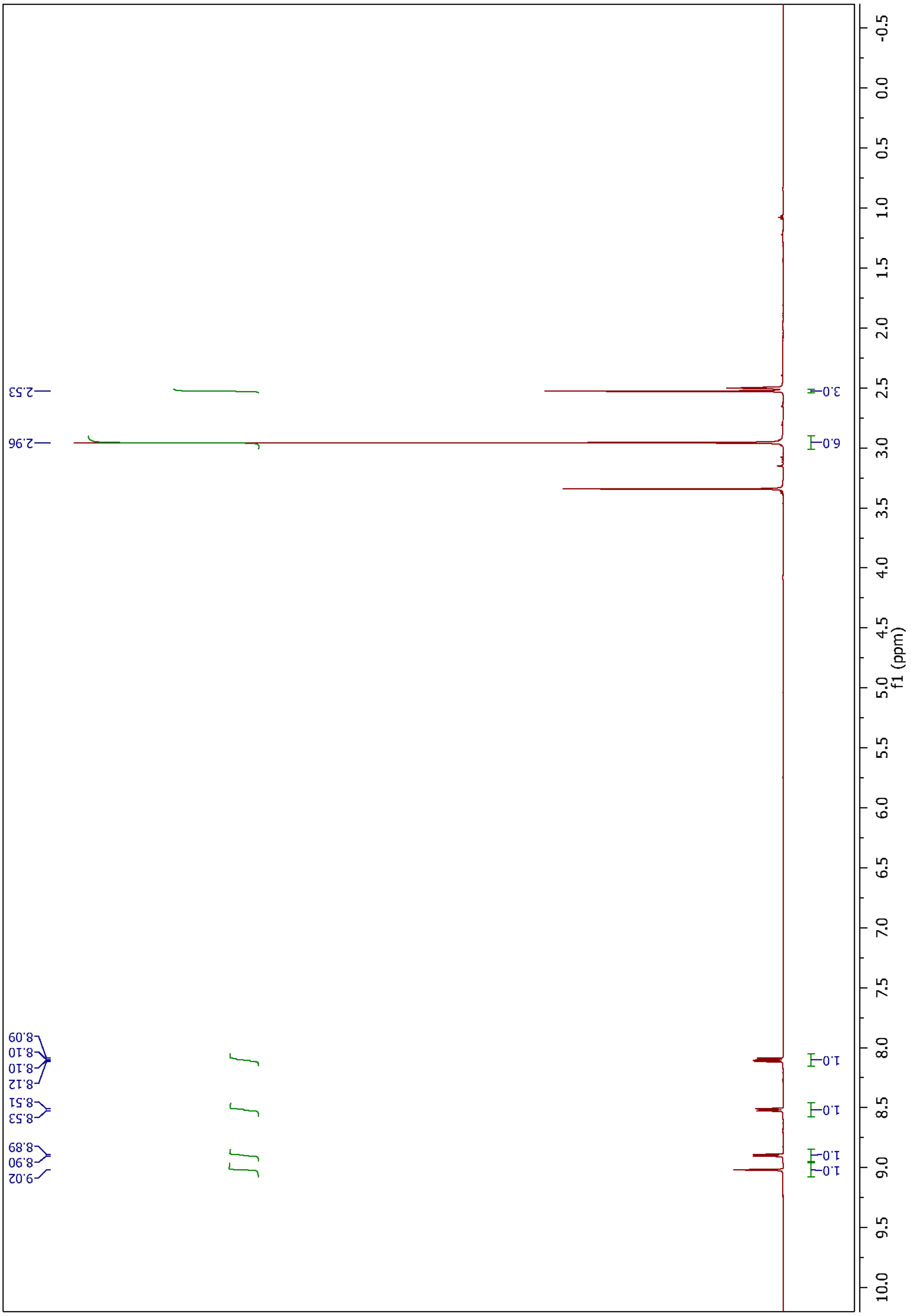

1-(3-Iodobicyclo[1.1.1]pentanyl)-3-methylpyridinium iodide (10h)

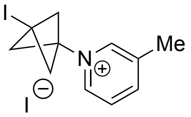

^{13}C NMR (126 MHz, DMSO-d_6)

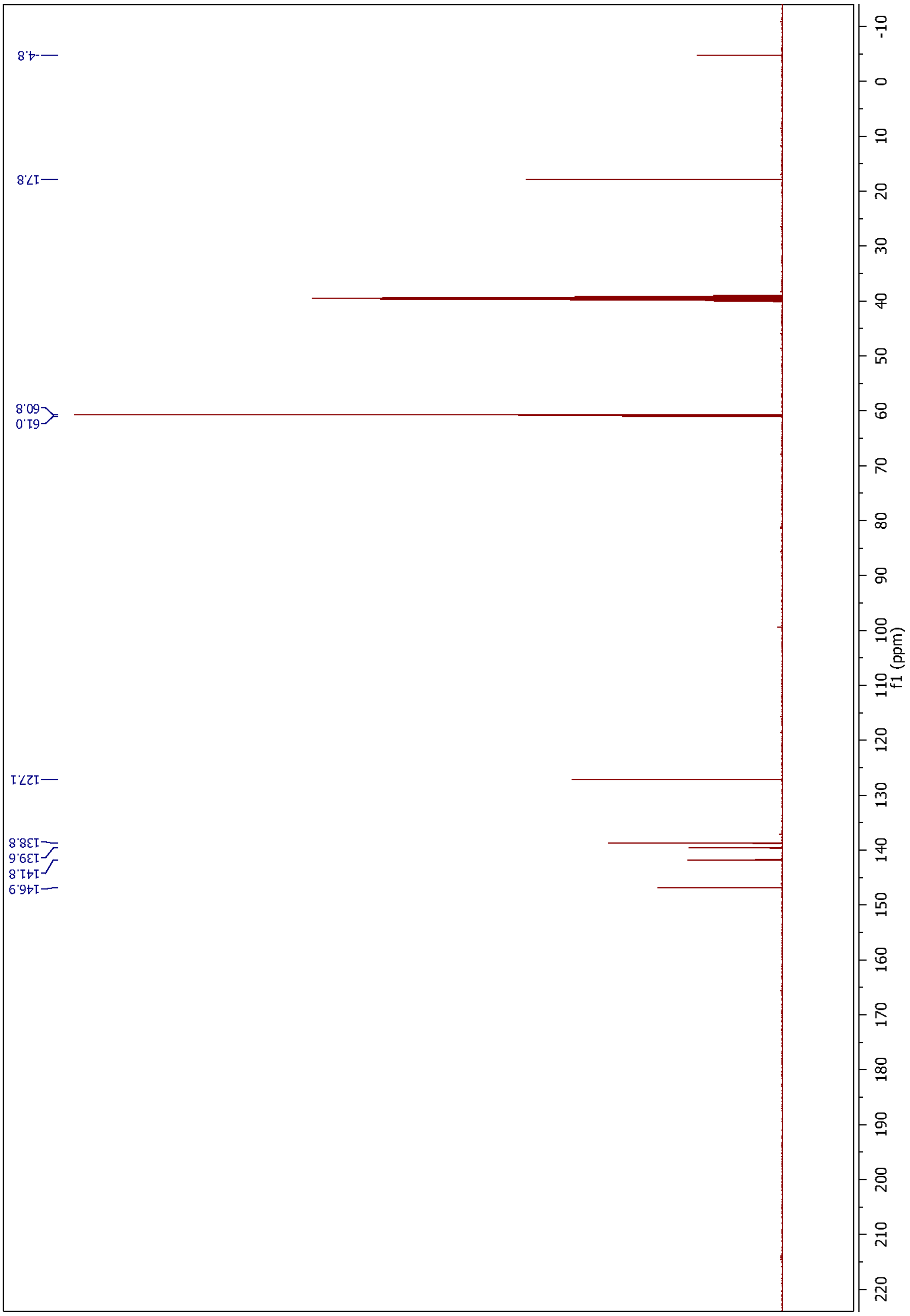

1-(3-Iodobicyclo[1.1.1]pentanyl)-3-chloropyridinium iodide (10i)

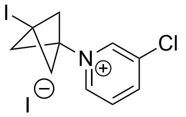

^{1}H NMR (500 MHz, DMSO-d_6)

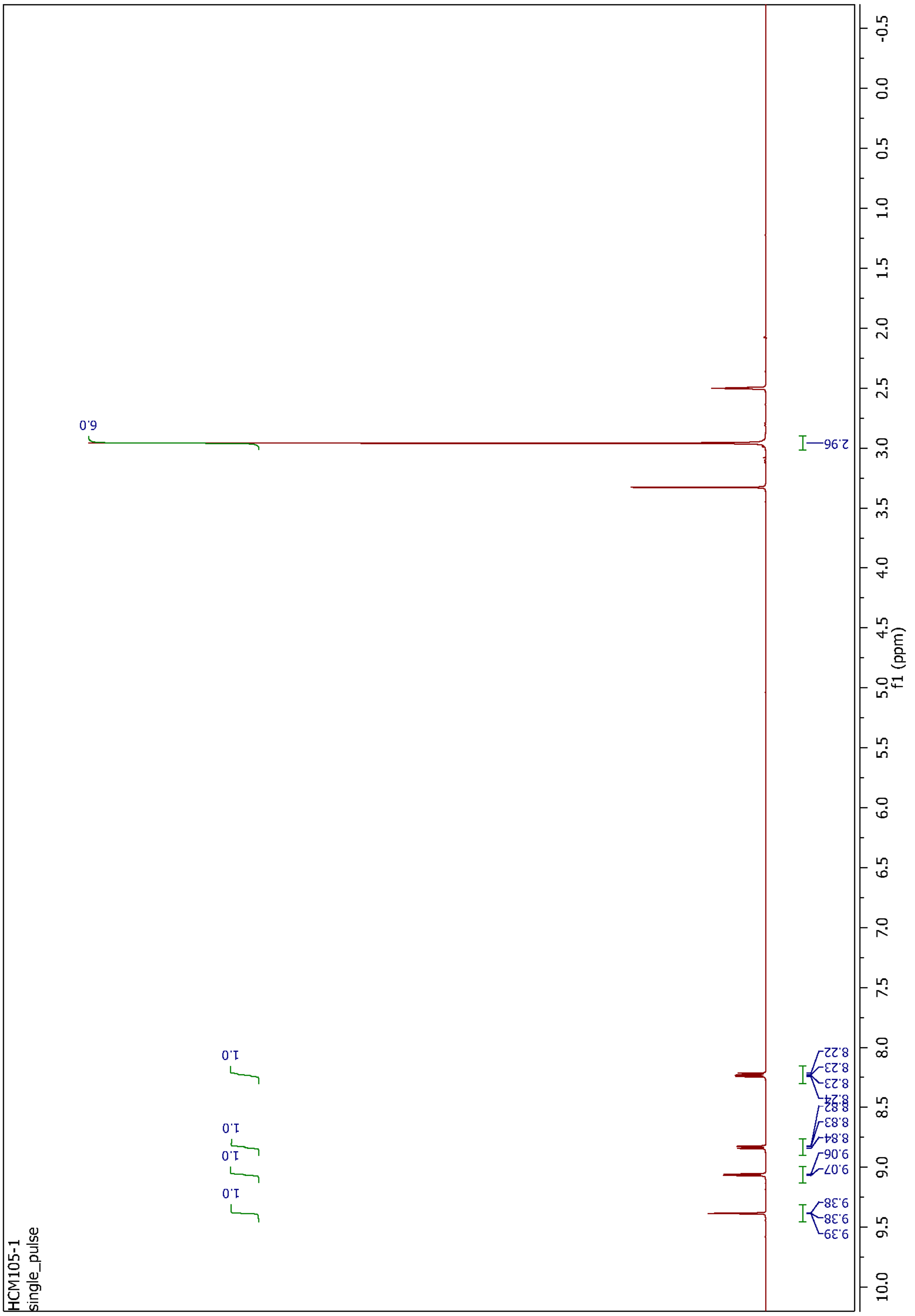

1-(3-Iodobicyclo[1.1.1]pentanyl)-3-chloropyridinium iodide (10i)

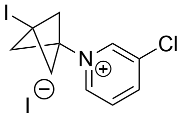

^{13}C NMR (126 MHz, DMSO-d_6)

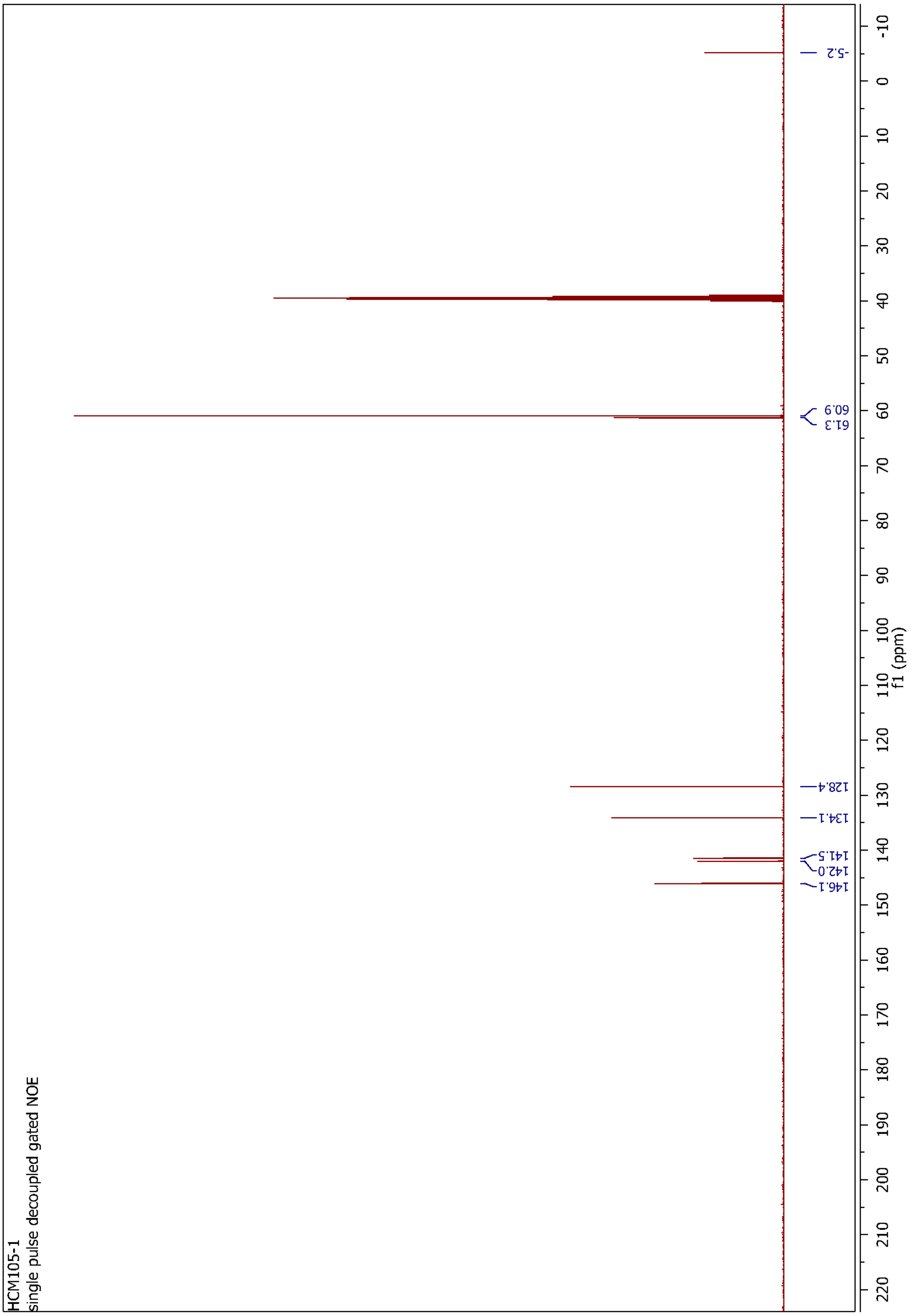

1-(3-Iodobicyclo[1.1.1]pentanyl)-3-bromopyridinium iodide (10j)

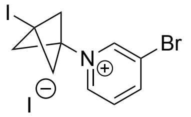

^{1}H NMR (500 MHz, DMSO-d_6)

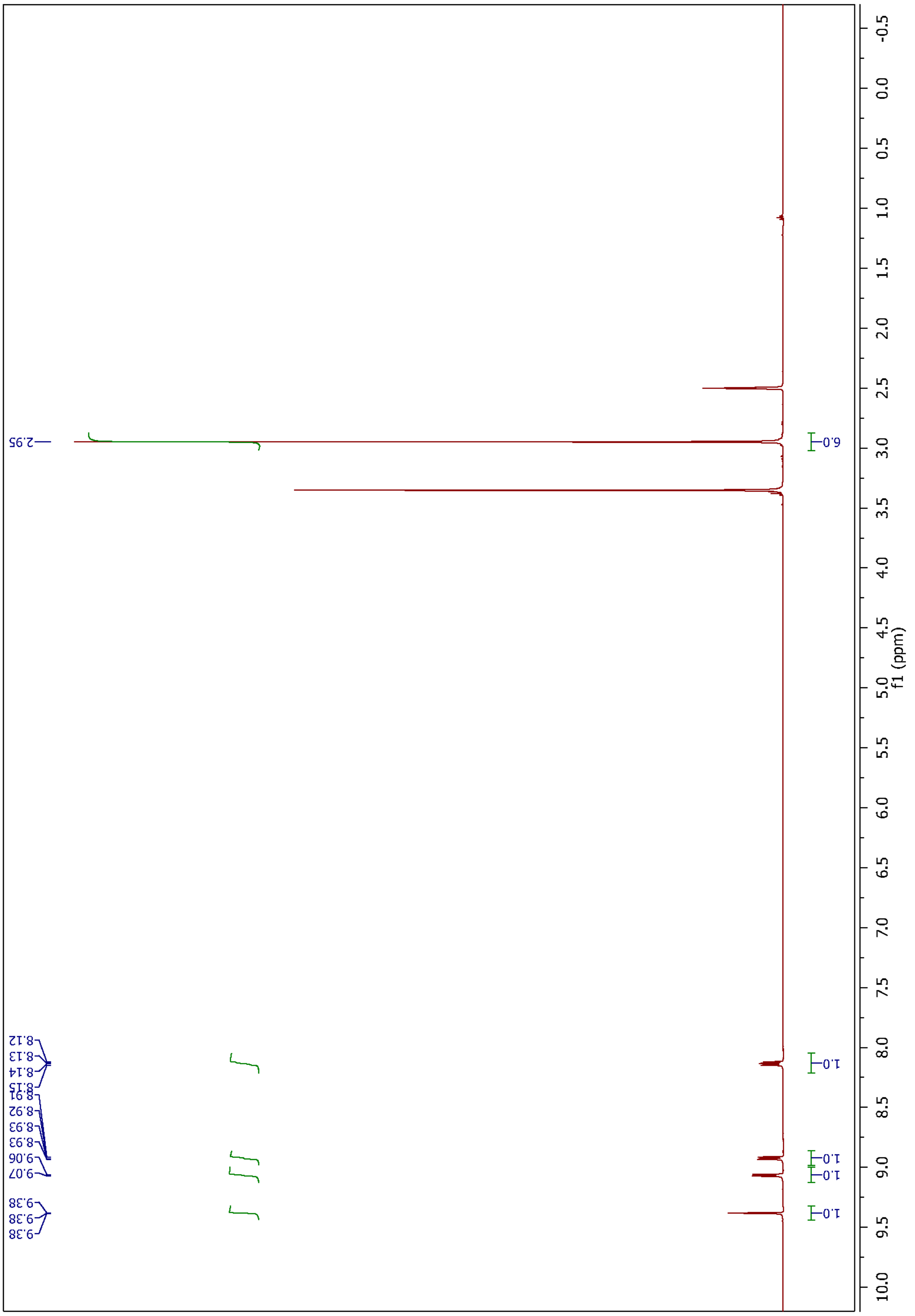

1-(3-Iodobicyclo[1.1.1]pentanyl)-3-bromopyridinium iodide (10j)

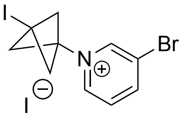

^{13}C NMR (126 MHz, DMSO-d_6)

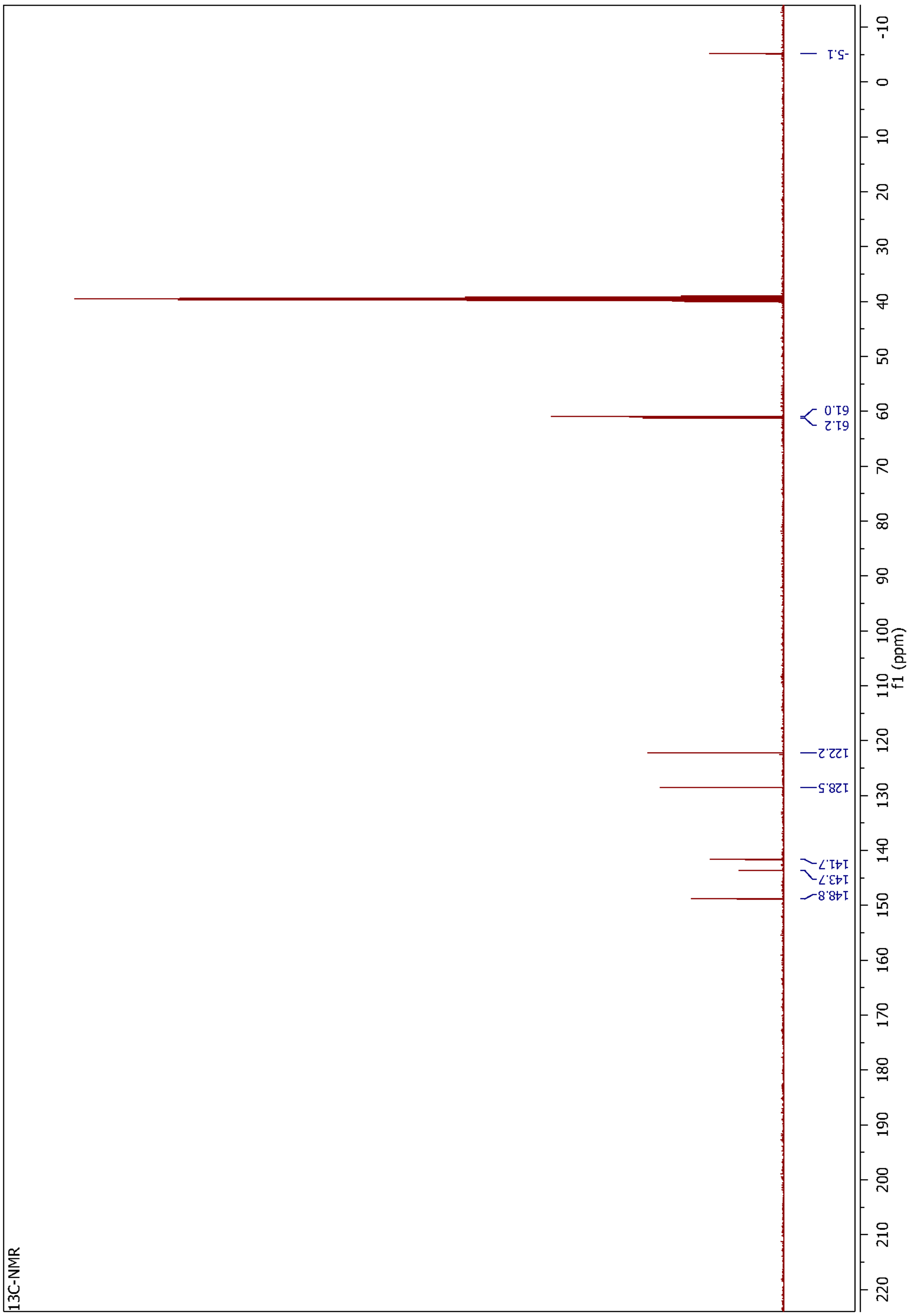

1-(3-Iodobicyclo[1.1.1]pentanyl)-3-fluoropyridinium iodide (10k)

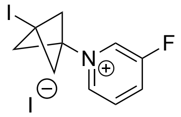

^{1}H NMR (500 MHz, DMSO-d_6)

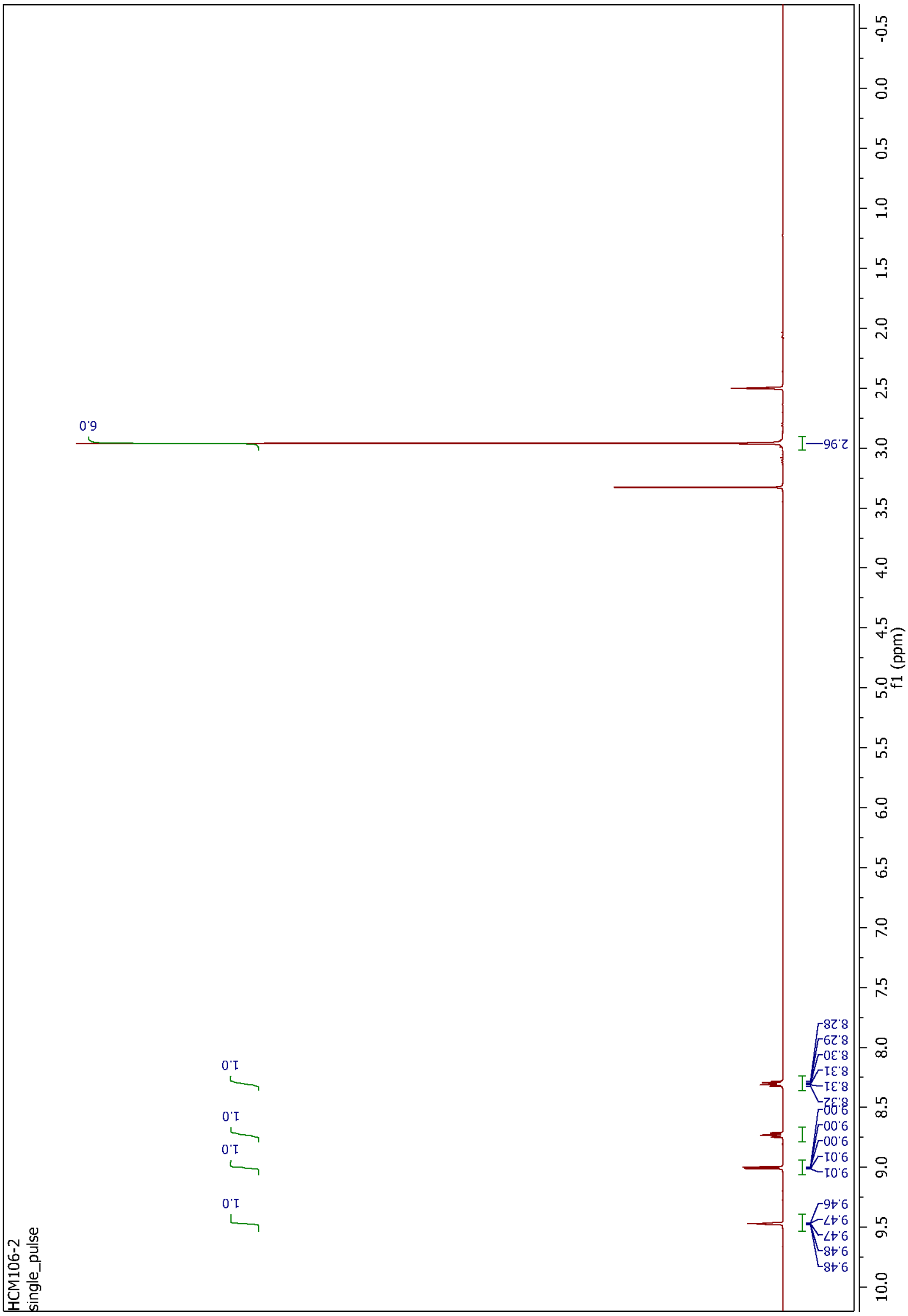

1-(3-Iodobicyclo[1.1.1]pentanyl)-3-fluoropyridinium iodide (10k)

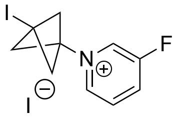

^{13}C NMR (126 MHz, DMSO-d_6)

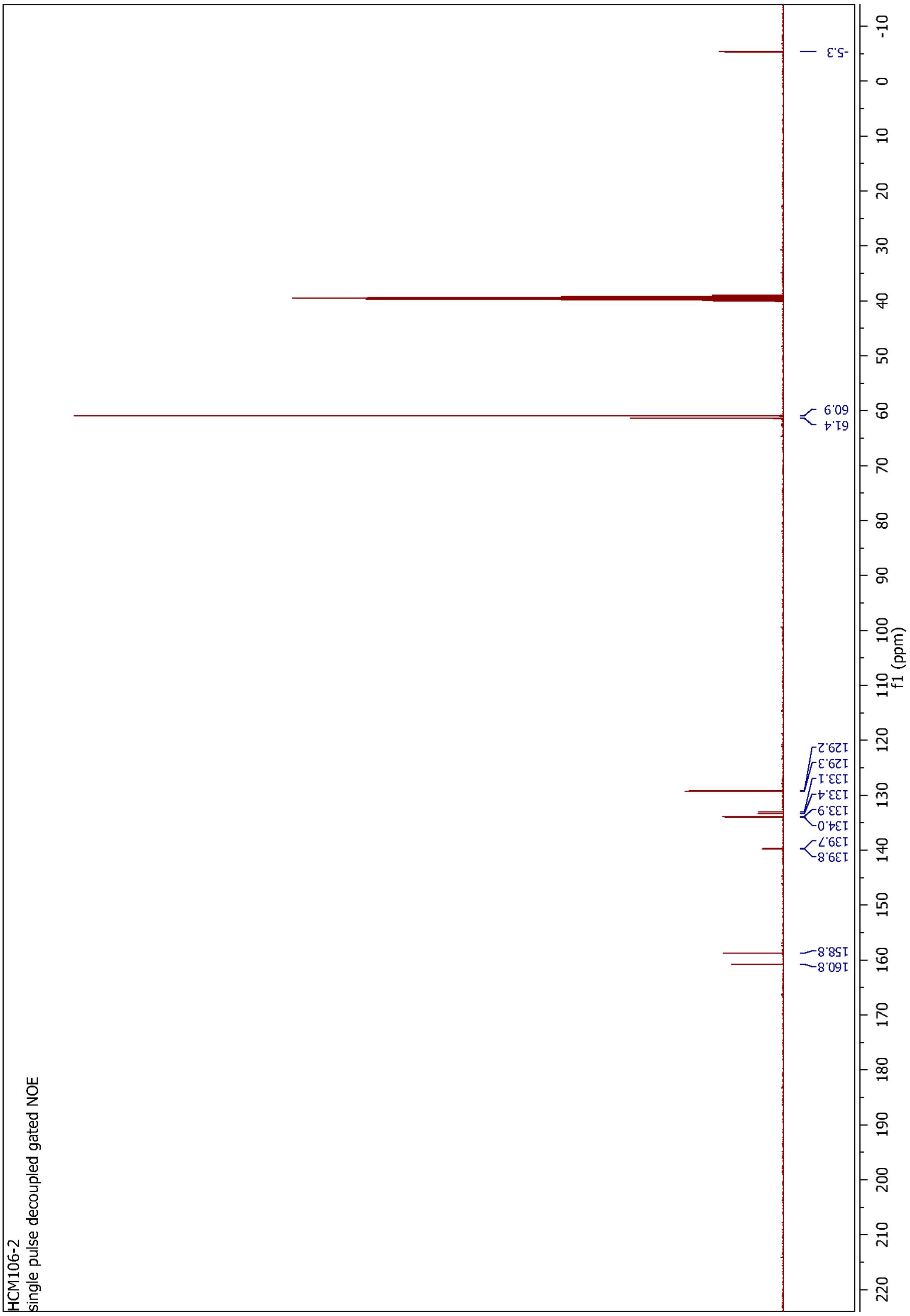

1-(3-Iodobicyclo[1.1.1]pentanyl)-3-fluoropyridinium iodide (10k)

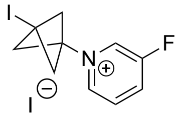

^{19}F NMR (500 MHz, DMSO-d_6)

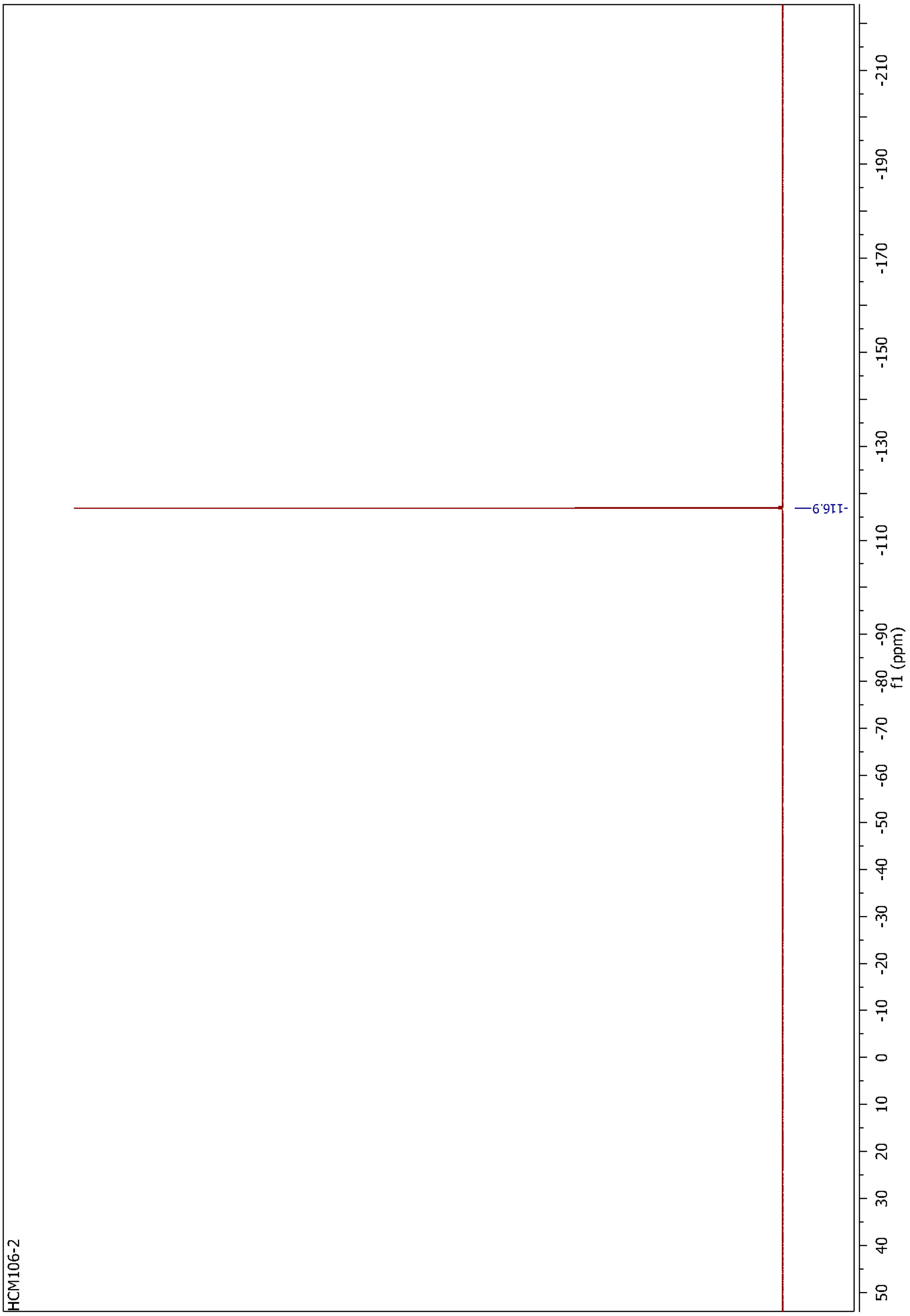

1-(3-Iodobicyclo[1.1.1]pentanyl)-3-formylpyridinium iodide (10l)

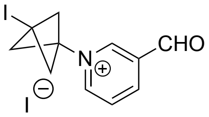

^{1}H NMR (500 MHz, DMSO-d_6)

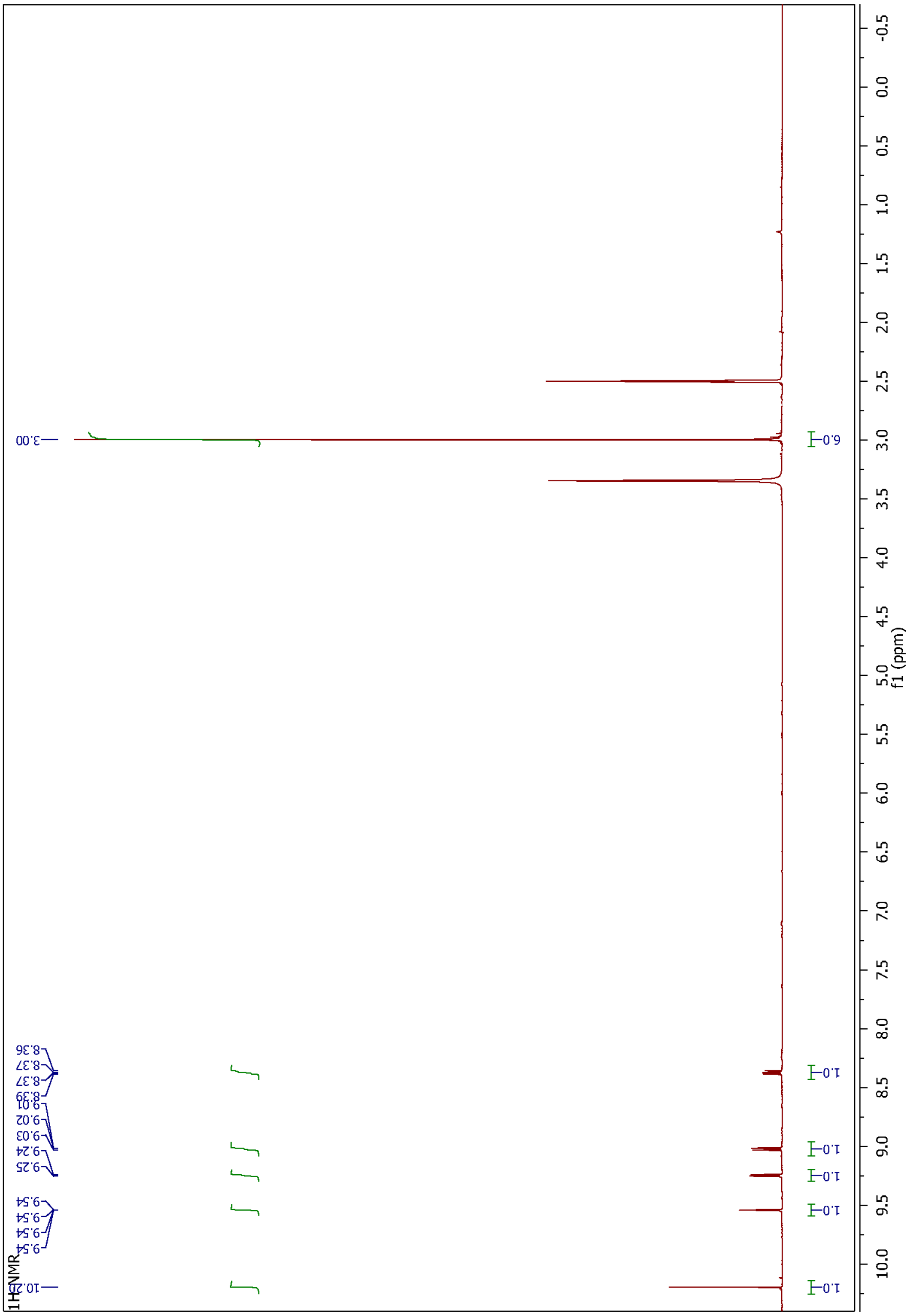

1-(3-Iodobicyclo[1.1.1]pentanyl)-3-formylpyridinium iodide (10l)

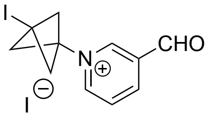

^{13}C NMR (126 MHz, DMSO-d_6)

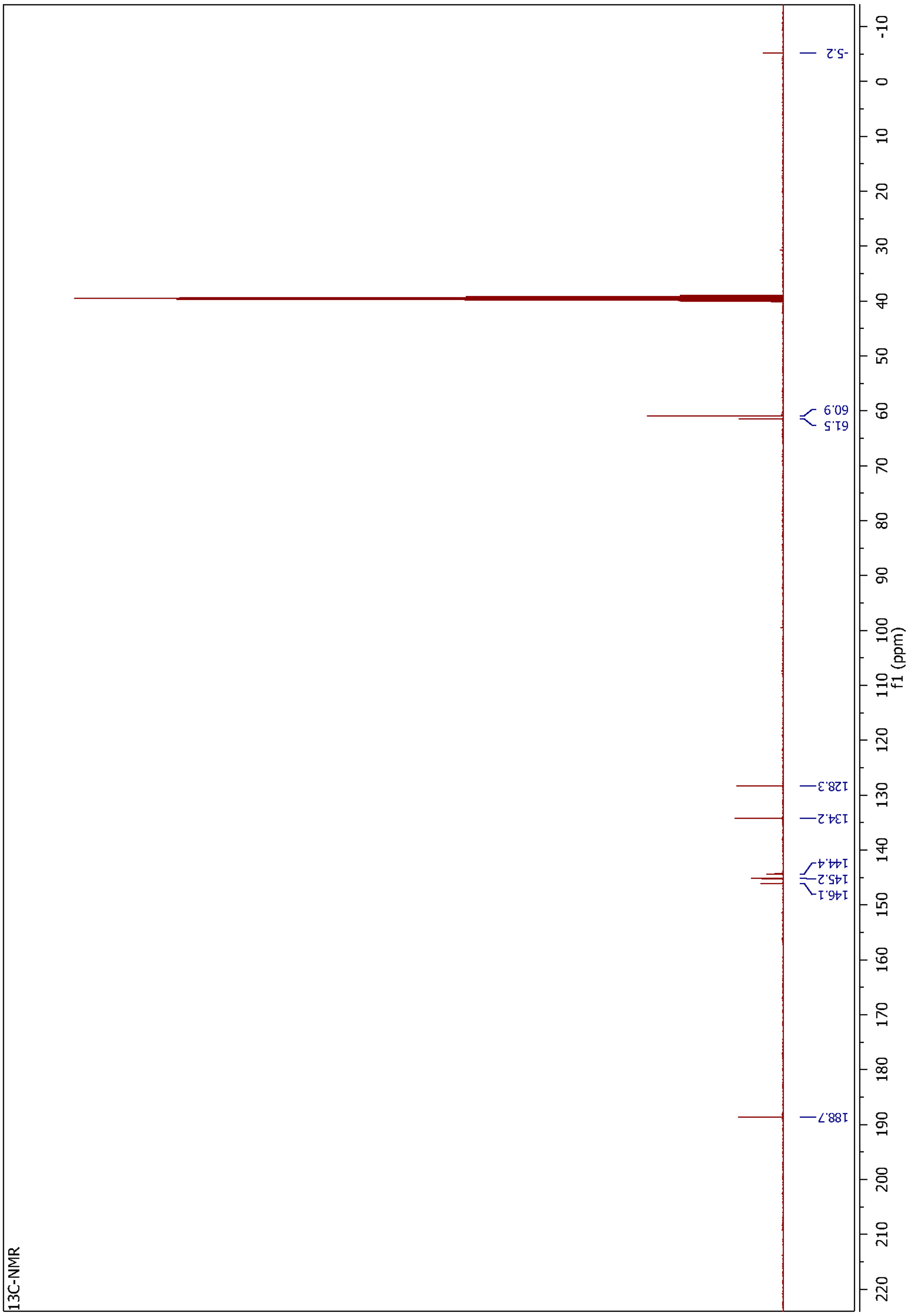

1-(3-Iodobicyclo[1.1.1]pentanyl)-3-acetylpyridinium iodide (10m)

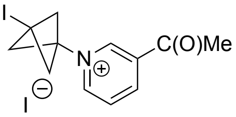

^{1}H NMR (500 MHz, DMSO-d_6)

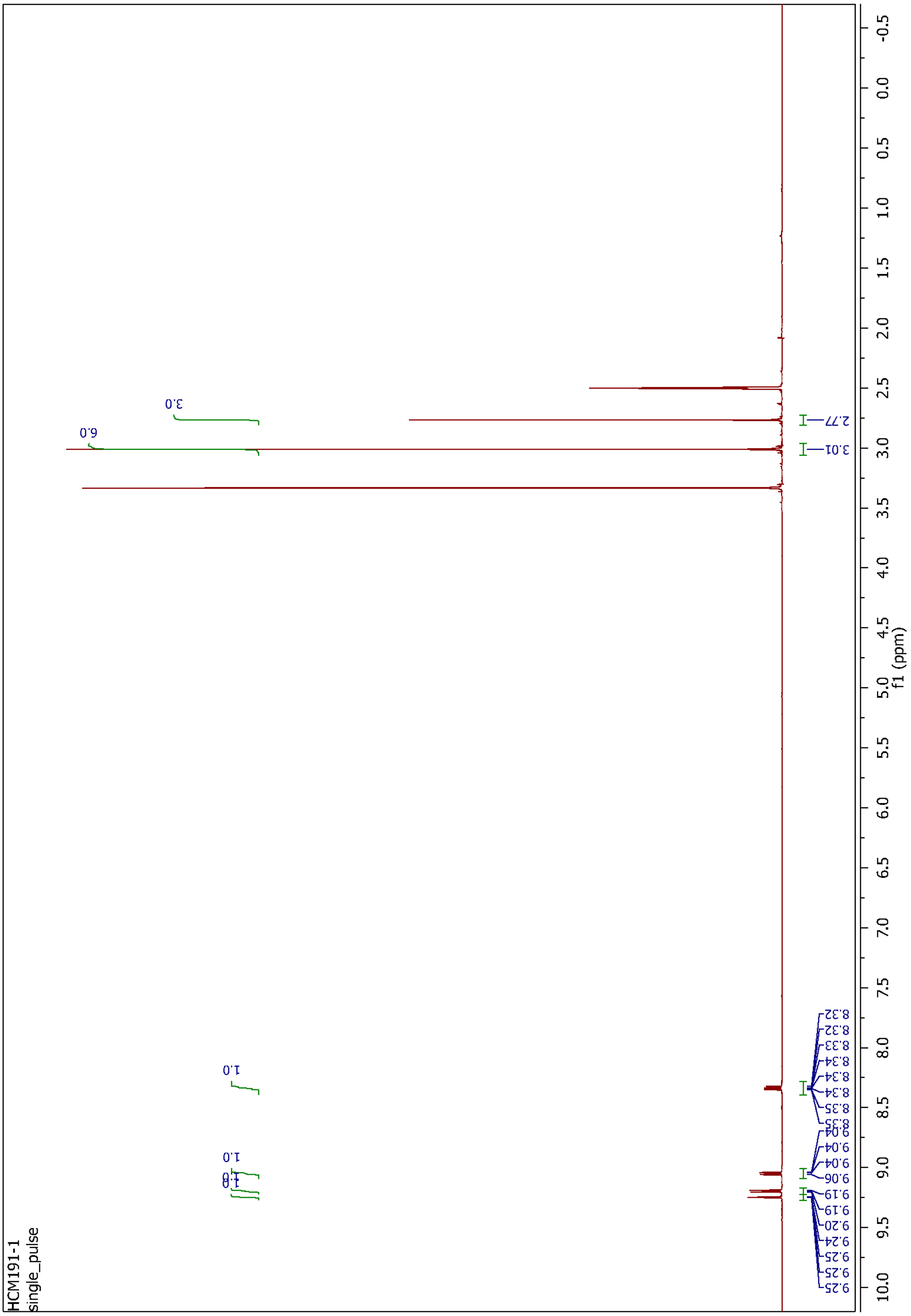

1-(3-Iodobicyclo[1.1.1]pentanyl)-3-acetylpyridinium iodide (10m)

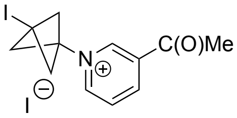

^{13}C NMR (126 MHz, DMSO-d_6)

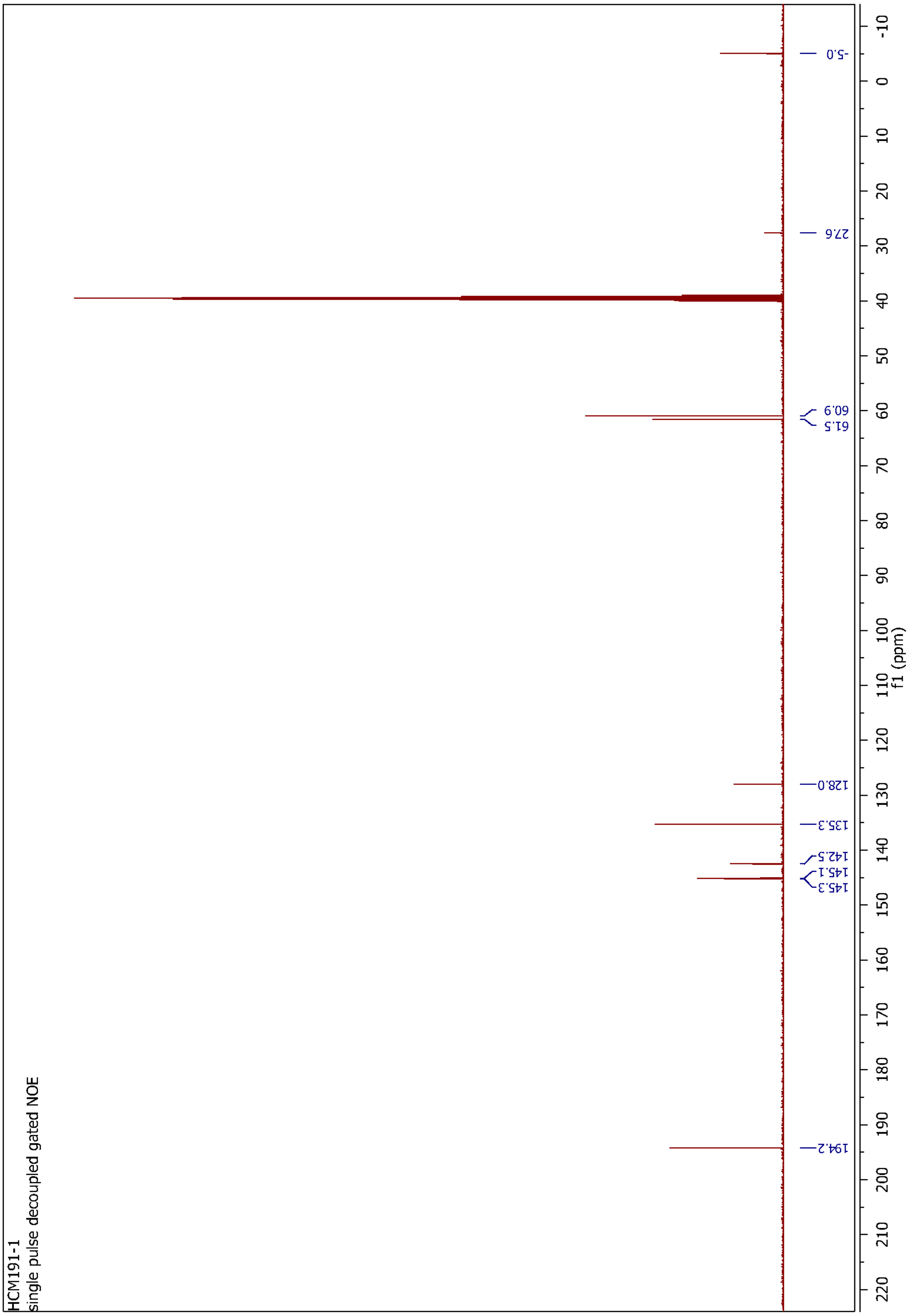

1-(3-Iodobicyclo[1.1.1]pentanyl)-3-ethynylpyridinium iodide (10n)

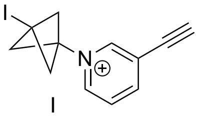

^{1}H NMR (400 MHz, DMSO-d_6)

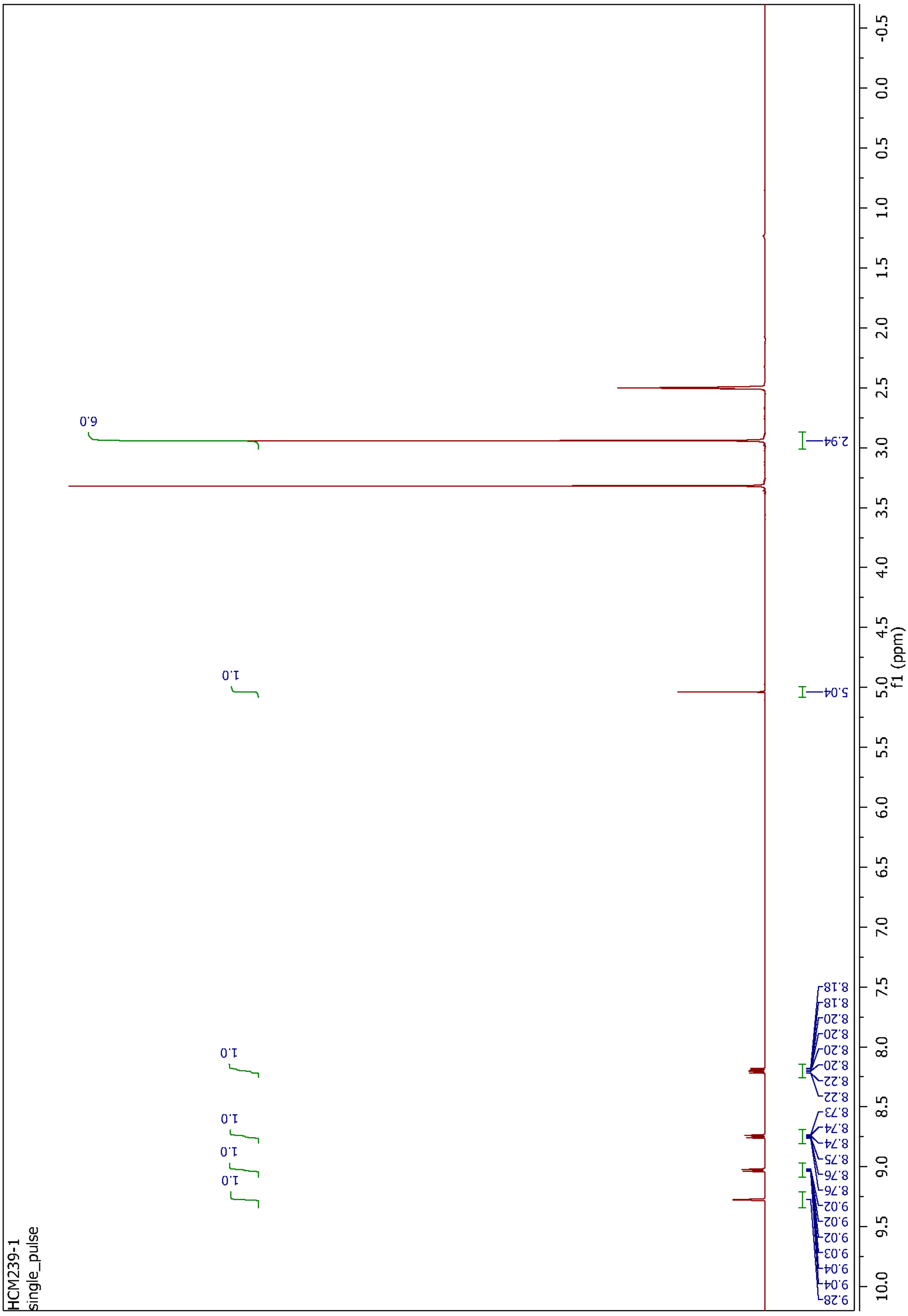

1-(3-Iodobicyclo[1.1.1]pentanyl)-3-ethynylpyridinium iodide (10n)

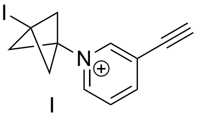

^{13}C NMR (101 MHz, DMSO-d_6)

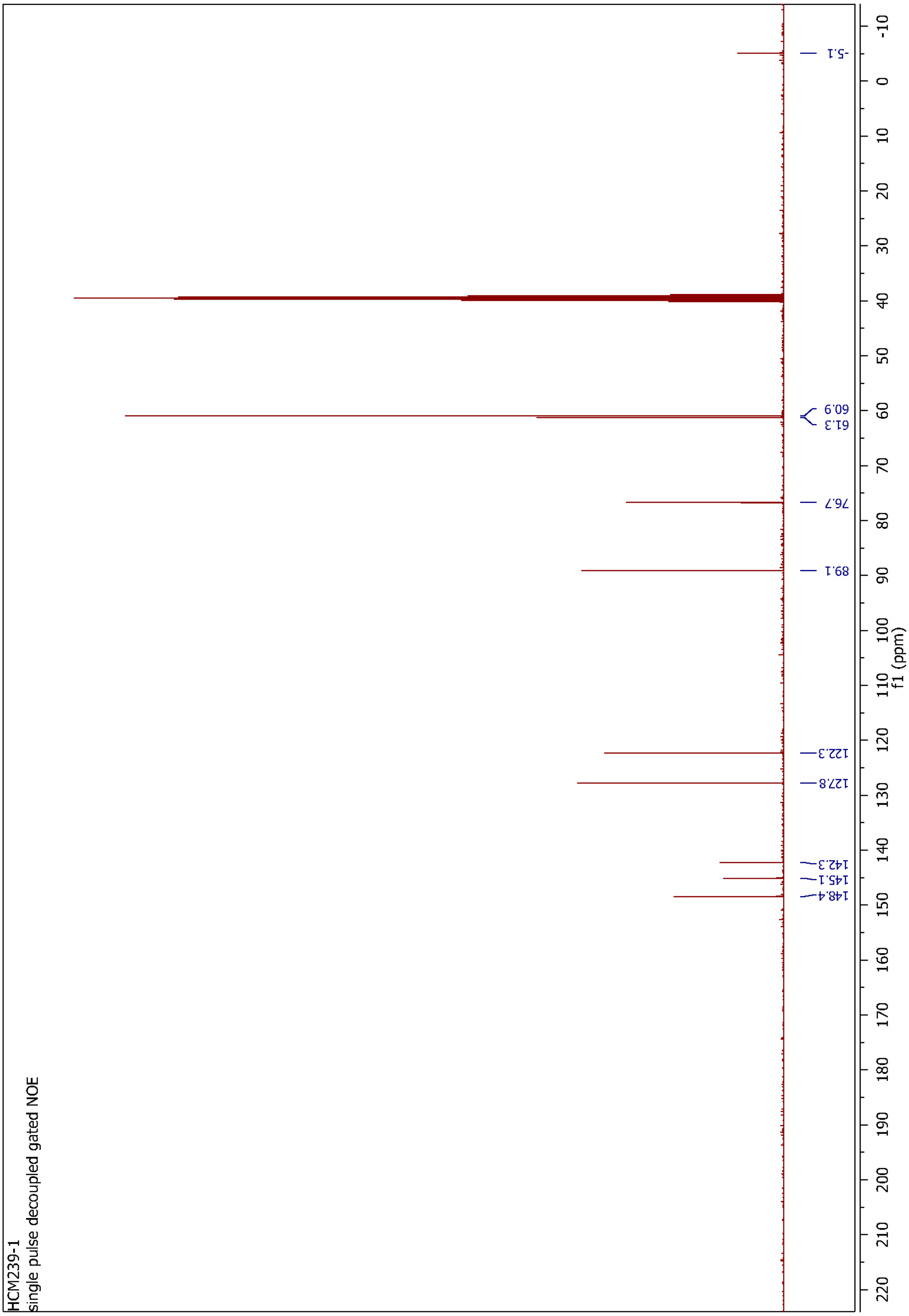

1-(3-Iodobicyclo[1.1.1]pentanyl)-2-methylpyridinium iodide (10o)

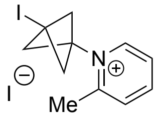

^{1}H NMR (500 MHz, DMSO-d_6)

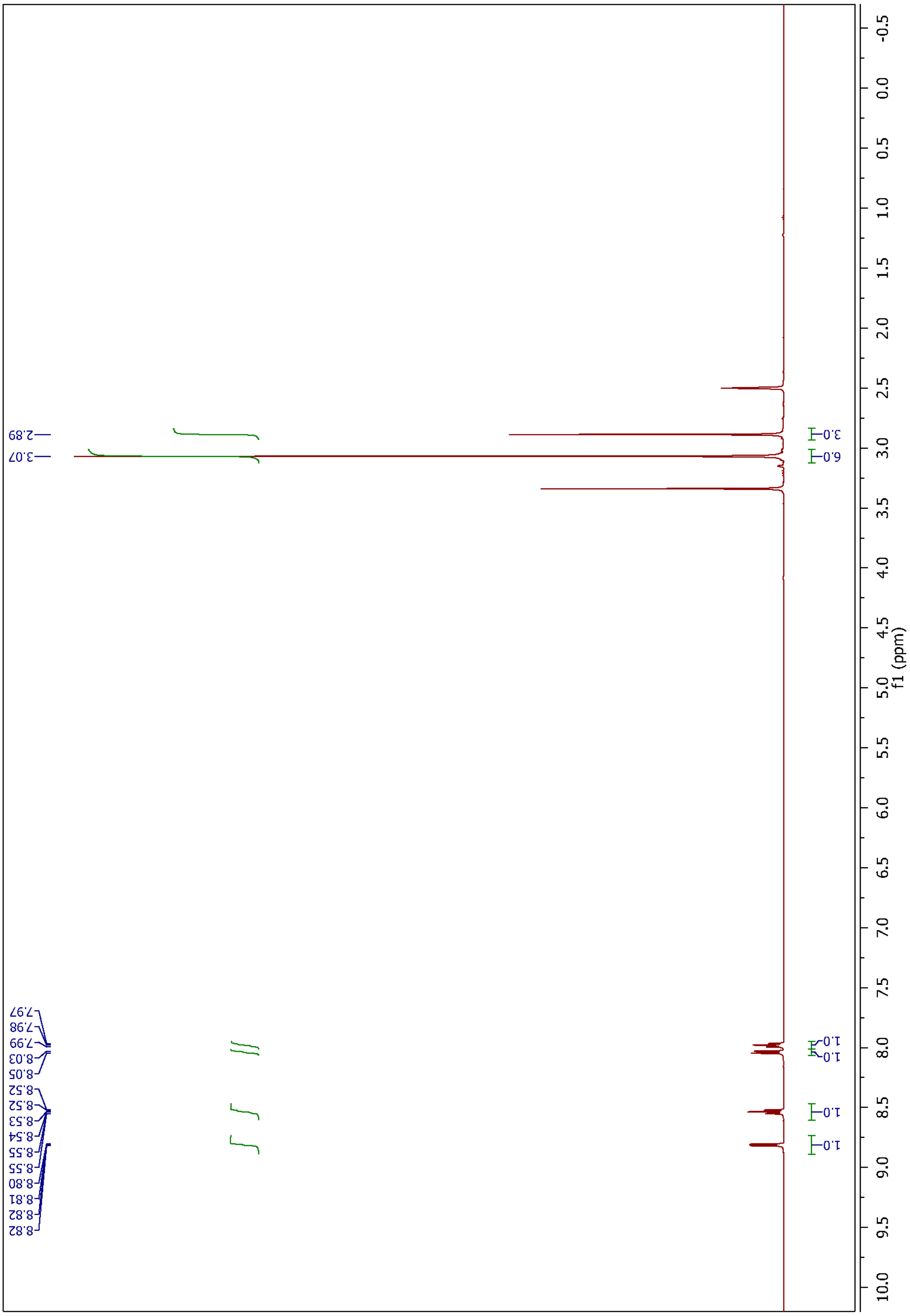

1-(3-Iodobicyclo[1.1.1]pentanyl)-2-methylpyridinium iodide (10o)

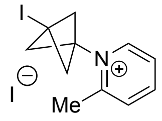

^{13}C NMR (101 MHz, DMSO-d_6)

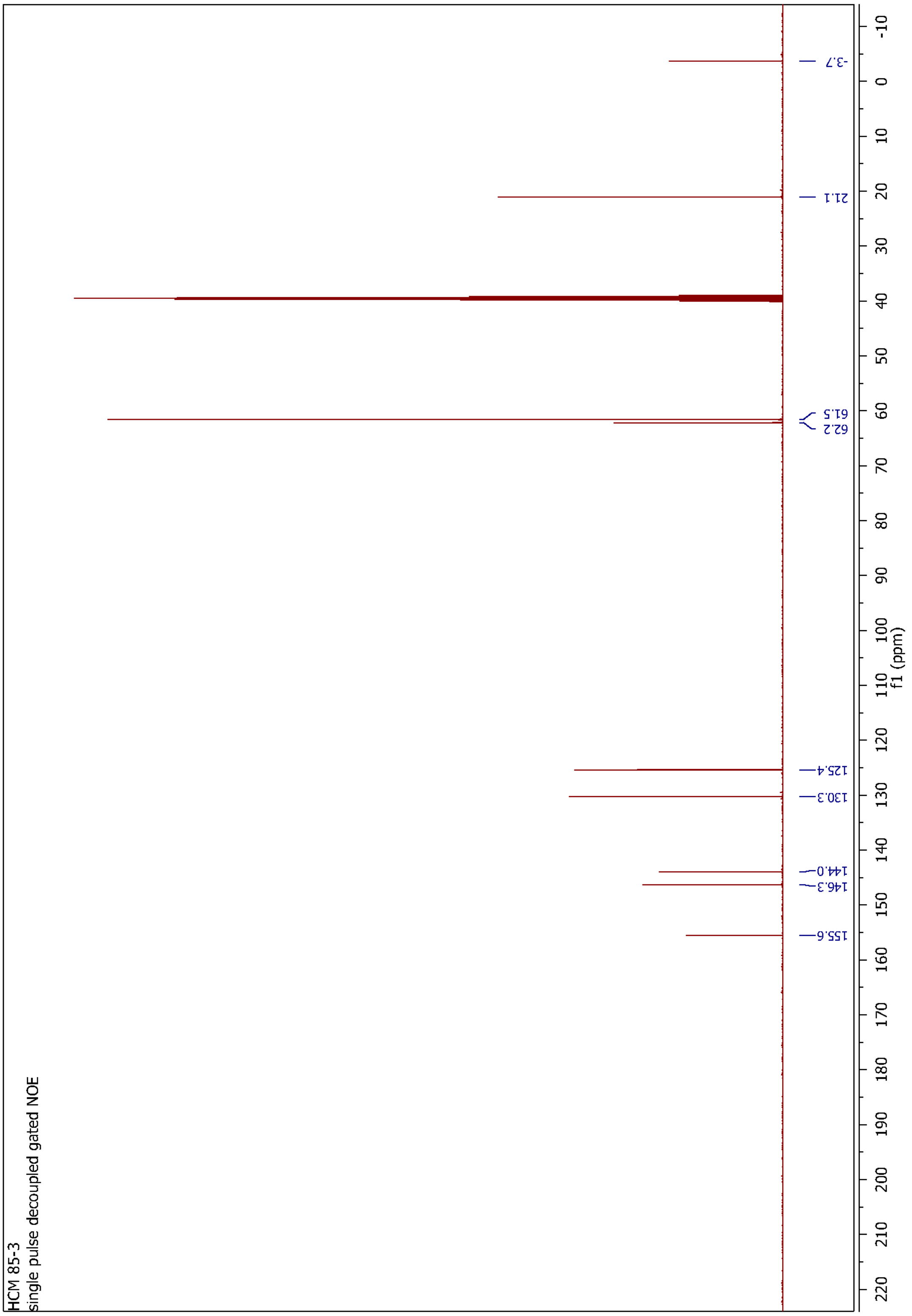

1-(3-Iodobicyclo[1.1.1]pentanyl)-3,5-dimethylpyridinium iodide (10p)

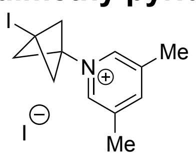

^{1}H NMR (500 MHz, DMSO-d_6)

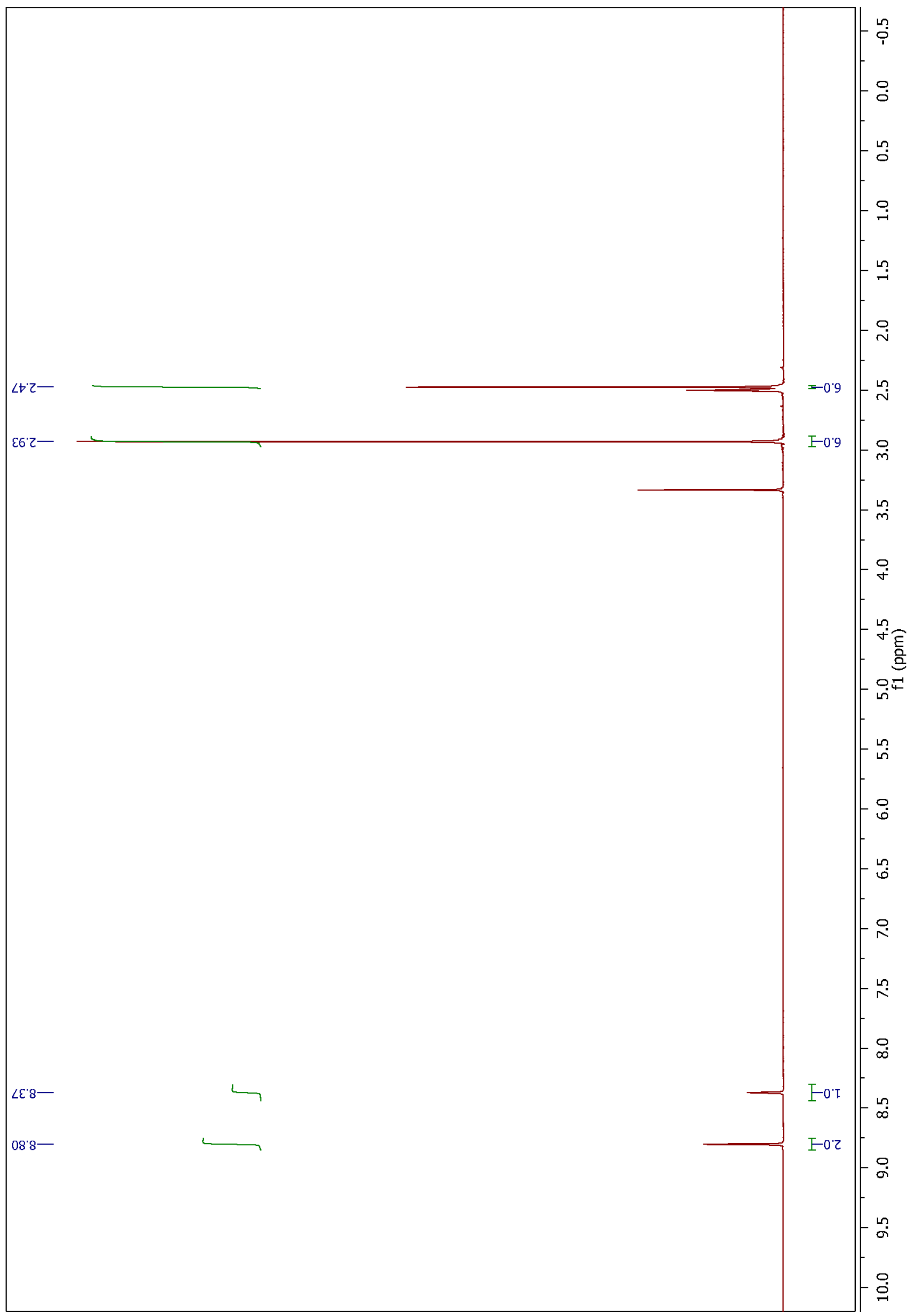

1-(3-Iodobicyclo[1.1.1]pentanyl)-3,5-dimethylpyridinium iodide (10p)

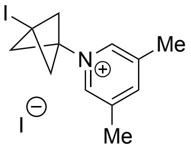

^{13}C NMR (126 MHz, DMSO-d_6)

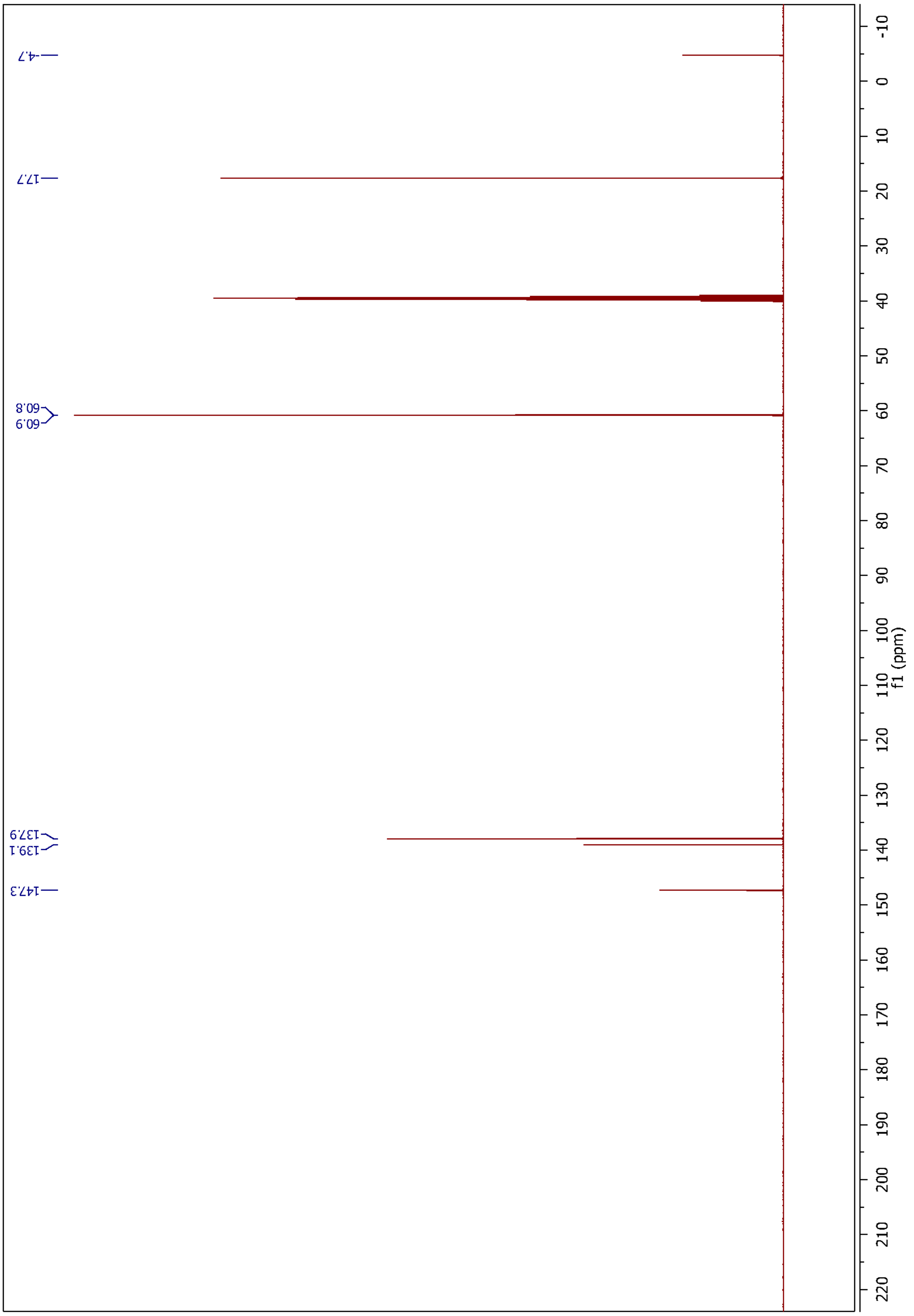

1-(3-Iodobicyclo[1.1.1]pentanyl)-2,4-dimethylpyridinium iodide (10q)

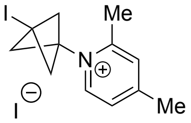

^1H NMR (400 MHz, DMSO-d_6)

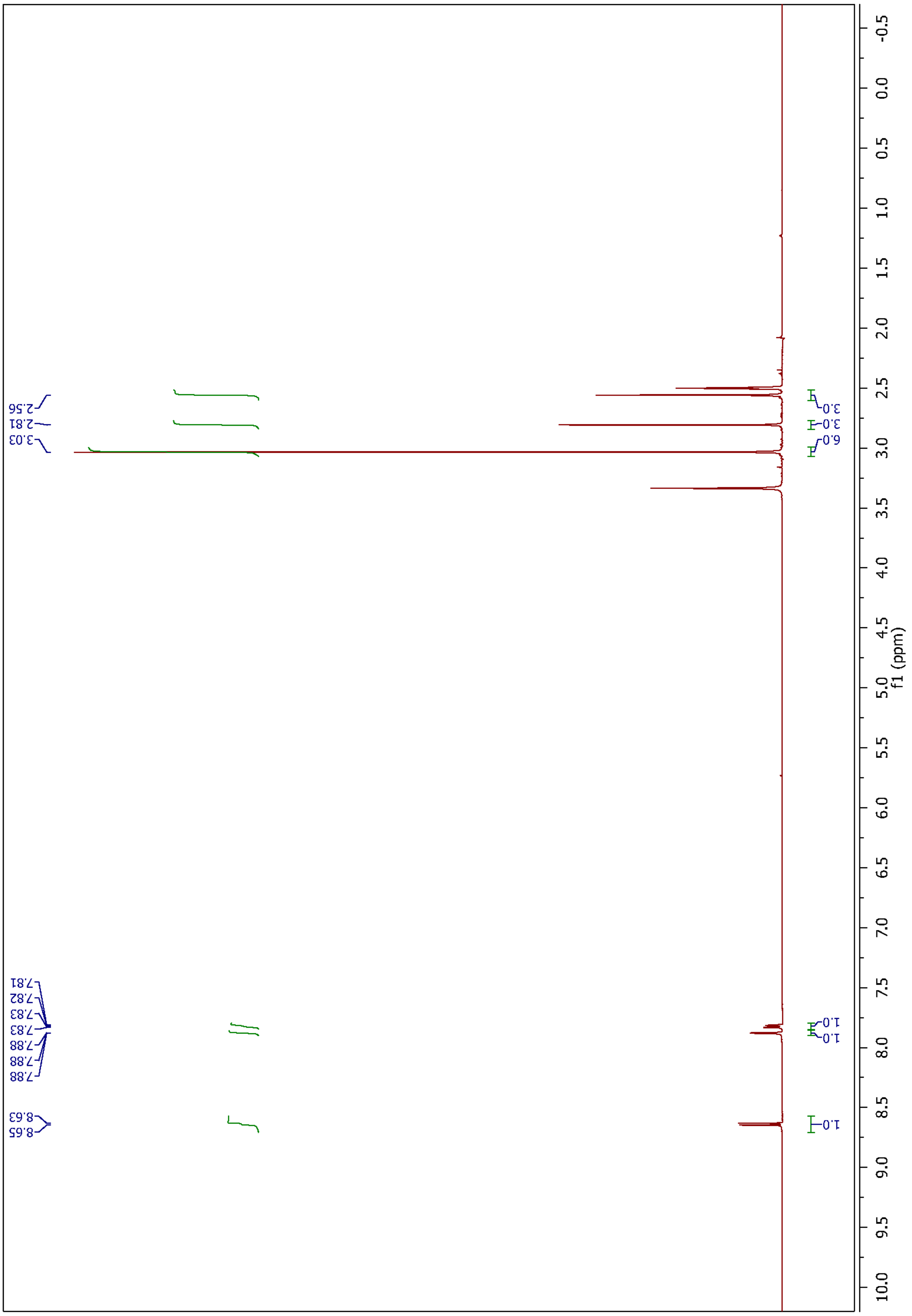

1-(3-Iodobicyclo[1.1.1]pentanyl)-2,4-dimethylpyridinium iodide (10q)

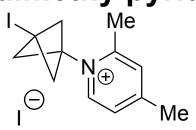

^{13}C NMR (126 MHz, DMSO-d_6)

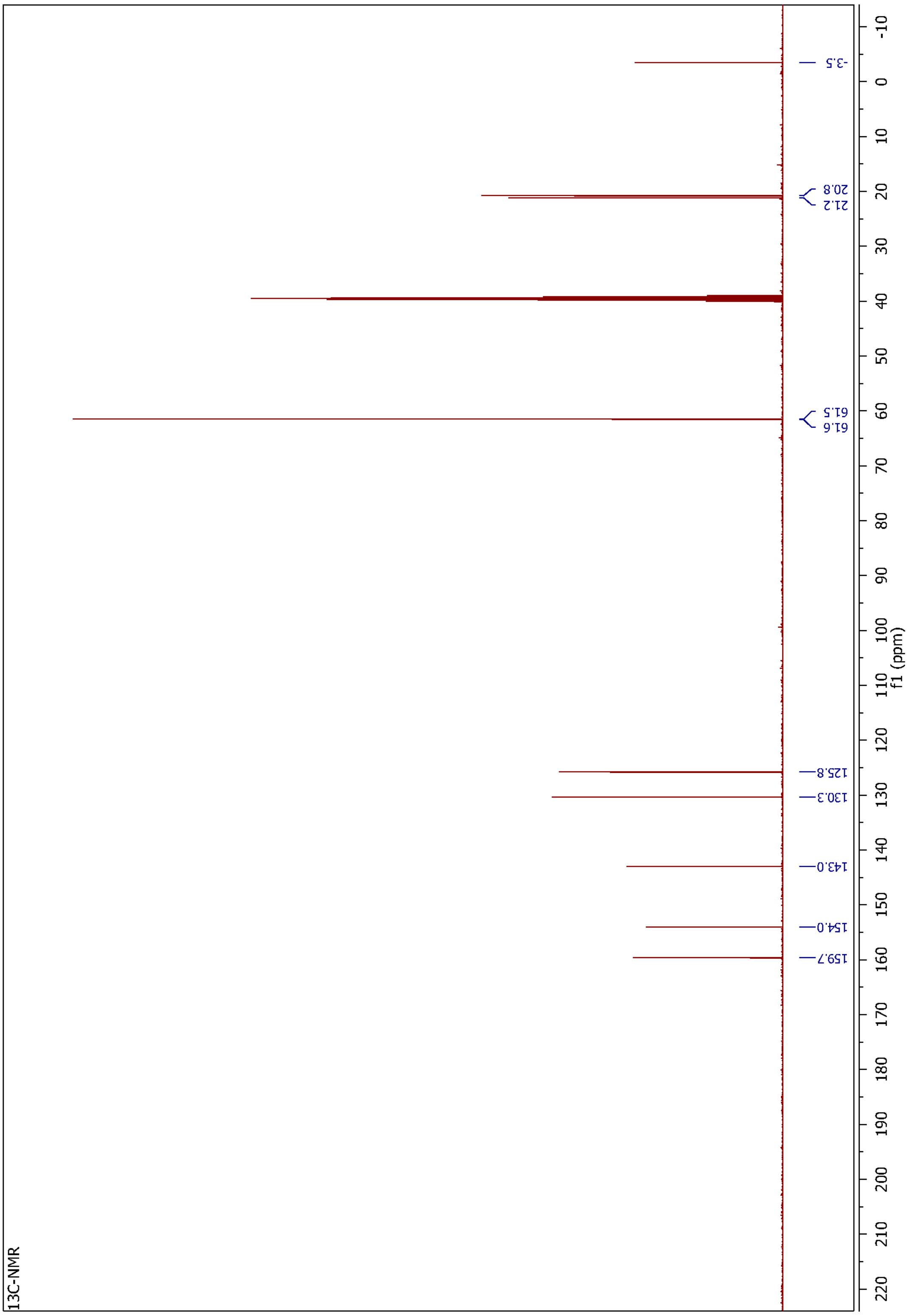

1-(3-Iodobicyclo[1.1.1]pentanyl)-6,7-dihydro-5*H*-cyclopenta[*b*]pyridinium iodide (10r)

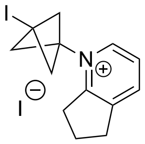

^{1}H NMR (500 MHz, DMSO-d_6)

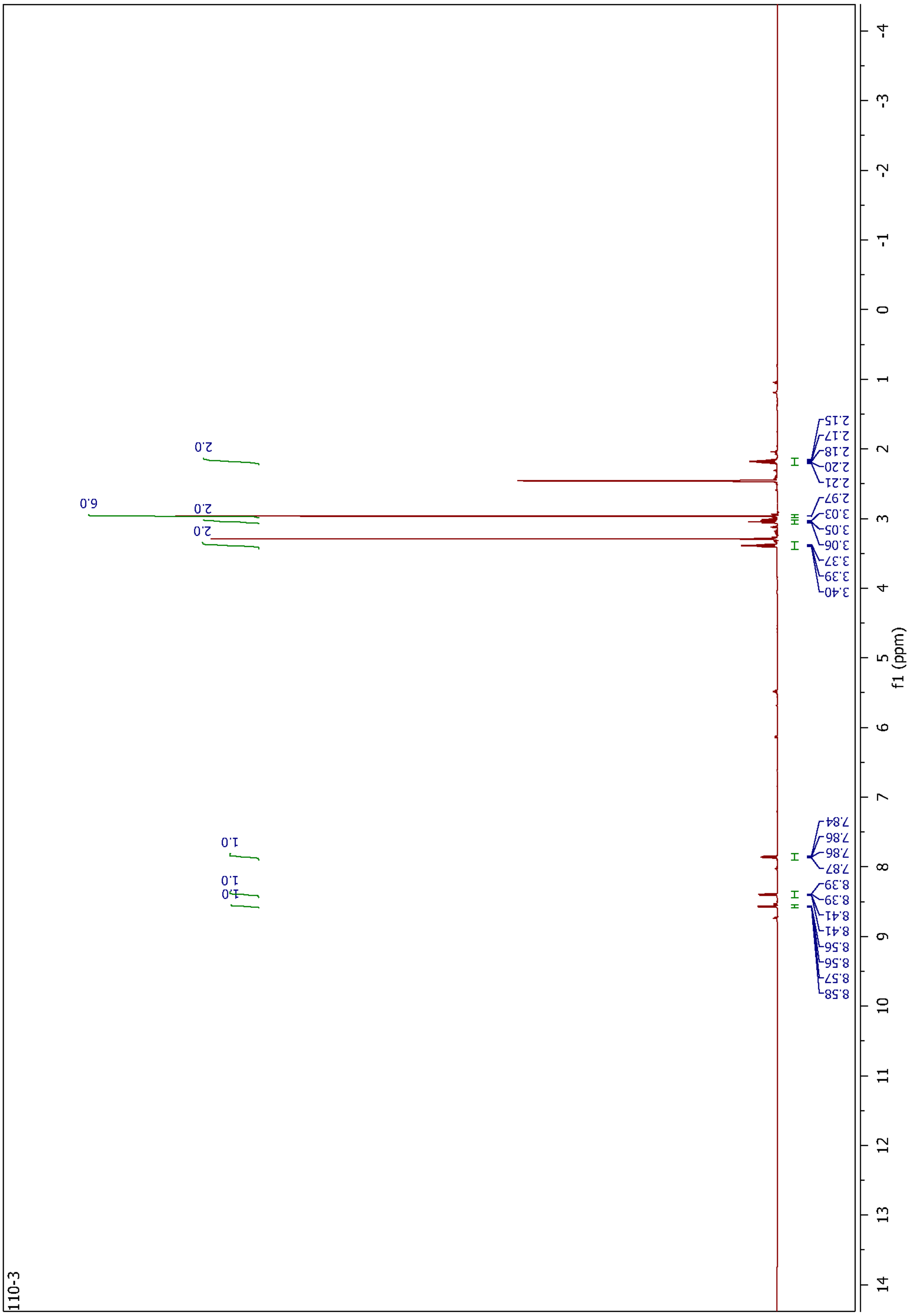

1-(3-Iodobicyclo[1.1.1]pentanyl)-6,7-dihydro-5*H*-cyclopenta[*b*]pyridinium iodide (10r)

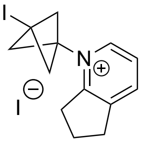

^{13}C NMR (126 MHz, DMSO-d_6)

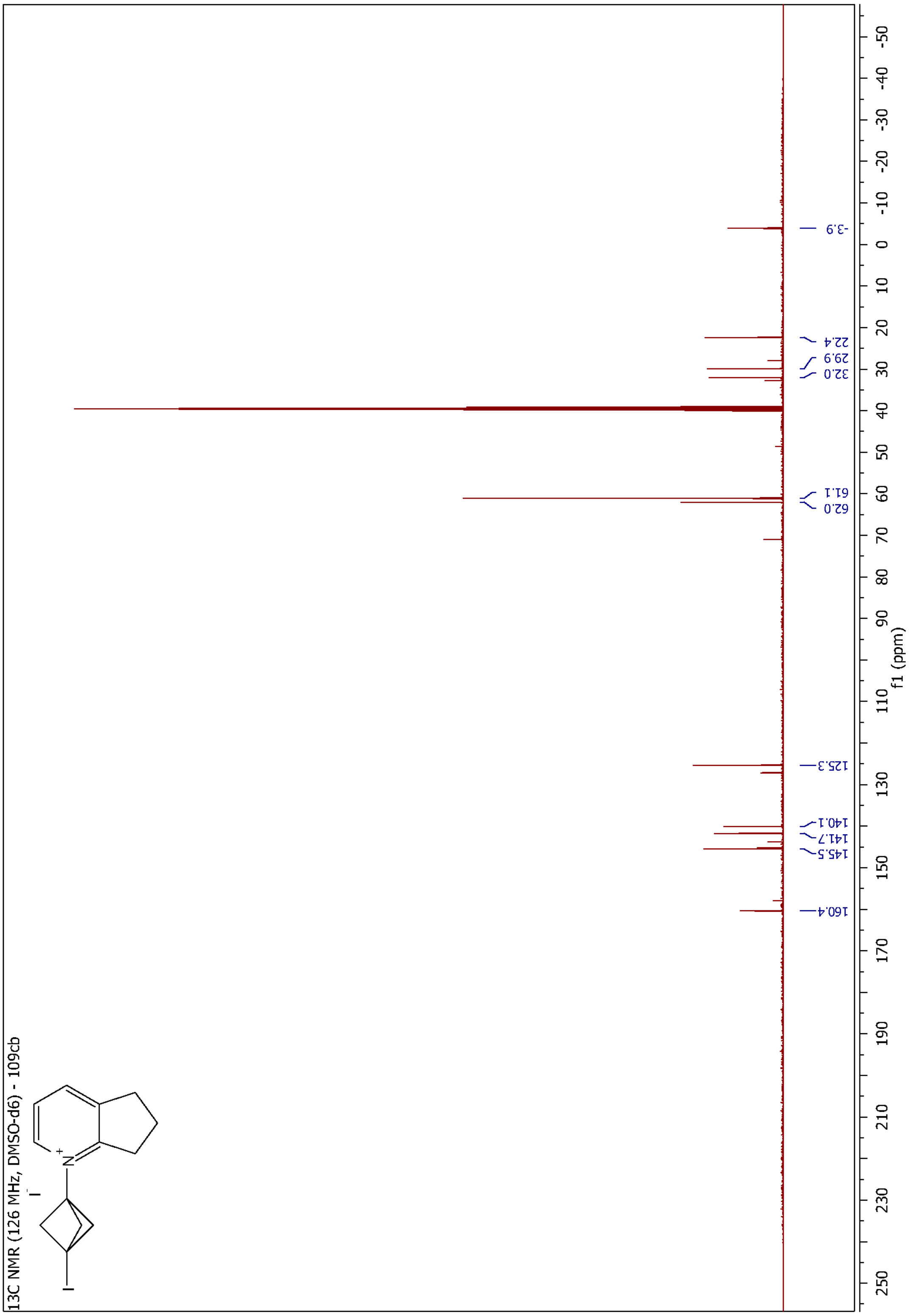

1-(3-Iodobicyclo[1.1.1]pentanyl)-2,3,5-trimethylpyridinium iodide (10s)

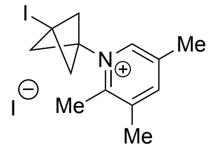

^{1}H NMR (400 MHz, DMSO-d_6)

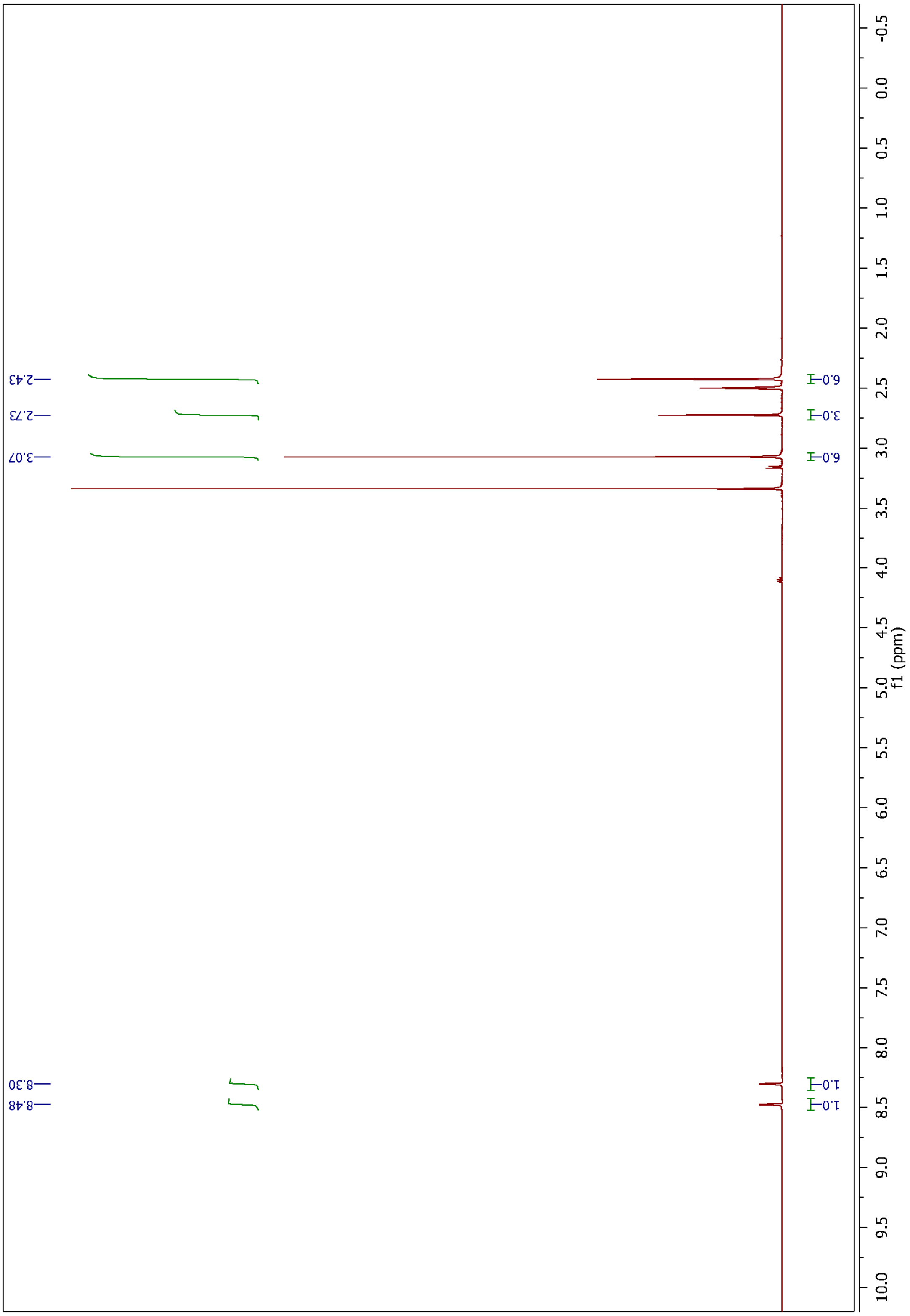

1-(3-Iodobicyclo[1.1.1]pentanyl)-2,3,5-trimethylpyridinium iodide (10s)

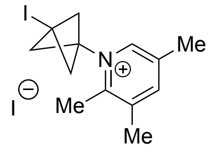

^{13}C NMR (126 MHz, DMSO-d_6)

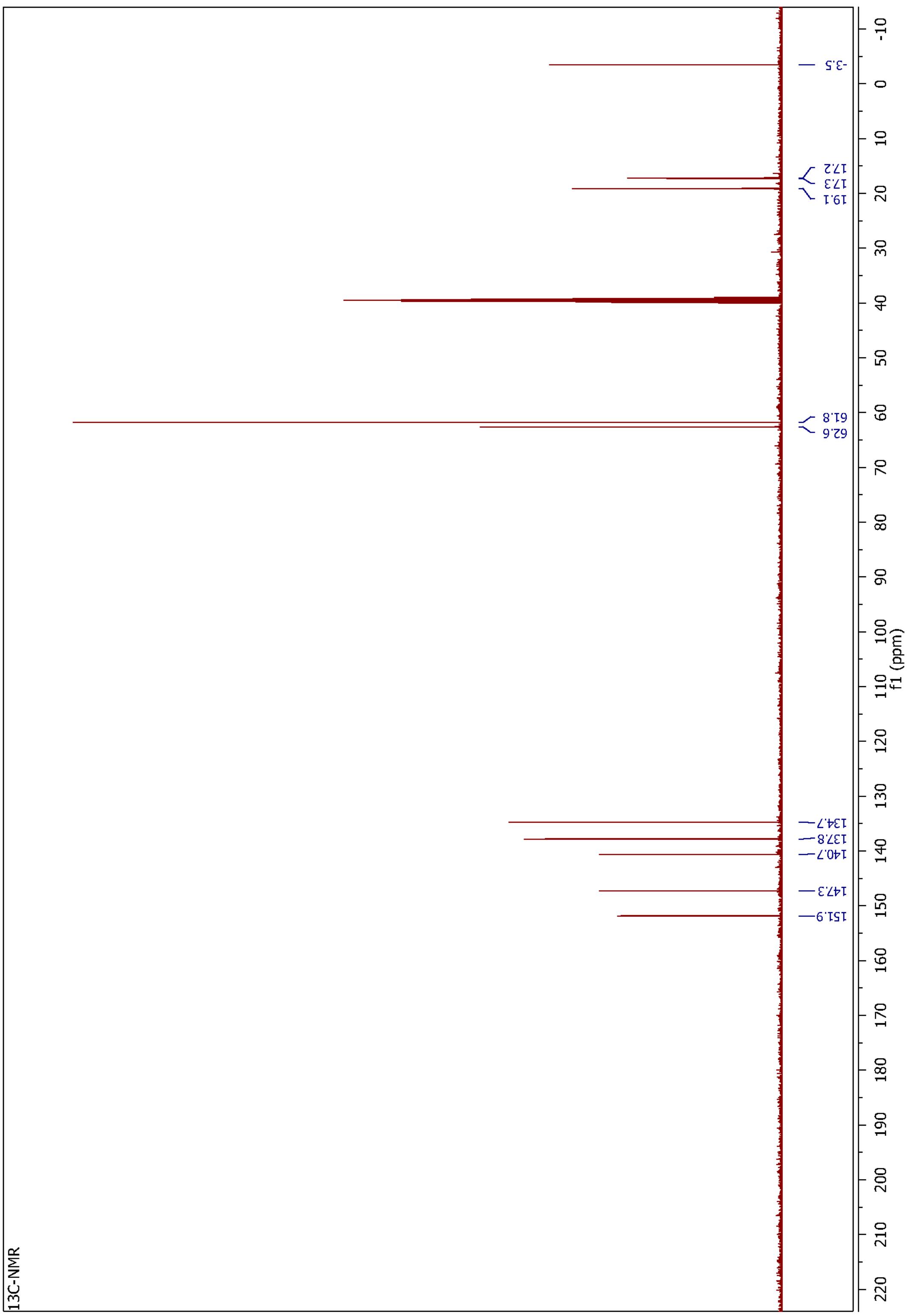

1-(3-Iodobicyclo[1.1.1]pentanyl)-[3,3'-bipyridin]-ium iodide (10t)

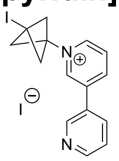

^{1}H NMR (400 MHz, DMSO-d_6)

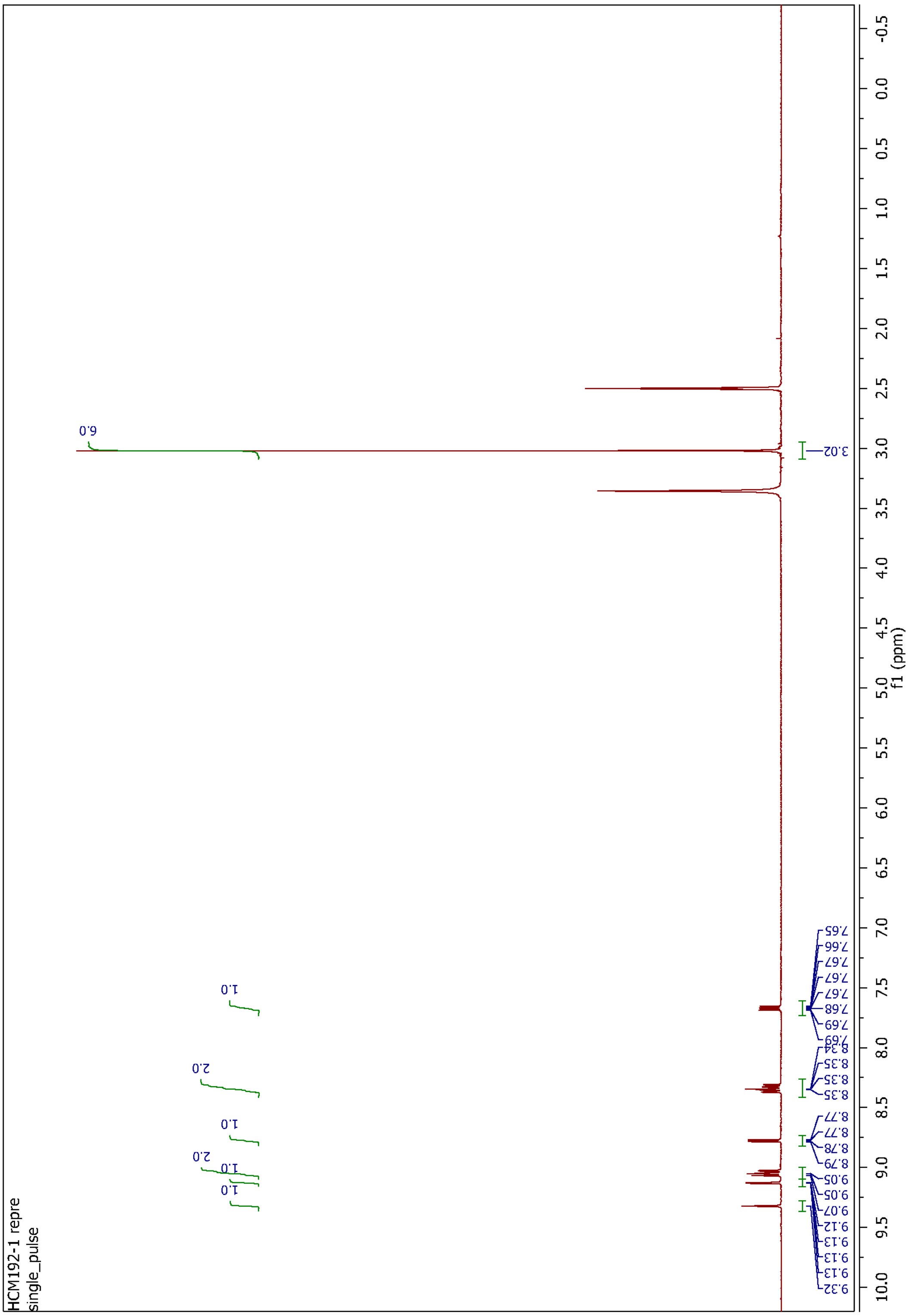

1-(3-Iodobicyclo[1.1.1]pentanyl)-[3,3'-bipyridin]-ium iodide (10t)

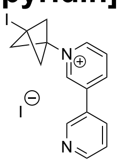

^{13}C NMR (101 MHz, DMSO-d_6)

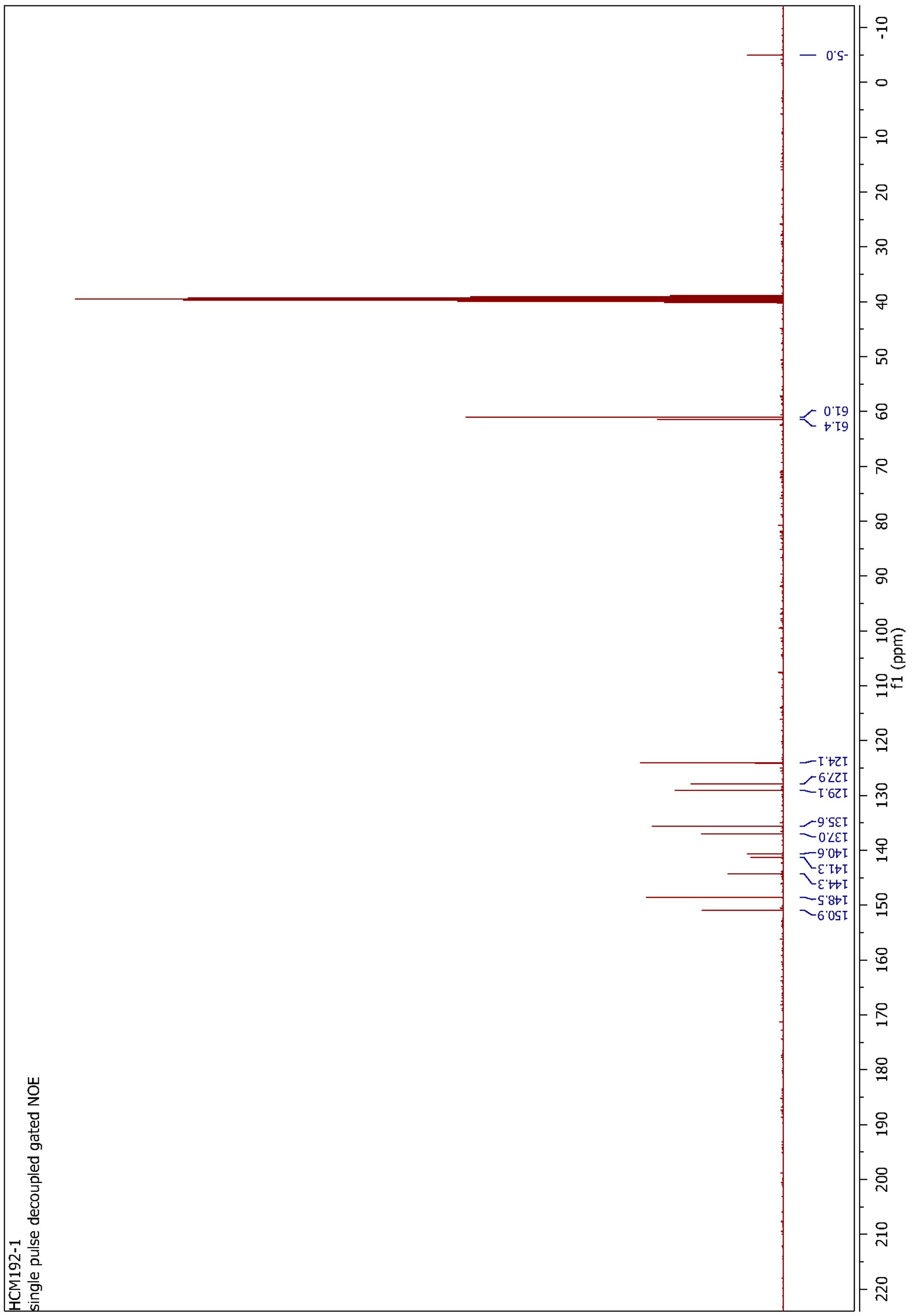

1-(3-iodobicyclo[1.1.1]pentanyl)-*N*,*N*-dimethylpyridin-4-aminium iodide (10u)

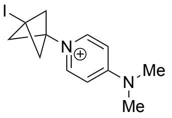

^{1}H NMR (500 MHz, DMSO-d_6)

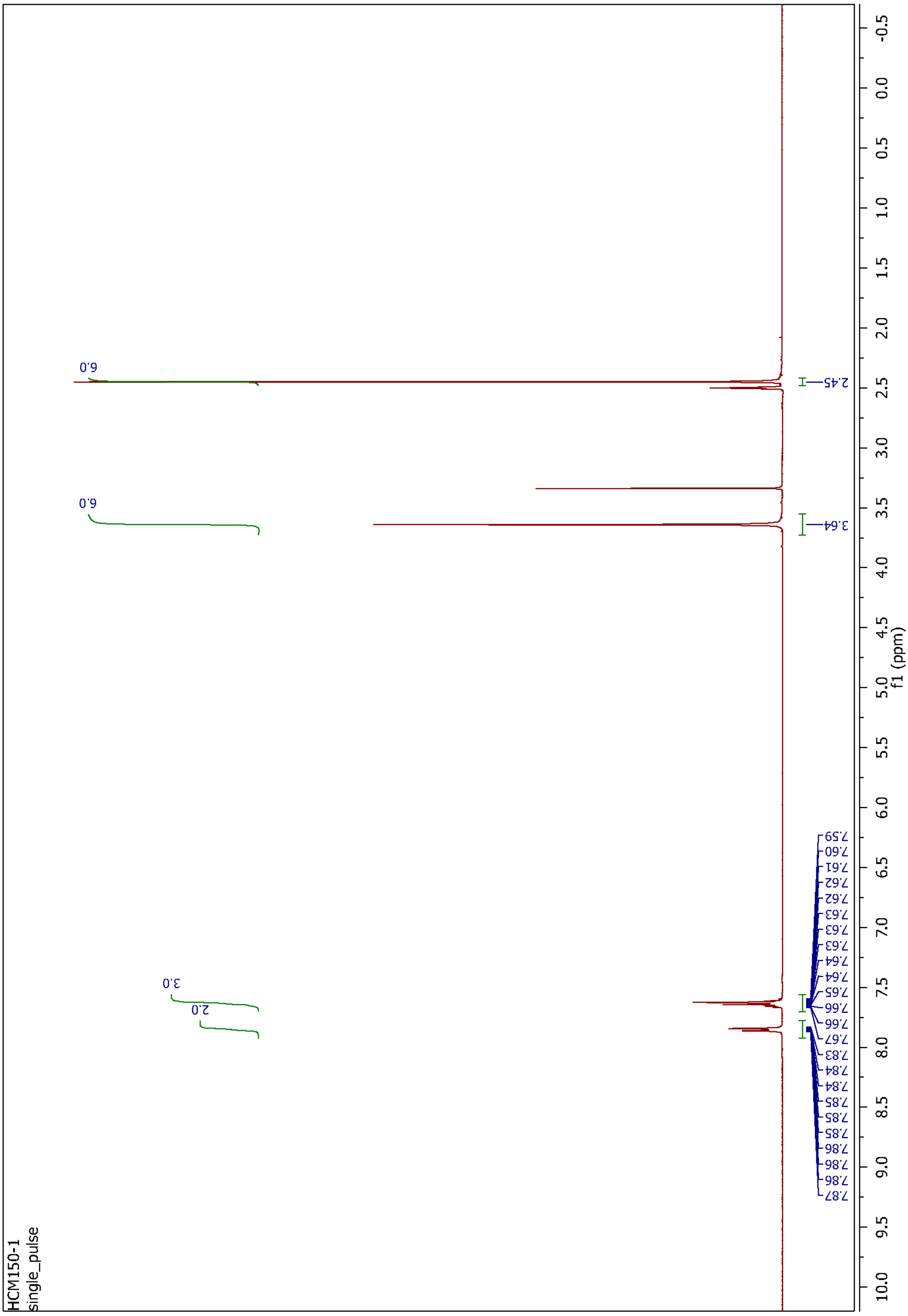

1-(3-iodobicyclo[1.1.1]pentanyl)-*N*,*N*-dimethylpyridin-4-aminium iodide (10u)

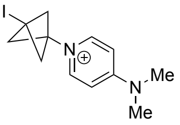

^{13}C NMR (126 MHz, DMSO-d_6)

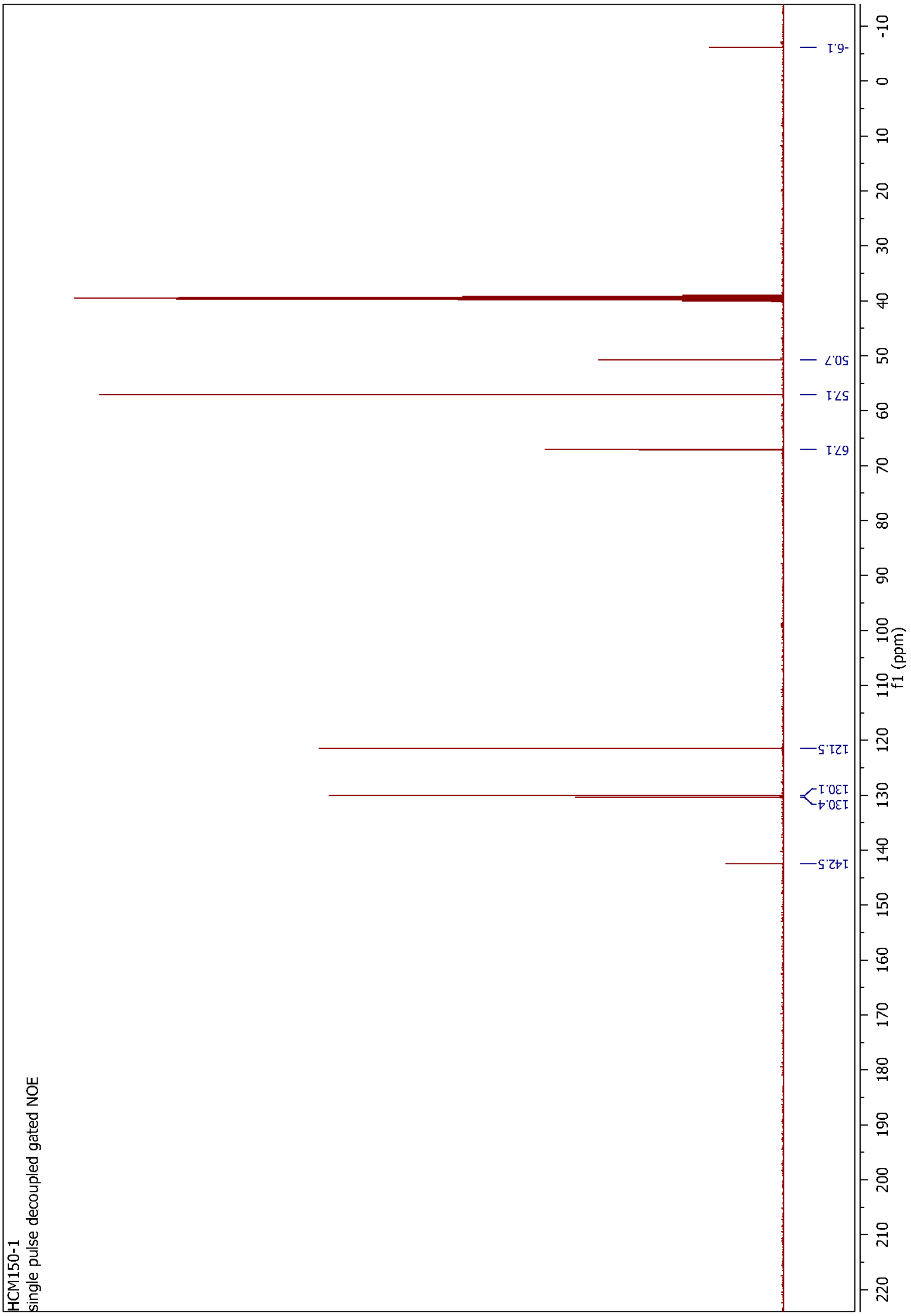

7-Chloro-4-(3-iodobicyclo[1.1.1]pentanyl)thieno[3,2-*b*]pyridinium iodide (10v)

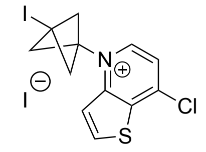

^{1}H NMR (500 MHz, DMSO-d_6)

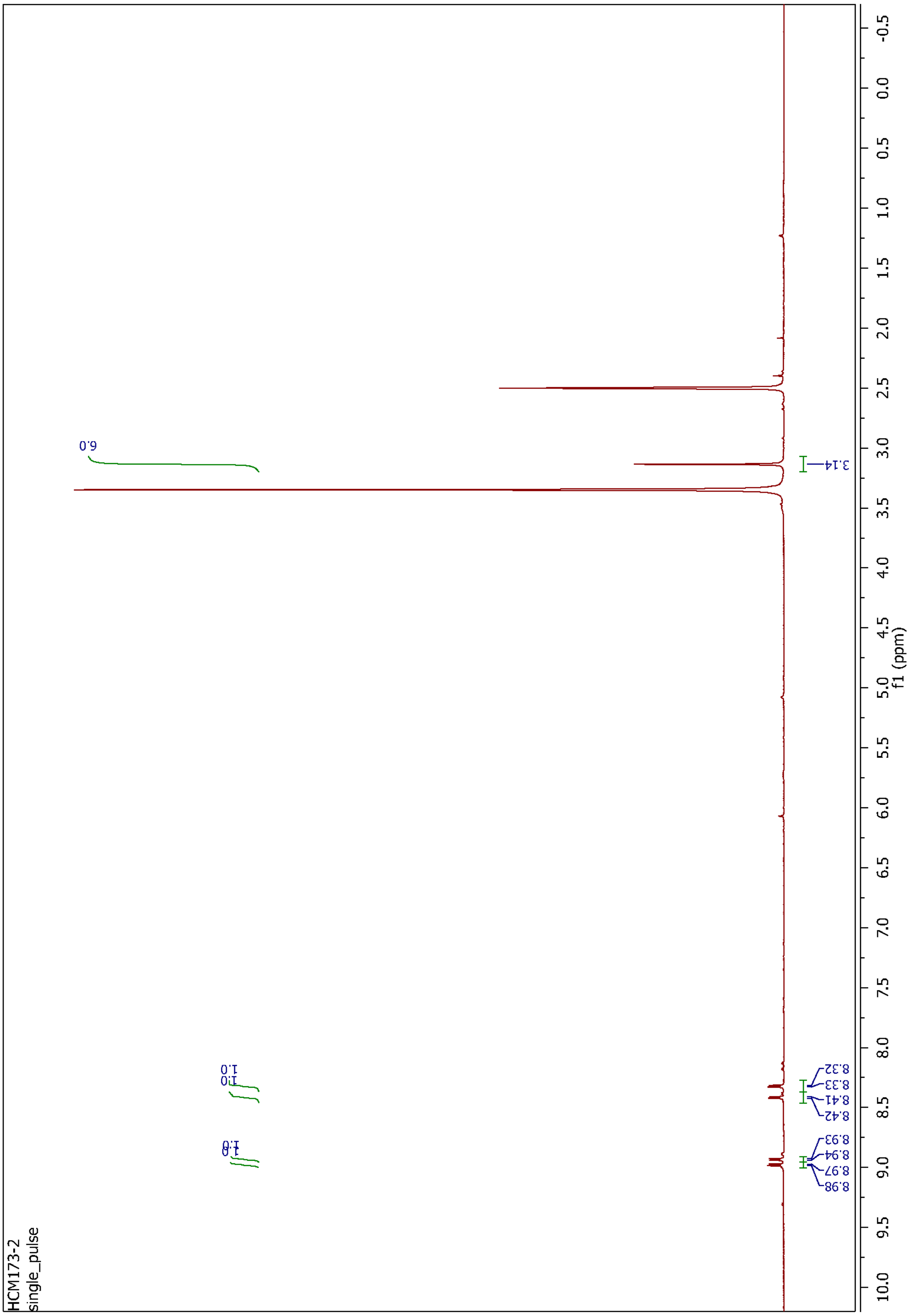

7-Chloro-4-(3-iodobicyclo[1.1.1]pentanyl)thieno[3,2-*b*]pyridinium iodide (10v)

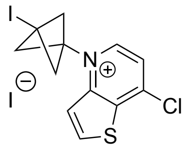

^{13}C NMR (126 MHz, DMSO-d_6)

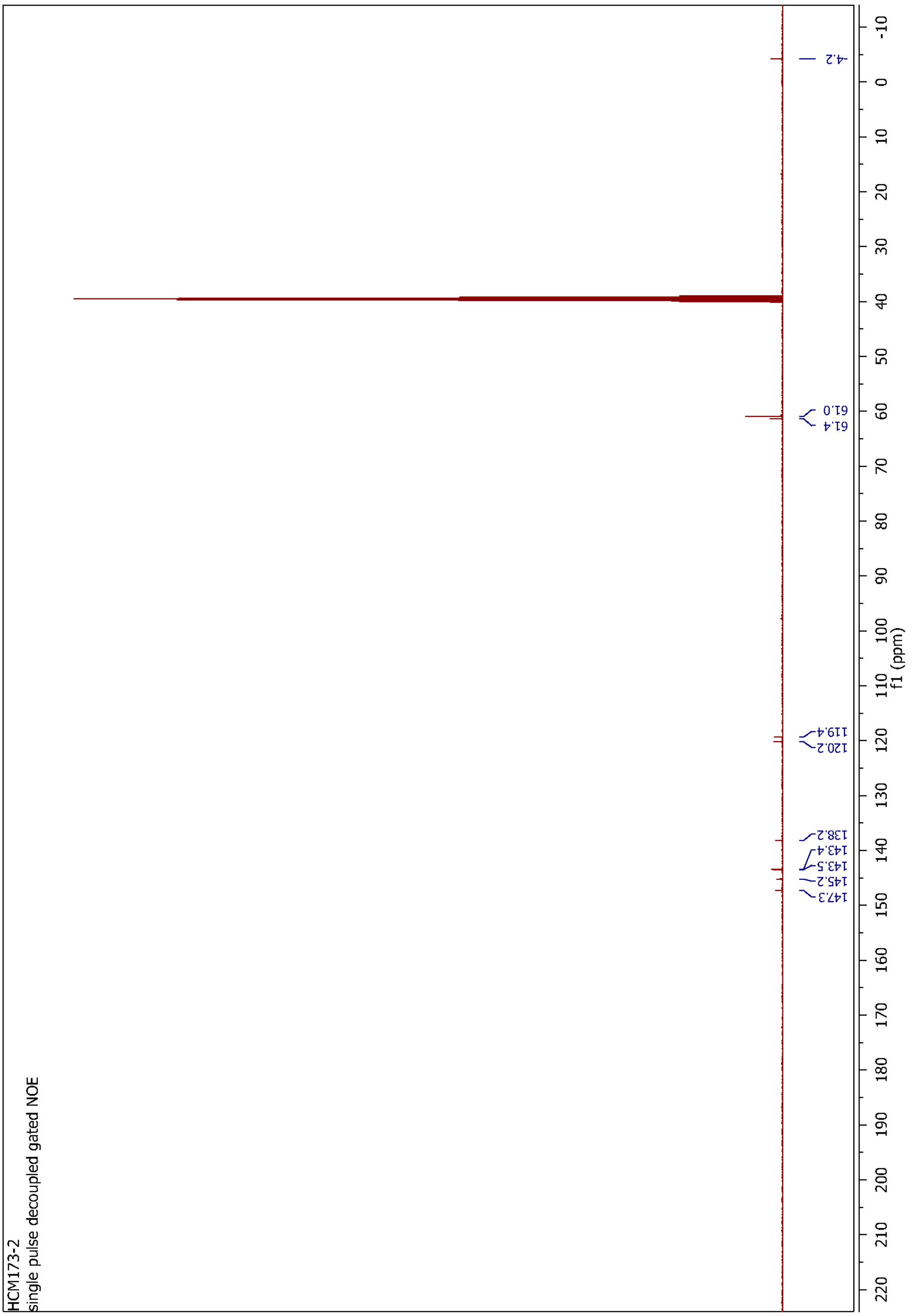

7-(3-Iodobicyclo[1.1.1]pentanyl)-1*H*-pyrrolo[2,3-*b*]pyridinium iodide (10w)

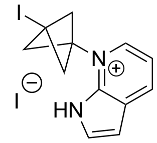

^{1}H NMR (500 MHz, DMSO-d_6)

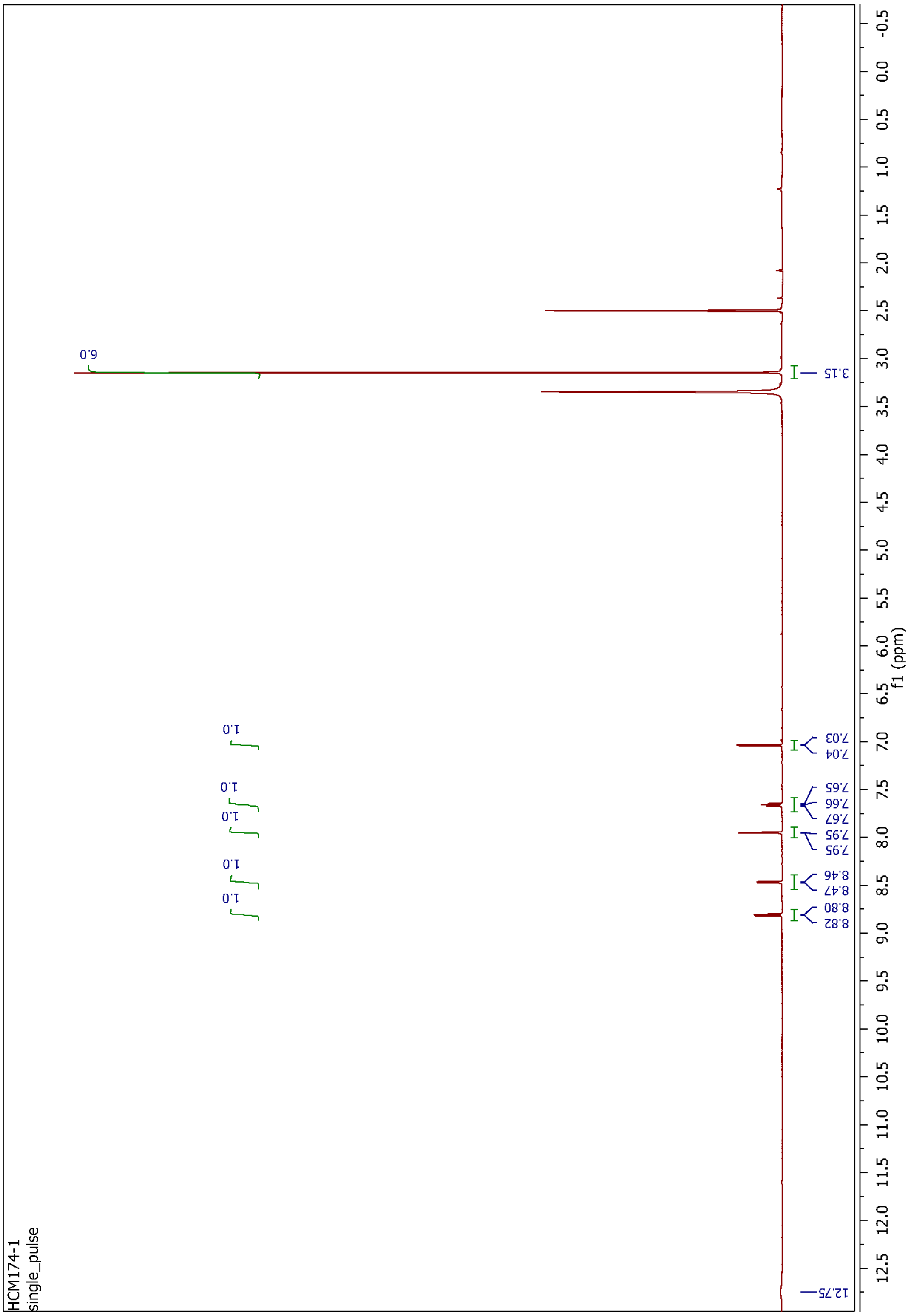

7-(3-Iodobicyclo[1.1.1]pentanyl)-1*H*-pyrrolo[2,3-*b*]pyridinium iodide (10w)

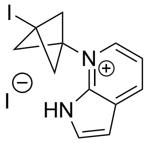

^{13}C NMR (126 MHz, DMSO-d_6)

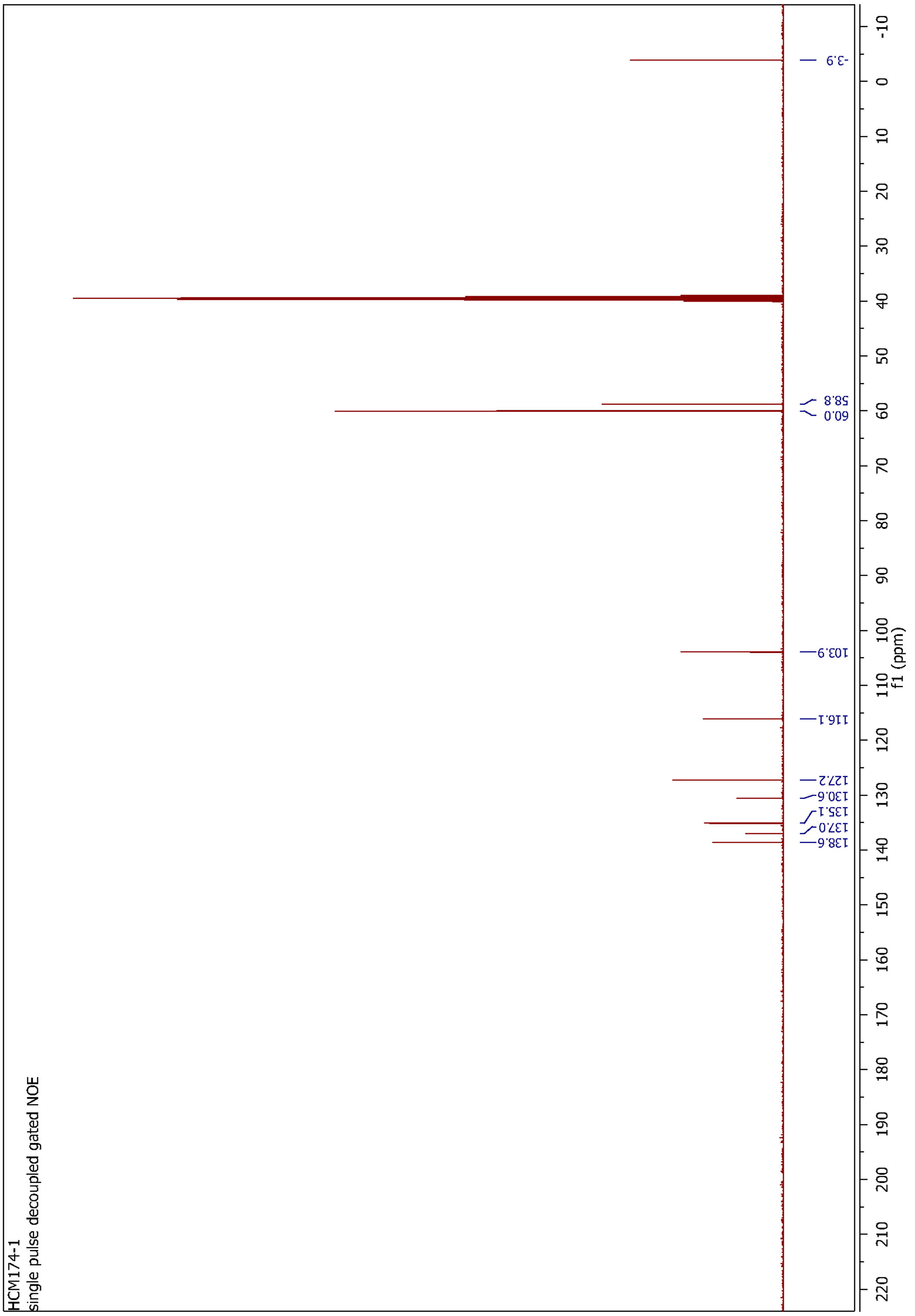

(*E*)-2-(hydroxyiminomethyl)-1-(3-iodobicyclo[1.1.1]pentanyl)pyridinium iodide (10x).

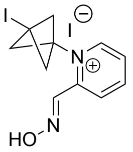

^{1}H NMR (500 MHz, DMSO-d_6)

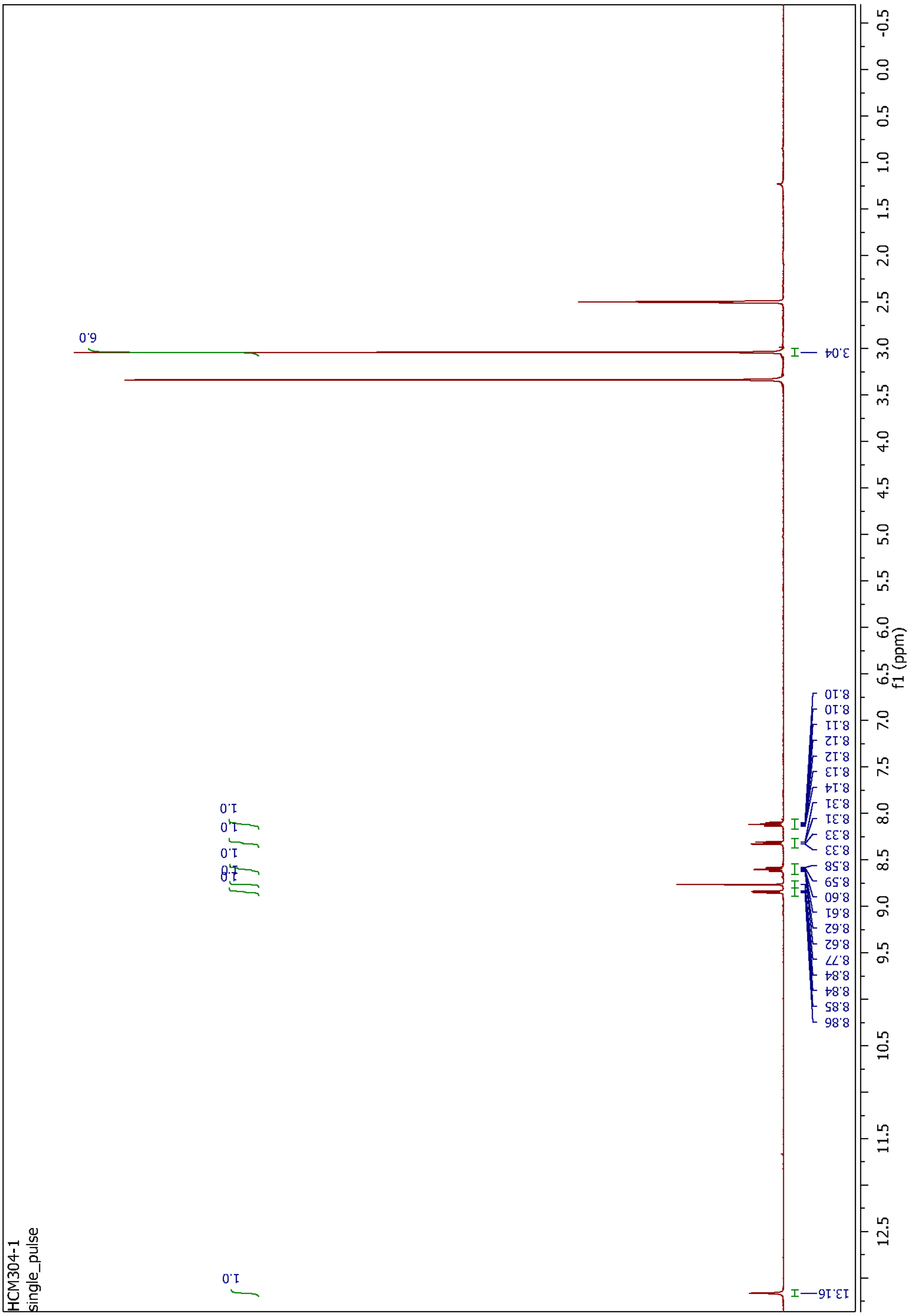

(E)-2-(hydroxyiminomethyl)-1-(3-iodobicyclo[1.1.1]pentanyl)pyridinium iodide (10x).

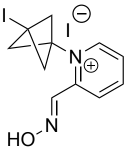

^{13}C NMR (126 MHz, DMSO-d_6)

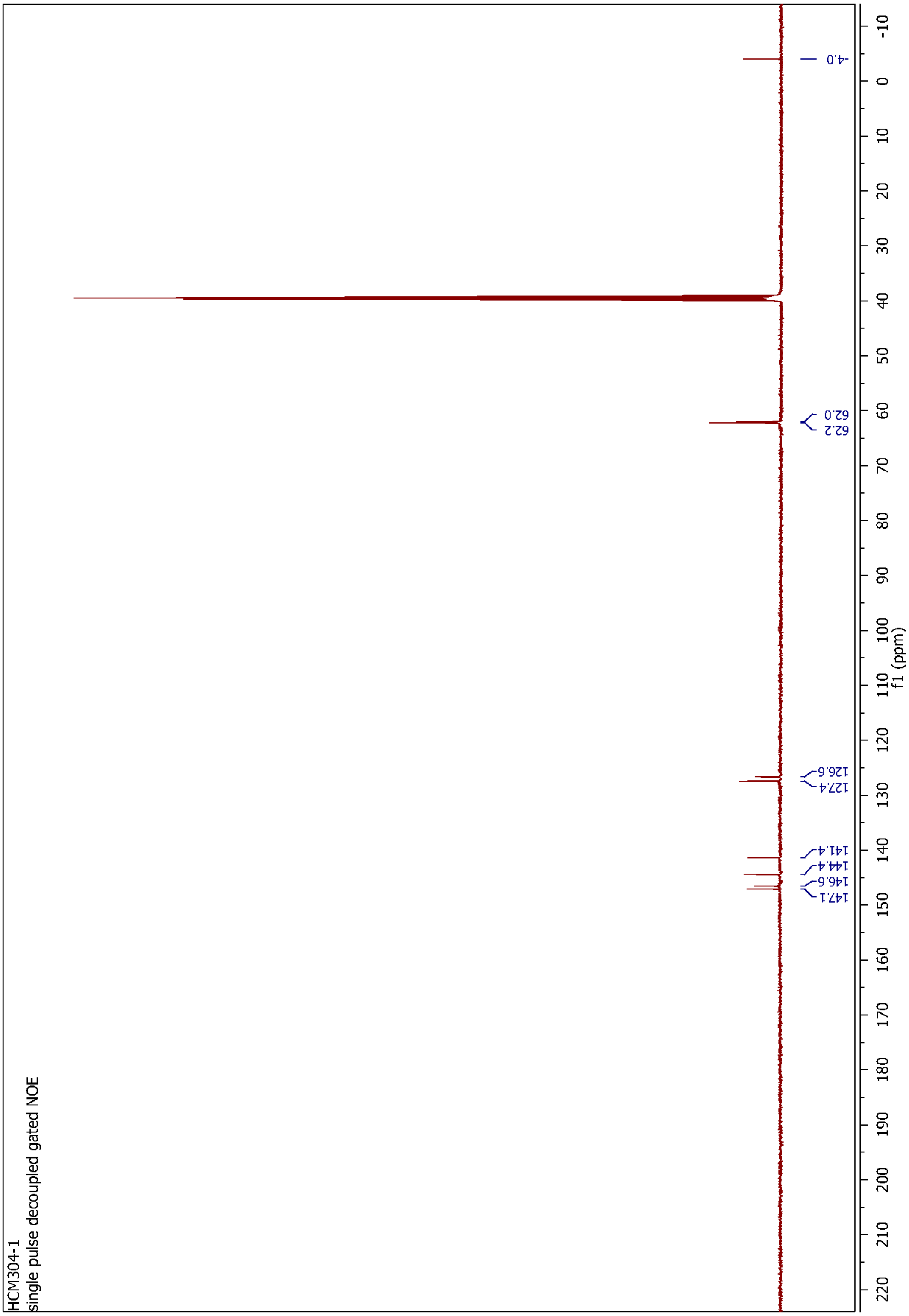

2-Chloro-1-(3-methylenecyclobutyl)pyridinium iodide (11).

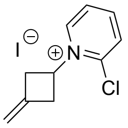

^{1}H NMR (400 MHz, DMSO-d_6)

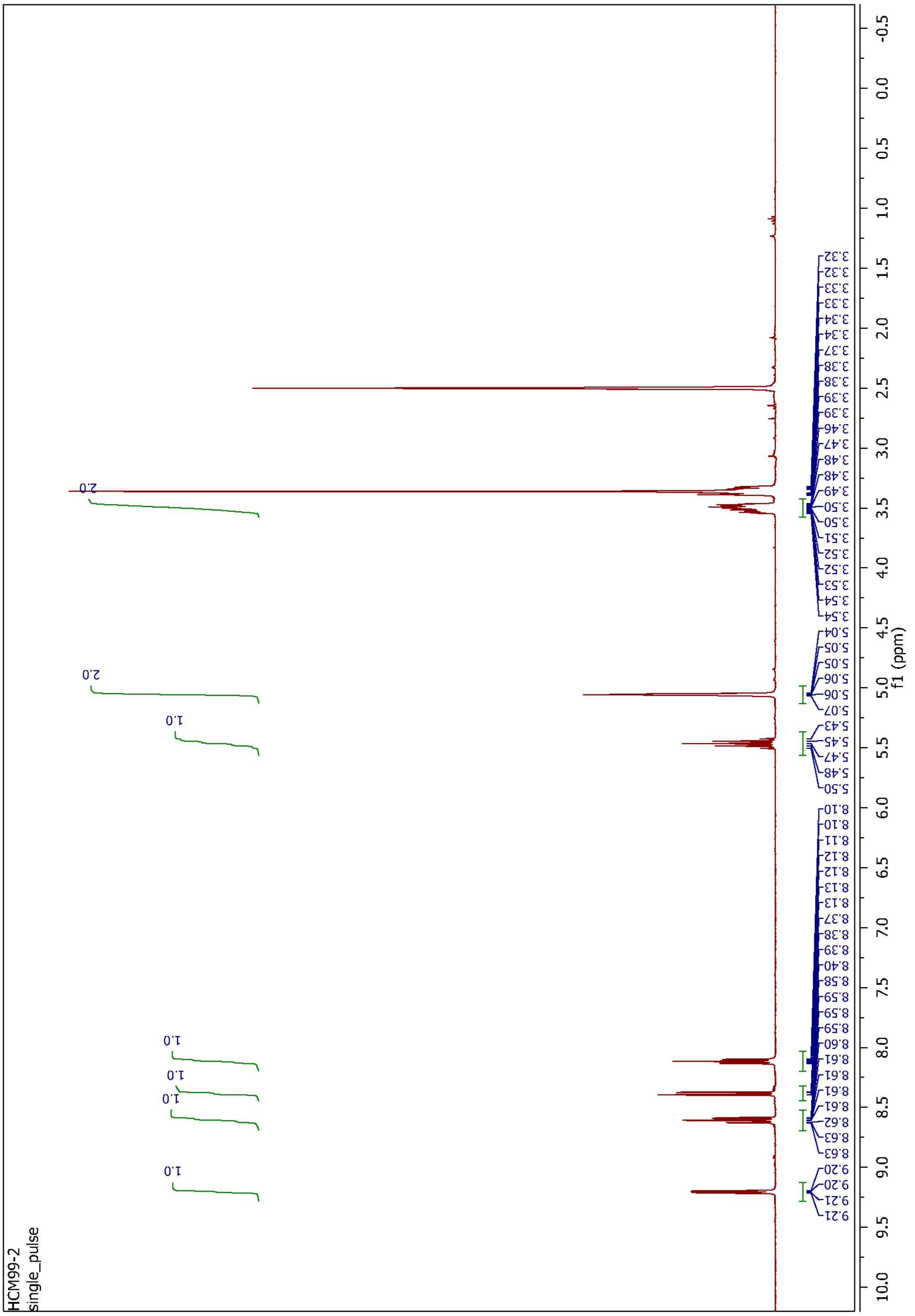

2-Chloro-1-(3-methylenecyclobutyl)pyridinium iodide (11).

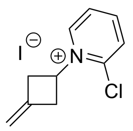

^{13}C NMR (101 MHz, DMSO-d_6)

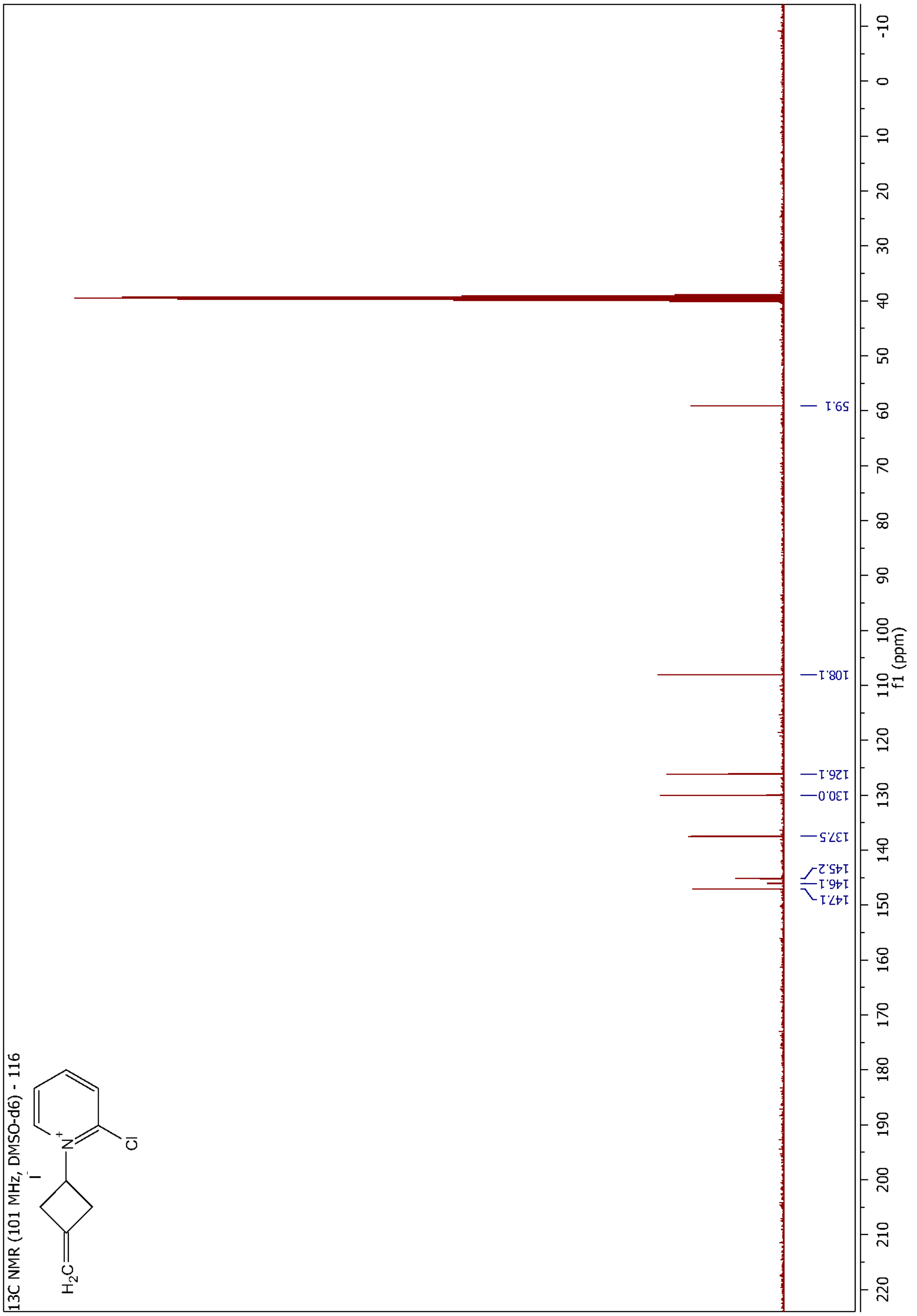

1-(3-Iodobicyclo[1.1.1]pentanyl)quinolinium iodide (12a).

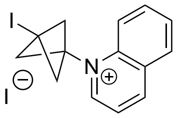

^{1}H NMR (500 MHz, DMSO-d_6)

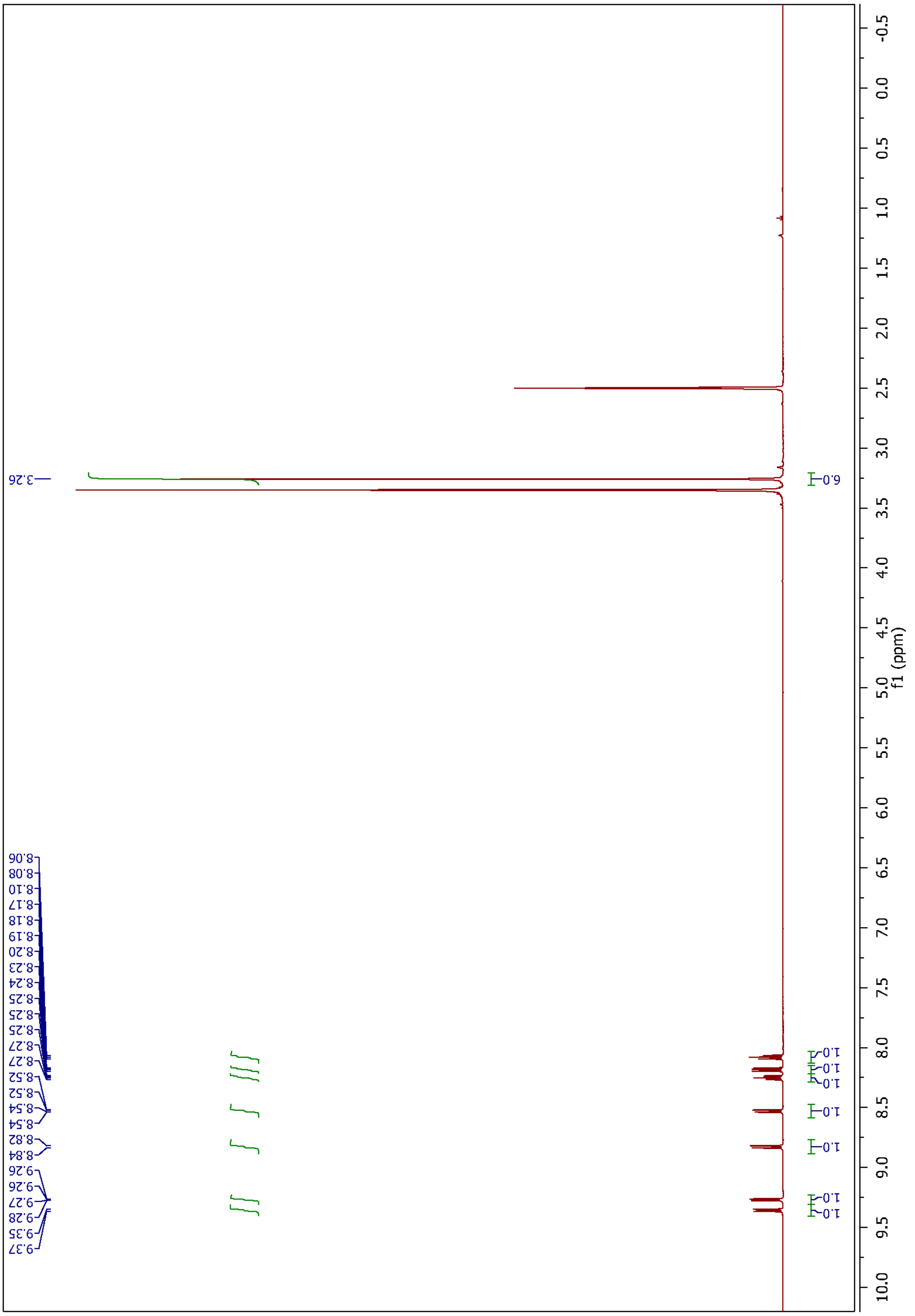

1-(3-Iodobicyclo[1.1.1]pentanyl)quinolinium iodide (12a).

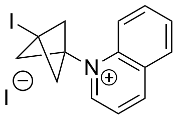

^{13}C NMR (126 MHz, DMSO-d_6)

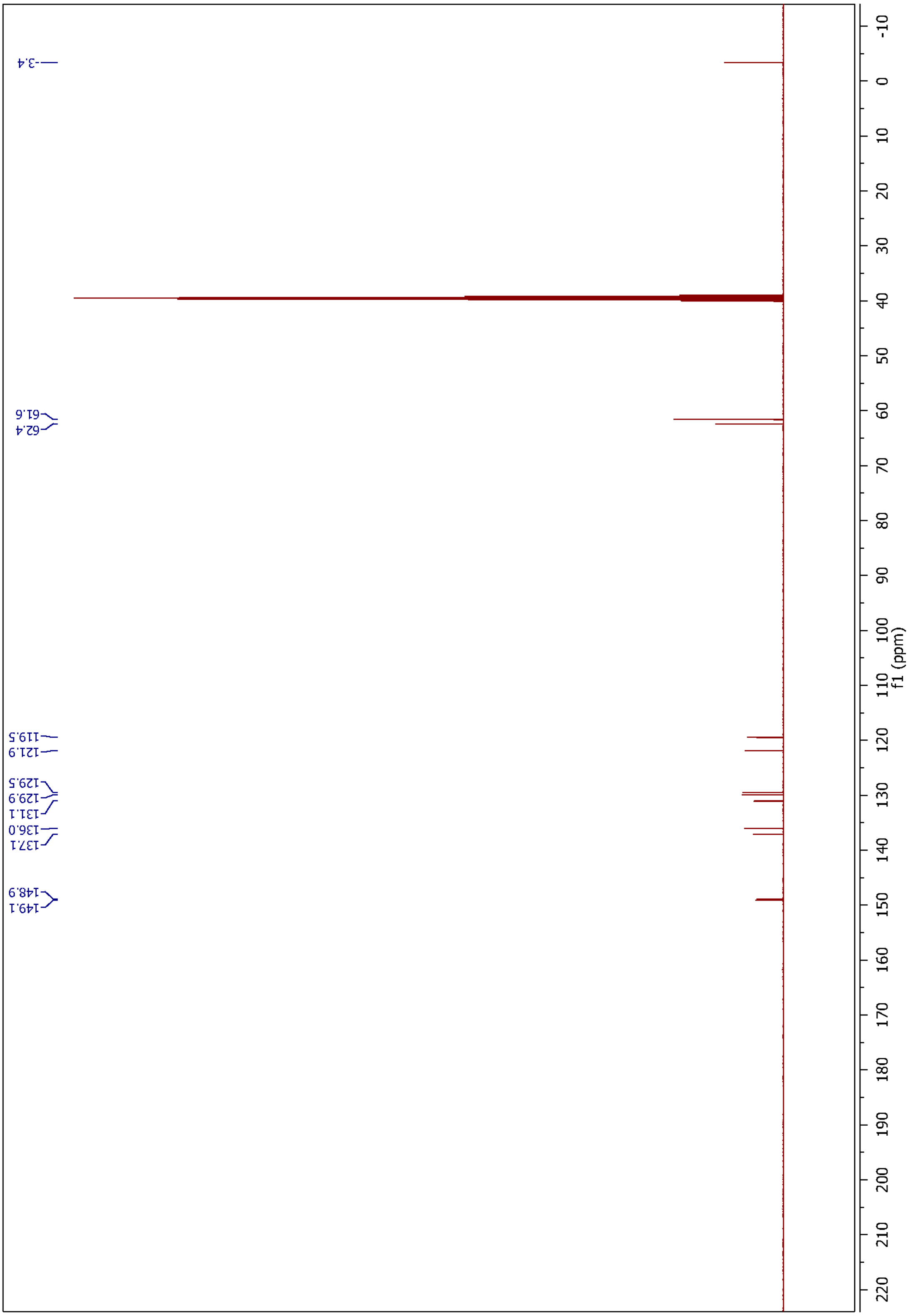

1-(3-Iodobicyclo[1.1.1]pentanyl)-4-methoxyquinolinium iodide (12b).

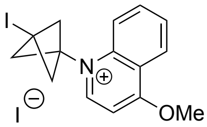

^{1}H NMR (500 MHz, DMSO-d_6)

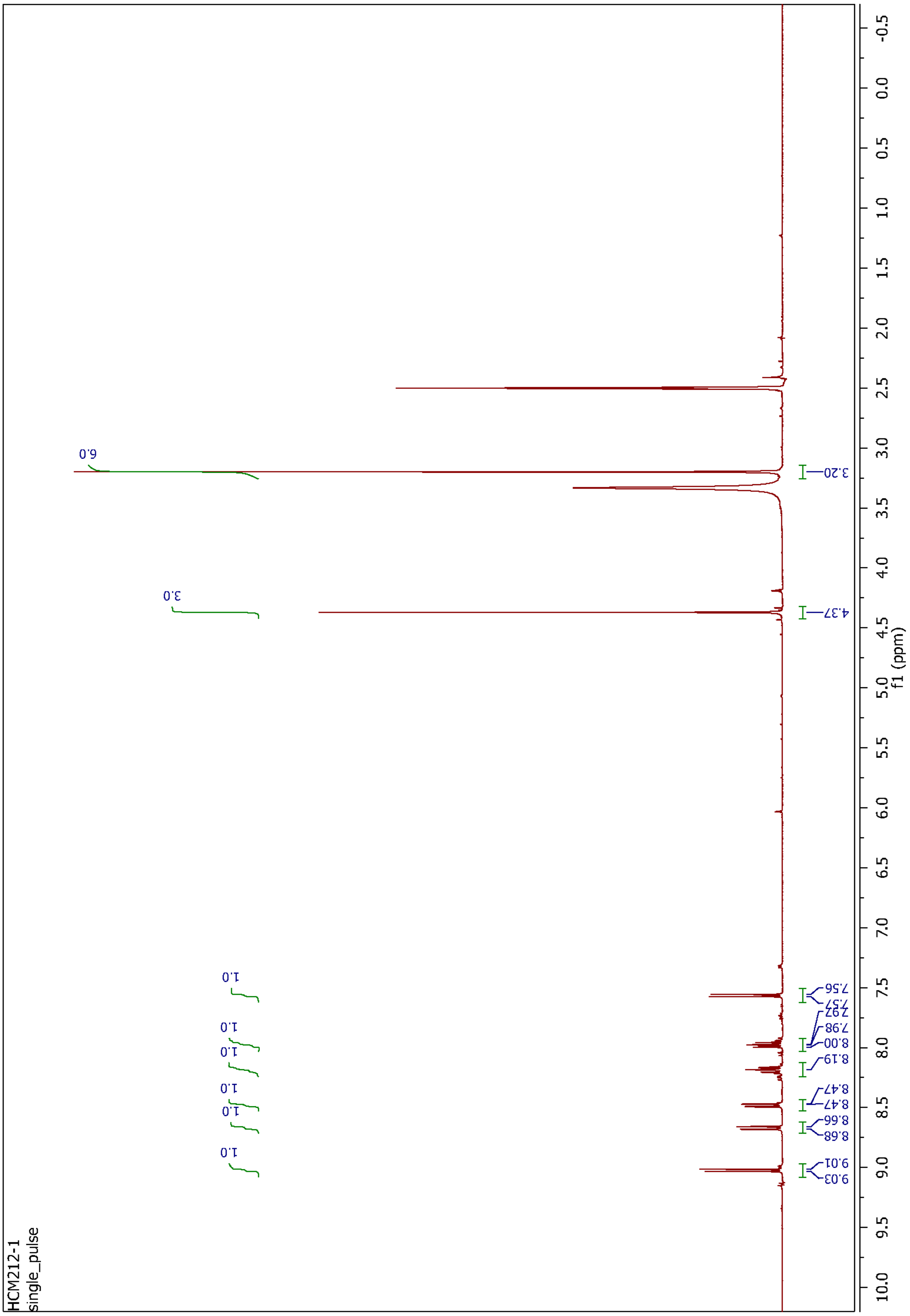

1-(3-Iodobicyclo[1.1.1]pentanyl)-4-methoxyquinolinium iodide (12b).

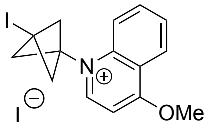

^{13}C NMR (126 MHz, DMSO-d_6)

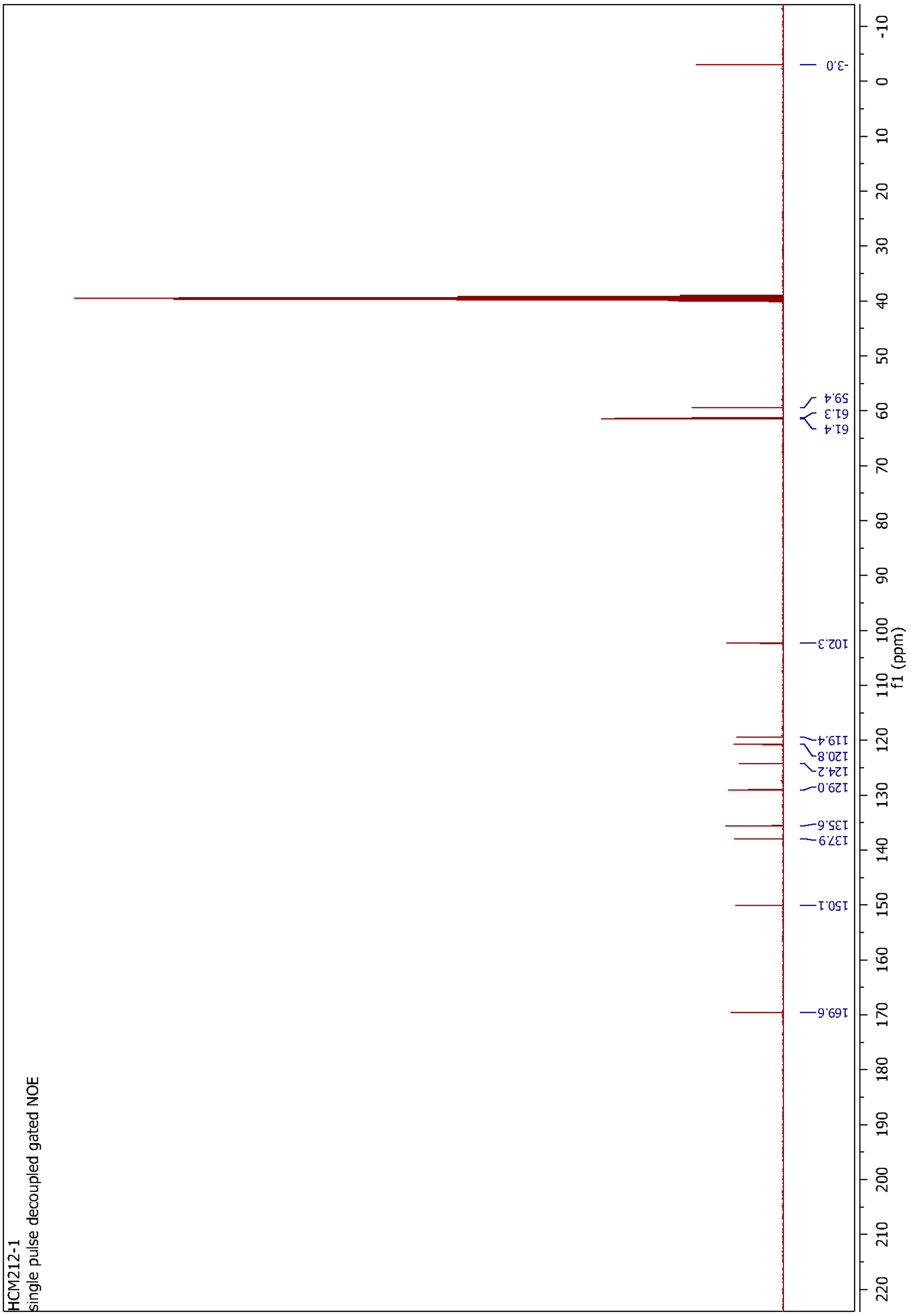

1-(3-Iodobicyclo[1.1.1]pentanyl)-5-bromoquinolinium iodide (12c).

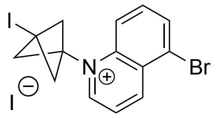

^{1}H NMR (500 MHz, DMSO-d_6)

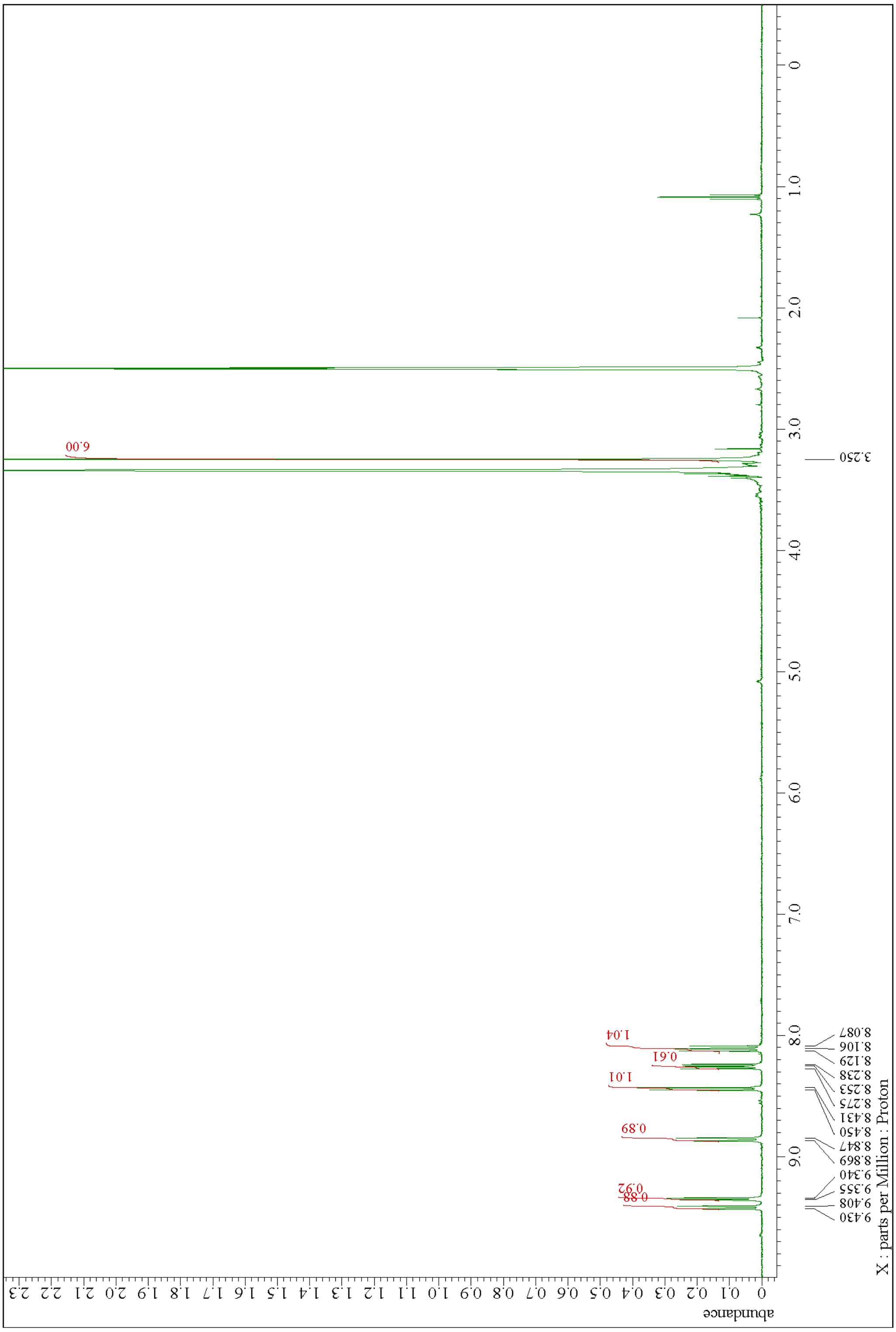

1-(3-Iodobicyclo[1.1.1]pentanyl)-5-bromoquinolinium iodide (12c).

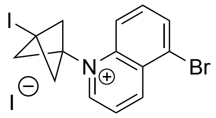

^{13}C NMR (126 MHz, DMSO-d_6)

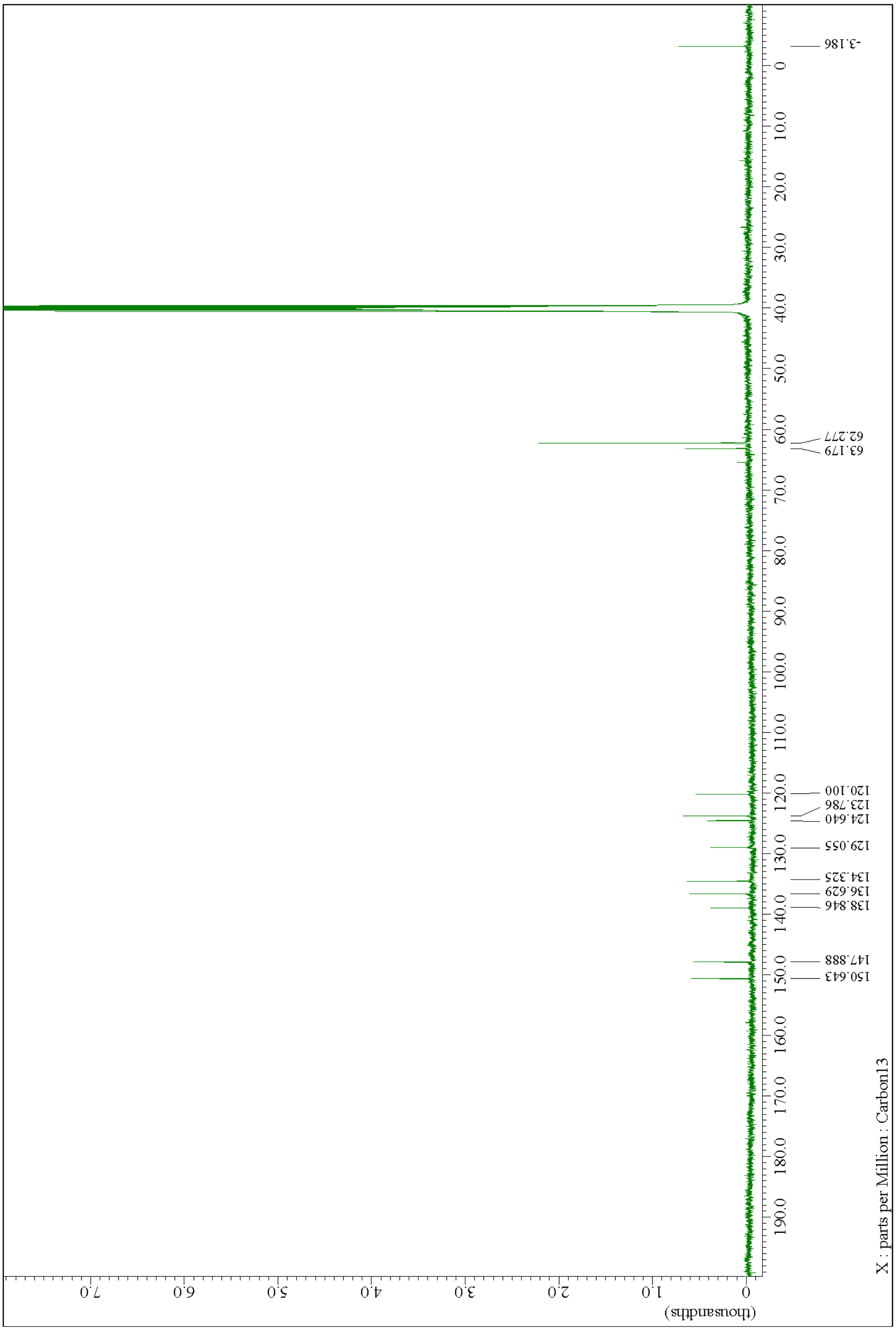

1-(3-Iodobicyclo[1.1.1]pentanyl)-7-chloro-4-methoxyquinolinium iodide (12d).

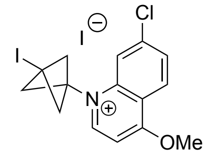

^{1}H NMR (500 MHz, DMSO-d_6)

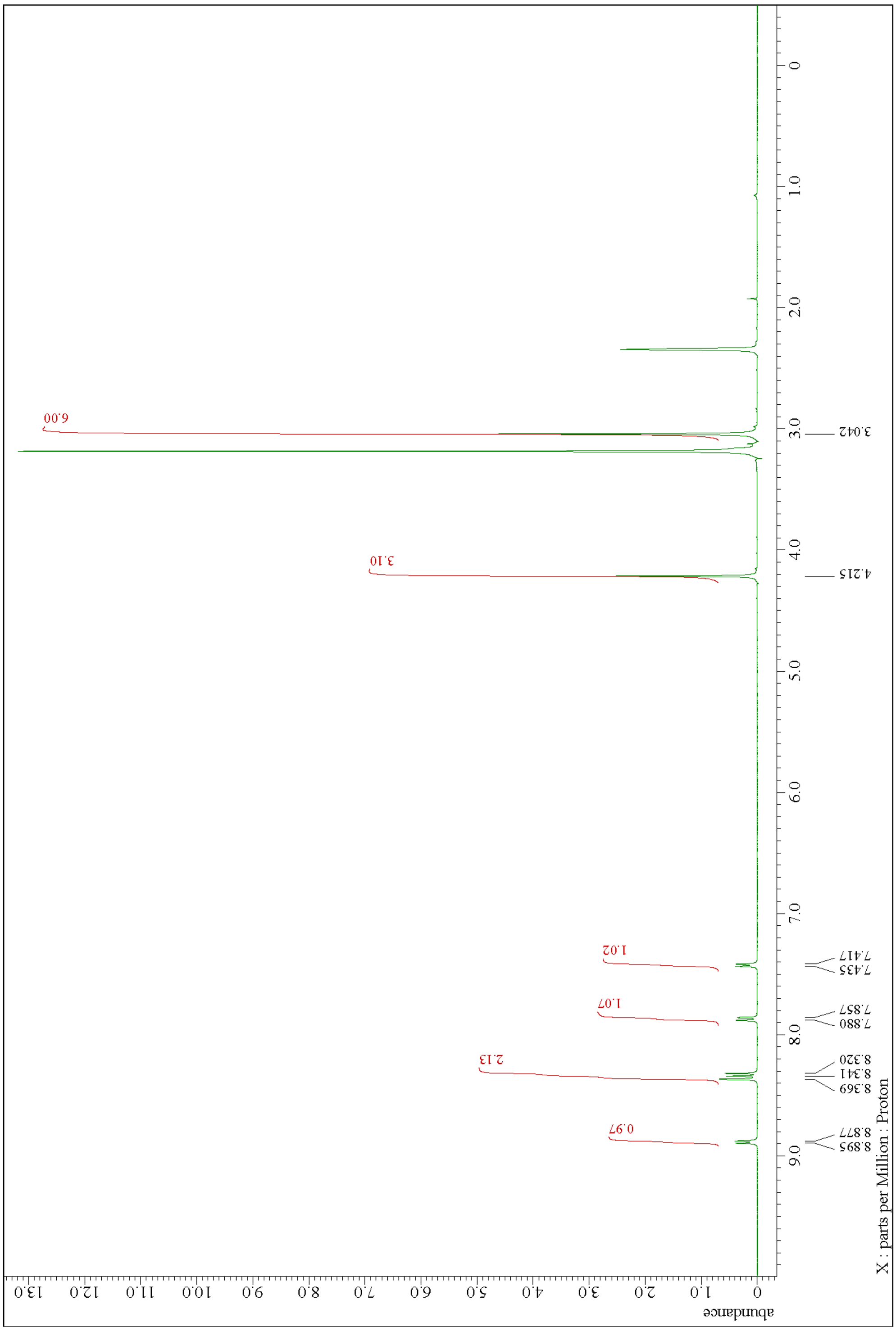

1-(3-Iodobicyclo[1.1.1]pentanyl)-7-chloro-4-methoxyquinolinium iodide (12d).

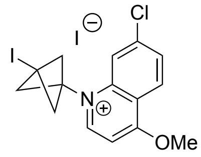

^{13}C NMR (126 MHz, DMSO-d_6)

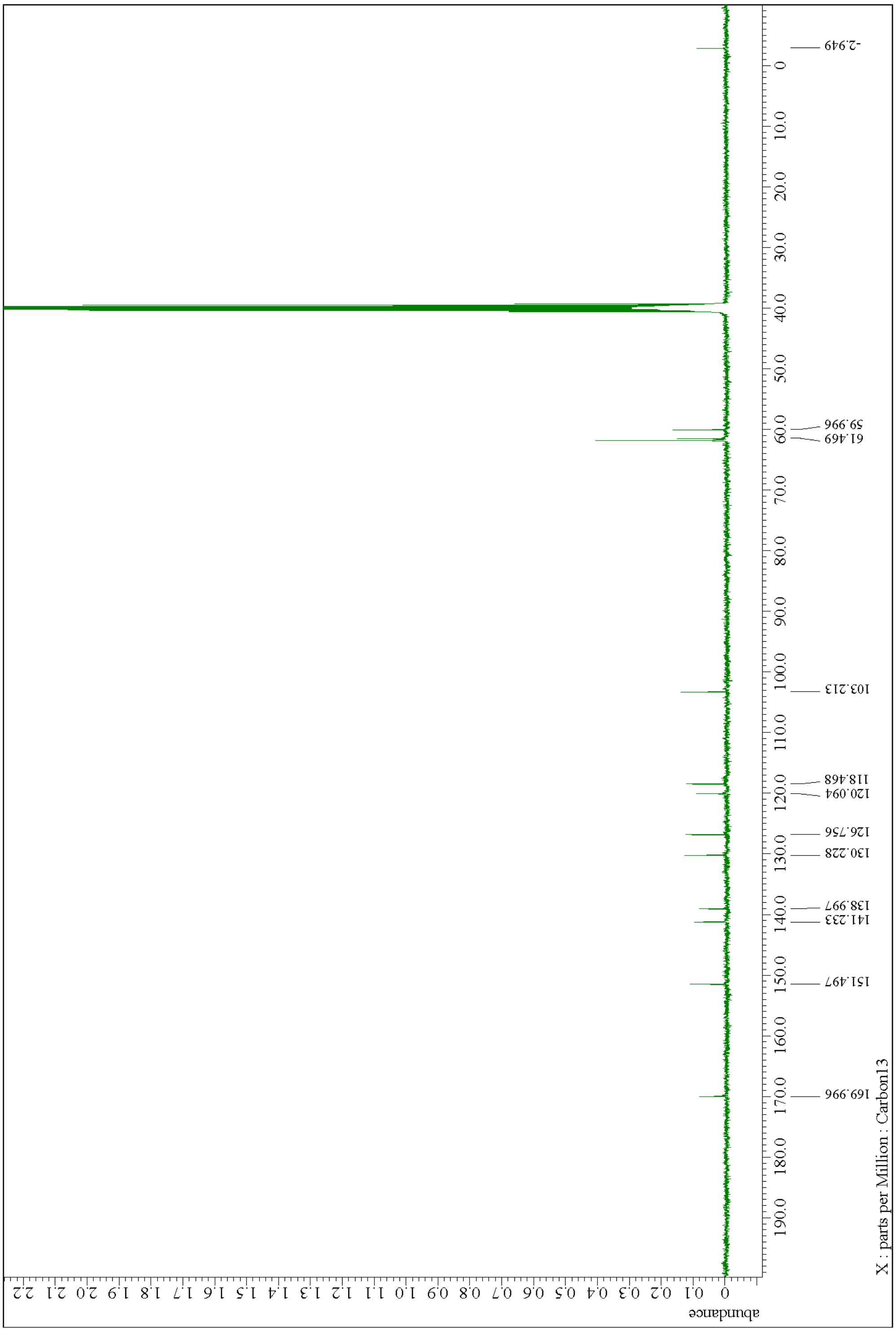

2-(3-Iodobicyclo[1.1.1]pentanyl)isoquinolinium iodide (13a).

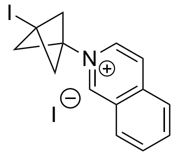

^1H NMR (500 MHz, DMSO-d_6)

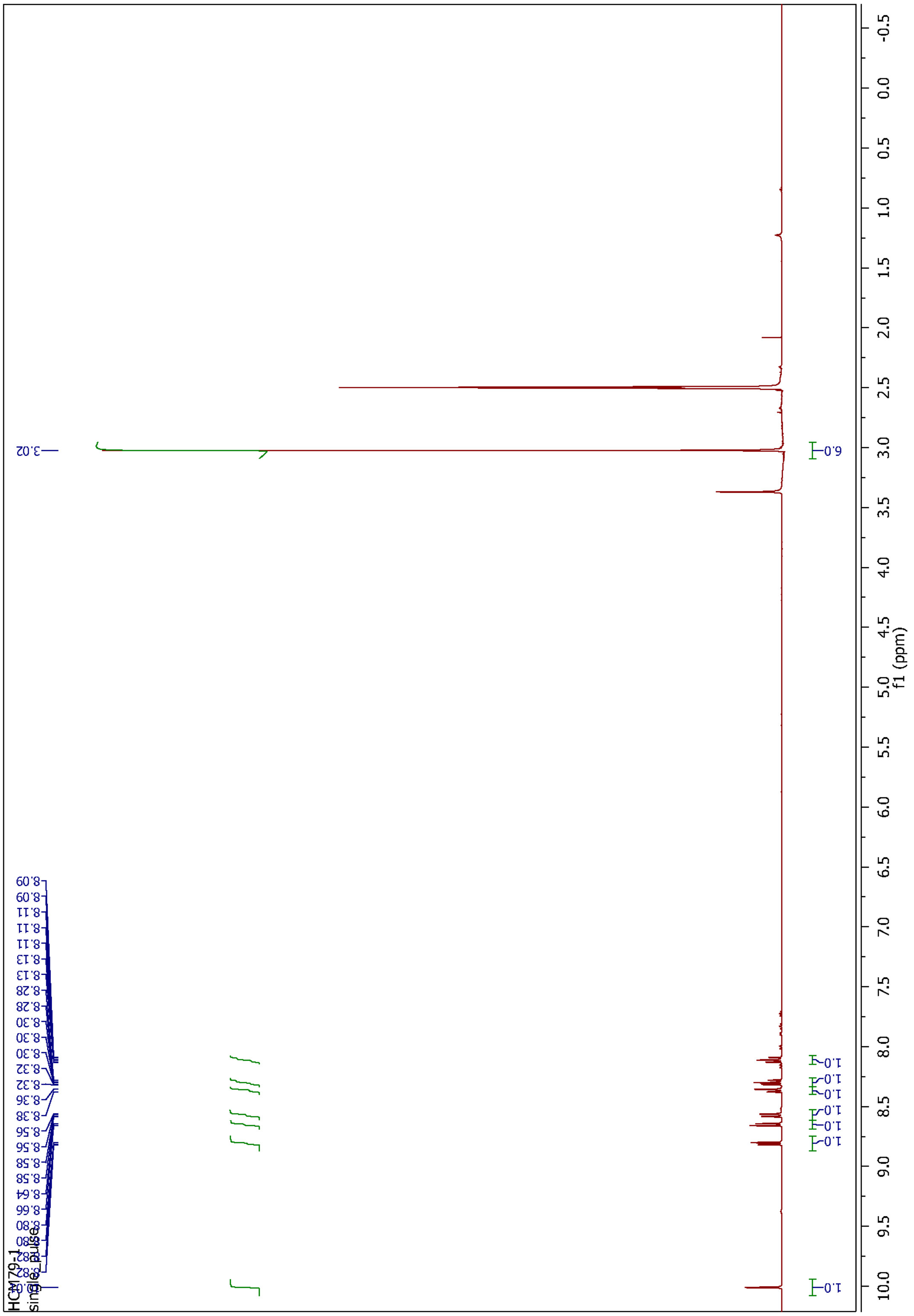

2-(3-Iodobicyclo[1.1.1]pentanyl)isoquinolinium iodide (13a).

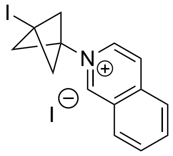

^{13}C NMR (126 MHz, DMSO-d_6)

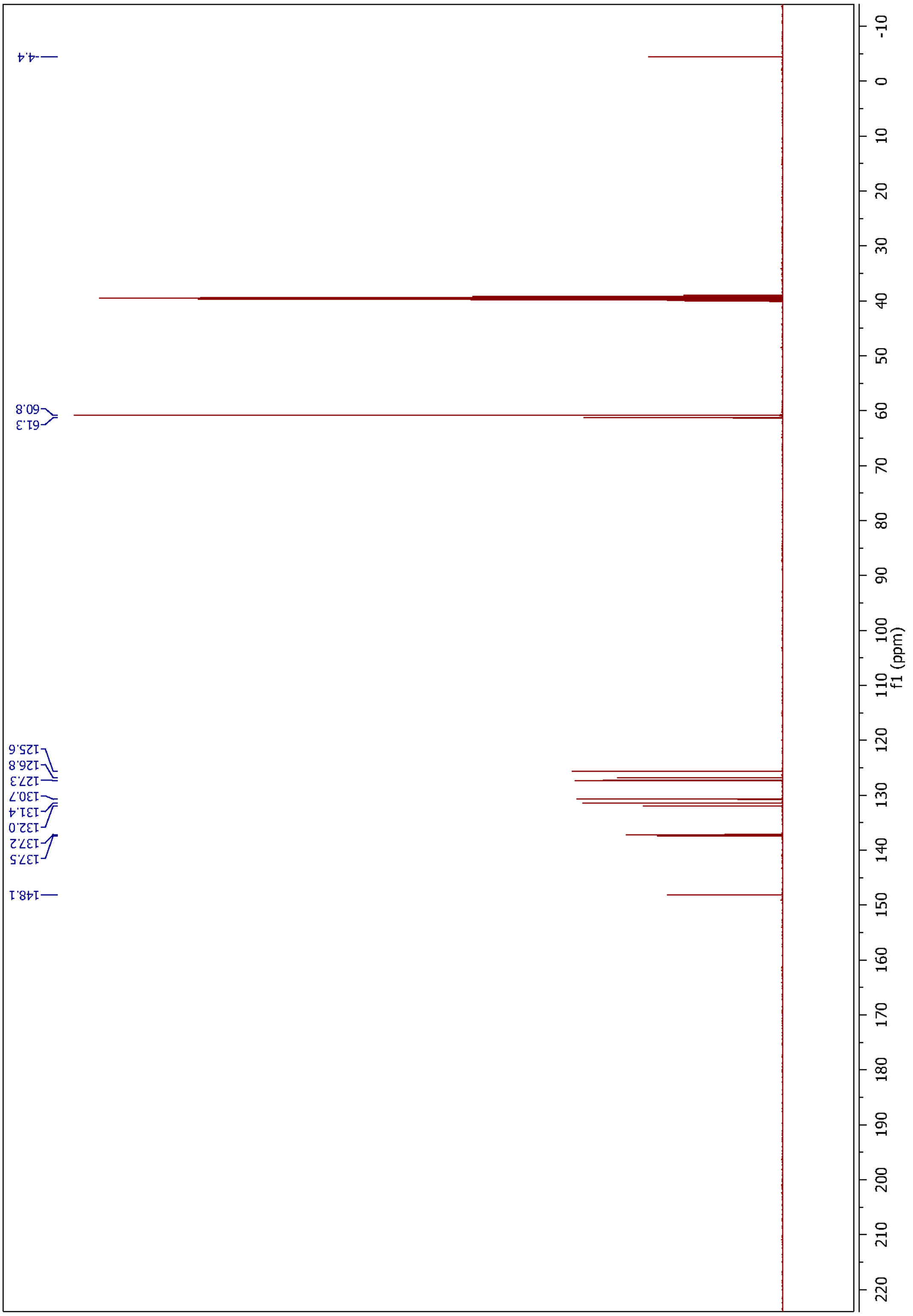

2-(3-Iodobicyclo[1.1.1]pentanyl)-6-methoxyisoquinolinium iodide (13b).

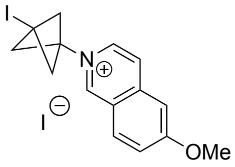

^{1}H NMR (500 MHz, DMSO-d_6)

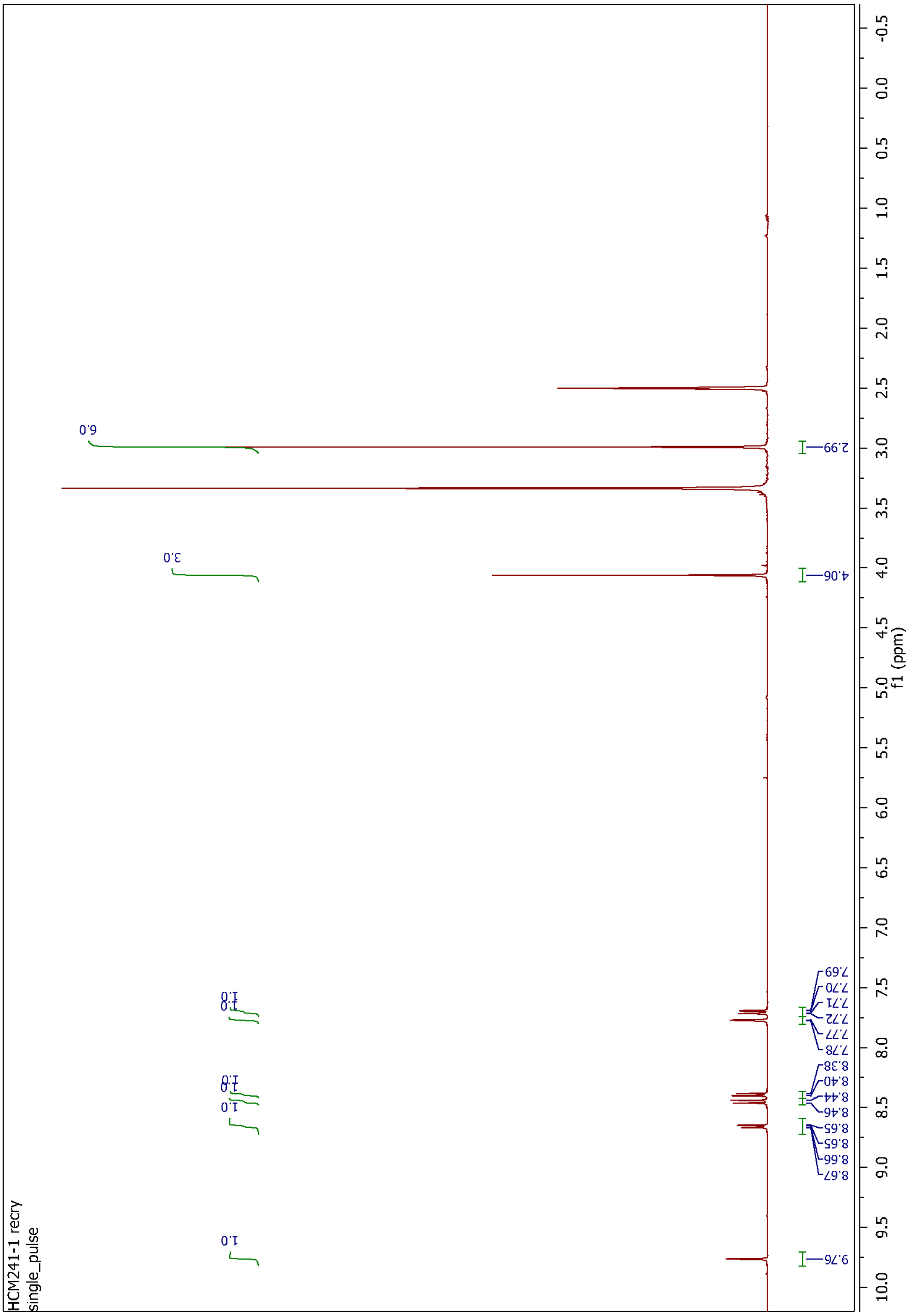

2-(3-Iodobicyclo[1.1.1]pentanyl)-6-methoxyisoquinolinium iodide (13b).

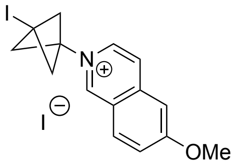

^{13}C NMR (126 MHz, DMSO-d_6)

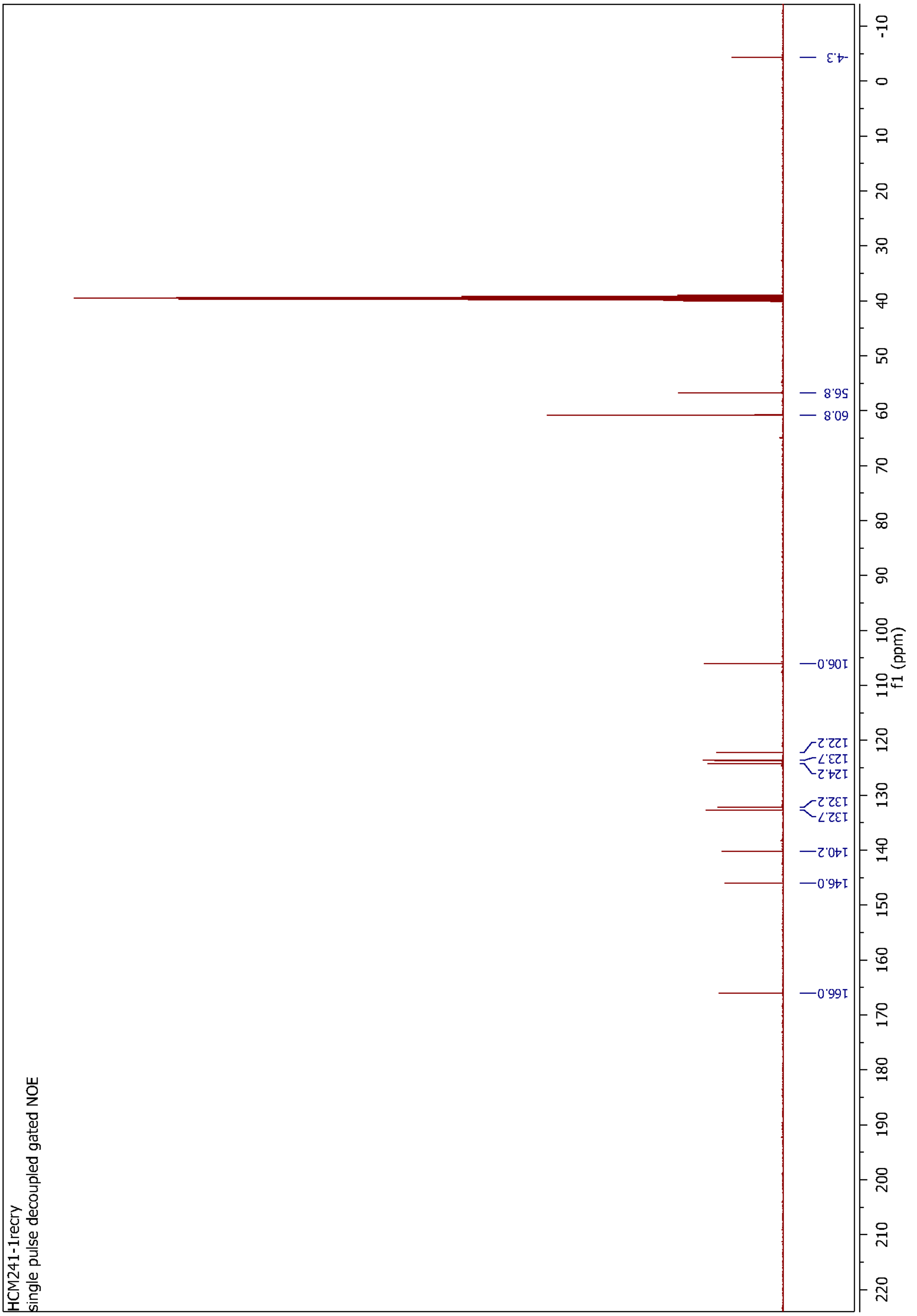

2-(3-Iodobicyclo[1.1.1]pentanyl)-6-methoxy-3-methylisoquinolinium iodide (13c).

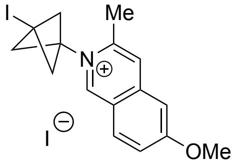

^{1}H NMR (500 MHz, DMSO-d_6)

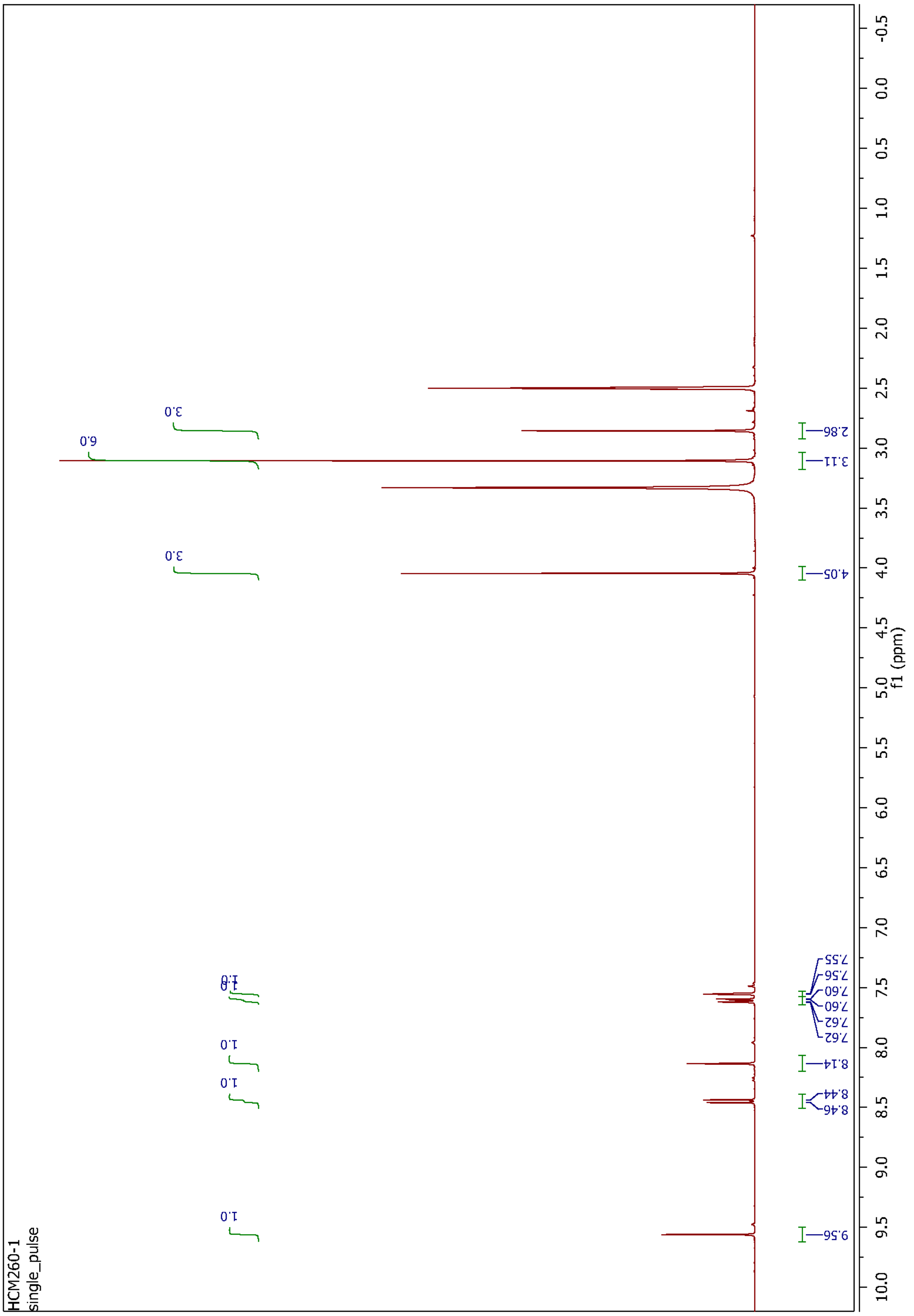

2-(3-Iodobicyclo[1.1.1]pentanyl)-6-methoxy-3-methylisoquinolinium iodide (13c).

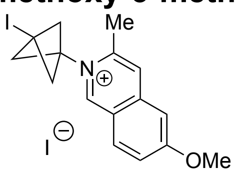

^{13}C NMR (126 MHz, DMSO-d_6)

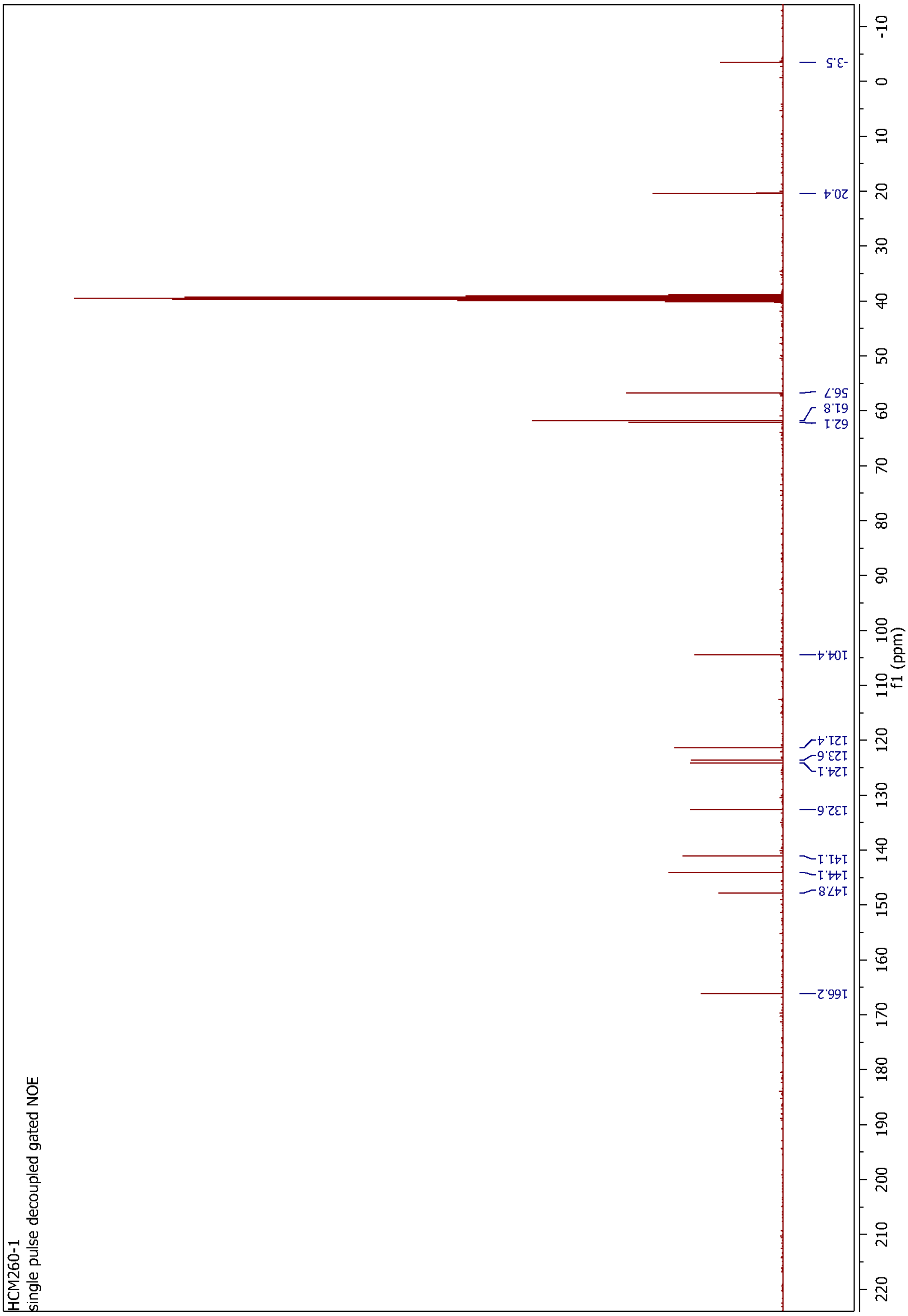

2-(3-Iodobicyclo[1.1.1]pentanyl)-1-methylpyrazolium iodide (14a).

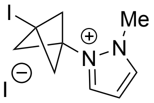

^{1}H NMR (500 MHz, DMSO-d_6)

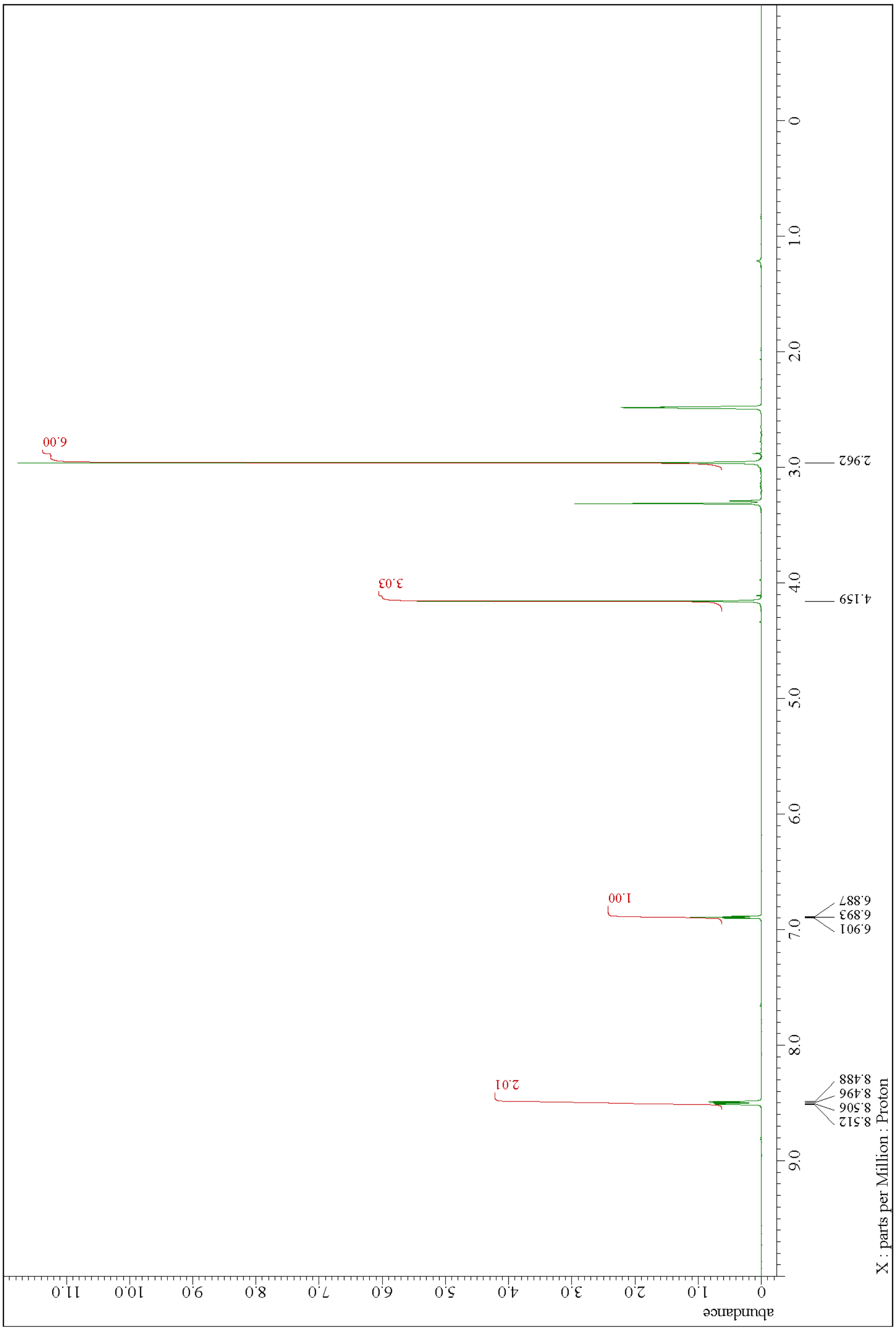

2-(3-Iodobicyclo[1.1.1]pentanyl)-1-methylpyrazolium iodide (14a).

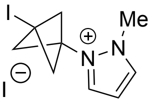

^{13}C NMR (126 MHz, DMSO-d_6)

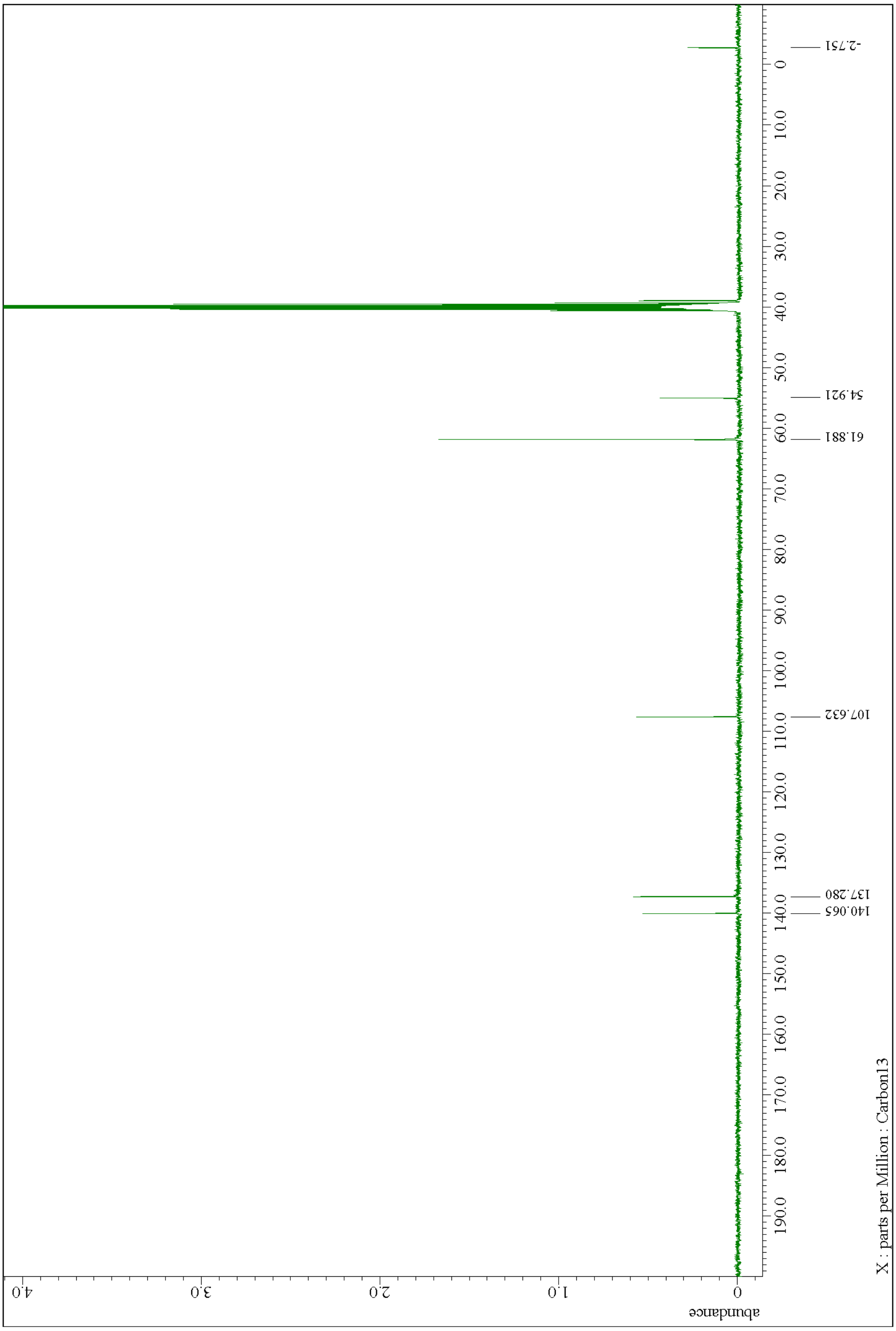

4-Iodo-2-(3-Iodobicyclo[1.1.1]pentanyl)-1-methylpyrazolium iodide (14b).

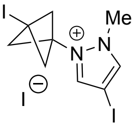

^{1}H NMR (500 MHz, DMSO-d_6)

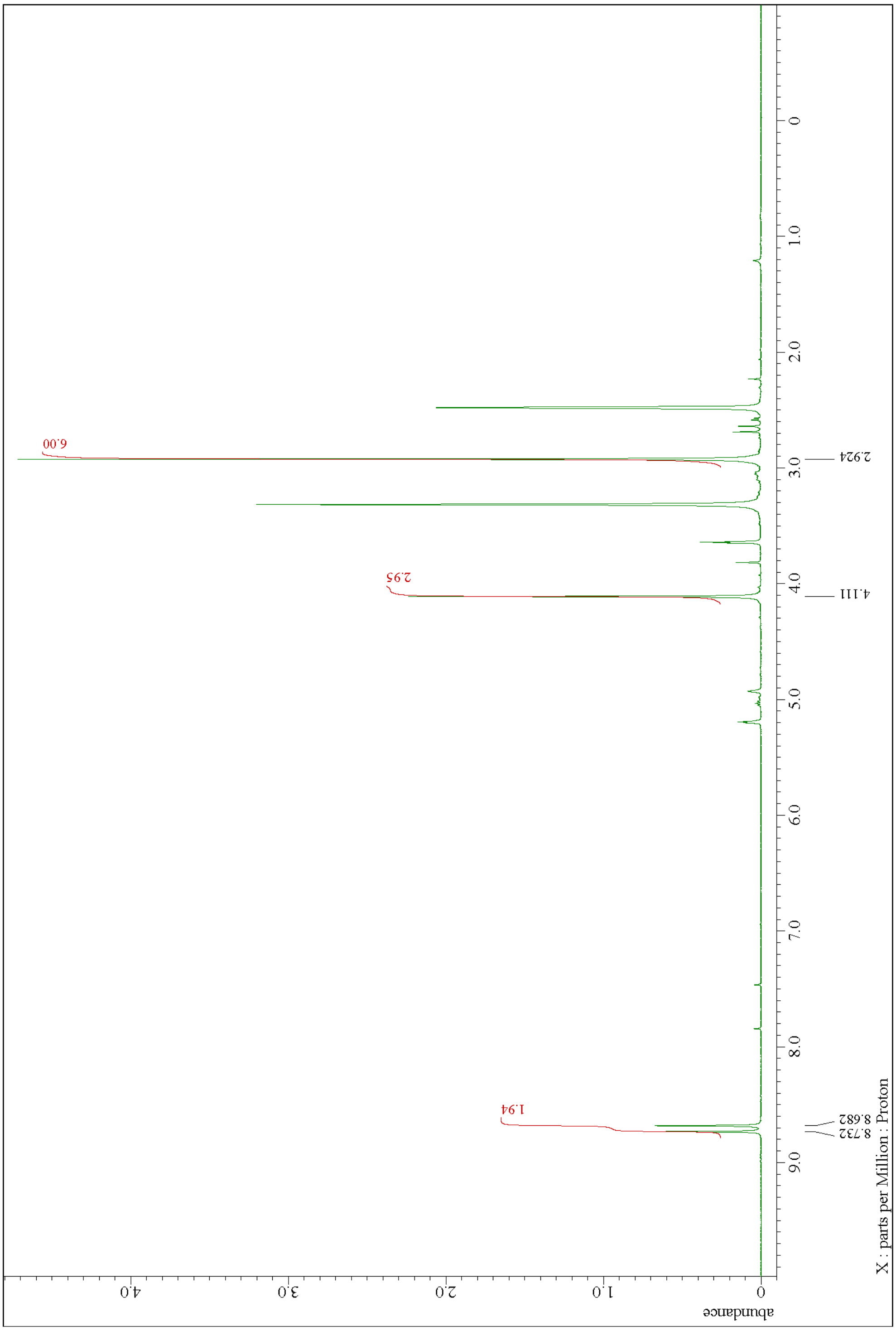

4-Iodo-2-(3-Iodobicyclo[1.1.1]pentanyl)-1-methylpyrazolium iodide (14b).

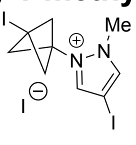

^{13}C NMR (126 MHz, DMSO-d_6)

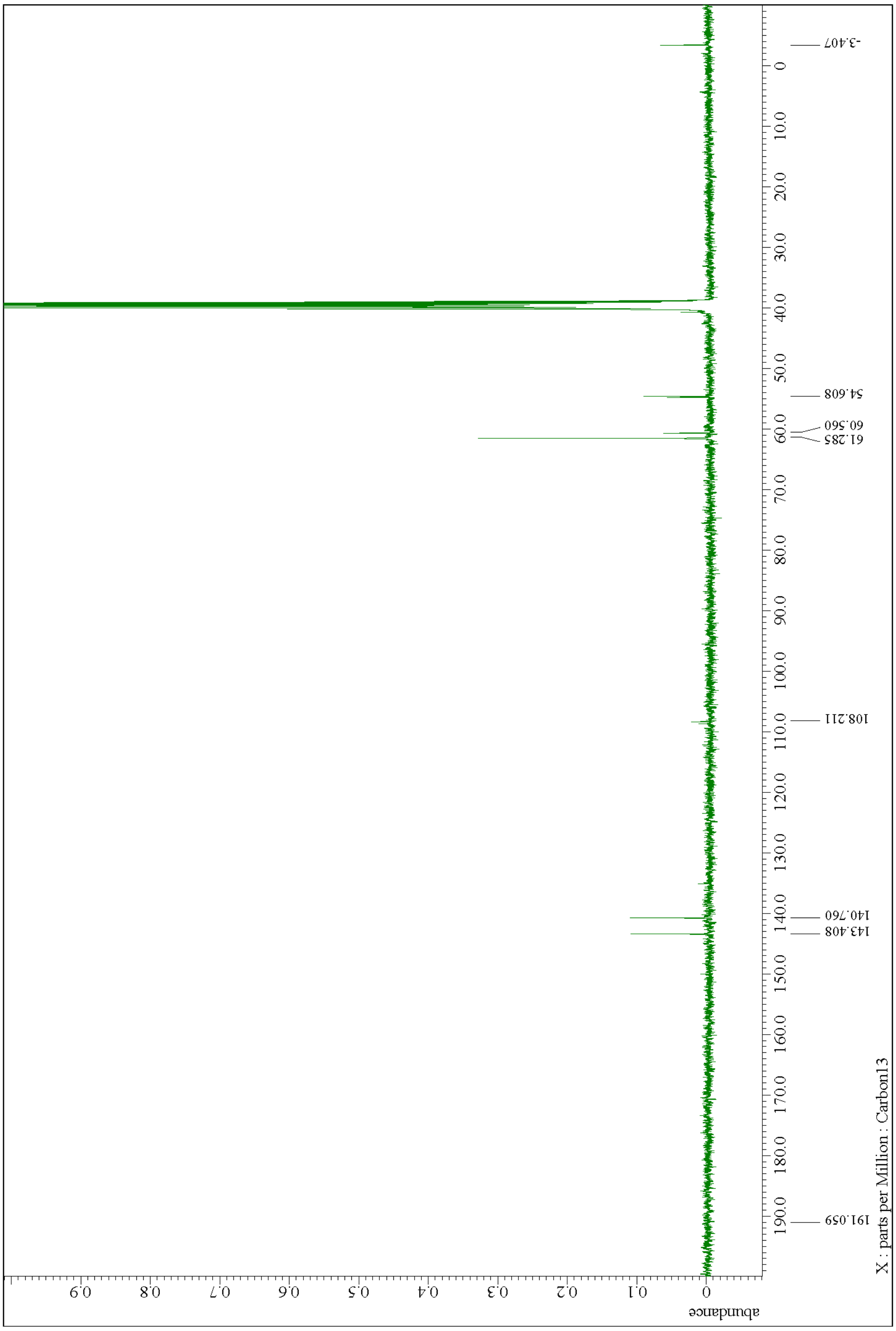

1-(3-Iodobicyclo[1.1.1]pentanyl)pyridin-4-one (15a).

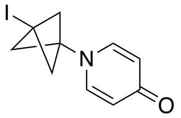

^{1}H NMR (500 MHz, DMSO-d_6)

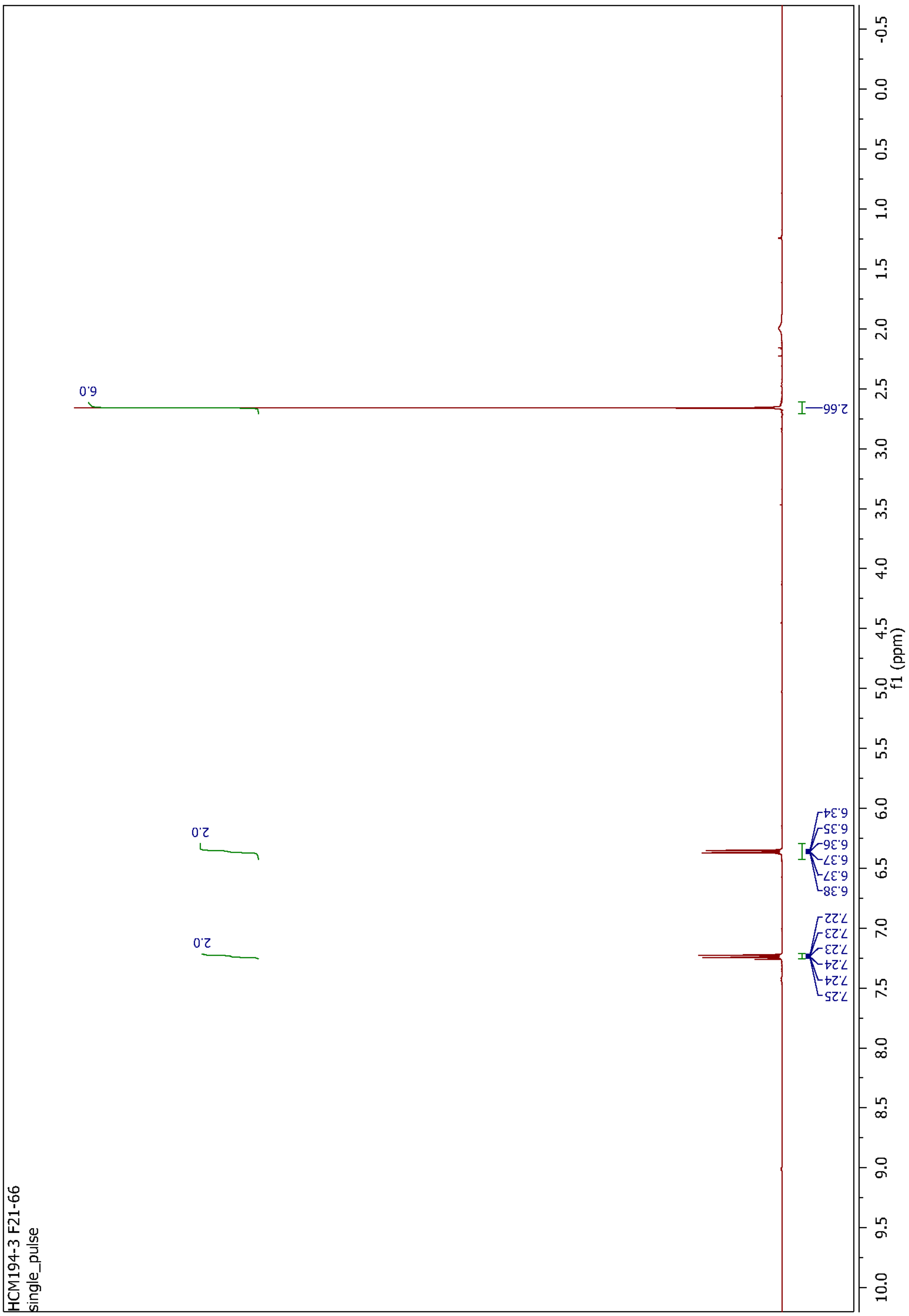

1-(3-Iodobicyclo[1.1.1]pentanyl)pyridin-4-one (15a).

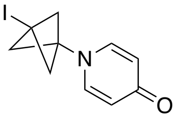

^{13}C NMR (126 MHz, DMSO-d_6)

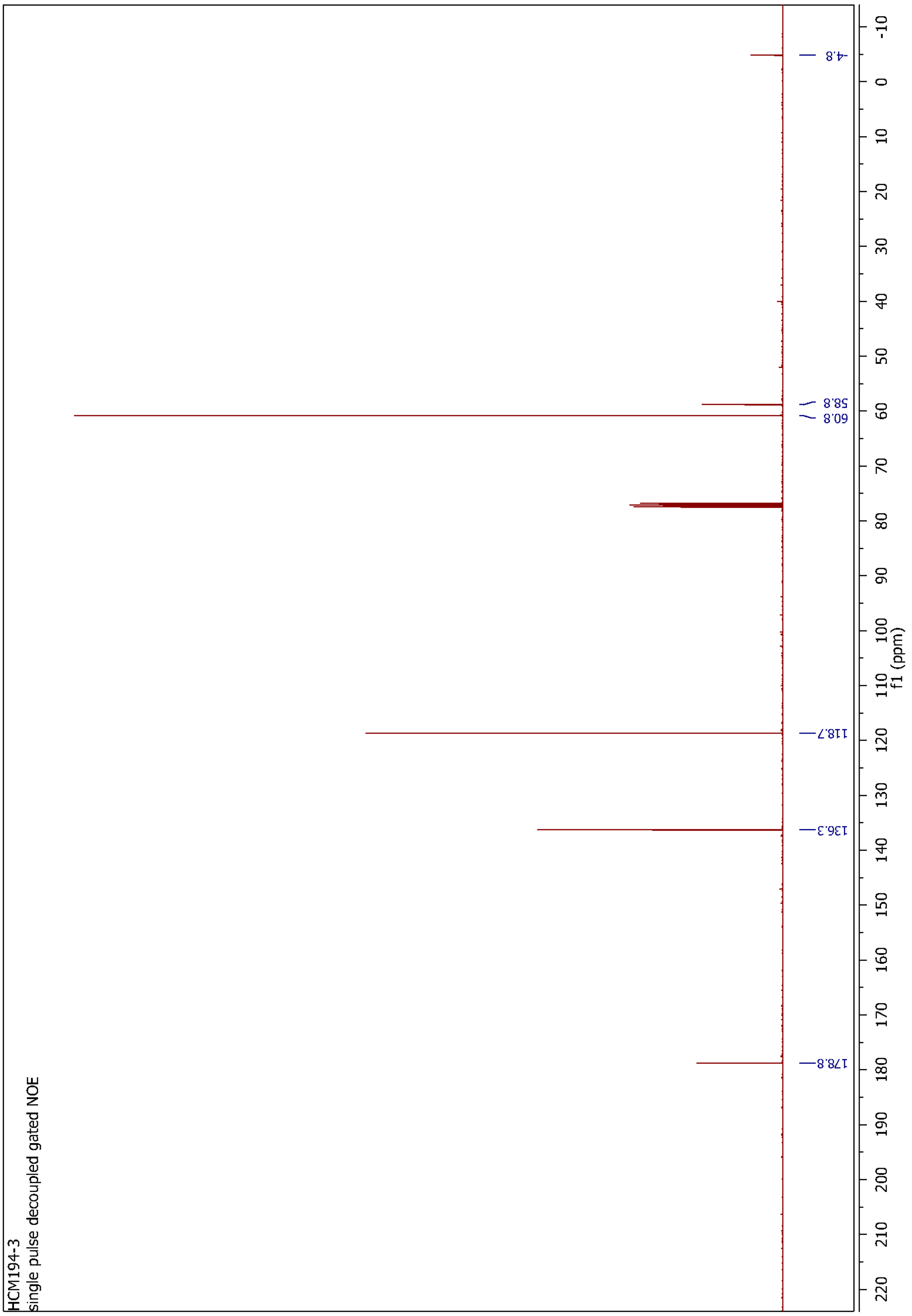

1-Bicyclo[1.1.1]pentanylpyridin-4-one (15b).

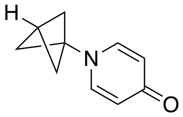

^{1}H NMR (500 MHz, DMSO-d_6)

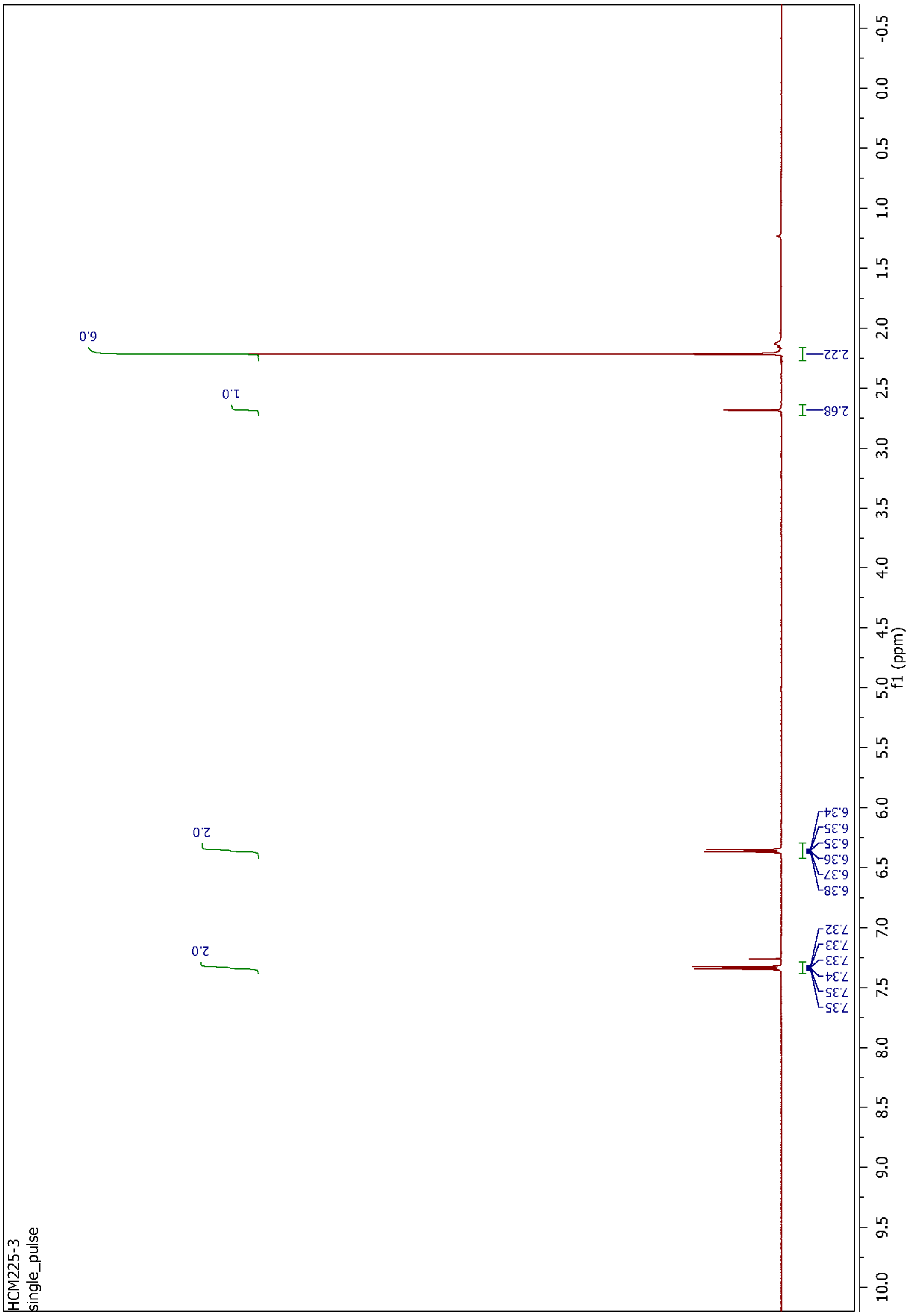

1-Bicyclo[1.1.1]pentanylpyridin-4-one (15b).

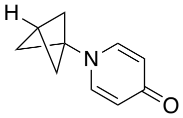

^{13}C NMR (126 MHz, DMSO-d_6)

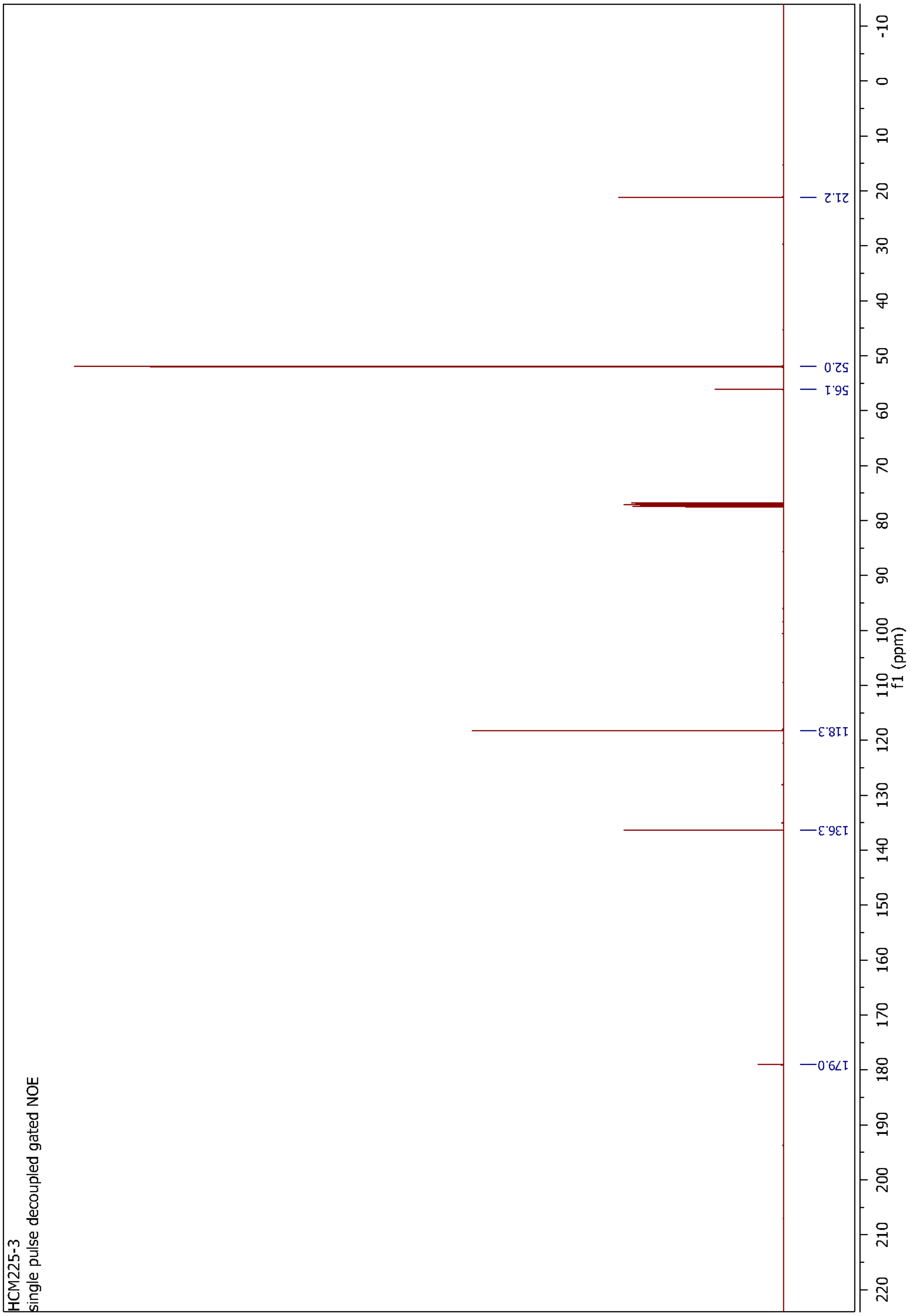

2-(1-(3-Iodobicycloc[1.1.1]pentanyl)pyridine-4-(1H)-ylidene)malononitrile (15c).

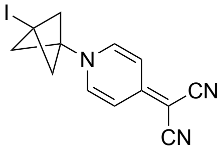

^{1}H NMR (400 MHz, DMSO-d_6)

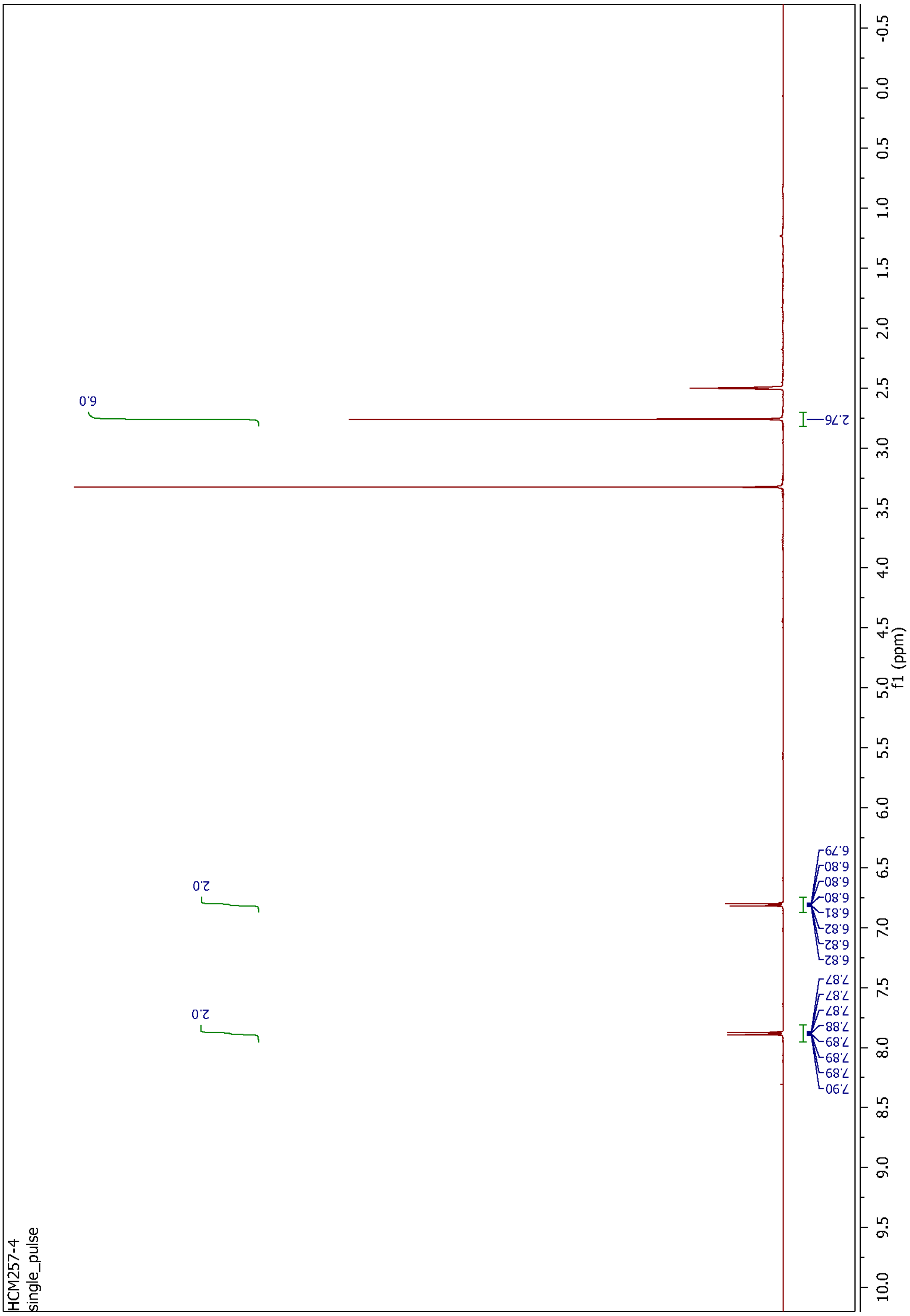

2-(1-(3-Iodobicycloc[1.1.1]pentanyl)pyridine-4-(1H)-ylidene)malononitrile (15c).

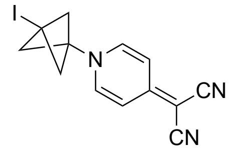

^{13}C NMR (101 MHz, DMSO-d_6)

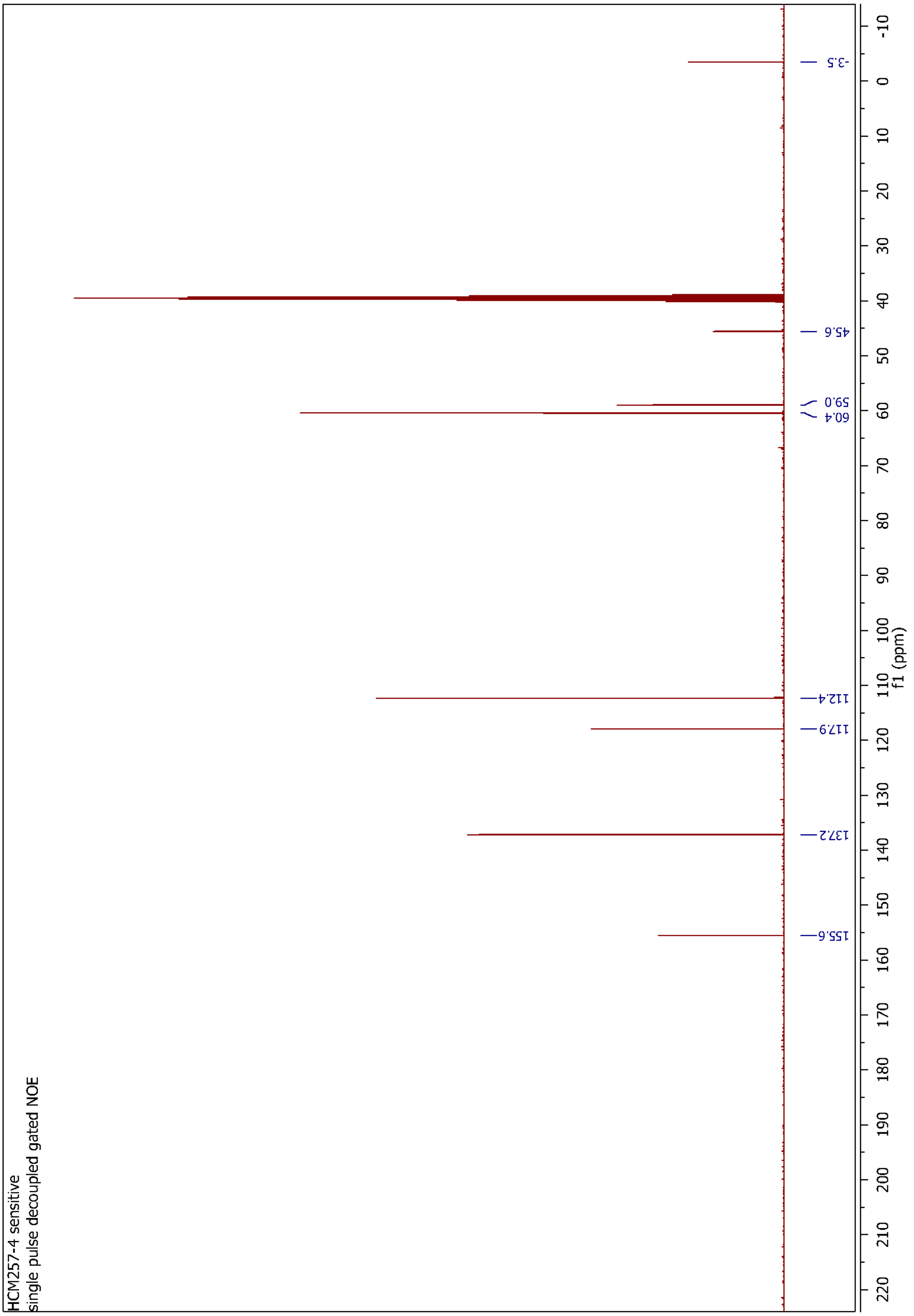

1-(3-Iodobicyclo[1.1.1]pentanyl)quinolin-4-one (16a).

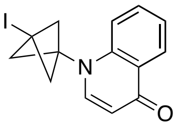

^{1}H NMR (500 MHz, DMSO-d_6)

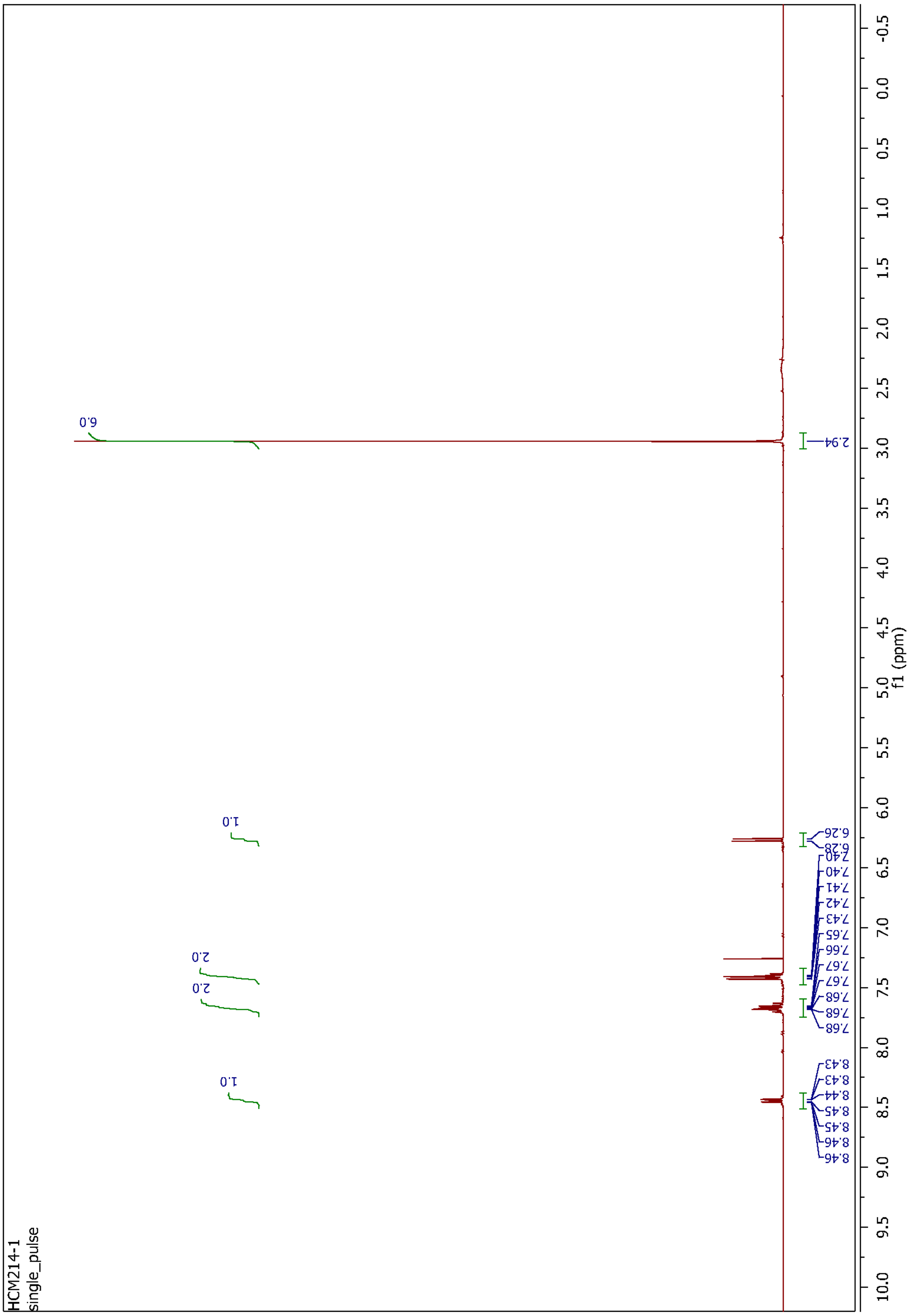

1-(3-Iodobicyclo[1.1.1]pentanyl)quinolin-4-one (16a).

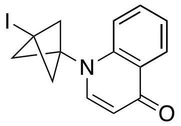

^{13}C NMR (126 MHz, DMSO-d_6)

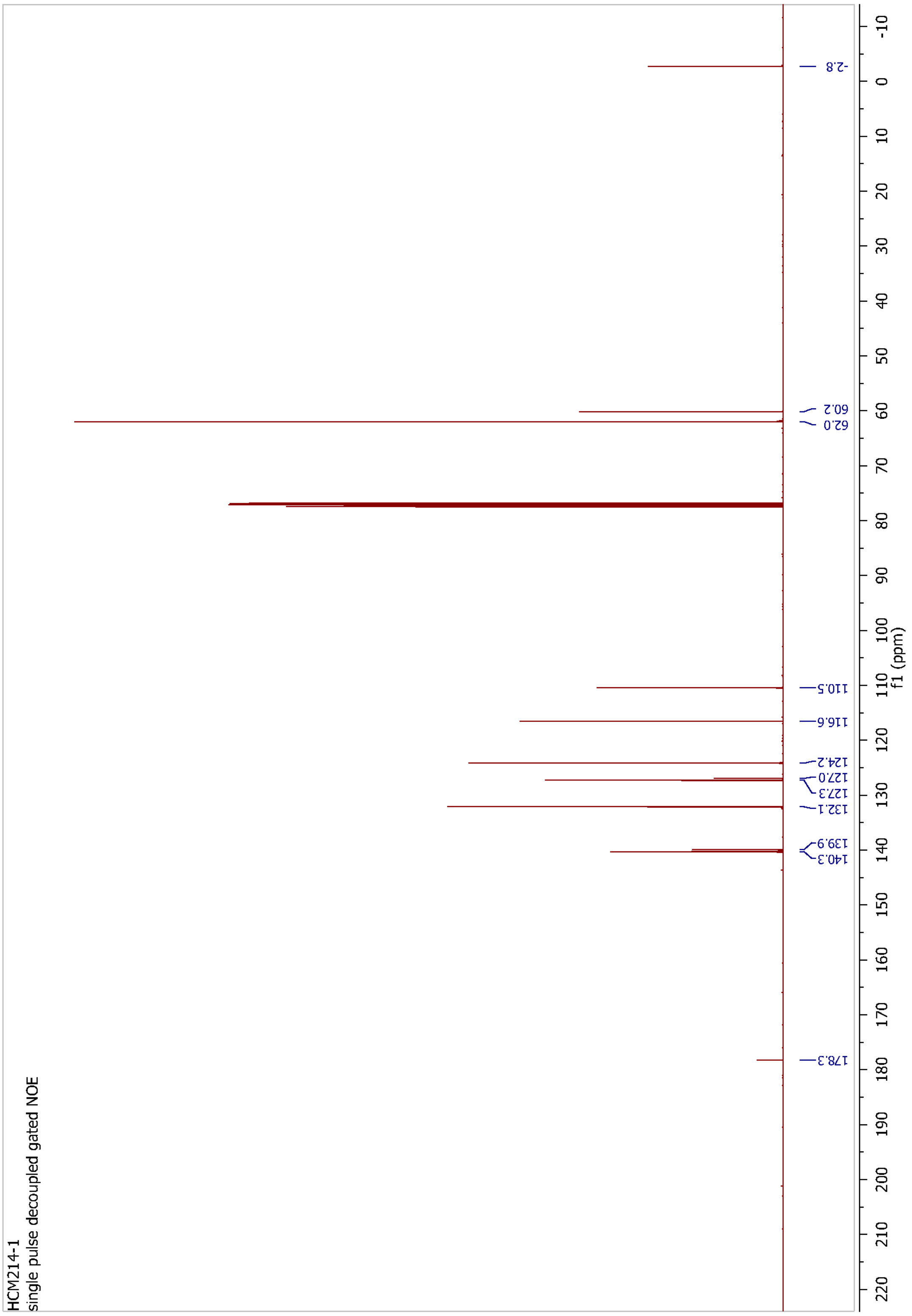

1-Bicyclo[1.1.1]pentanylquinolin-4-one (16b).

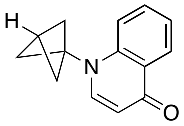

^{1}H NMR (500 MHz, DMSO-d_6)

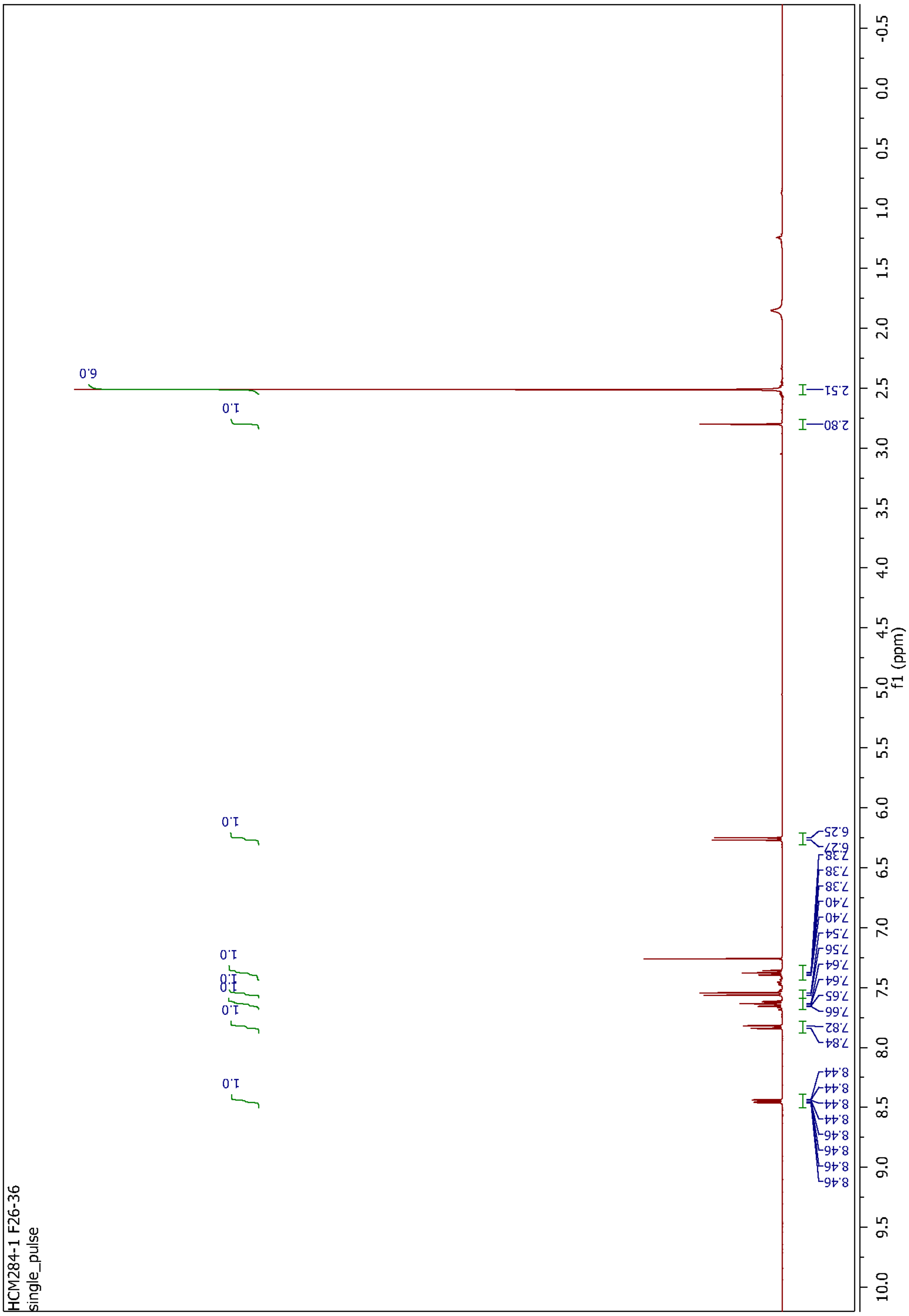

1-Bicyclo[1.1.1]pentanylquinolin-4-one (16b).

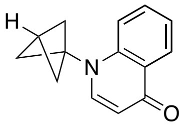

^{13}C NMR (126 MHz, DMSO-d_6)

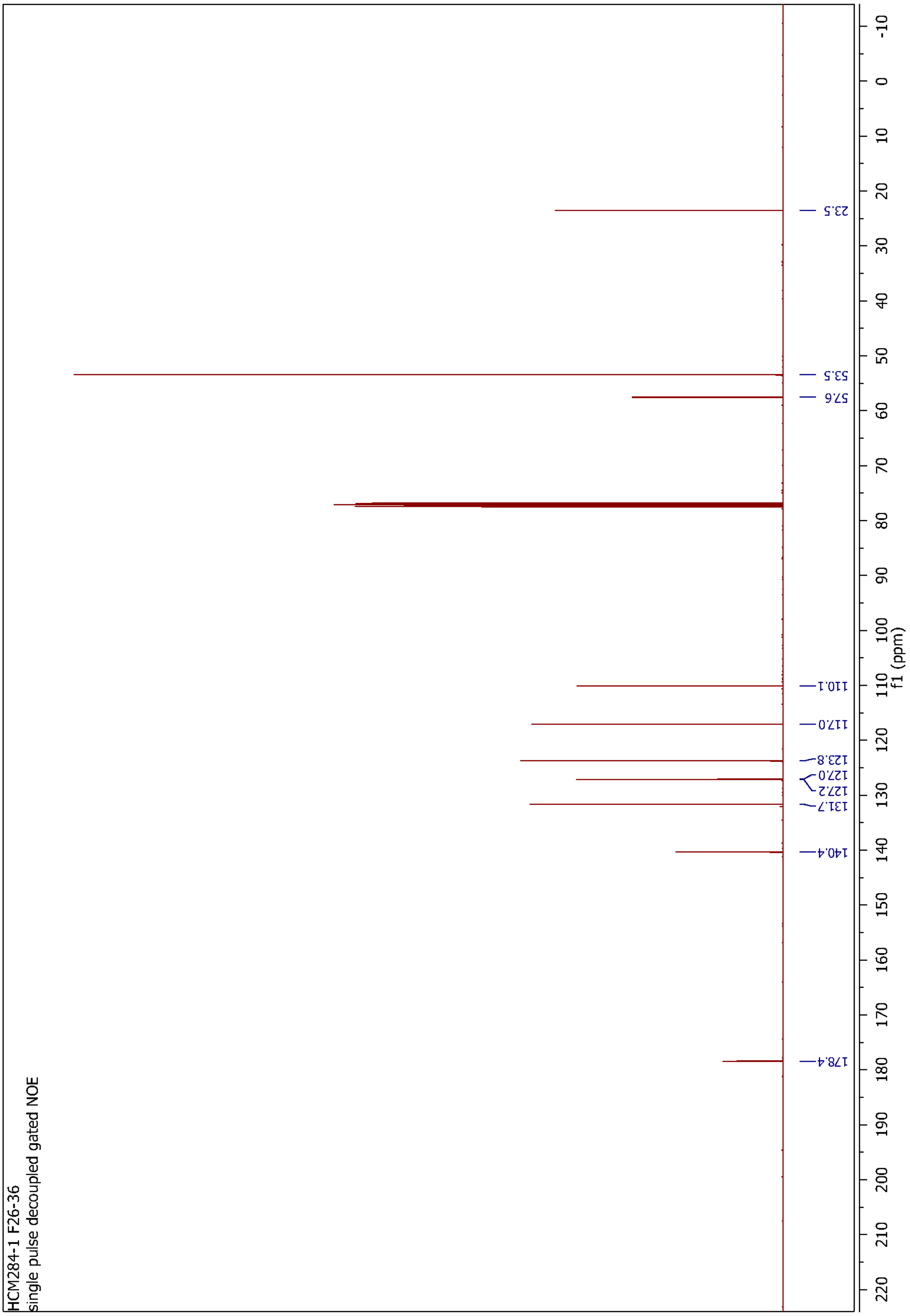

1-(1-(3-Iodobicyclo[1.1.1]pentanyl)-(6-(1H-indol-3-yl)-1,6-dihydropyridin-3-yl)ethenone (17a).

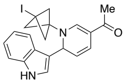

^{1}H NMR (500 MHz, DMSO-d_6)

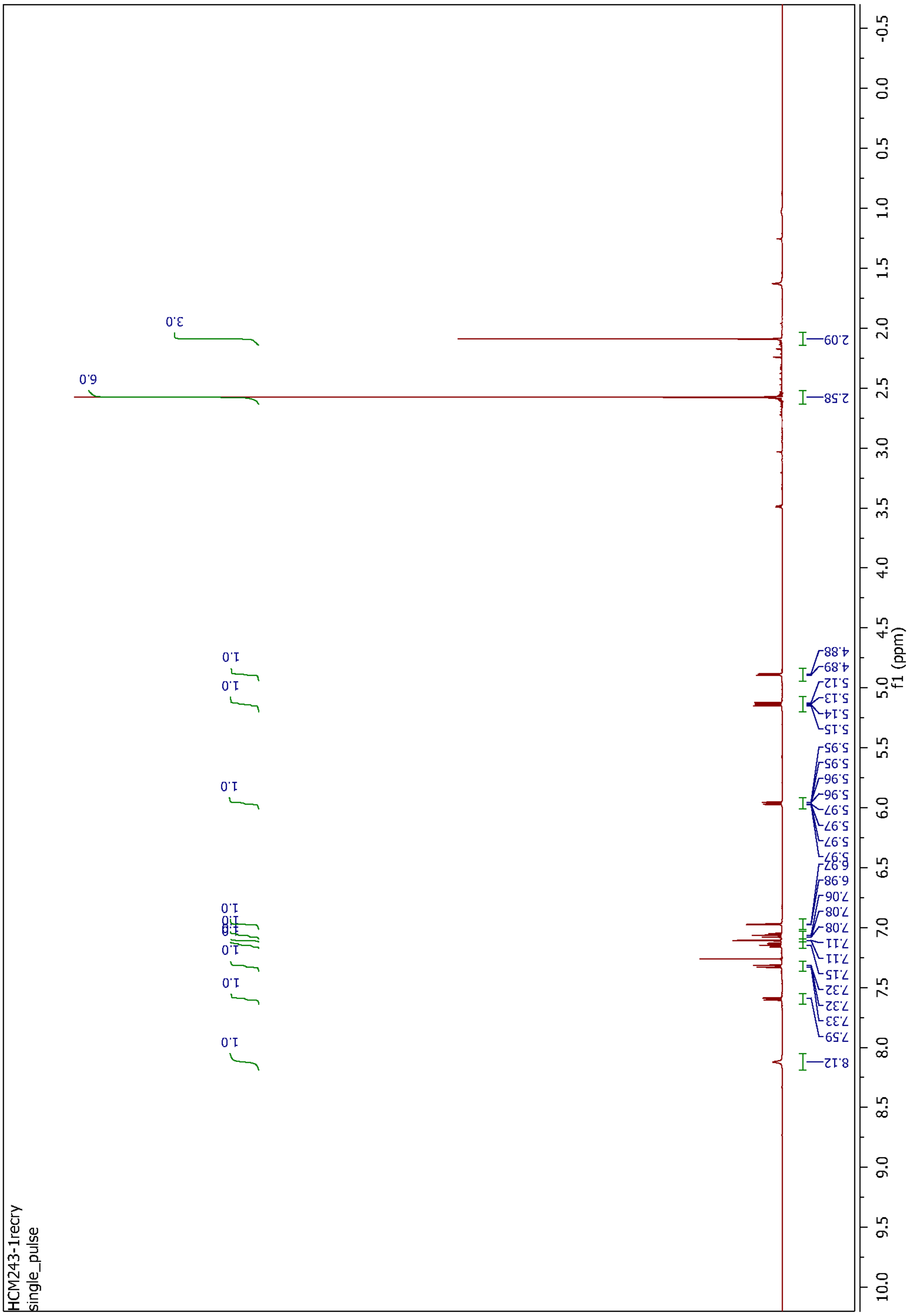

1-(1-(3-Iodobicyclo[1.1.1]pentanyl)-(6-(1H-indol-3-yl)-1,6-dihydropyridin-3-yl)ethenone (17a).

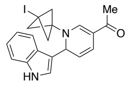

^{13}C NMR (126 MHz, DMSO-d_6)

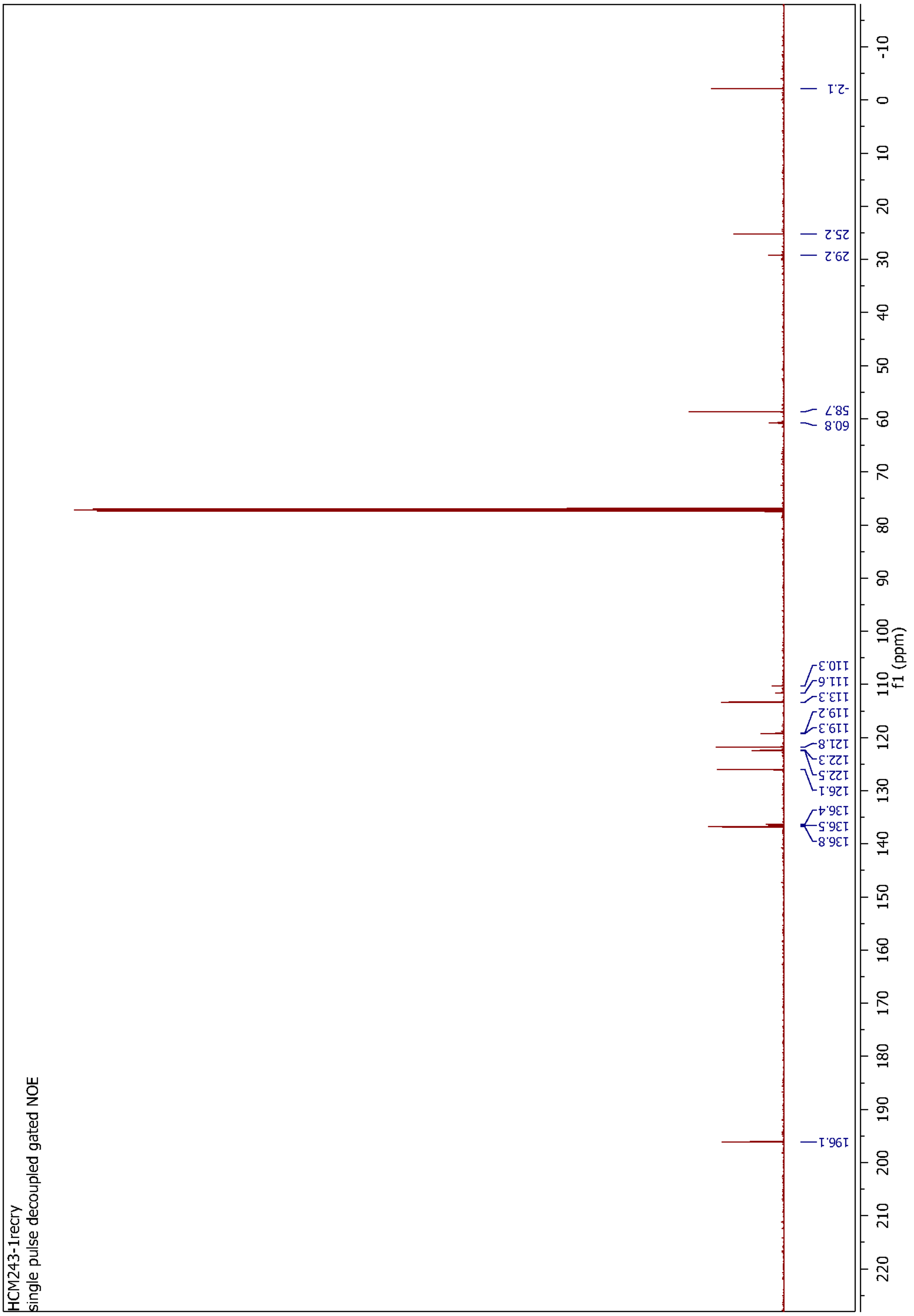

1-(1-(3-iodobicyclo[1.1.1]pentanyl)-(6-(2-methyl-1H-indol-3-yl)-1,6-dihydropyridin-3-yl)ethenone (17b).

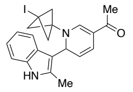

^{1}H NMR (500 MHz, DMSO-d_6)

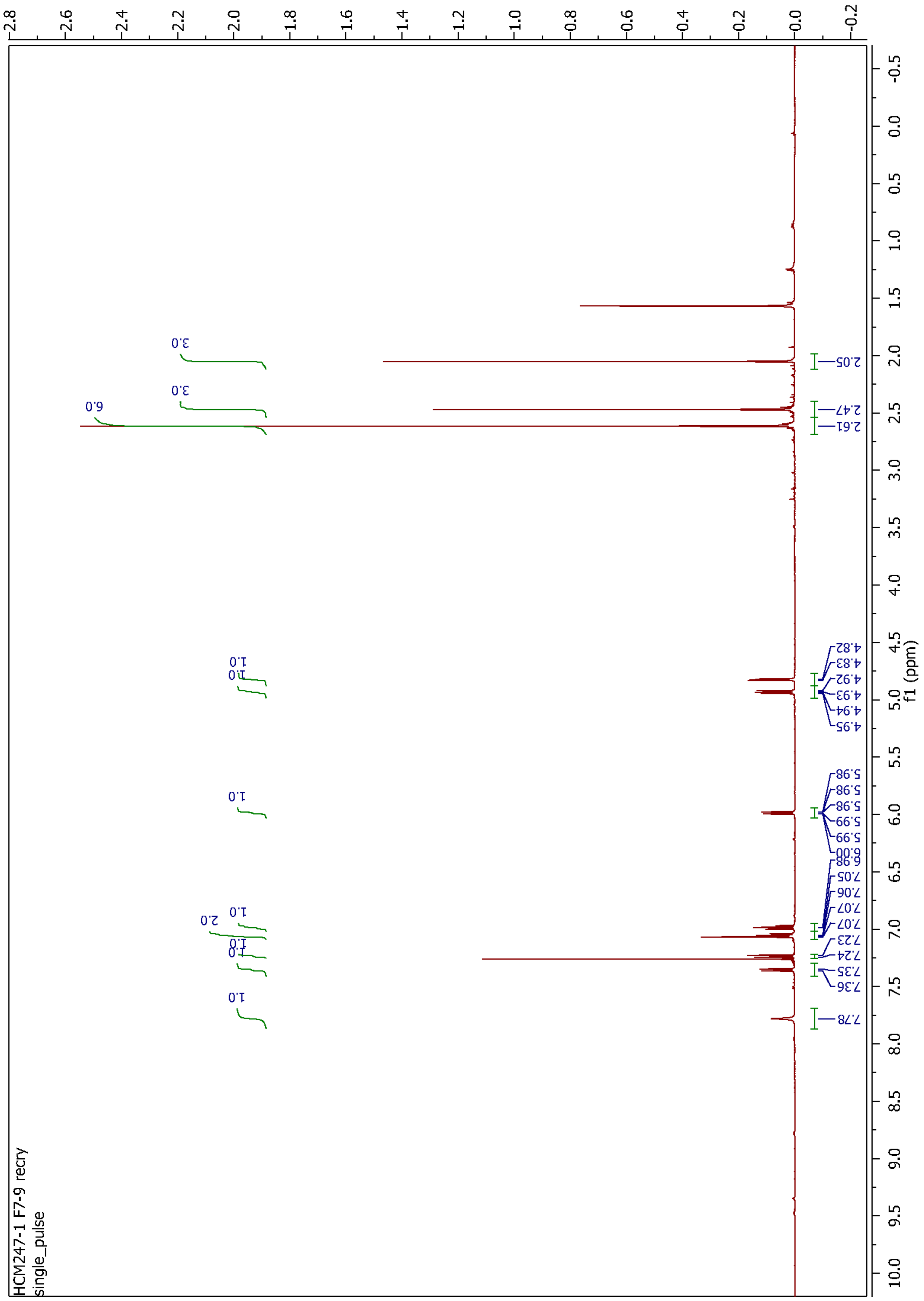

1-(1-(3-iodobicyclo[1.1.1]pentanyl)-(6-(2-methyl-1H-indol-3-yl)-1,6-dihydropyridin-3-yl)ethenone (17b).

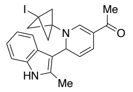

^{13}C NMR (126 MHz, DMSO-d_6)

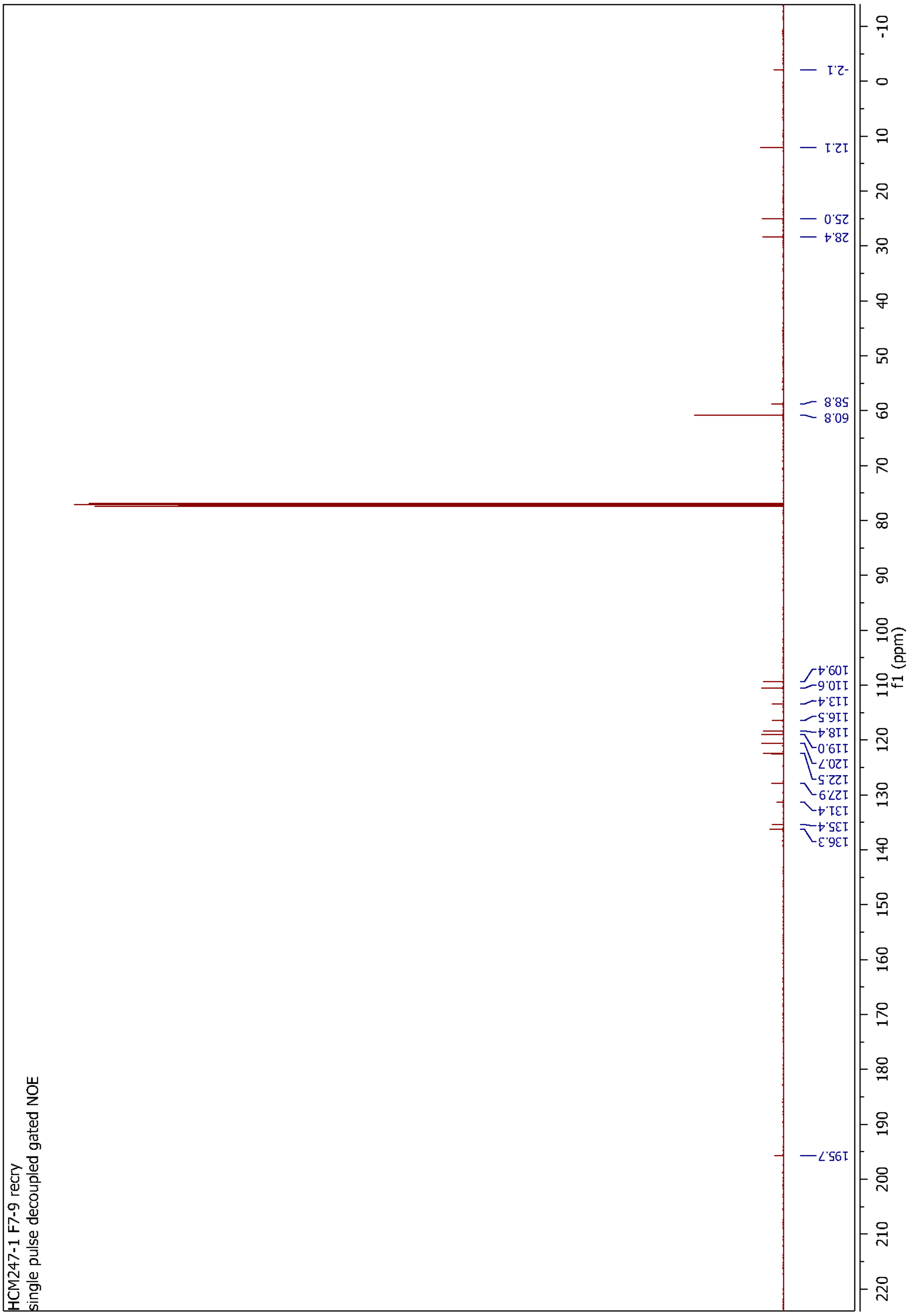

Supplement: Supplementary file 1 [file jo5c00565_si_001.pdf]
